# C‑20 Diterpenoid Alkaloids with PD-L1 Inhibitory Activity from the Roots of *Aconitum coreanum*

**DOI:** 10.1021/acsomega.6c05802

**Published:** 2026-09-22

**Authors:** Kei-Fong Ng, Xiao-Qian Li, Hao-Ming Xiong, Lin Chen, Xi Chen, Li-Ping Bai, Wei Zhang, Ancheng C. Huang, Zhi-Hong Jiang, Guo-Yuan Zhu

**Affiliations:** † State Key Laboratory of Mechanism and Quality of Chinese Medicine & Faculty of Chinese Medicine, Macau Institute for Applied Research in Medicine and Health, 58816 Macau University of Science and Technology, Taipa, Macau 999078, People’s Republic of China; ‡ Zhuhai MUST Science and Technology Research Institute, Macau University of Science and Technology, Zhuhai 519000, People’s Republic of China; § Shenzhen Key Laboratory of Plant Genetic Engineering and Molecular Design, SUSTech−PKU Institute of Plant and Food Science, Department of Biology, School of Life Sciences, Southern University of Science and Technology, Shenzhen 518000, People’s Republic of China

## Abstract

Fifteen previously unreported diterpenoid alkaloids, named aconicorines A-O (**1**–**15**), and 13 known diterpenoid alkaloids (**16**–**28**) were isolated from the roots of *Aconitum coreanum*. Their structures and configurations were determined by comprehensive spectroscopic analysis including HRMS, NMR, and single-crystal X-ray diffraction. Structurally, all new compounds are classic C20-hetisine-type diterpenoid alkaloids with various oxidation sites. Among them, aconicorines A-D (**1**–**4**) are unique C20-diterpenoid alkaloids containing a rare sulfonate group. Bioassay results showed that **1**, **7**, **13**–**17**, and **20** dose-dependently inhibited the PD-L1 protein expression at 2.5–20 μM in MDA-MB-231 cells. Furthermore, immunofluorescence staining confirmed the inhibitory effect of **7**, **14**, **15**, and **17** on PD-L1 expression. These findings first demonstrated the effects of C20-diterpenoid alkaloids on PD-L1 expression and suggested that C20-diterpenoid alkaloids are a new type of PD-L1 modulators.

## Graphical abstract

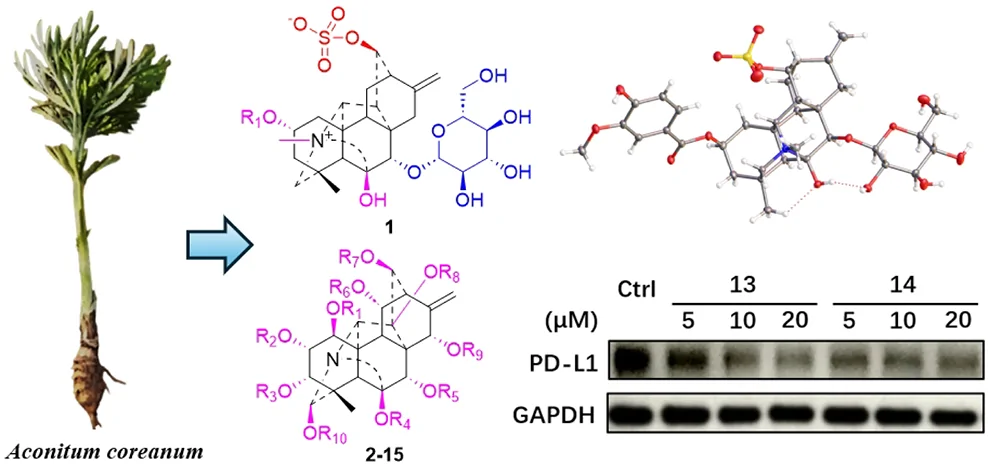

## Introduction

Programmed death-ligand 1 (PD-L1), an immunomodulatory transmembrane protein, interacts with its receptor PD-1 on T cells to help control immune activity and maintain immune tolerance.^1^ In the tumor microenvironment, PD-L1 is highly expressed in cancer cells and achieves immune escape after binding to PD-1. Blocking the binding of PD-L1 to PD-1 can reactivate T cells to kill tumor cells.^2^ To date, a large amount of clinical data have demonstrated the good efficacy of PD-L1 inhibitors, specifically PD-L1 monoclonal antibodies, as immunotherapies in various cancers.^3^ However, PD-L1 monoclonal antibodies still face limitations in cancer treatment, including adverse immune responses, poor oral bioavailability, and high production costs.^4^ In recent years, the development of small-molecule PD-L1 inhibitors has attracted widespread attention and has become a promising field of cancer immunotherapy.^5^ Natural products, as an important source of small-molecule drugs, offer various compounds with PD-L1 inhibitory activities including diterpenoids, triterpenoids, flavonoids, and alkaloids.^6^ In our recent work, oridonin, a natural diterpenoid, was identified as a potential PD-L1 inhibitor capable of boosting antitumor immunity.^7^

The genus *Aconitum* (Ranunculaceae), comprising over 300 species globally, represents one of the most prominent resources for bioactive diterpenoid alkaloids.^8^ In last 5 years, various C19- and C20-diterpenoid alkaloids with aconitine-, hetisine-, denudatine-, napelline-, and lycoctine-type skeletons were reported from *A. heterophyllum*,^9^ *A. carmichaeli* *i*,^10^ *A. gymnandrum*,^11^ *A. nagarum*,^12^ *A. pendulum*,^13^ and *A. refractum*.^14^ These metabolites have been associated with multiple bioactivities, including anti-inflammatory,^11^ analgesic,^10^ antiplasmodial,^15^ insecticidal activities,^16^ NLRP3 inflammasome-inhibitory,^10^ and autophagy-inducing effects.^14^ Among *Aconitum* species, *A. coreanum* is an important medicinal plant due to the long-standing traditional medicinal use and its diverse diterpenoid alkaloids. *A. coreanum* is widely distributed in northeast China, Mongolia, Korea, and the Russian Far East.^17^ The roots of *A. coreanum* are processed and used as an herbal medicine, called Guanbaifu in China or Korean monkshood in Korea, to treat stroke, facial paralysis, epilepsy, and migraine.^18^ A variety of chemical constituents have been isolated from *A. coreanum*, including diterpenoid alkaloids,^17,19–21^ phenolic acids,^22–24^ phytosterols,^17,22^ fatty acids,^17,22^ and polysaccharides.^25–28^ These compounds have demonstrated a range of significant biological activities such as antiarrhythmia,^17,29,30^ anti-inflammation,^18,27^ antioxidation,^18,28^ antitumor,^26,31^ antiplatelet aggregation,^23^ and insecticidal activities.^32^ Guanfu base A, also known as acehytisine, is a major hetisine-type diterpenoid alkaloid from *A. coreanum* and has antiarrhythmic effects.^29^ Our preliminary experiment results showed that guanfu base A had an inhibitory effect on PD-L1 expression. To explore more alkaloids with PD-L1 inhibitory activity, LC-MS-guided alkaloid separation was performed on the root of *A. coreanum* in this study and resulted in the isolation of 15 previously unreported (**1**–**15**) and 13 known diterpenoid alkaloids (**16**–**28**). The effects of the isolated compounds on PD-L1 expression were then evaluated.

## Results and Discussion

The ethanol extract of *A. coreanum* roots (10 kg) was partitioned sequentially with petroleum ether (PE), ethyl acetate (EA), and *n*-butanol. These extracts were repeatedly chromatographed on silica gel CC, MHPLC, and HPLC, yielding 15 new compounds, named aconicorines A–O (**1**–**15**), along with 13 known compounds (**16**–**28**) (Figure 1). All known compounds are C20-diterpenoid alkaloids, which were identified by their HRMS and NMR data as zeravshanisine (**16**),^33^ coreanine D (**17**),^19^ guanfu base G (**18**),^34^ guanfu base A (**19**),^30^ guanfu base F (**20**),^35^ guanfu base Q (**21**),^36^ guanfu base Z (**22**),^37^ acorine (**23**),^38^ hetisinone (**24**),^39^ guanfu base H (**25**),^40^ guanfu base A_1_ (**26**),^36^ guanfu base P (**27**),^35^ and coreanine A (**28**).^19^

**Figure 1:**
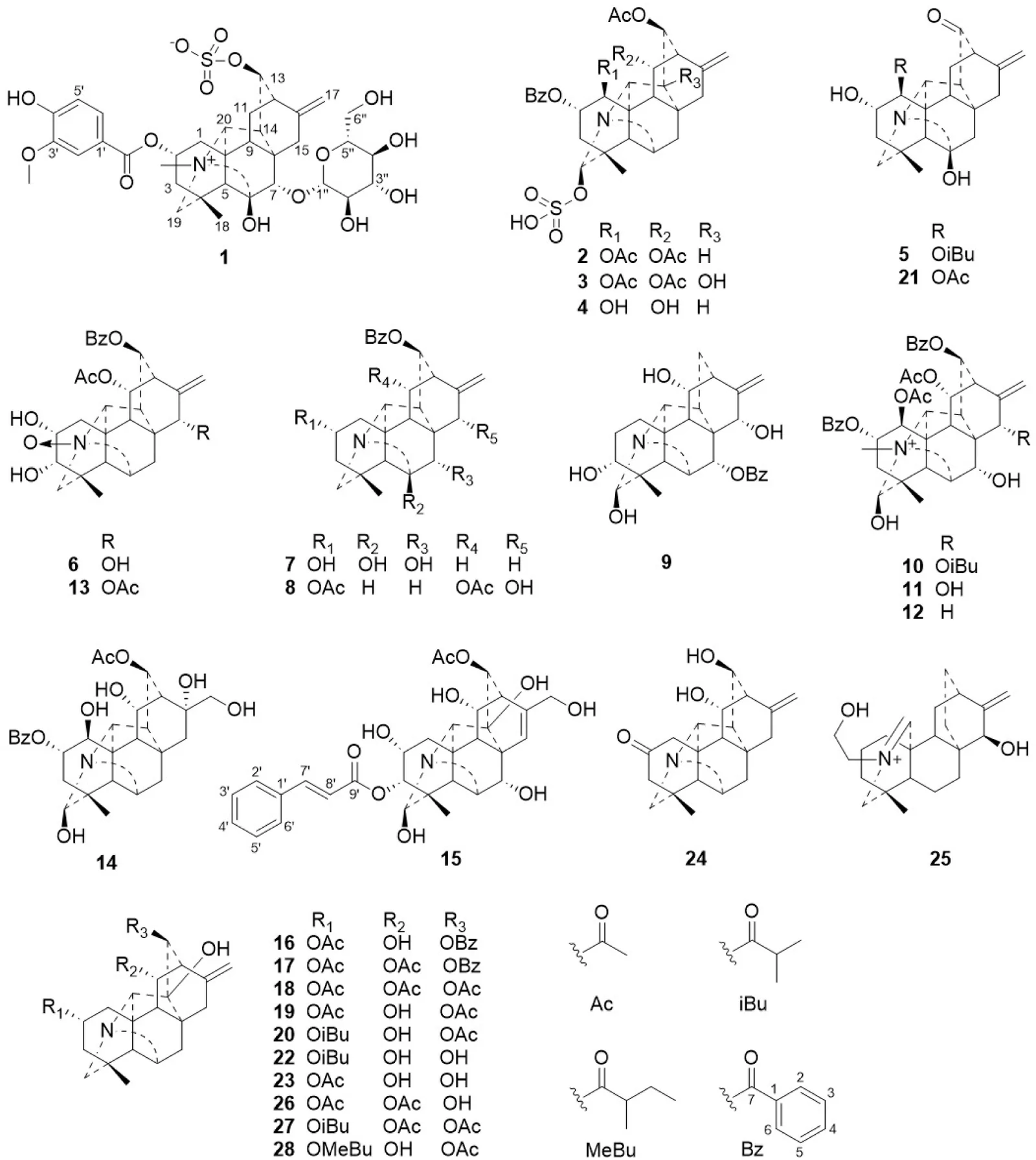
Chemical structures of compounds **1**–**28**.

Aconicorine A (**1**) was purified as needle-like crystals. The molecular formula was determined as C_35_H_45_NO_15_S based on HR-ESI-MS at *m*/*z* 752.2586 [M + H]^+^ (calc. 752.2583). In ^1^H and ^13^C NMR spectra of **1**, characteristic signals were observed for a glucose moiety [δ_H_ 4.87 (d, *J* = 7.9 Hz, H-1″), 4.08 (t, *J =* 8.8 Hz, H-2″), 4.21 (t, *J =* 9.0 Hz, H-3″), 4.39 (t, *J =* 9.2 Hz, H-4″), 3.90 (m, H-5″), 4.47 (dd, *J =* 4.3 Hz, 11.9, H-6″a), and 4.50 (dd, *J =* 2.0 Hz, 11.9, H-6″b); δ_C_ 107.7, 75.9, 78.1, 70.6, 79.1, and 61.9] and a vanillyl group [δ_H_ 8.14 (d, *J =* 1.9 Hz, H-3′), 7.66 (d, *J =* 8.3 Hz, H-6′), 8.76 (m, H-7′), and 3.98 (s, H-8′); δ_C_ 166.6, 121.6, 113.8, 148.4, 153.4, 116.9, 127.1, and 56.3]. Its ^1^H NMR data (Table 1) also showed resonances for the exocyclic double bond at δ_H_ 4.67 and 4.84 (s, H-17), three oxygenated methines at δ_H_ 5.54 (m, H-1), 4.36 (s, H-7), and 5.23 (m, H-13), one nitrogen-containing methyl at δ_H_ 3.21 (s, H-21), a *N*-methylene at δ_H_ 3.36 and 3.98 (d, *J* = 11.7 Hz, H-19), a *N*-methine at δ_H_ 5.15 (s, H-20), and one methyl singlet at δ_H_ 1.50 (s, H-18). Besides 14 carbon signals for the glucose and vanillyl groups, 21 carbon signals were observed in the ^13^C NMR spectrum, which were classed into five quaternary carbons (including an olefinic and an oxygenated carbon), eight methines (including a nitrogenated and three oxygenated carbons), six methylenes (including an olefinic and a nitrogenated carbon), and two methyl carbons by the DEPT and HSQC data analysis. These NMR data indicated that **1** is a C-20 hetisine-type diterpenoid alkaloid glucoside, which was further supported by typical HMBC correlations (Figure 2) from H-20 (δ_H_ 5.15) to C-6 (δ_C_ 104.2) and C-19 (δ_C_ 71.5).^41^ Furthermore, in the HMBC spectrum, the correlation between H-2 (δ_H_ 5.54) and C-1′ (δ_C_ 166.6) indicated that the vanillyl group was attached to C-2, while the correlation between H-7 (δ_H_ 4.36) and C-1″ (δ_C_ 107.7) suggested that the glucose moiety was located at C-7. The presence of nitrogenated methyl was supported by HMBC correlations from *N*-CH_3_ (δ_H_ 3.21) to C-6, C-19, and C-20 (δ_C_ 75.4). Based on the molecular formula provided by HR-ESI-MS, compound **1** also contained a sulfonate group and a hydroxy group. In the DMSO-*d*_6_ NMR data (Table 1) of **1**, the HMBC correlations from the hydroxy proton (δ_H_ 8.43, H-6-OH) to C-5, C-6, and C-7 confirmed that the hydroxy group was located at C-6, and the sulfonate group was then assigned at C-13 (δ_C_ 73.6). Compound **1** contained both a quaternary ammonium group and the sulfonate, which may allow it to exist as an inner salt.

**Table 1:** ^1^H NMR (600 MHz) and ^13^C NMR (150 MHz) Data of **1** (δ in ppm, *J* in Hz)

	**1** (pyridine-*d*_5_)		**1** (DMSO-*d*_6_)	
No	δ_C_	δ_H_	δ_C_	δ_H_
1	29.4	1.43, dd (5.2, 15.3)	28.5	1.72, m
		2.82, m		2.45, d (15.5)
2	68.2	5.54, m	67.7	5.34, m
3	38.0	1.63, dd (4.2, 15.9)	37.0	1.72, m
		2.28, d (15.9)		1.99, d (15.7)
4	36.1		35.3	
5	56.5	2.12, s	55.0	2.17, s
6	104.2		102.9	
7	86.9	4.36, s	84.9	4.28, s
8	46.8		45.8	
9	48.5	1.96, d (10.1)	46.8	2.03, d (10.4)
10	45.1		44.3	
11	22.3	1.51, m	21.2	1.46, m
		2.29, m		2.09, m
12	40.4	2.82, m	39.2	2.88, m
13	73.6	5.23, m	73.1	4.44, m
14	43.3	3.20, s	41.7	2.88, s
15	29.1	2.31, d (18.2)	28.3	2.23, d (18.6)
		3.16, d (18.2)		2.90, m
16	146.7		146.5	
17	108.1	4.67, brs	107.7	4.74, d (1.7)
		4.84, brs		4.88, d (1.7)
18	29.8	1.50, s	29.0	1.40, s
19	71.5	3.36, d (11.7)	70.7	3.35, d (12.2)
		3.98, d (11.7)		3.57, d (12.2)
20	75.4	5.15, s	73.9	4.39, s
N-CH_3_	40.0	3.21, s	48.4	3.17, s
2-OVan-1′	121.6		120.4	
2′	113.8	8.14, d (1.9)	112.6	7.46, d (1.9)
3′	148.4		147.0	
4′	153.4		151.2	
5′	116.9	7.66, d (8.3)	115.1	6.83, d (8.3)
6′	127.1	8.76, m	124.8	7.75, dd (2.0, 8.3)
7′	166.6		165.2	
3′-OCH_3_	56.3	3.98, s	55.4	3.85, s
7-OGlu-1″	107.7	4.87, d (7.9)	105.7	4.40, d (7.9)
2″	75.9	4.08, t (8.8)	74.3	3.19, m
3″	78.1	4.21, t (9.0)	76.1	3.24, t (8.8)
4″	70.6	4.39, t (9.2)	69.6	3.14, t (9.2)
5″	79.1	3.90, m	77.1	3.20, m
6″	61.9	4.47, dd (4.3, 11.9)	60.7	3.53, dd (5.8, 12.0)
		4.50, dd (2.0, 11.9)		3.71, dd (2.2, 11.6)
6-OH				8.43, s

**Figure 2:**
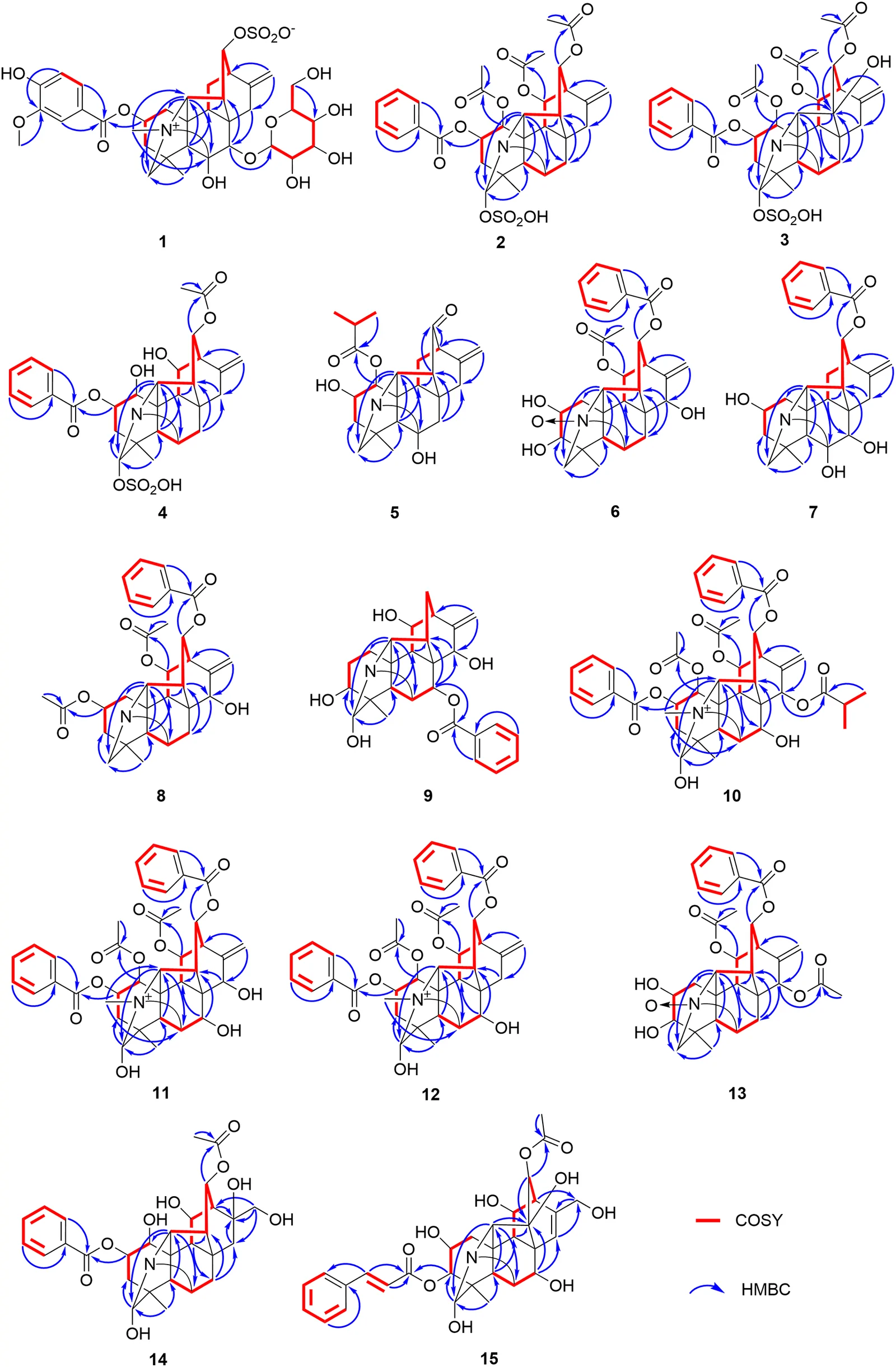
^1^H-^1^H COSY and HMBC correlations of compounds **1**–**15**.

The orthocoupling constants of H-1″ (*J* = 7.9 Hz) indicated that the glucose moiety was β-glucose. After glucose was released by 1N HCl acid hydrolysis, HPLC analysis of the chiral derivative confirmed that the glucose is d-glucose. Moreover, the relative configuration of **1** was also determined by NOESY spectra. The correlations (Figure 3) of H-2/H-18 and H-7/H-9 confirmed that both H-2 and H-7 were β-configurations. The H-13 signal overlapped with the water peak, making it difficult to determine the relative configuration of H-13. Ultimately, a crystal of **1** was successfully cultured, and single-crystal X-ray diffraction revealed that C-13 was *S*-configuration and compound **1** is an inner salt. Furthermore, the absolute configuration of **1** was determined by single-crystal X-ray diffraction with a Flack parameter of 0.019(10) (CCDC: 2553859, Figure 4).

**Figure 3:**
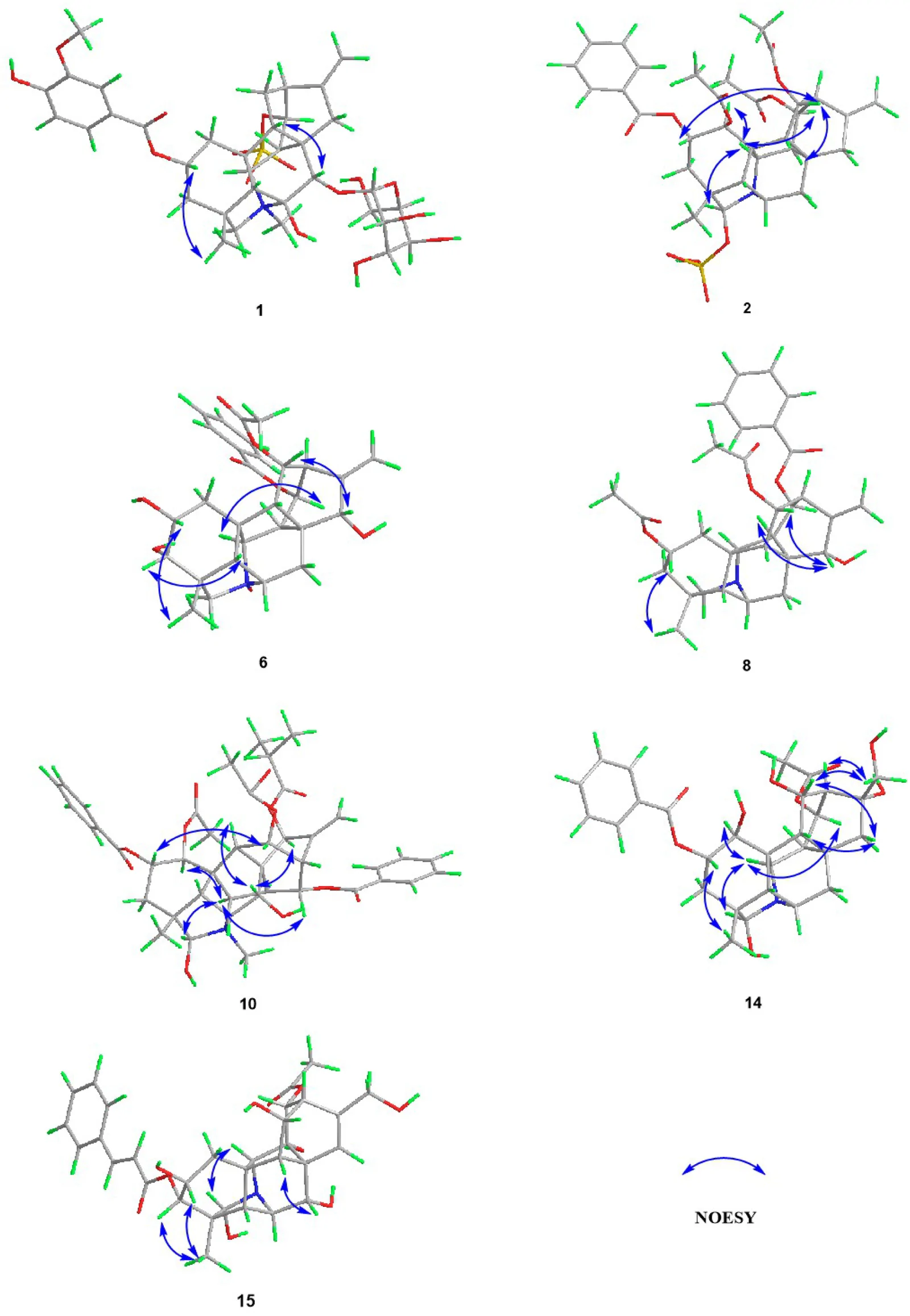
Key NOESY correlations of compounds **1**, **2**, **6**, **8**, **10**, **14**, and **15**.

**Figure 4:**
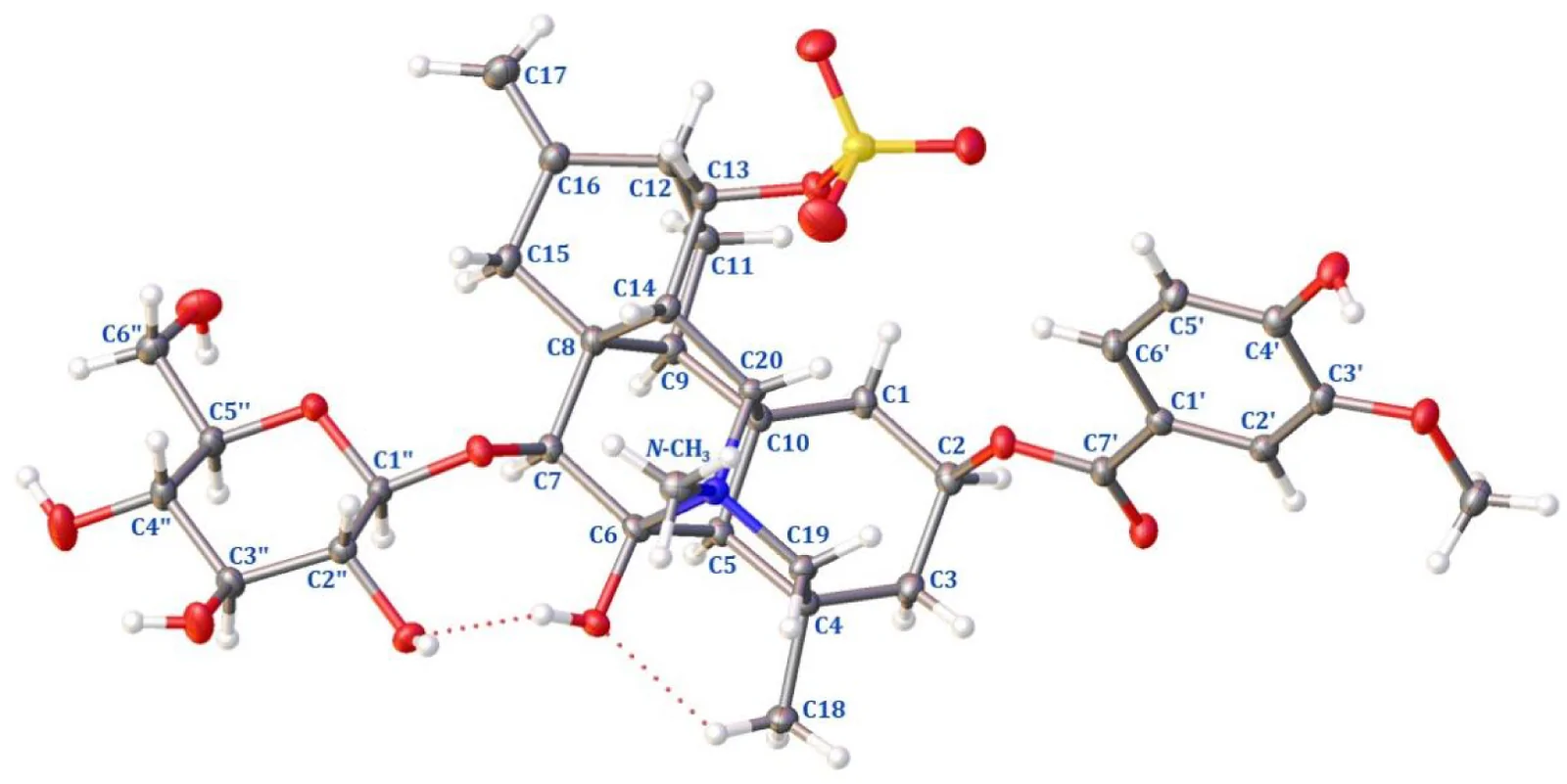
ORTEP representation of **1**.

Aconicorine B (**2**) was purified as a white, amorphous powder. The molecular formula was determined as C_33_H_37_NO_12_S based on HR-ESI-MS at *m*/*z* 672.2114 [M + H]^+^ (calc. 672.2109). Comparing guanfu base A (**19**),^42^ the NMR data of **2** (Tables 2 and 4) indicated that they have the same C-20 hetisine-type diterpenoid alkaloid skeleton, apart from differences in the type, number, and substitution position of substituents. In ^1^H and ^13^C NMR spectra of **2**, it was observed the characteristic signals for a benzoyloxy group [δ_H_ 8.13 (d, J = 7.3 Hz, H-2′/6′), 7.54 (t, J = 7.7 Hz, H-3′/5′), and 7.66 (t, J = 7.4 Hz, H-4′); δ_C_ 130.7, 131.2, 130.2, 135.0, and 166.4] and three acetyloxy groups [δ_H_ 2.09 (s, H_3_-1-OAc), 1.95 (s, H_3_-11-OAc), and 1.53 (s, H_3_-13-OAc); δ_C_ 171.4, 21.0, 172.0, 21.4, 170.9, and 20.7]. The HMBC correlations (Figure 2) between H-1 (δ_H_ 5.84) and carbonyl carbon of 1-OAc (δ_C_ 171.4), H-2 (δ_H_ 5.84), and C-7′ (δ_C_ 166.4), H-11 (δ_H_ 5.36) and carbonyl carbon of 11-OAc (δ_C_ 172.0), and between H-13 (δ_H_ 5.02) and carbonyl carbon of 13-OAc (δ_C_ 170.9) indicated that the benzoyloxy group was substituted at C-2, and the three acetyloxy groups were located at C-1, C-11, and C-13, respectively. The molecular formula provided by HR-ESI-MS revealed that **2** contained a sulfonate group, which was assigned to C-19 according to the chemical shifts of H-19 (δ_H_ 6.04, s) and C-19 (δ_C_ 94.2). The relative configuration of **2** was determined by NOESY spectra. NOESY correlations (Figure 3) between H-1/H-13/H-19/H-20 indicated that H-1, H-13, and H-19 were all α-oriented. The β-configurations of H-2 and H-11 were supported by the NOESY correlations of H-11/H-14 and H-2/H-11. Therefore, the structure of **2** was elucidated as shown in Figure 1.

**Table 2:** ^1^H NMR (600 MHz) Data of **2**–**5** (δ in ppm, *J* in Hz)

No	**2** ^a^	**3** ^a^	**4** ^b^	**5** ^a^
1	5.84, m	5.81, d (2.5)	4.80, m	4.69, d (2.6)
2	5.84, m	5.85, m	5.33, m	3.85, m
3	2.21, m	2.12, m	2.02, m	1.72, dd (4.4, 15.4)
	2.18, m	2.20, m	1.95, m	1.86, m
5	2.59, s	2.63, s	2.36, s	2.26, s
6	4.36, s	4.44, s	4.20, s	
7	2.07, m	1.90, d (14.7)	1.89, m	2.19, d (14.2)
	2.06, m	2.31, dd (2.9, 14.8)		2.41, d (14.2)
9	2.70, d (9.5)	2.78, d (9.2)	2.40, d (8.4)	2.34, d (9.7)
11	5.36, d (9.5)	5.33, d (9.2)	4.19, m	1.60, dd (4.4, 14.7)
				1.84, m
12	2.57, d (2.5)	2.67, d (2.4)	2.45, d (2.1)	2.95, d (3.8)
13	5.03, dt (2.1, 10.0)	4.88, m	4.85, m	
14	2.74, d (10.0)		2.71, d (10.0)	2.62, d (2.0)
15	2.52, brd (18.1)	2.26, brd (18.2)	2.32, m	2.46, brd (17.5)
	2.23, brd (18.1)	2.37, brd (18.2)	2.02, m	2.72, brd (17.5)
17	4.98, s	4.91, s	4.86, s	4.92, s
	4.89, s	5.01, s	4.74, s	5.03, s
18	1.25, s	1.28, s	1.11, s	1.53, s
19	6.04, s	6.04, s	5.92, m	3.45, d (11.3)
				3.87, d (11.3)
20	4.58, s	4.55, s	3.90, s	4.37, s
	1-OAc	1-OAc		1-OiBu
	2.09, s	2.10, s		2.58, m
	11-OAc	11-OAc		1.14, d (7.0)
	1.95, s	1.95, s		1.16, d (7.0)
	13-OAc	13-OAc	13-OAc	
	1.53, s	1.59, s	1.38, s	
	2-OBz	2-OBz	2-OBz	
2′/6′	8.13, d (7.3)	8.12, d (7.9)	8.09, d (7.7)	
3′/5′	7.54, t (7.7)	7.56, t (7.7)	7.51, t (7.7)	
4′	7.66, t (7.4)	7.68, t (7.3)	7.69, t (7.5)	
1-OH			5.02, d (5.0)	
11-OH			5.63, d (3.0)	

a Methanol-*d*_4_.

b DMSO-*d*_6_.

Aconicorine C (**3**) and D (**4**) were both purified to obtain white amorphous powders. The molecular formulas of **3** and **4** were determined as C_33_H_37_NO_13_S and C_29_H_33_NO_10_S based on HR-ESI-MS at *m*/*z* 688.2055 [M + H]^+^ (calc. 688.2058) and 588.1901 [M + H]^+^ (calc. 588.1898), respectively. The ^1^H and ^13^C NMR data of **3** and **4** (Tables 2 and 4) were comparable to those of **2.** Combined with their HR-MS data, **3** and **4** were suggested to be abnormal C20-hetisine diterpenoid alkaloids containing a sulfonate group. Comparing with **2**, an oxygenated quaternary carbon (δ_C_ 79.4, C-14) in **3** instead of the methine at C-14 (δ_C_ 49.1, δ_H_ 2.74) of **2** was observed, which suggested that a hydroxy group was attached to C-14 in **3**. For aconicorine D (**4**), resonances of two acetyloxy groups at C-1 and C-11 were absent in its NMR spectra compared to those of **2**, indicating that **4** is a de-1,11-diacetyloxy derivative of **2**. The ^1^H-^1^H COSY, HMBC, and NOESY data analysis (Figures 2 and S151) fully confirmed the structures of **3** and **4** as shown in Figure 1.

Compound **5** was purified as a white, amorphous powder. The molecular formula was determined as C_24_H_31_NO_5_ based on HR-ESI-MS at *m*/*z* 414.2281 [M + H]^+^ (calc. 414.2275). The NMR data of **5** (Tables 2 and 4) were found to be similar to those of guanfu base Q (**21**),^36^ except for the substituent at the C-1. In ^1^H and ^13^C NMR spectra of **5**, the characteristic signals of an isobutyryloxy group were observed [δ_H_ 2.58 (m, H-OiBu), 1.14 (d, *J* = 7.0 Hz, H_3_-OiBu), and 1.16 (d, *J* = 7.0 Hz, H_3_-OiBu); δ_C_ 177.0, 35.1, 19.3, and 19.0]. The HMBC correlation (Figure 2) between H-1 (δ_H_ 4.69) and carbonyl carbon of 1-OiBu (δ_C_ 177.0) indicated that the isobutyryloxy group was connected at C-1. The planar structure of **5** was deduced by fully ^1^H-^1^H COSY and HMBC data analysis. The α-configuration of H-1 was determined by the NOESY correlation (Figure S151) between H-20 and H-1. The small coupling constant of H-1 (*J* = 2.6 Hz) confirmed that H-2 is in the β-configuration, which was also consistent with guanfu base Q. Thus, the structure of **5** was established as shown (Figure 1) and named aconicorine E.

Aconicorine F (**6**) and M (**13**) have the molecular formulas C_29_H_33_NO_8_ and C_31_H_35_NO_9_ established by their HR-ESI-MS ions at *m*/*z* 524.2282 [M + H]^+^ (calc. 524.2279) and 566.2387 [M + H]^+^ (calc. 566.2385), respectively. The ^1^H and ^13^C NMR data of **6** and **13** (Tables 3 and 7) were similar to those of C-20 hetisine-type diterpenoid alkaloids. Five oxygenated positions at C-2, C-3, C-11, C-13, and C-15 in the hetisine skeleton were revealed in **6** and **13**. A benzoyloxy group and an acetyloxy group in **6** were assigned at C-13 and C-11, respectively, based on the observation of HMBC correlations (Figure 2) between H-11 (δ_H_ 5.49) and carbonyl carbon of 11-OAc (δ_C_ 171.7) and between H-13 (δ_H_ 5.30) and C-7′ (δ_C_ 167.7). Additionally, downfield shifts of C-6 (δ_C_ 74.5), C-19 (δ_C_ 71.7), and C-20 (δ_C_ 78.3) in **6** suggested oxidation at the nitrogen atom, which is similar to the analogous guanfu base X.^21^ The low-field chemical shifts of H-2 (δ_H_ 4.05), H-3 (δ_H_ 3.39), and H-15 (δ_H_ 4.07) confirmed the substitution of hydroxy groups at C-2, C-3, and C-15. The relative configuration of **6** was elucidated by NOESY spectra. The NOESY correlations (Figure 3) of H-2 and H-18 (δ_H_ 1.39), H-3 and H-5 (δ_H_ 2.41) indicated that the hydroxy groups at C-2 and C-3 were oriented on the same side as the α-configuration. The NOESY correlations of H-13/H-20 (δ_H_ 5.02) suggested that H-13 was α-oriented. Additionally, the orthocoupling constants of H-9 and H-11 (*J* = 9.1 Hz) indicated that H-11 was β-oriented, while the NOESY correlation between H-11 and H-15 proved that they were both β-configurations. Thus, the structure of **6** was determined as 2α,3α,15α-trihydroxy-11α-acetyloxy-13β-benzoyloxy-hetisine *N*-oxide. The NMR data for **13** were remarkably similar to those for **6**, except that the hydroxy group at C-15 in **6** was changed into an acetyloxy group in **13**. This change was confirmed by the lower field shift of the H-15 (δ_H_ 5.54) and the HMBC correlation (Figure 2) between H-15 (δ_H_ 5.54) and the carbonyl carbon of 15-OAc (δ_C_ 172.3). Similarly, the structure of **13** was elucidated as 2α,3α-dihydroxy-11α,15α-diacetyloxy-13β-benzoyloxy-hetisine *N*-oxide.

**Table 3:** ^1^H NMR (600 MHz) Data of **6**, **13**–**15** (Methanol-*d*_4_, δ in ppm, *J* in Hz)

No	6	13	14	15
1	3.29, dd (3.9, 15.0)	3.29, dd (3.9, 14.9)	4.98, m	1.89, dd (3.7, 15.6)
	1.76, dd (3.9, 15.0)	1.78, dd (3.9, 14.9)		3.49, dd (2.7, 15.6)
2	4.05, q (3.7)	4.05, m	5.51, m	4.27, m
3	3.39, d (4.3)	3.39, m	2.02, d (16.0)	4.93, d (4.1)
			2.11, dd (4.5, 16.0)	
5	2.41, s	2.44, s	2.41, s	2.39, s
6	4.37, s	4.36, s	4.34, s	4.26, m
7	2.15, dd (3.3, 15.0)	2.19, dd (3.2, 14.9)	1.95, m	4.31, d (2.2)
	2.42, dd (3.3, 15.0)	2.16, dd (3.2, 14.9)	1.95, m	
9	2.68, dd (2.0, 9.1)	2.73, dd (2.1, 9.0)	2.38, d (8.9)	1.85, d (9.1)
11	5.49, d (9.0)	5.43, brd (9.0)	4.41, d (8.8)	3.89, d (8.9)
12	2.90, d (2.4)	2.94, d (2.4)	2.18, s	2.82, m
13	5.31, m	5.35, dt (2.1, 10.1)	5.78, d (8.5)	4.84, m
14	3.24, s	3.40, m	2.71, d (8.9)	
15	4.07, s	5.54, s	1.26, d (15.0)	5.93, s
			1.60, d (15.0)	
17	5.32, s	5.40, s	3.46, d (11.3)	4.18, d (1.4)
	5.31, s	5.38, s	3.63, d (11.3)	4.17, d (1.4)
18	1.39, s	1.39, s	1.17, s	1.22, s
19	5.01, m	5.02, m	5.51, m	6.31, s
	3.25, m	3.26, m		
20	5.02, s	5.05, s	4.24, s	4.65, s
		11-OAc		
		1.90, s		
	11-OAc	15-OAc	13-OAc	13-OAc
	1.88, s	2.13, s	1.54, s	2.16, s
	13-OBz	13-OBz	2-OBz	3-OCin
2“/6”	8.34, d (7.2)	8.34, d (7.2)	8.05, d (7.3)	7.62, dd (2.9, 6.5)
3‘/5′	7.52, t (7.9)	7.52, t (7.9)	7.57, t (7.0)	7.43, m
4’	7.64, t (7.3)	7.65, t (7.3)	7.70, t (6.6)	7.43, m
7’				7.78, d (16.0)
8’				6.59, d (16.0)

Aconicorines G–I (**7**–**9**) were obtained as a white, amorphous powder. Their molecular formulas were determined as C_27_H_31_NO_5_, C_31_H_35_NO_7_, and C_27_H_31_NO_6_ based on HR-ESI-MS positive ions at *m*/*z* 450.2277 [M + H]^+^ (calc. 450.2275), 534.2488 [M + H]^+^ (calc. 534.2486), and 466.2225 [M + H]^+^ (calc. 466.2224), respectively. Comprehensive ^1^H and ^13^C NMR data (Tables 4 and 5) analysis of **7**–**9** revealed that **7**–**9** are undescribed highly oxygenated C-20 hetisine-type diterpenoid alkaloids. The difference between them lies in the different oxidation sites and substituents on the hetisine skeleton. For compound **7**, oxidation sites were assigned at C-2 (δ_C_ 66.0), C-6 (δ_C_ 103.3), C-7 (δ_C_ 73.4), and C-13 (δ_C_ 74.9) based on the ^1^H-^1^H COSY correlations (Figure 2) of H-1/H-2/H-3 and H-12/H-13/H-14 as well as the HMBC correlation (Figure 2) between H-13 (δ_H_ 5.21) and C-7′ (δ_C_ 167.6) of the benzoyloxy group. The NOESY correlations (Figure S151) of H-2/H-18β, H-7/H-9β, and H-13/H-20α in the NOESY spectrum of **7** indicated that H-2 and H-7 are β-oriented and H-13 is α-oriented. Thus, the structure of **7** was elucidated as 2α,6β,7α-trihydroxy-13β-benzoyloxy-hetisine. Comparing the NMR data of **7**, two additional acetyloxy groups at C-2 and C-11 were observed in **8**, which was verified by the HMBC correlations (Figure 2) from H-2 (δ_H_ 5.23) to C-2-OAc (δ_C_ 170.9) and from H-11 (δ_H_ 5.59) to C-11-OAc (δ_C_ 171.7). The ^1^H-^1^H COSY correlations (Figure 2) of H-1/H-2/H-3 and H-9/H-11/H-12/H-13/H-14 as well as HMBC correlations of H-13/C-7′, H-17/C-15, and H-15/C-7/C-14 further confirmed the connections of a benzoyloxy group at C-13 and a hydroxy group at C-15. In the NOESY spectrum of **8**, correlations (Figure S151) of H-15/H-14 and H-11/H-15 indicated that H-11 and H-15 were both in the β-configuration. Furthermore, the configurations of C-2 and C-13 were the same as those of **7**. Similarly, five oxidation sites in **9** were established for four hydroxy groups at C-3, C-11, C-15, and C-19, and a benzoyloxy group at C-7 according to its 1D and 2D NMR data analysis (Figures 2 and S151). Therefore, the structures of **8** and **9** were elucidated as 2α,11α-diacetyloxy-13β-benzoyloxy-15α-hydroxy-hetisine (**8**) and 3α,11α,15α,19β-tetraacetyloxy-7α-benzoyloxy-hetisine (**9**).

**Table 4:** ^13^C NMR (150 MHz) Data of **2**–**5**, **7**–**9** (δ in ppm, *J* in Hz)

No	2^a^	3^a^	4^b^	5^a^	7^a^	8^a^	9^a^
1	70.5	70.2	64.5	70.3	32.2	30.9	27.9
2	68.7	68.5	72.4	67.1	66.0	69.5	28.5
3	33.4	33.3	32.1	38.0	43.0	36.2	72.9
4	43.3	43.5	41.6	37.3	37.7	36.7	49.6
5	55.8	54.6	53.1	56.1	56.7	58.1	58.1
6	64.8	64.0	63.3	103.2	103.3	67.3	66.3
7	34.0	29.9	32.5	40.9	73.4	32.0	69.3
8	45.1	46.1	43.2	44.1	49.0	49.7	53.3
9	52.1	50.7	51.9	47.4	48.1	49.9	49.5
10	55.0	50.4	54.8	52.5	48.2	51.5	51.7
11	75.7	75.2	72.0	23.6	23.2	75.0	73.5
12	46.1	47.1	46.8	53.3	40.8	46.0	44.1
13	73.6	82.3	72.5	207.6	74.9	74.4	28.2
14	49.1	79.4	46.7	59.2	44.5	48.0	39.2
15	33.5	30.7	32.2	32.7	30.1	70.5	65.9
16	143.0	142.0	144.2	142.4	147.3	148.5	153.1
17	111.6	111.8	109.3	112.5	109.2	115.8	112.2
18	22.1	21.8	21.8	30.4	30.7	29.0	19.1
19	94.2	93.9	92.1	58.4	58.8	61.5	90.7
20	65.4	66.7	63.6	65.8	67.9	69.6	68.7
	1-OAc	1-OAc		1-OiBu		2-OAc	
	171.4	171.3		177.0		170.9	
	21.0	21.0		35.1		20.6	
				19.0			
				19.3			
	11-OAc	11-OAc	11-OAc			11-OAc	
	172.0	171.8	169.4			171.7	
	21.4	21.4	20.3			21.4	
	13-OAc	13-OAc					
	170.9	170.2					
	20.7	20.6					
	2-OBz	2-OBz	2-OBz		13-OBz	13-OBz	7-OBz
1′	130.7	130.5	129.3		130.8	130.6	130.4
2′/6′	131.2	131.0	129.9		131.2	131.0	131.0
3′/5′	130.2	130.2	129.2		129.5	130.1	129.7
4′	135.0	135.0	134.1		134.5	135.2	134.9
7′	166.4	166.2	165.2		167.6	167.2	166.8

a Methanol-*d*_4_.

b DMSO-*d*_6_.

**Table 5:** ^1^H NMR (600 MHz) Data of **7**–**9** (Methanol-*d*_4_, δ in ppm, *J* in Hz)

No	7	8	9
1	2.44, brd (14.4)	3.11, brd (15.5)	2.89, brd (15.1)
	1.53, dd (4.3,14.4)	1.96, m	1.86, dd (4.0, 14.4)
2	4.18, m	5.23, m	1.93, m
			1.55, m
3	1.90, m	1.87, brd (15.8)	3.55, dd (5.6, 12.0)
	1.63, m	1.65, dd (4.5, 15.8)	
5	1.97, s	2.26, s	2.29, s
6		4.23, s	4.36, s
7	3.86, s	2.47, dd (1.9, 15.0)	5.63, d (2.2)
		1.94, dd (1.9, 11.6)	
9	2.00, m	2.74, d (2.1)	2.35, d (8.7)
11	2.33, dd (4.2, 14.9)	5.59, brd (9.1)	4.44, d (8.5)
	1.65, m		
12	2.51, m	2.75, d (2.2)	2.26, m
13	5.21, dt (2.2, 10.2)	5.34, d (9.7)	1.91, m
			1.71, brd (14.0)
14	2.94, dd (1.8, 10.1)	2.71, dd (1.6, 9.8)	2.65, d (10.7)
15	2.78, dt (2.4, 17.9)	4.04, s	4.21, s
	2.37, dt (2.4, 17.9)		
17	5.00, s	5.31, m	5.06, s
	4.86, s	5.31, m	5.03, s
18	1.50, s	1.17, s	1.24, s
19	3.90, d (11.1)	3.42, d (12.0)	5.07, s
	3.30, d (11.1)	3.02, d (12.0)	
20	4.41, s	3.97, s	3.70, s
		2-OAc	
		1.18, s	
		11-OAc	
		2.17, s	
	13-OBz	13-OBz	7-OBz
2′/6′	8.24, d (7.2)	8.13, d (7.2)	8.15, d (7.3)
3′/5′	7.47, t (8.1)	7.54, t (7.9)	7.53, t (7.7)
4′	7.61, t (7.7)	7.70, t (7.5)	7.66, t (7.5)

Aconicorine J (**10**) has a molecular formula C_43_H_48_NO_12_ determined by HR-ESI-MS ion at *m*/*z* 770.3178 [M]^+^ (calc. 770.3171). Like those of **2**, two acetyloxy groups at C-1 and C-11, and two benzoyloxy groups at C-3 and C-13 of hetisine skeleton in **10** were deduced. Besides the benzoyloxy groups and acetyloxy groups, an isobutyryloxy group and two hydroxy groups were assigned at C-15, C-7, and C-19, respectively, supported by HMBC correlations (Figure 2) from H-15 (δ_H_ 6.00) to carbonyl carbon of 15-OiBu (δ_C_ 177.6), from H-7 (δ_H_ 4.26) to C-6, C-9, and C-14, and from H-19 (δ_H_ 5.39) to C-6, C-18, and C-20. The HMBC correlations from *N*-CH_3_ (δ_H_ 3.10) to C-6, C-19, and C-20 confirmed the presence of the nitrogen-methyl group. NOESY correlations (Figure 3) between H-1/H-13/H-19/H-20 confirmed that H-1, H-13, and H-19 were all α-oriented. The NOESY correlation between H-2 and H-11 suggested that H-2 and H-11 had the same configuration, and the small coupling constant of H-1 (*J* = 2.1 Hz) inferred that H-2 is opposite to H-1. Furthermore, NOESY correlations of H-7/H-9 and H-7/H-15 indicated that H-7 and H-15 were β-oriented. The structure of **10** was deduced as 1β,11α-diacetyloxy-2α,13β-dibenzoyloxy-7α,19β-hydroxy-15α-isobutyryloxy-*N*-methyl-hetisine (**10**).

Aconicorines K (**11**) and L (**12**) have molecular formulas C_39_H_42_NO_11_ and C_39_H_42_NO_10_ based on HR-ESI-MS ions at *m*/*z* 700.2747 [M]^+^ (calc. 700.2752) and 684.2805 [M]^+^ (calc. 684.2803), respectively. The NMR data of **11** and **12** were extremely similar to those of **10**. As analogs of **10**, they differ only in C-15. The chemical shifts of H-15 in **11** and **12** were both upfield-shifted compared to those of **10**. The chemical shift of H-15 (δ_H_ 4.64) in **11** indicated that a hydroxy group was connected to C-15 in **11**, but not an isobutyryloxy group. The chemical shift for H-15 (δ_H_ 2.38, 2.88) of **12** suggested that C-15 was converted to a methylene in **12**. The HMBC correlations (Figure 2) from H-15 and C-9, C-14, and C-17 in **11** and **12** further supported this assignment. NOESY spectra of **11** and **12** confirmed that the relative configurations of **11** and **12** were the same as those of **10**. Thus, the structures of **11** and **12** were determined as shown (Figure 1).

Aconicorines N (**14**) and O (**15**) were obtained as white, amorphous powder with molecular formulas C_29_H_35_NO_9_ and C_31_H_35_NO_10_, respectively. In the ^1^H and ^13^C NMR spectra of **14** and **15** (Tables 3 and 7), no typical signal for the exocyclic double bond of hetisine was observed. Two hydroxy groups were assigned at C-16 and C-17 in **14** by their chemical shifts and HMBC correlations (Figure 2) from H-12 (δ_H_ 2.18) and H-15 (δ_H_ 1.26, 1.60) to C-16 (δ_C_ 73.4), and from H-17 (δ_H_ 3.46, 3.63) to C-12, C-15, and C-16. Like those of **4**, 1,11,19-trihydroxy-2-dibenzoyloxy-13-acetyloxy substituents at the hetisine skeleton in **14** were determined by the NMR spectroscopic analysis. The NOESY correlations (Figure 3) from H-20 to H-1, H-13, and H-19 confirmed that H-1, H-13, and H-19 of **14** were all α-oriented. The β-oriented H-2 was determined by the NOESY correlation between H-2 and H-18. The NOESY correlations of H-9/H-15β/H-11 and from H-17 to H-11 and H-12 proved that 11-OH and 16-OH were α-oriented. In ^1^H and ^13^C NMR spectra of **15**, the signals of the intracyclic double bond at C-15 and C-16 were observed, similar to those in the compound guanfu base W.^21^ A hydroxy group was connected to C-17 in **15** based on the chemical shifts of C-17 (δ_C_ 62.8) and HMBC correlations (Figure 2) from H-17 (δ_H_ 4.17) to C-12 (δ_C_ 45.6), C-15 (δ_C_ 121.1), and C-16 (δ_C_ 146.9). In addition, the characteristic signals for a cinnamic acid group [δ_H_ 7.62 (dd, *J* = 2.9, 6.5 Hz, H-2′/6′), 7.43 (m, H-3′/5′), 7.43 (m, H-4′), 7.78 (d, *J* = 16.0 Hz, H-7′), and 6.59 (d, *J* = 16.0 Hz, H-8′)] were also observed, and the substitution of the cinnamic acid group at C-3 was determined by HMBC correlation between H-3 (δ_H_ 4.93) and C-9′ (δ_C_ 167.7). Meanwhile, HMBC correlation between H-13 (δ_H_ 4.84) and carbonyl carbon of 13-OAc (δ_C_ 172.0) indicated that the acetyloxy group was located at C-13. The remaining hydroxy groups were assigned at C-2, C-7, C-11, C-14, and C-19 by ^1^H-^1^H COSY correlations (Figure 2) of H-1/H-2/H-3, H-5/H-6/H-7, and H-9/H-11/H-12/H-13 as well as HMBC correlations from H-7, H-9, H-12, and H-20 to C-14, and from H-19 to C-6 and C-20. NOESY correlations (Figure 3) of H-2/H-3/H-18 confirmed that H-2 and H-3 of **15** were both β-oriented. The α-configured hydroxy group at C-7 was supported by the NOESY correlation of H-7/H-9. The NOESY correlation of H-19/H-20 indicated that the hydroxy group at C-19 was β-oriented. The coupling constant of H-9 (*J* = 9.1 Hz) inferred that H-11 is in the same side as H-9. Finally, the structures of **14** and **15** were established as shown (Figure 1).

Based on preliminary experiments, the effects of isolated diterpenoid alkaloids (**1**–**25**) on PD-L1 expression were screened at a noncytotoxic concentration (Figures S151–S152) in human breast cancer cells (MDA-MB-231 cells). Western blot analysis showed that compounds **1**, **7**, **13**–**17**, and **20** inhibited PD-L1 expression at a concentration of 10 μM (Figure 5). Subsequently, the effects of different concentrations of **7** (2.5, 5, and 10 μM), **1**, **13**–**17**, and **20** (5, 10, and 20 μM) were evaluated. Results demonstrated that **7**, **14**, **15**, and **17** significantly inhibited PD-L1 expression in a dose-dependent manner, while other tested compounds (**1**, **13**, **16**, and **20**) exhibited varying degrees of inhibitory effects (Figure 6A and B). Additionally, the PD-L1 inhibitory effects of **7**, **14**, **15**, and **17** were verified by immunofluorescence staining (Figure 6C). Compared with the blank control group, the bright spots of PD-L1 in the field of view were reduced after the treatment with 10 μM of **7**, **14**, **15**, and **17**, respectively, in MDA-MB-231 cells (Figure 6C). According to the above-mentioned first discovery of the effect of diterpenoid alkaloids on PD-L1, it was found that the type of oxidizing group and the substitution position on the hetisine skeleton have different effects on PD-L1. Although compound **1** showed potential PD-L1 downregulation activity, other sulfonate-containing diterpenoid alkaloids (**2**–**4**) did not exhibit significant activity. Furthermore, compared to **8** and **9**, compound **7** exhibited better PD-L1 inhibition due to simultaneous oxidative substitution at C-6 and C-7. Compounds **16** and **17** exhibited better PD-L1 inhibition compared to **18** and **19**, suggesting that benzoylation at C-13 could be important for this activity. To refine more effective structure–activity relationships, more bioassay of hetisine-type alkaloids will be investigated in future.

**Figure 5:**
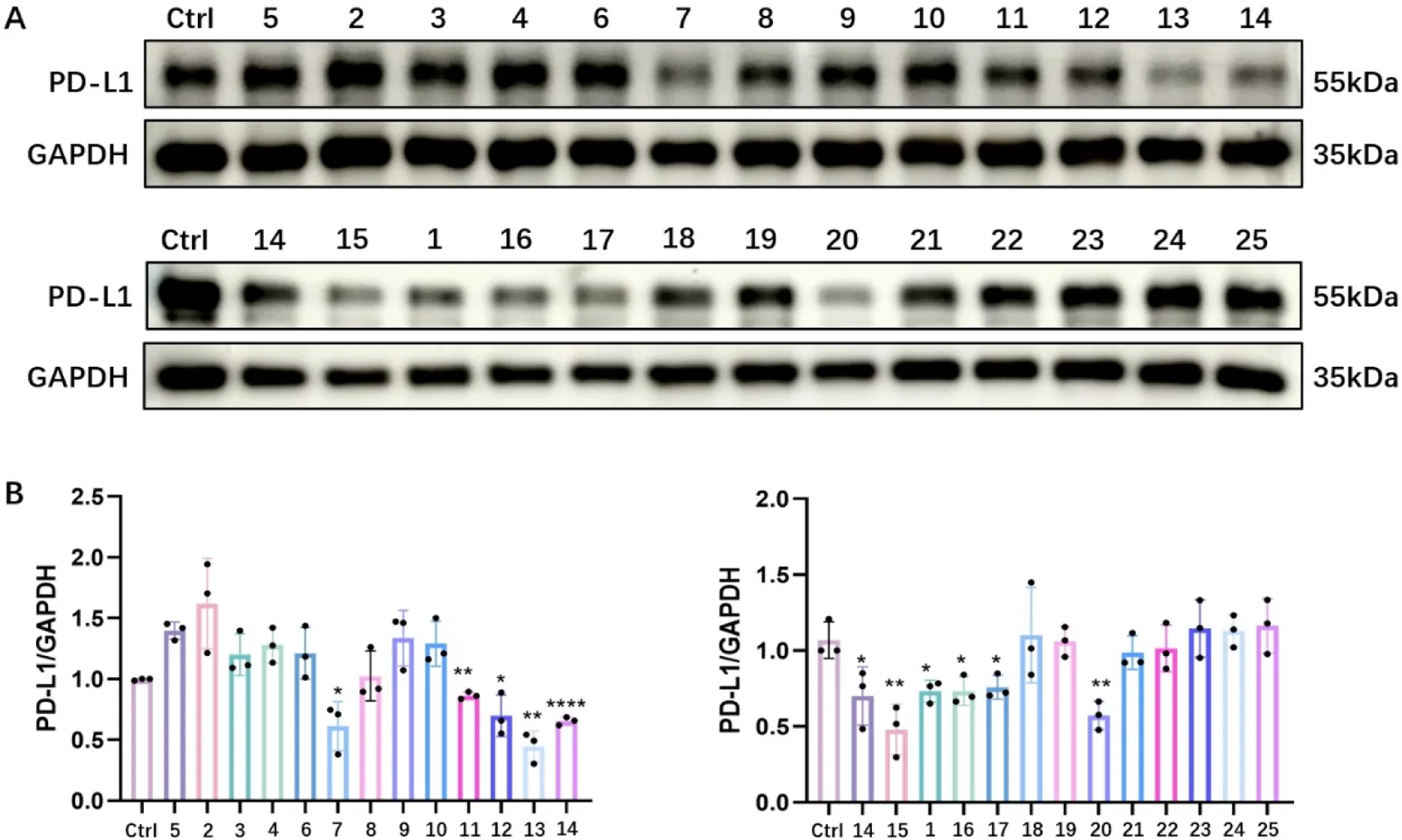
Effects of compounds **1**–**25** on PD-L1 expression in MDA-MB-231 cells. (A) The effects of compounds **1**–**25** on PD-L1 expression at a concentration of 10 μM, with a blank group as a control. (B) Relative quantification of PD-L1 in A. Data shown are mean ± SEM *p < 0.1, **p < 0.01, ***p < 0.001, compared with the control group.

**Figure 6:**
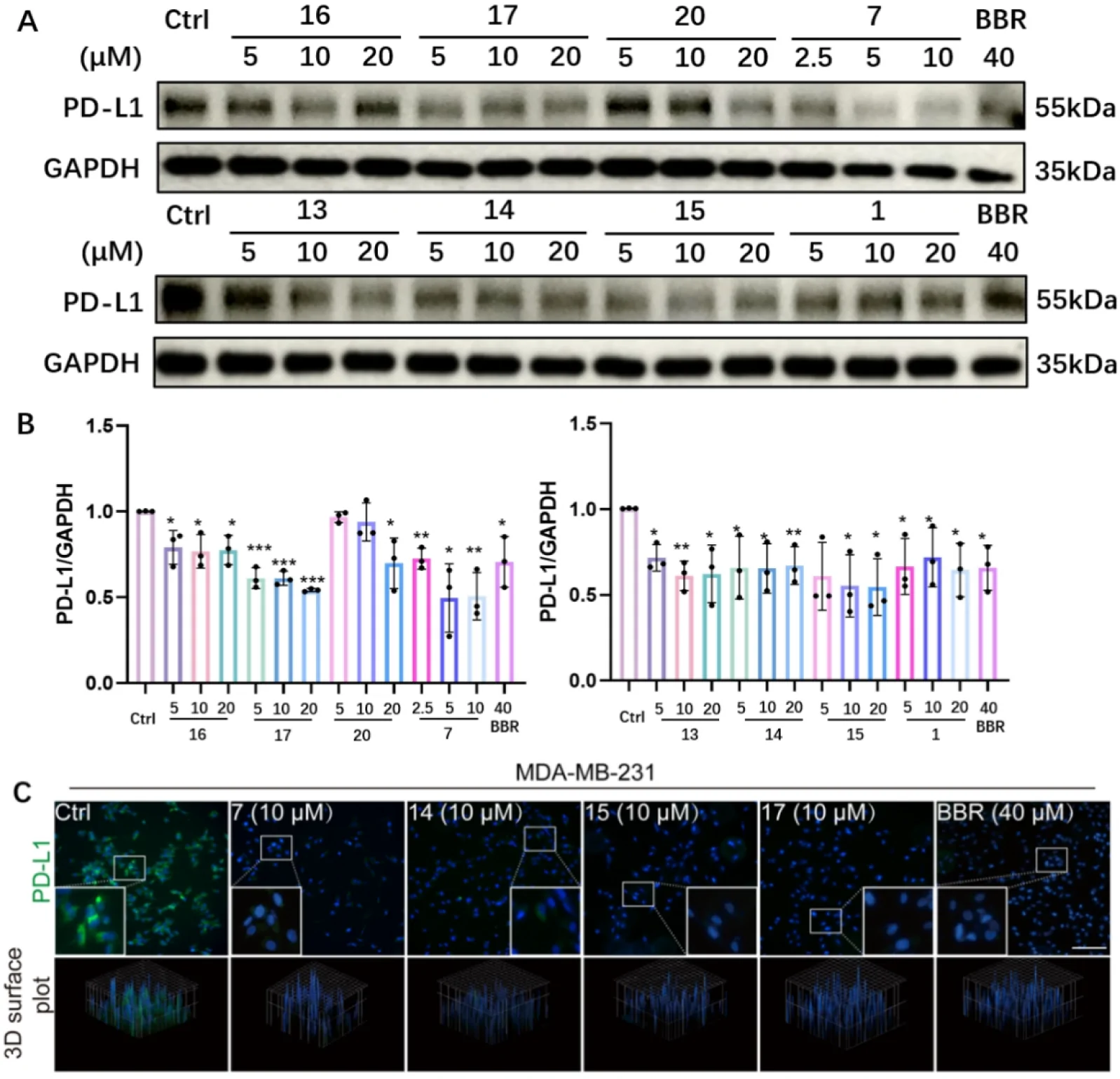
Effects of compounds **1**, **7**, **13**–**17**, and **20** on PD-L1 expression in MDA-MB-231 cells. (A) The effects of compounds **1**, **7**, **13**–**17**, and **20** on PD-L1 protein expression at concentration gradients of 2.5–20 μM, with a blank group and a 40 μM BBR group as controls. (B) Relative quantification of PD-L1 in A. Data shown are mean ± SEM *p < 0.1, **p < 0.01, ***p < 0.001, compared with the control group. (C) PD-L1 protein expression (green) and cell nuclei (blue) after drug administration were observed using an Agilent BioTek Cytation 5 cell imaging system.

In summary, phytochemical studies of *A. coreanum* identified 15 new C20 hetisine-type diterpenoid alkaloids (**1**–**15**) and 13 known diterpenoid alkaloids (**16**–**28**). All new compounds are highly oxygenated hetisine-type diterpenoid alkaloids with diverse oxidation sites and substituents at C-1–3, C-6, C-7, C-11, C-13–15, or C-19 of hetisine skeleton. Compounds **1**–**4** are abnormal C20 diterpenoid alkaloids possessing a rare sulfonate group. Eight alkaloids (**1**, **7**, **13**–**17**, and **20**) exhibited PD-L1 inhibitory activity at concentrations ranging from 2.5 to 20 μM in MDA-MB-231 cells. This study first reported the effects of C20 diterpenoid alkaloids on PD-L1 expression and suggested that C20 diterpenoid alkaloids could be a new type of PD-L1 modulator.

## Experimental Section

### General Experimental Procedures

Optical rotation was measured at room temperature using an Autopol I automatic polarimeter. UV and circular dichroism spectra were measured using a JASCO J-1500 circular dichroism spectrophotometer. HRESIMS spectra were measured using an Agilent 6230 TOF mass spectrometer. Medium-pressure liquid chromatography (MPLC) was performed using a Buchi C-620 system (Buchi, Switzerland) equipped with a C-635 UV detector and a SiliaBond C18 column (ODSGel, 40–63 μm, 26 × 460 mm). Semipreparative HPLC was performed on an Agilent Technologies 1200 series and ThermoFisher Ultimate 3000 system, using a variety of chromatographic columns, including an Atlantis T3 OBD column (5 μm, 10 × 250 mm, Waters), a SunFire C18 OBD column (5 μm, 10 × 250 mm, Waters), a ChromCore Phenyl-Ether column (5 μm, 10 × 250 mm, NanoChrom), and a Symmetry C18 column (5 μm, 4.6 × 250 mm, Waters). The flow rate was 3 mL/min, and UV detection was performed at 210 and 254 nm. ^1^H, ^13^C, DEPT, HSQC, HMBC, ^1^H-^1^H COSY, and NOESY NMR spectra were acquired on a Bruker Avance 600 MHz spectrometer (Bruker, Germany) using TMS as the internal standard. Thin-layer chromatography was performed using Precoated silica gel GF254 plates (Merck, Darmstadt, Germany), and the diterpenoid alkaloid-containing fractions were visualized with potassium bismuth iodide. Automated CO_2_ Incubator at 37 °C and 5% CO_2_, Thermo Scientific Heraeus X1R Refrigerated Centrifuge, Olympus CKX53 IPC Inverted Microscope, Tecan Infinite M200 PRO, and Agilent BioTek Cytation 5 were used.

### Plant Material

The root samples of *A. coreanum* were harvested in May 2022 from a cultivated plantation located in Qingyuan County, Fushun City, Liaoning Province, China. The plant was identified by Dr. Zhu Guo-Yuan of Macau University of Science and Technology based on its morphological characteristics in comparison with the standard descriptions in the Flora of China. The voucher sample (AC-2022–05) was stored at the State Key Laboratory for Quality Research in Chinese Medicine, Macau University of Science and Technology.

### Extraction and Isolation

The dried roots of *A. coreanum* (10 kg) were powdered and percolated by 80% ethanol at room temperature. After combination, the ethanol solution was concentrated under reduced pressure. The obtained crude extract (307.5 g) was dispersed in water and sequentially partitioned using PE, EtOAc, and *n*-BuOH. The EtOAc part (33.5 g) was fractionated via Sephadex LH-20 column chromatography (eluted by MeOH), yielding 8 fractions (Fr.E-1–8). The PE part (106.3 g) was loaded onto a silica gel column, employing a stepwise gradient elution of PE-EtOAc-MeOH (v/v/v 20:1:0–0:1:0–0:0:1) to give 11 fractions (Fr.P-1–11). These fractions were monitored by LC-MS and TLC. Fractions (P-9–10, 45.2 g and E-3–7, 21.7 g) containing alkaloids were then combined and further purified on a silica gel column using a gradient mixture of CHCl_2_-MeOH (v/v 100:1–1:1) to give 9 fractions (Fr.EP-1–9).

By employing a MeCN-H_2_O-AcOH gradient elution (5:95:0.1 to 100:0:0.1, v/v/v) in MPLC, 11 subfractions (EP-1–1 to EP-1–11) were successfully obtained from Fr.EP-1 (6.50 g). Fr.EP-1–4 (211.7 mg) was separated by semipreparative HPLC with MeCN-H_2_O-AcOH (30:70:0.1) to give **21** (32.1 mg, *t*_R_ = 5.9 min). Fr.EP-1–7 (251.5 mg) underwent semipreparative HPLC separation eluted with MeCN-H_2_O-AcOH (40:60:0.1) to give Fr.EP-1–7–2 (21.0 mg) and Fr.EP-1–7–8 (24.1 mg). Subsequently, compound **5** (8.6 mg, *t*_R_ = 11.9 min) was obtained from Fr.EP-1–7–2 via semipreparative HPLC purification (MeCN-H_2_O-AcOH, 28:72:0.1). Meanwhile, processing Fr.EP-1–7–8 through semipreparative HPLC eluted with MeCN-H_2_O-AcOH (36:64:0.1) to yield **2** (1.9 mg, *t*_R_ = 22.5 min) and **3** (2.0 mg, *t*_R_ = 23.5 min). After LC-MS analysis, Fr.EP-1–8–10 (0.73 g) were combined and separated by isocratic MHPLC with 35% MeCN for 5 h to obtain 11 fractions (Fr.EP-1–10–1–11). Fr.EP-1–10–6 and Fr.EP-1–10–7 were subjected to semipreparative HPLC eluted with MeCN-H_2_O-AcOH (33:67:0.1) to yield **10** (14.8 mg, *t*_R_ = 13.3 min). Mass spectrometry analysis showed that fractions EP-2 and EP-3 contained essentially the same components. The separation of combined Fr.EP-2 (2.56 g) and Fr.EP-3 (3.15 g) was performed on MPLC with a gradient elution of MeCN-H_2_O-TFA (v/v/v 5:95:0.05–100:0:0.05) to provide 9 subfractions (Fr.EP-2–1–9). Fr.EP-2–4 (759.1 mg) was separated and purified by semipreparative HPLC using MeCN-H_2_O-TFA (22:78:0.05) to yield **18** (17.6 mg, *t*_R_ = 25.5 min), **22** (30.6 mg, *t*_R_ = 13.3 min), and **26** (39.2 mg, *t*_R_ = 11.3 min). Fr.EP-2–5 (492.8 mg) was separated and purified by semipreparative HPLC using MeCN-H_2_O-TFA (30:70:0.05) to yield **4** (3.3 mg, *t*_R_ = 18.8 min), **7** (26.8 mg, *t*_R_ = 52.0 min), **17** (13.8 mg, *t*_R_ = 29.8 min), and **27** (26.3 mg, *t*_R_ = 15.3 min). Fr.EP-5 (6.64 g) was subsequently subjected to MPLC separation, utilizing a gradient elution of MeCN-H_2_O-AcOH (v/v/v 5:95:0.1–100:0:0.1) to provide 8 subfractions (Fr.EP-5–1–8). Fr.EP-5–4 (167.2 mg) was isolated via semipreparative HPLC, eluting with MeCN-H_2_O-TFA (20:80:0.05) to yield **20** (20.0 mg) and **22** (75.5 mg). Fr.EP-5–7 (105.7 mg) was subjected to semipreparative HPLC eluted with MeCN-H_2_O-TFA (40:60:0.05) to yield **8** (1.7 mg, *t*_R_ = 26.9 min) and **28** (3.6 mg, *t*_R_ = 14.3 min). Fr.EP-5–8 (486.1 mg) was subjected to semipreparative HPLC eluted with MeCN-H_2_O-TFA (50:50:0.05) to yield **11** (54.9 mg, *t*_R_ = 9.2 min) and **12** (39.7 mg, *t*_R_ = 12.8 min). Fr.EP-7 (2.05 g) was chromatographed by MPLC, employing a gradient elution of MeCN-H_2_O-TFA (v/v/v 5:95:0.1–100:0:0.05) to provide 12 subfractions (Fr.EP-7–1–12). Compound **16** (13.2 mg, *t*_R_ = 16.6 min) was isolated and purified from Fr.EP-7–8 (88.6 mg) by semipreparative HPLC, MeCN-H_2_O-TFA (23:77:0.05). Fr.EP-7–9 (84.0 mg) was separated and purified by semipreparative HPLC using MeCN-H_2_O-TFA (35:65:0.05) to yield **6** (3.3 mg, *t*_R_ = 6.3 min) and **13** (9.9 mg, *t*_R_ = 9.7 min). Fr.EP-8 (6.32 g) was chromatographed by MPLC with a gradient elution of MeCN-H_2_O-TFA (v/v/v 5:95:0.1–100:0:0.05) to provide 11 subfractions (Fr.EP-8–1–11). Compound **23** (67.8 mg, *t*_R_ = 16.1 min) was isolated and purified from Fr.EP-8–5 (745.9 mg) by semipreparative HPLC, MeCN-H_2_O-TFA (13:87:0.05). Fr.EP-8–7 (393.6 mg) was separated and purified by semipreparative HPLC using MeCN-H_2_O-TFA (15:85:0.05) to yield **9** (31.0 mg, *t*_R_ = 23.9 min).

The *n*-BuOH part (167.7 g) was fractionated by a silica gel CC using a stepwise elution of CHCl_2_-MeOH (v/v 100:1–1:1) to give 14 fractions (Fr.Z-1–14). Fr.Z-5 was recrystallized to obtain **19** (429.5 mg). Fr.Z-6 (3.86 g), Fr.Z-7 (3.49 g), Fr.Z-9 (5.83 g), and Fr.Z-10 (5.63 g) were individually subjected to Sephadex LH-20 chromatography, eluted with pure MeOH, to furnish subfractions including Fr.Z-6–1–5, Fr.Z-7–1–5, Fr.Z-9–1–6, and Fr.Z-10–1–4. After TLC identification, Z-6–3 (675.4 mg), Z-7–4 (1.30 g), Z-9–4 (1.25 g), Z-9–5 (1.20 g), Z-9–6 (991.7 mg), Z-10–3 (709.9 mg), and Z-10–4 (434.8 mg) were combined and then chromatographed by MPLC with a gradient elution of MeCN-H_2_O-AcOH (v/v/v 5:95:0.1–100:0:0.1) to provide 9 subfractions (Fr.Z-A-1–9). Fr.Z-A-2 (1.43 g) was separated and purified by semipreparative HPLC using MeOH-H_2_O-TFA (10:90:0.05) to yield **24** (282.7 mg, *t*_R_ = 17.0 min). Compound **15** (2.7 mg, *t*_R_ = 16.6 min) was isolated and purified from Fr.Z-A-6 (141 mg) by semipreparative HPLC, MeCN-H_2_O-TFA (21:79:0.05). Fr.Z-A-5 (723.9 mg) was separated and purified by semipreparative HPLC using MeCN-H_2_O-TFA (19:81:0.05) to yield **1** (21.0 mg, *t*_R_ = 27.0 min) and **14** (50.5 mg, *t*_R_ = 13.5 min). Fr.Z-A-4 (613.8 mg) was separated and purified by semipreparative HPLC using MeCN-H_2_O-TFA (13:87:0.05) to yield **25** (76.1 mg, *t*_R_ = 16.8 min).

Aconicorine A (**1**). Needle-like crystals; [α]^25^_D_ = 10.5 (c 0.5, MeOH); UV (MeOH) λmax (log ε) 195 (0.61), 266 (0.39) nm; ^1^H (Pyridine-*d*_5_ and DMSO-*d*_6_, 600 MHz) and ^13^C (Pyridine-*d*_5_ and DMSO-*d*_6_, 150 MHz) NMR data see Table 1; HRESIMS at *m*/*z* 752.2586 [M + H]^+^ (calcd for C_35_H_45_NO_15_S *m*/*z* 752.2583).

Aconicorine B (**2**). White powder; [α]^25^_D_ = 5.5 (c 0.5, MeOH); UV (MeOH) λmax (log ε) 195 (0.83), 229 (0.23) nm; ^1^H (Methanol-*d*_4_, 600 MHz) and ^13^C (Methanol-*d*_4_, 150 MHz) NMR data see Tables 2 and 4; HRESIMS at *m*/*z* 672.2114 [M + H]^+^ (calcd for C_33_H_37_NO_12_S *m*/*z* 672.2109).

Aconicorine C (**3**). White powder; [α]^25^_D_ = 8.5 (c 0.5, MeOH); UV (MeOH) λmax (log ε) 195 (1.10), 230 (0.29) nm; ^1^H (Methanol-*d*_4_, 600 MHz) and ^13^C (Methanol-*d*_4_, 150 MHz) NMR data see Tables 2 and 4; HRESIMS at *m*/*z* 688.2055 [M + H]^+^ (calcd for C_33_H_37_NO_13_S *m*/*z* 688.2058).

Aconicorine D (**4**). White powder; [α]^25^_D_ = 15.1 (c 0.5, MeOH); UV (MeOH) λmax (log ε) 195 (1.65), 229 (0.25) nm; ^1^H (DMSO-*d*_6_, 600 MHz) and ^13^C (DMSO-*d*_6_, 150 MHz) NMR data see Tables 2 and 4; HRESIMS at *m*/*z* 588.1901 [M + H]^+^ (calcd for C_29_H_33_NO_10_S *m*/*z* 588.1898).

Aconicorine E (**5**). White powder; [α]^25^_D_ = −37.0 (c 0.5, MeOH); UV (MeOH) λmax (log ε) 195 (0.41), 217 (0.10) nm; ^1^H (Methanol-*d*_4_, 600 MHz) and ^13^C (Methanol-*d*_4_, 150 MHz) NMR data see Tables 2 and 4; HRESIMS at *m*/*z* 414.2281 [M + H]^+^ (calcd for C_24_H_31_NO_5_ *m*/*z* 414.2275).

Aconicorine F (**6**). White powder; [α]^25^_D_ = 9.9 (c 0.5, MeOH); UV (MeOH) λmax (log ε) 195 (1.30), 229 (0.21) nm; ^1^H (Methanol-*d*_4_, 600 MHz) and ^13^C (Methanol-*d*_4_, 150 MHz) NMR data see Tables 3 and 7; HRESIMS at *m*/*z* 524.2282 [M + H]^+^ (calcd for C_29_H_33_NO_8_ *m*/*z* 524.2279).

Aconicorine G (**7**). White powder; [α]^25^_D_ = 0.9 (c 0.5, MeOH); UV (MeOH) λmax (log ε) 195 (1.93), 232 (0.36) nm; ^1^H (Methanol-*d*_4_, 600 MHz) and ^13^C (Methanol-*d*_4_, 150 MHz) NMR data see Tables 4 and 5; HRESIMS at *m*/*z* 450.2277 [M + H]^+^ (calcd for C_27_H_31_NO_5_ *m*/*z* 450.2275).

Aconicorine H (**8**). White powder; [α]^25^_D_ = 26.3 (c 0.5, MeOH); UV (MeOH) λmax (log ε) 195 (1.14), 232 (0.36) nm; ^1^H (Methanol-*d*_4_, 600 MHz) and ^13^C (Methanol-*d*_4_, 150 MHz) NMR data see Tables 4 and 5; HRESIMS at *m*/*z* 534.2488 [M + H]^+^ (calcd for C_31_H_35_NO_7_ *m*/*z* 534.2486).

Aconicorine I (**9**). White powder; [α]^25^_D_ = 27.3 (c 0.5, MeOH); UV (MeOH) λmax (log ε) 195 (2.74), 232 (0.79) nm; ^1^H (Methanol-*d*_4_, 600 MHz) and ^13^C (Methanol-*d*_4_, 150 MHz) NMR data see Tables 4 and 5; HRESIMS at *m*/*z* 466.2225 [M + H]^+^ (calcd for C_27_H_31_NO_6_ *m*/*z* 466.2224).

Aconicorine J (**10**). White powder; [α]^25^_D_ = −7.9 (c 0.5, MeOH); UV (MeOH) λmax (log ε) 195 (1.91), 233 (0.55) nm; ^1^H (Methanol-*d*_4_, 600 MHz) and ^13^C (Methanol-*d*_4_, 150 MHz) NMR data see Tables 6 and 7; HRESIMS at *m*/*z* 770.3178 [M]^+^ (calcd for C_43_H_48_NO_12_ *m*/*z* 770.3171).

**Table 6:** ^1^H NMR (600 MHz) Data of **10**–**12** (Methanol-*d*_4_, δ in ppm, *J* in Hz)

No	10	11	12
1	6.11, d (2.1)	6.09, d (2.3)	6.08, d (2.3)
2	5.84, m	5.82, m	5.81, m
3	1.95, d (16.2)	1.94, d (16.2)	1.93, d (16.2)
	2.08, dd (4.7, 16.2)	2.05, dd (4.8, 16.2)	2.05, dd (4.8, 16.2)
5	2.90, s	2.87, s	2.83, s
6	3.98, d (2.4)	4.00, d (3.3)	3.93, d (2.9)
7	4.26, d (3.4)	4.57, d (3.8)	4.14, d (2.5)
9	3.28, m	3.19, dd (2.2, 9.5)	3.13, m
11	5.43, d (9.5)	5.38, m	5.43, d (9.2)
12	2.80, d (1.9)	2.74, d (2.7)	2.64, d (2.6)
13	5.58, brd (9.7)	5.51, brd (9.9)	5.49, brd (10.0)
14	3.69, d (9.6)	3.60, m	3.49, dd (1.4, 10.0)
15	6.00, s	4.64, s	2.38, dt (18.2)
			2.88, dt (18.2)
17	5.16, d (1.5)	5.30, m	5.00, s
	5.31, d (1.8)	5.45, m	5.08, s
18	1.17, s	1.16, s	1.15, s
19	5.39, s	5.38, s	5.37, s
20	4.31, s	4.25, s	4.21, s
N-CH_3_	3.10, s	3.09, s	3.13, s
	1-OAc	1-OAc	1-OAc
	2.17, s	2.17, s	2.17, s
	11-OAc	11-OAc	11-OAc
	2.01, s	2.02, s	2.02, s
	2-OBz	2-OBz	2-OBz
2′/6′	7.49, d (7.7)	7.47, d (7.7)	7.47, d (7.5)
3′/5′	7.36, t (7.7)	7.35, t (7.7)	7.35, t (7.7)
4′	7.56, t (7.5)	7.54, t (7.5)	7.54, t (7.4)
	13-OBz	13-OBz	13-OBz
2″/6″	7.76, d (7.6)	7.74, d (7.7)	7.75, d (7.5)
3″/5″	7.07, t (7.7)	7.06, t (7.7)	7.06, t (7.7)
4″	7.30, t (7.3)	7.29, t (7.2)	7.29, t (7.7)
	15-OiBu		
	2.78, m		
	1.29, d (2.5)		
	1.27, d (2.5)		

**Table 7:** ^13^C NMR (150 MHz) Data of **6**, **10**–**15** (Methanol-*d*_4_, δ in ppm, *J* in Hz)

No	6^a^	10^a^	11^a^	12^a^	13^a^	14^a^	15^a^
1	32.8	69.9	70.3	70.4	32.7	66.5	32.9
2	69.8	67.5	67.8	67.9	69.8	73.6	66.9
3	73.0	32.8	33.2	33.3	72.9	33.5	77.1
4	43.2	41.2	41.4	41.5	43.3	42.8	48.4
5	56.0	53.4	53.7	53.9	55.9	55.1	58.9
6	74.5	69.4	69.7	70.1	74.2	63.4	68.1
7	29.6	62.3	62.7	67.3	29.2	34.6	70.2
8	49.2	53.6	55.4	51.0	47.5	43.5	51.8
9	49.8	46.2	46.5	47.2	50.3	53.5	47.0
10	52.9	55.0	55.5	55.5	52.9	55.6	46.6
11	74.8	74.0	74.3	75.7	74.6	71.4	77.3
12	45.4	46.3	46.8	47.1	45.2	43.5	45.6
13	74.3	73.4	74.2	74.5	74.0	71.6	82.7
14	44.0	41.5	41.0	45.0	43.9	48.2	83.6
15	70.2	66.7	65.2	30.4	71.8	37.6	121.1
16	148.2	143.5	148.0	142.9	144.4	73.4	146.9
17	116.0	115.6	114.4	112.1	118.7	67.4	62.8
18	26.0	21.4	21.8	21.9	26.0	22.1	19.1
19	71.7	102.6	102.6	102.6	71.7	93.1	91.3
20	78.3	71.8	72.0	72.4	78.1	64.2	67.3
N-CH_3_		36.2	36.2	36.3			
	13-OBz	2-OBz	2-OBz	2-OBz	13-OBz	2-OBz	3-OCin
1′	130.8	129.3	129.6	129.6	130.7	130.8	135.7
2′/6′	131.2	130.2	130.4	130.5	131.3	130.6	129.2
3′/5′	129.7	129.5	129.7	129.8	129.7	130.1	130.1
4′	134.7	134.4	134.6	134.7	134.7	134.9	131.7
7′	167.7	164.9	165.2	165.3	167.6	166.3	146.8
8′							118.5
9′							167.7
		13-OBz	13-OBz	13-OBz			
1’″		129.3	129.5	129.6			
2″/6″		130.3	130.5	130.6			
3″/5″		129.2	129.4	129.5			
4″		134.4	134.5	134.6			
7″		167.1	167.5	167.6			

a Data for **6**: 11-OAc (δ_C_ 171.7, 21.0); Data for **10**: 1-OAc (δ_C_ 171.4, 20.8), 11-OAc (δ_C_ 171.5, 21.1), 15-OiBu (δ_C_ 177.6, 34.9, 19.2, 19.0); Data for **11**: 1-OAc (δ_C_ 171.7, 21.1), 11-OAc (δ_C_ 171.9, 21.5); Data for **12**: 1-OAc (δ_C_ 172.1, 21.2), 11-OAc (δ_C_ 172.1, 21.6); Data for **13**: 11-OAc (δ_C_ 171.7, 21.0), 15-OAc (δ_C_ 172.3, 20.9); Data for **14**: 13-OAc (δ_C_ 171.7, 21.0); Data for **15**: 13-OAc (δ_C_ 172.0, 21.1).

Aconicorine K (**11**). White powder; [α]^25^_D_ = −24.5 (c 0.5, MeOH); UV (MeOH) λmax (log ε) 195 (2.06), 234 (0.59) nm; ^1^H (Methanol-*d*_4_, 600 MHz) and ^13^C (Methanol-*d*_4_, 150 MHz) NMR data see Tables 6 and 7; HRESIMS at *m*/*z* 700.2747 [M]^+^ (calcd for C_39_H_42_NO_11_ *m*/*z* 700.2752).

Aconicorine L (**12**). White powder; [α]^25^_D_ = −17.3 (c 0.5, MeOH); UV (MeOH) λmax (log ε) 195 (2.38), 234 (0.69) nm; ^1^H (Methanol-*d*_4_, 600 MHz) and ^13^C (Methanol-*d*_4_, 150 MHz) NMR data see Tables 6 and 7; HRESIMS at *m*/*z* 684.2805 [M]^+^ (calcd for C_39_H_42_NO_10_ *m*/*z* 684.2803).

Aconicorine M (**13**). White powder; [α]^25^_D_ = −38.3 (c 0.5, MeOH); UV (MeOH) λmax (log ε) 195 (1.32), 232 (0.38) nm; ^1^H (Methanol-*d*_4_, 600 MHz) and ^13^C (Methanol-*d*_4_, 150 MHz) NMR data see Tables 3 and 7; HRESIMS at *m*/*z* 566.2387 [M + H]^+^ (calcd for C_31_H_35_NO_9_ *m*/*z* 566.2385).

Aconicorine N (**14**). White powder; [α]^25^_D_ = 29.3 (c 0.5, MeOH); UV (MeOH) λmax (log ε) 195 (1.91), 229 (0.40) nm; ^1^H (Methanol-*d*_4_, 600 MHz) and ^13^C (Methanol-*d*_4_, 150 MHz) NMR data see Tables 3 and 7; HRESIMS at *m*/*z* 542.2388 [M + H]^+^ (calcd for C_29_H_35_NO_9_ *m*/*z* 542.2385).

Aconicorine O (**15**). White powder; [α]^25^_D_ = 27.9 (c 0.5, MeOH); UV (MeOH) λmax (log ε) 195 (0.58), 277 (0.41) nm; ^1^H (Methanol-*d*_4_, 600 MHz) and ^13^C (Methanol-*d*_4_, 150 MHz) NMR data see Tables 3 and 7; HRESIMS at *m*/*z* 582.2334 [M + H]^+^ (calcd for C_31_H_35_NO_10_ *m*/*z* 582.2333).

### X-ray Crystallographic Data

Crystallographic data for compound **1** were acquired using an XtaLAB AFC12 (RINC): Kappa single-crystal diffractometer equipped with an AtlasS2 CCD detector and Cu Kα radiation.^43^ Details of the crystal structure (CCDC number 2553859) have been stored at the Cambridge Crystallography Data Center.

Crystal Data of **1**: C_35_H_45_NO_15_S, orthorhombic, space group *P*2_1_2_1_2_1_, *a* = 11.10644(11) Å, *b* = 14.17941(17) Å, *c* = 27.4838(3) Å, *V* = 4328.22(8) Å^3^, *Z* = 4, *T* = 199.97(10) K, μ­(Cu Kα) = 1.317 mm^–1^, *Dcalc* = 1.278 g/cm^3^, 43944 reflections measured (7.014° ≤ 2Θ ≤ 146.828°), 8345 unique (*R*_int_ = 0.0401, *R*_sigma_ = 0.0318) which were used in all calculations. The final *R*_1_ was 0.0455 (*I* > 2σ­(I)), and *wR*_2_ was 0.1352.

### Absolute Configuration Analysis of Sugar

The absolute configuration of sugars in **1** was determined using methods previously reported in the literature.^44^ Compound **1** (1 mg) underwent acid hydrolysis in 1 N HCl (0.1 mL) at 80 °C for 4 h, followed by solvent removal. The residue was dissolved in a pyridine solution of L-cysteine methyl ester hydrochloride (0.5 mg in 0.1 mL) at 60 °C for 1 h, followed by a secondary derivatization reaction with phenyl isothiocyanate (0.1 mL, 0.5 mg/mL in pyridine) at 60 °C for 1 h. The resulting reaction product (5 μL) was determined under the following chromatographic conditions: Symmetry C18 column (5 μm, 4.6 × 250 mm, Waters), DAD detection wavelength 250 nm, column temperature 35 °C, mobile phase 25% MeCN 0.1% phosphoric acid solution, and flow rate 0.8 mL/min. The presence of d-glucose in **1** was confirmed by comparing the retention time (16.98 min) of its derivative to those of standard d-glucose (17.09 min, TCI) and l-glucose (16.02 min, MACKLIN).

### Cell Lines and Culture Conditions

Human breast cancer cell line (MDA-MB-231) was sourced from the American Type Culture Collection (ATCC, Rockville, Maryland, USA). Cells were incubated in DMEM high-glucose medium supplemented with 10% fetal bovine serum (FBS) and 1% penicillin–streptomycin antibiotics under standard humidified conditions at 37 °C, 5% CO_2_. Upon achieving 80–90% confluence, the cells were subcultured at intervals of 2–3 days.

### In Vitro Cytotoxicity Assay

MDA-MB-231 cells were initially plated in 96-well culture plates at a density of 5 × 10^3^ cells/well and cultured overnight. Following a 48 h incubation with a series of concentrations (10, 20, and 40 μM) of compounds **1**–**25**, cell viability was evaluated using the MTT assay as previously reported.^7^

### Western Blot Analysis

For protein analysis, MDA-MB-231 cells were plated in 6-well plates (5 × 10^5^ cells/well) and treated with 10 μM compounds **1**–**25** and 40 μM berberine for 48 h. Following treatment, cells were harvested and lysed using RIPA buffer. Equal amounts of proteins were electrophoresed on an 8% SDS-PAGE gel and then transferred to a PVDF membrane. The membranes were blocked and probed overnight at 4 °C with an anti-PD-L1 primary antibody (ABclone, USA). After washing three times with TBST, the blots were incubated with a secondary antibody (rabbit monoclonal) at ambient temperature for 1 h. Ultimately, the protein blots were analyzed using a LI-COR Odyssey imaging system (Lincoln, Nevada, USA).

### Immunofluorescence Staining

MDA-MB-231 cells were treated with compounds **7**, **14**, **15**, and **17** (with blank solvent and berberine as controls), followed by fixation with 4% paraformaldehyde. Following cell permeabilization and blocking, samples were probed overnight at 4 °C with anti-PD-L1 primary antibody. On the following day, the cells were incubated for 2 h with FITC-labeled goat antirabbit IgG (H+L) secondary antibody. Nuclear counterstaining was subsequently carried out using DAPI. Fluorescence imaging was conducted on an ImageXpress Micro confocal system to observe DAPI (nucleus) and FITC (target protein) channels, respectively.

## Supplementary Material



## Notes

HRMS, UV, ECD, 1D and 2D NMR spectra of compounds **1**–**15**; Key NOESY correlations of compounds **3**, **4**, **5**, **7**, **9**, **11**, **12**, **13**; Cytotoxicity assay results of compounds **1**–**25** (PDF)
