# Supplementary material for: C‑20 Diterpenoid Alkaloids with PD-L1 Inhibitory Activity from the Roots of Aconitum coreanum
Source: ACS Omega. 2026 Sep 22;11(39):59532–46. doi: 10.1021/acsomega.6c05802 (PMC13647379; doi:10.1021/acsomega.6c05802)
Supplement: Supplementary file 1 [file ao6c05802_si_001.pdf]

**Supporting Information**

# C-20 diterpenoid alkaloids with PD-L1 inhibitory activity from the roots of *Aconitum coreanum*

*Kei-Fong Ng,*[†] *Xiao-Qian Li,*[†] *Hao-Ming Xiong,*[†] *Lin Chen,*[†] *Xi Chen,*[†] *Li-Ping Bai,*[†] *Wei Zhang,*[†,‡] *Ancheng C. Huang,*[§] *Zhi-Hong Jiang,*[†,*] *and Guo-Yuan Zhu*[†,‡,*]

[†] State Key Laboratory of Mechanism and Quality of Chinese Medicine & Faculty of Chinese Medicine, Macau Institute for Applied Research in Medicine and Health, Macau University of Science and Technology, Macau 999078, People's Republic of China

[‡] Zhuhai MUST Science and Technology Research Institute, Macau University of Science and Technology, Zhuhai 519000, People's Republic of China

[§] Shenzhen Key Laboratory of Plant Genetic Engineering and Molecular Design, SUSTech–PKU Institute of Plant and Food Science, Department of Biology, School of Life Sciences, Southern University of Science and Technology, Shenzhen 518000, People's Republic of China

*Corresponding authors. E-mail addresses: zhjiang@must.edu.mo (Z.-H. Jiang), gyzhu@must.edu.mo (G.-Y. Zhu)

NMR, HRESIMS, ECD, and UV spectra of compounds **1–15** .......... 1

**Figure S1.** $^{1}$H NMR spectrum of **1** in pyridine-$d_5$ .......... 1

**Figure S2.** $^{13}$C NMR spectrum of 1 in pyridine-$d_5$ .......... 1

**Figure S3.** DEPT NMR spectrum of **1** in pyridine-$d_5$ .......... 2

**Figure S4.** HSQC spectrum of **1** .......... 2

**Figure S5.** HMBC spectrum of **1** .......... 3

**Figure S6.** $^{1}$H-$^{1}$H COSY spectrum of **1** .......... 4

**Figure S7.** NOESY spectrum of **1** .......... 5

**Figure S8.** HRESIMS spectrum of **1** .......... 5

**Figure S9.** ECD spectrum of **1** .......... 6

**Figure S10.** UV spectrum of **1** .......... 7

**Figure S11.** $^{1}$H NMR spectrum of **2** in methanol-$d_4$ .......... 7

**Figure S12.** $^{13}$C NMR spectrum of **2** in methanol-$d_4$ .......... 7

**Figure S13.** DEPT NMR spectrum of **2** in methanol-$d_4$ .......... 8

**Figure S14.** HSQC spectrum of **2** .......... 8

**Figure S15.** HMBC spectrum of **2** .......... 9

**Figure S16.** $^{1}$H-$^{1}$H COSY spectrum of **2** .......... 10

**Figure S17.** NOESY spectrum of **2** ........ 11

**Figure S18.** HRESIMS spectrum of **2** ........ 11

**Figure S19.** ECD spectrum of **2** ........ 12

**Figure S20.** UV spectrum of **2** ........ 12

**Figure S21.** $^{1}$H NMR spectrum of **3** in methanol-$d_4$ ........ 13

**Figure S22.** $^{13}$C NMR spectrum of **3** in methanol-$d_4$ ........ 13

**Figure S23.** DEPT NMR spectrum of **3** in methanol-$d_4$ ........ 14

**Figure S24.** HSQC spectrum of **3** ........ 14

**Figure S25.** HMBC spectrum of **3** ........ 15

**Figure S26.** $^{1}$H-$^{1}$H COSY spectrum of **3** ........ 16

**Figure S27.** NOESY spectrum of **3** ........ 17

**Figure S28.** HRESIMS spectrum of **3** ........ 17

**Figure S29.** ECD spectrum of **3** ........ 18

**Figure S30.** UV spectrum of **3** ........ 19

**Figure S31.** $^{1}$H NMR spectrum of **4** in DMSO-$d_6$ ........ 19

**Figure S32.** $^{13}$C NMR spectrum of **4** in DMSO-$d_6$ ........ 19

**Figure S33.** DEPT NMR spectrum of **4** in DMSO-$d_6$ ........ 20

**Figure S34.** HSQC spectrum of **4** ........ 20

**Figure S35.** HMBC spectrum of **4** .......... 21

**Figure S36.** $^1$H-$^1$H COSY spectrum of **4** .......... 22

**Figure S37.** NOESY spectrum of **4** .......... 23

**Figure S38.** HRESIMS spectrum of **4** .......... 23

**Figure S39.** ECD spectrum of **4** .......... 24

**Figure S40.** UV spectrum of **4** .......... 24

**Figure S41.** $^1$H NMR spectrum of **5** in methanol-$d_4$ .......... 25

**Figure S42.** $^{13}$C NMR spectrum of **5** in methanol-$d_4$ .......... 25

**Figure S43.** DEPT NMR spectrum of **5** in methanol-$d_4$ .......... 26

**Figure S44.** HSQC spectrum of **5** .......... 26

**Figure S45.** HMBC spectrum of **5** .......... 27

**Figure S46.** $^1$H-$^1$H COSY spectrum of **5** .......... 28

**Figure S47.** NOESY spectrum of **5** .......... 29

**Figure S48.** HRESIMS spectrum of **5** .......... 29

**Figure S49.** ECD spectrum of **5** .......... 30

**Figure S50.** UV spectrum of **5** .......... 30

**Figure S51.** $^1$H NMR spectrum of **6** in methanol-$d_4$ .......... 31

**Figure S52.** $^{13}$C NMR spectrum of **6** in methanol-$d_4$ .......... 31

**Figure S53.** DEPT NMR spectrum of **6** in methanol-$d_4$ ........ 32

**Figure S54.** HSQC spectrum of **6** ........ 32

**Figure S55.** HMBC spectrum of **6** ........ 33

**Figure S56.** $^1$H-$^1$H COSY spectrum of **6** ........ 34

**Figure S57.** NOESY spectrum of **6** ........ 35

**Figure S58.** HRESIMS spectrum of **6** ........ 35

**Figure S59.** ECD spectrum of **6** ........ 36

**Figure S60.** UV spectrum of **6** ........ 36

**Figure S61.** $^1$H NMR spectrum of **7** in methanol-$d_4$ ........ 37

**Figure S62.** $^{13}$C NMR spectrum of **7** in methanol-$d_4$ ........ 37

**Figure S63.** DEPT NMR spectrum of **7** in methanol-$d_4$ ........ 38

**Figure S64.** HSQC spectrum of **7** ........ 38

**Figure S65.** HMBC spectrum of **7** ........ 39

**Figure S66.** $^1$H-$^1$H COSY spectrum of **7** ........ 40

**Figure S67.** NOESY spectrum of **7** ........ 41

**Figure S68.** HRESIMS spectrum of **7** ........ 41

**Figure S69.** ECD spectrum of **7** ........ 42

**Figure S70.** UV spectrum of **7** ........ 43

**Figure S71.** $^{1}H$ NMR spectrum of **8** in methanol-$d_4$ ........................................................43

**Figure S72.** $^{13}C$ NMR spectrum of **8** in methanol-$d_4$ ........................................................43

**Figure S73.** DEPT NMR spectrum of **8** in methanol-$d_4$ ....................................................44

**Figure S74.** HSQC spectrum of **8**.........................................................................44

**Figure S75.** HMBC spectrum of **8**.........................................................................45

**Figure S76.** $^{1}H$-$^{1}H$ COSY spectrum of **8** ..........................................................46

**Figure S77.** NOESY spectrum of **8** .......................................................................47

**Figure S78.** HRESIMS spectrum of **8**....................................................................47

**Figure S79.** ECD spectrum of **8** .........................................................................48

**Figure S80.** UV spectrum of **8** ...........................................................................48

**Figure S81.** $^{1}H$ NMR spectrum of **9** in methanol-$d_4$ ........................................................49

**Figure S82.** $^{13}C$ NMR spectrum of **9** in methanol-$d_4$ ........................................................49

**Figure S83.** DEPT NMR spectrum of **9** in methanol-$d_4$ ....................................................50

**Figure S84.** HSQC spectrum of **9**.........................................................................50

**Figure S85.** HMBC spectrum of **9**.........................................................................51

**Figure S86.** $^{1}H$-$^{1}H$ COSY spectrum of **9** ..........................................................52

**Figure S87.** NOESY spectrum of **9** .......................................................................53

**Figure S88.** HRESIMS spectrum of **9**....................................................................53

**Figure S89.** ECD spectrum of **9** ........................................................................ 54

**Figure S90.** UV spectrum of **9** ........................................................................ 54

**Figure S91.** $^{1}$H NMR spectrum of **10** in methanol-$d_4$ ........................................................ 55

**Figure S92.** $^{13}$C NMR spectrum of **10** in methanol-$d_4$ ........................................................ 55

**Figure S93.** DEPT NMR spectrum of **10** in methanol-$d_4$ ........................................................ 56

**Figure S94.** HSQC spectrum of **10** ........................................................................ 56

**Figure S95.** HMBC spectrum of **10** ........................................................................ 57

**Figure S96.** $^{1}$H-$^{1}$H COSY spectrum of **10** ........................................................................ 58

**Figure S97.** NOESY spectrum of **10** ........................................................................ 59

**Figure S98.** HRESIMS spectrum of **10** ........................................................................ 59

**Figure S99.** ECD spectrum of **10** ........................................................................ 60

**Figure S100.** UV spectrum of **10** ........................................................................ 60

**Figure S101.** $^{1}$H NMR spectrum of **11** in methanol-$d_4$ ........................................................ 61

**Figure S102.** $^{13}$C NMR spectrum of **11** in methanol-$d_4$ ........................................................ 61

**Figure S103.** DEPT NMR spectrum of **11** in methanol-$d_4$ ........................................................ 62

**Figure S104.** HSQC spectrum of **11** ........................................................................ 62

**Figure S105.** HMBC spectrum of **11** ........................................................................ 63

**Figure S106.** $^{1}$H-$^{1}$H COSY spectrum of **11** ........................................................................ 64

**Figure S107.** NOESY spectrum of **11**......65

**Figure S108.** HRESIMS spectrum of **11**......65

**Figure S109.** ECD spectrum of **11**......66

**Figure S110.** UV spectrum of **11**......66

**Figure S111.** $^{1}$H NMR spectrum of **12** in methanol-$d_4$......67

**Figure S112.** $^{13}$C NMR spectrum of **12** in methanol-$d_4$......67

**Figure S113.** DEPT NMR spectrum of **12** in methanol-$d_4$......68

**Figure S115.** HMBC spectrum of **12**......69

**Figure S116.** $^{1}$H-$^{1}$H COSY spectrum of **12**......70

**Figure S117.** NOESY spectrum of **12**......71

**Figure S118.** HRESIMS spectrum of **12**......71

**Figure S119.** ECD spectrum of **12**......72

**Figure S120.** UV spectrum of **12**......72

**Figure S121.** $^{1}$H NMR spectrum of **13** in methanol-$d_4$......73

**Figure S122.** $^{13}$C NMR spectrum of **13** in methanol-$d_4$......73

**Figure S123.** DEPT NMR spectrum of **13** in methanol-$d_4$......74

**Figure S124.** HSQC spectrum of **13**......74

**Figure S125.** HMBC spectrum of **13**......75

**Figure S126.** $^1$H-$^1$H COSY spectrum of **13** ........................................................................76

**Figure S127.** NOESY spectrum of **13**...............................................................................77

**Figure S128.** HRESIMS spectrum of **13** ...........................................................................77

**Figure S129.** ECD spectrum of **13** ....................................................................................78

**Figure S130.** UV spectrum of **13** .......................................................................................78

**Figure S131.** $^1$H NMR spectrum of **14** in methanol-$d_4$.........................................................79

**Figure S132.** $^{13}$C NMR spectrum of **14** in methanol-$d_4$.......................................................79

**Figure S133.** DEPT NMR spectrum of **14** in methanol-$d_4$ ................................................80

**Figure S134.** HSQC spectrum of **14** ..................................................................................80

**Figure S135.** HMBC spectrum of **14** ..................................................................................81

**Figure S136.** $^1$H-$^1$H COSY spectrum of **14** ........................................................................82

**Figure S137.** NOESY spectrum of **14**...............................................................................83

**Figure S138.** HRESIMS spectrum of **14** ...........................................................................83

**Figure S139.** ECD spectrum of **14** ....................................................................................84

**Figure S140.** UV spectrum of **14** .......................................................................................84

**Figure S141.** $^1$H NMR spectrum of **15** in methanol-$d_4$.........................................................85

**Figure S142.** $^{13}$C NMR spectrum of **15** in methanol-$d_4$.......................................................85

**Figure S143.** DEPT NMR spectrum of **15** in methanol-$d_4$ ................................................86

**Figure S144.** HSQC spectrum of **15** .................................................................. 86

**Figure S145.** HMBC spectrum of **15** .................................................................. 87

**Figure S146.** $^{1}$H-$^{1}$H COSY spectrum of **15** .................................................................. 88

**Figure S147.** NOESY spectrum of **15** .................................................................. 89

**Figure S148.** HRESIMS spectrum of **15** .................................................................. 89

**Figure S149.** ECD spectrum of **15** .................................................................. 90

**Figure S150.** UV spectrum of **15** .................................................................. 90

**Figure S151.** Key NOESY correlations of compounds **3**, **4**, **5**, **7**, **9**, **11**, **12**, **13** .................... 91

**Figure S152.** Cytotoxicity assay results of compounds **1−12** on MDA-MB-231 cell at a certain concentration .................................................................. 92

**Figure S153.** Cytotoxicity assay results of compounds **13−25** on MDA-MB-231 cell at a certain concentration .................................................................. 93

NMR, HRESIMS, ECD, and UV spectra of compounds **1—15**

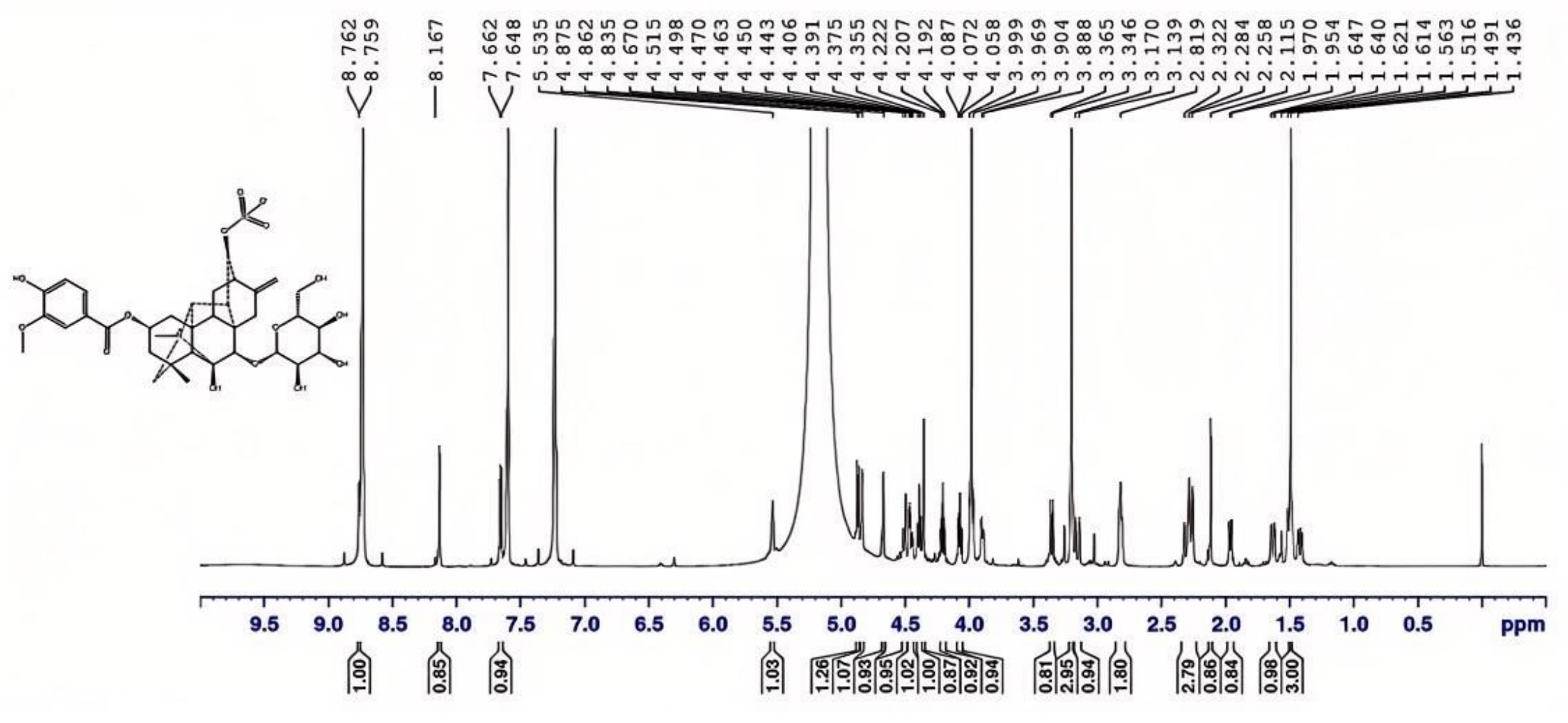

**Figure S1.** $^1H$ NMR spectrum of **1** in pyridine-$d_5$

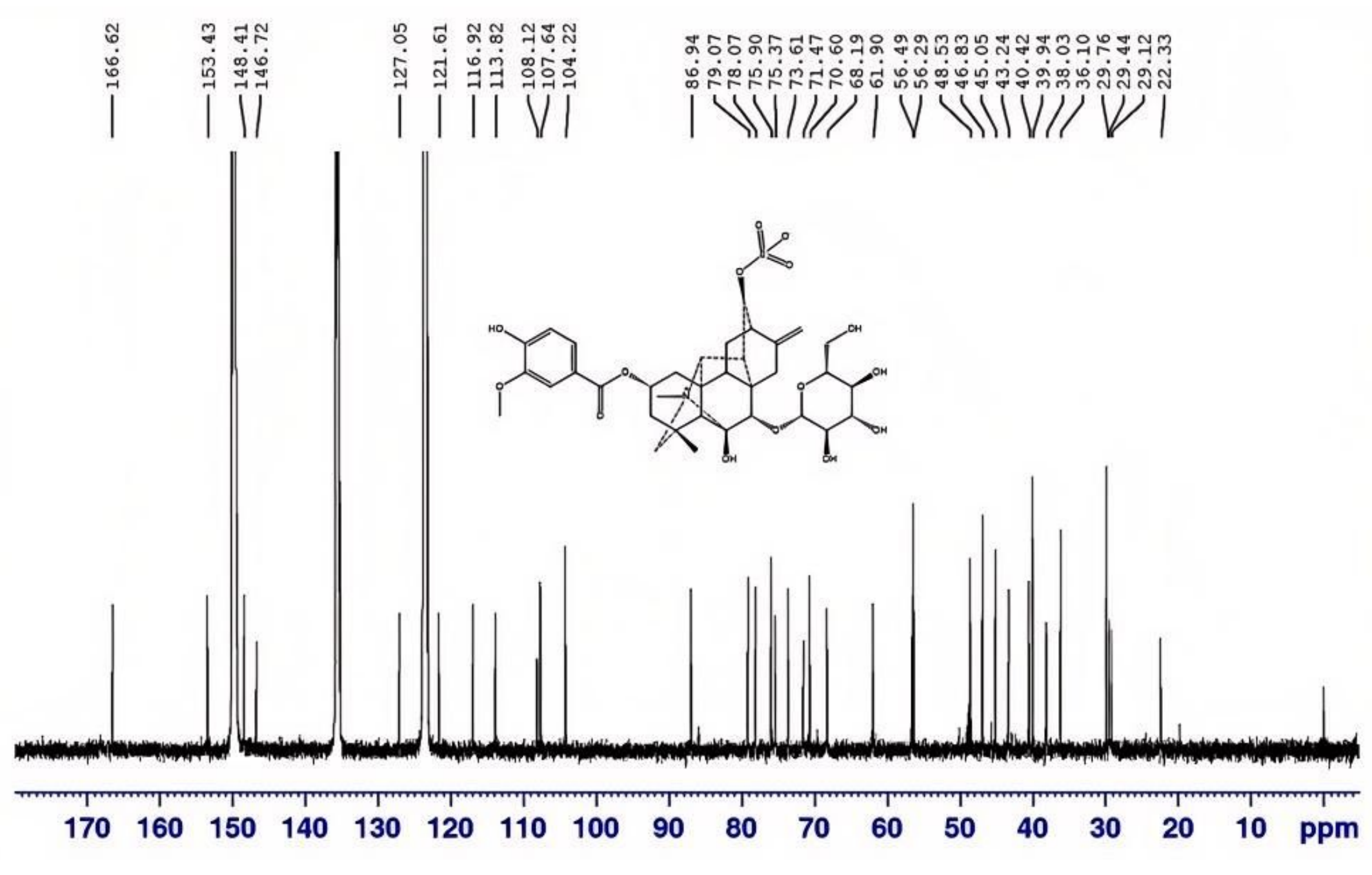

**Figure S2.** $^{13}C$ NMR spectrum of **1** in pyridine-$d_5$

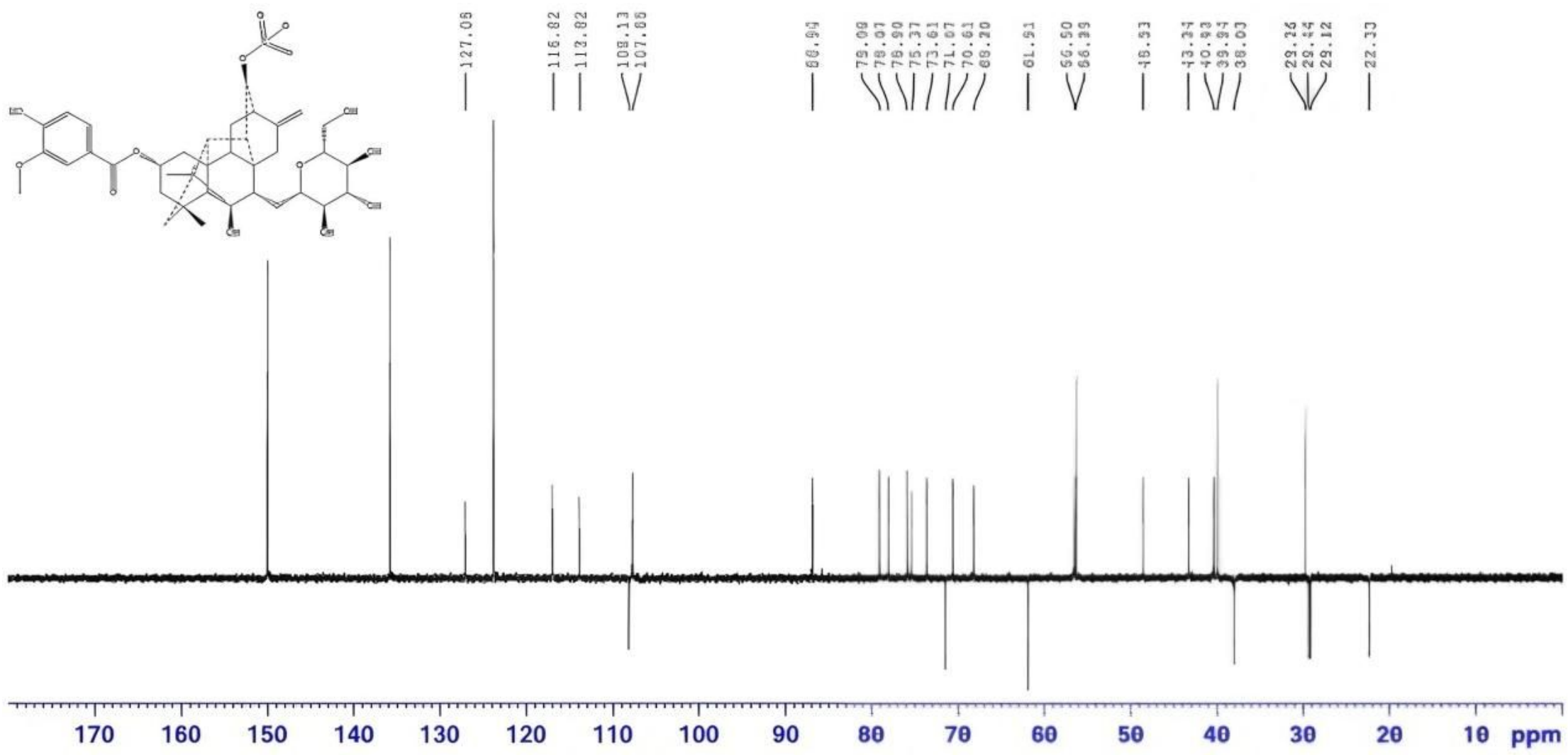

**Figure S3.** DEPT NMR spectrum of **1** in pyridine-$d_5$

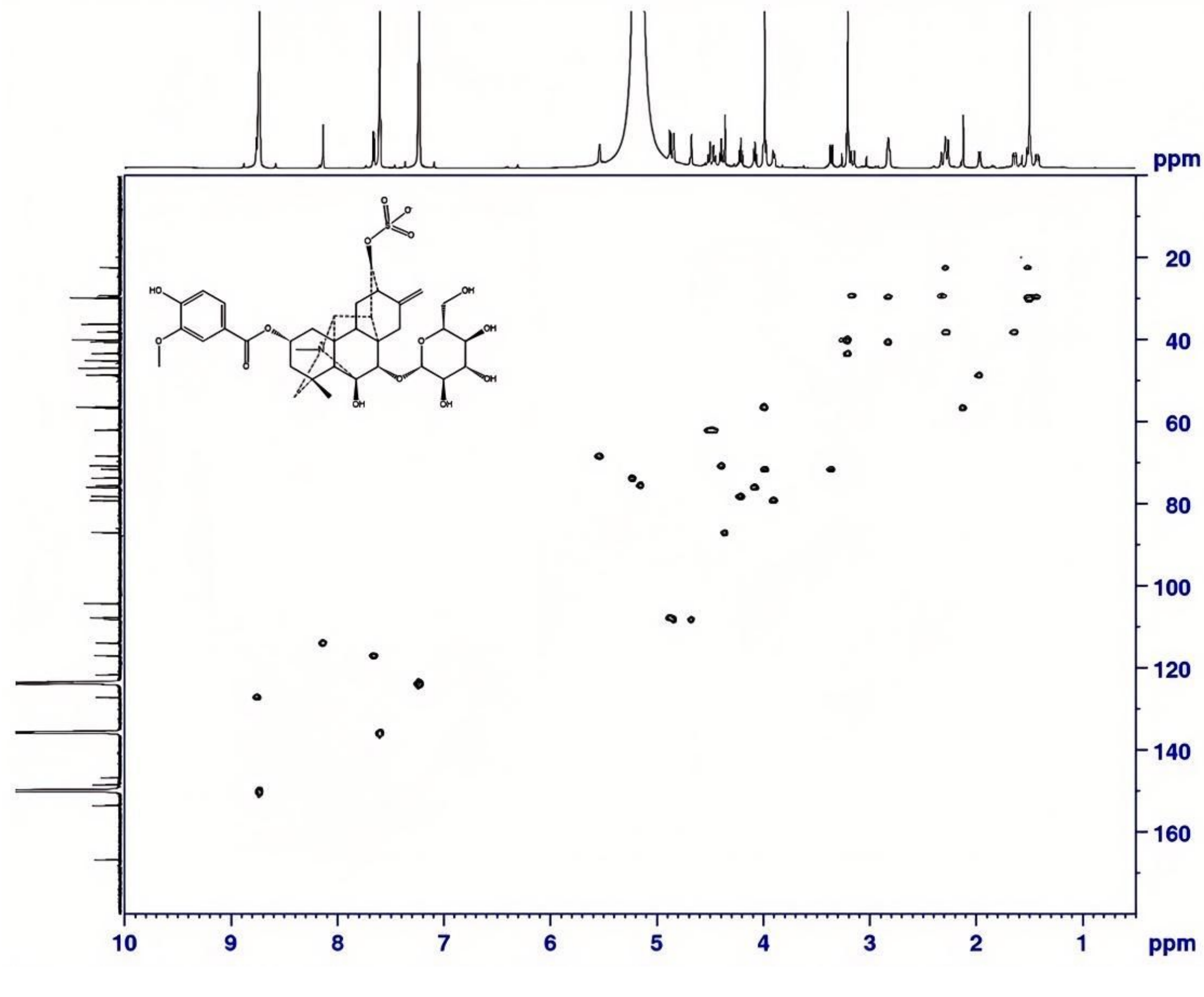

**Figure S4.** HSQC spectrum of **1**

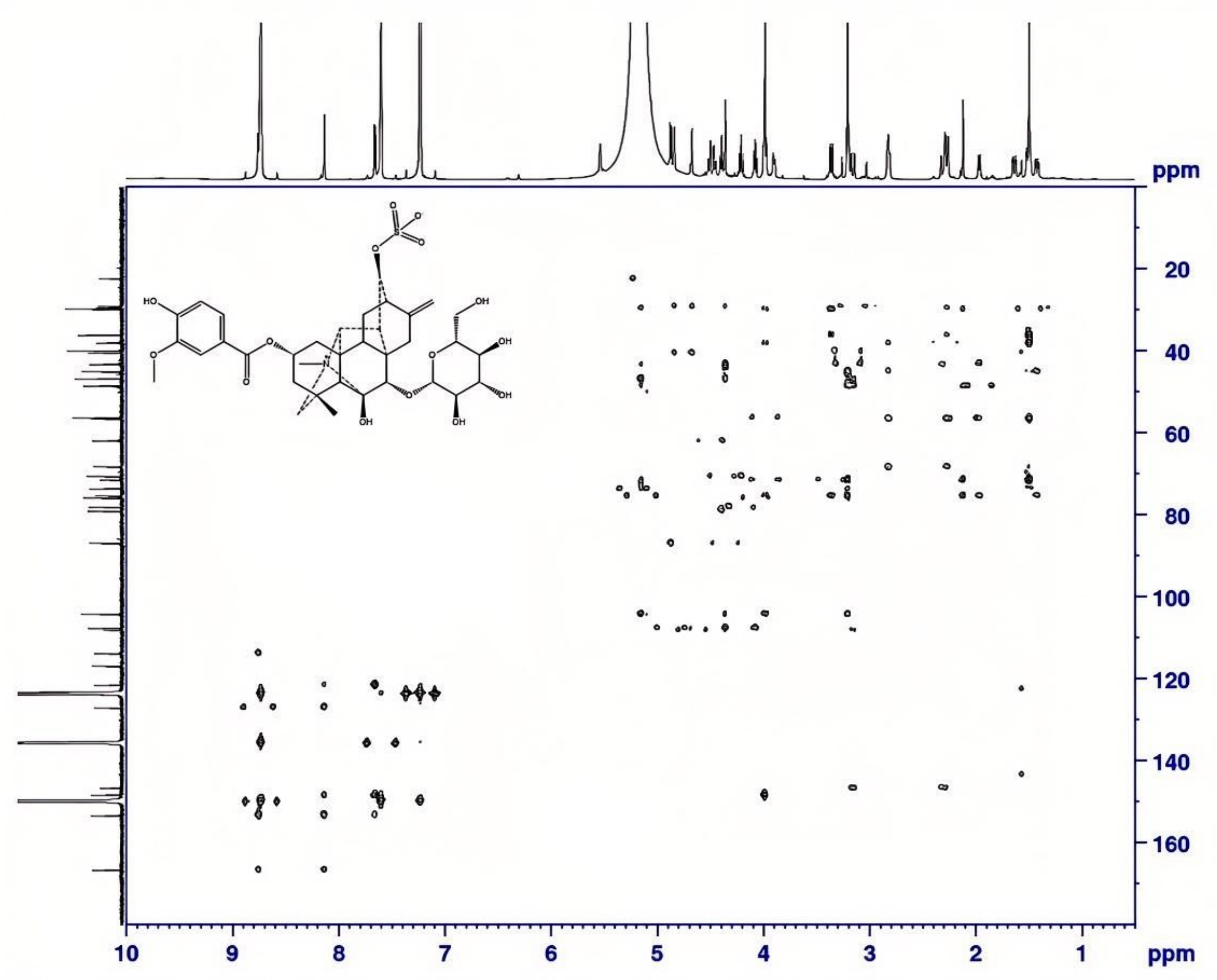

**Figure S5.** HMBC spectrum of **1**

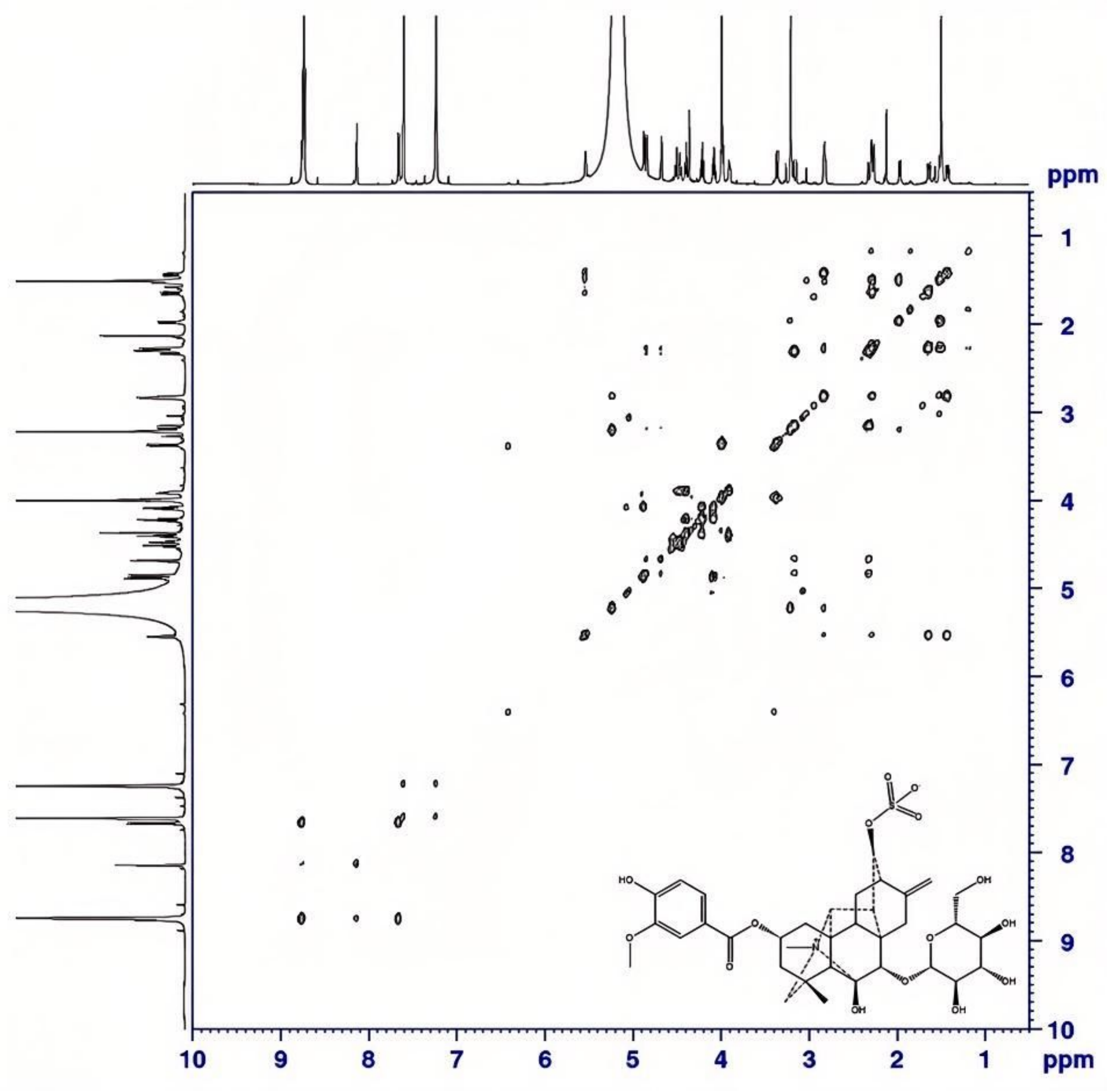


**Figure S6.** $^{1}H$-$^{1}H$ COSY spectrum of **1**

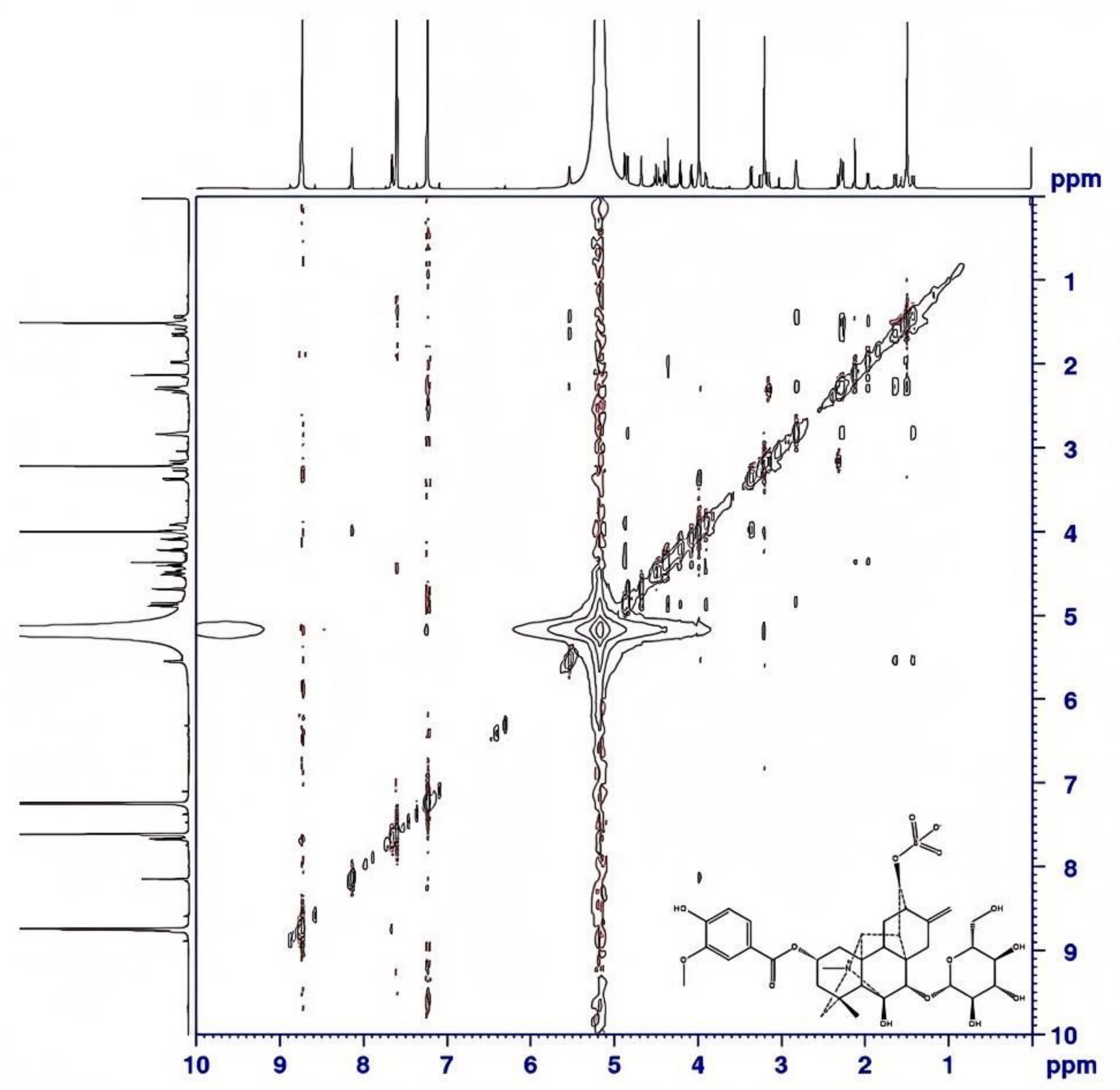


**Figure S7.** NOESY spectrum of **1**

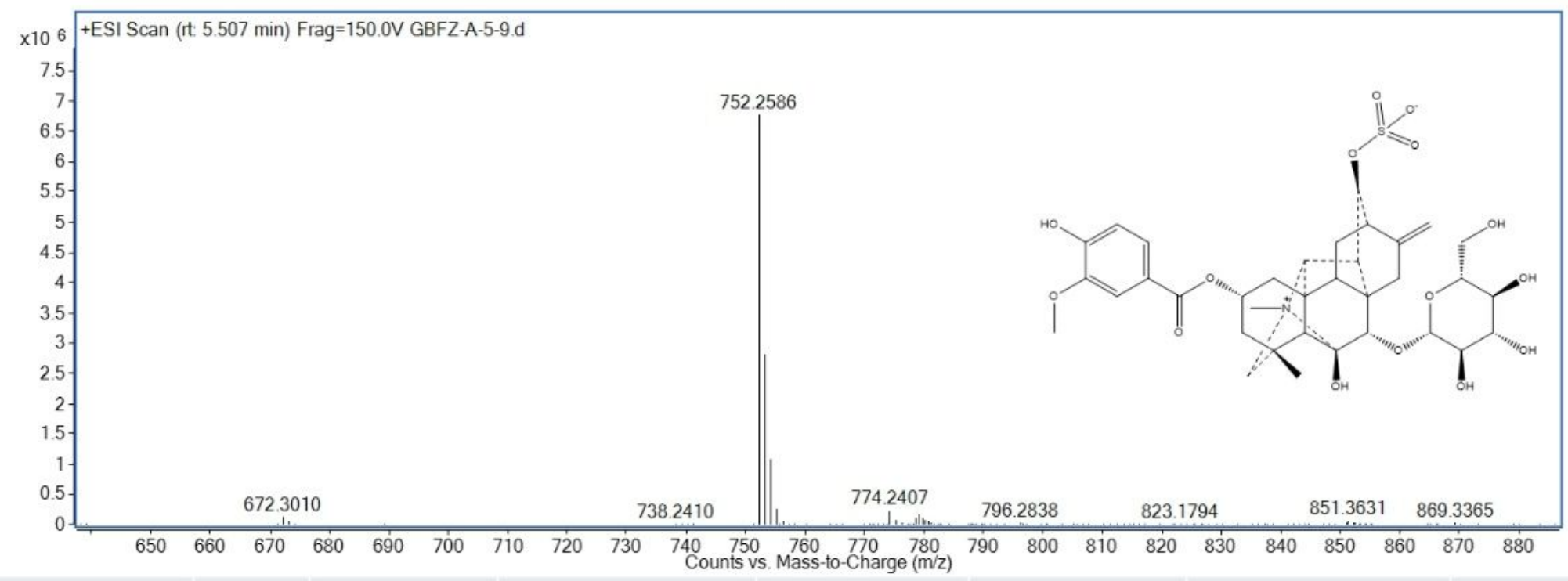


| Formula | Score | Mass | Mass (MFG) | m/z (Calc) | Diff (ppm) | Ion Formula | m/z |
|---|---|---|---|---|---|---|---|
| $C_{35}H_{45}NO_{15}S$ | 99.86 | 751.2513 | 751.2510 | 752.2583 | -0.44 | $C_{35}H_{46}NO_{15}S$ | 752.2586 |
| $C_{35}H_{45}NO_{15}S$ | 99.71 | 751.2515 | 751.2510 | 774.2402 | -0.65 | $C_{35}H_{45}NO_{15}SNa$ | 774.2407 |

**Figure S8.** HRESIMS spectrum of **1**

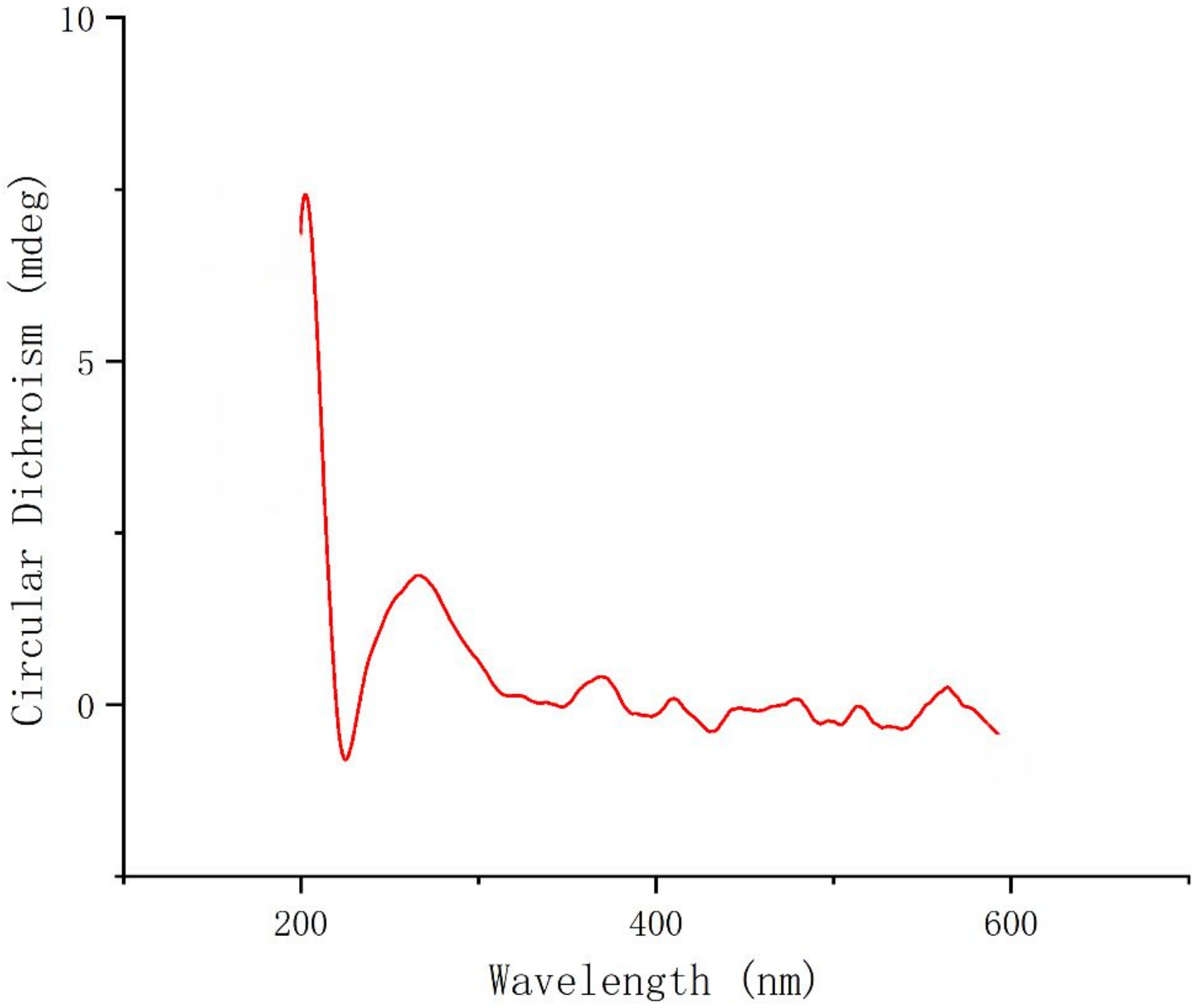


**Figure S9.** ECD spectrum of **1**

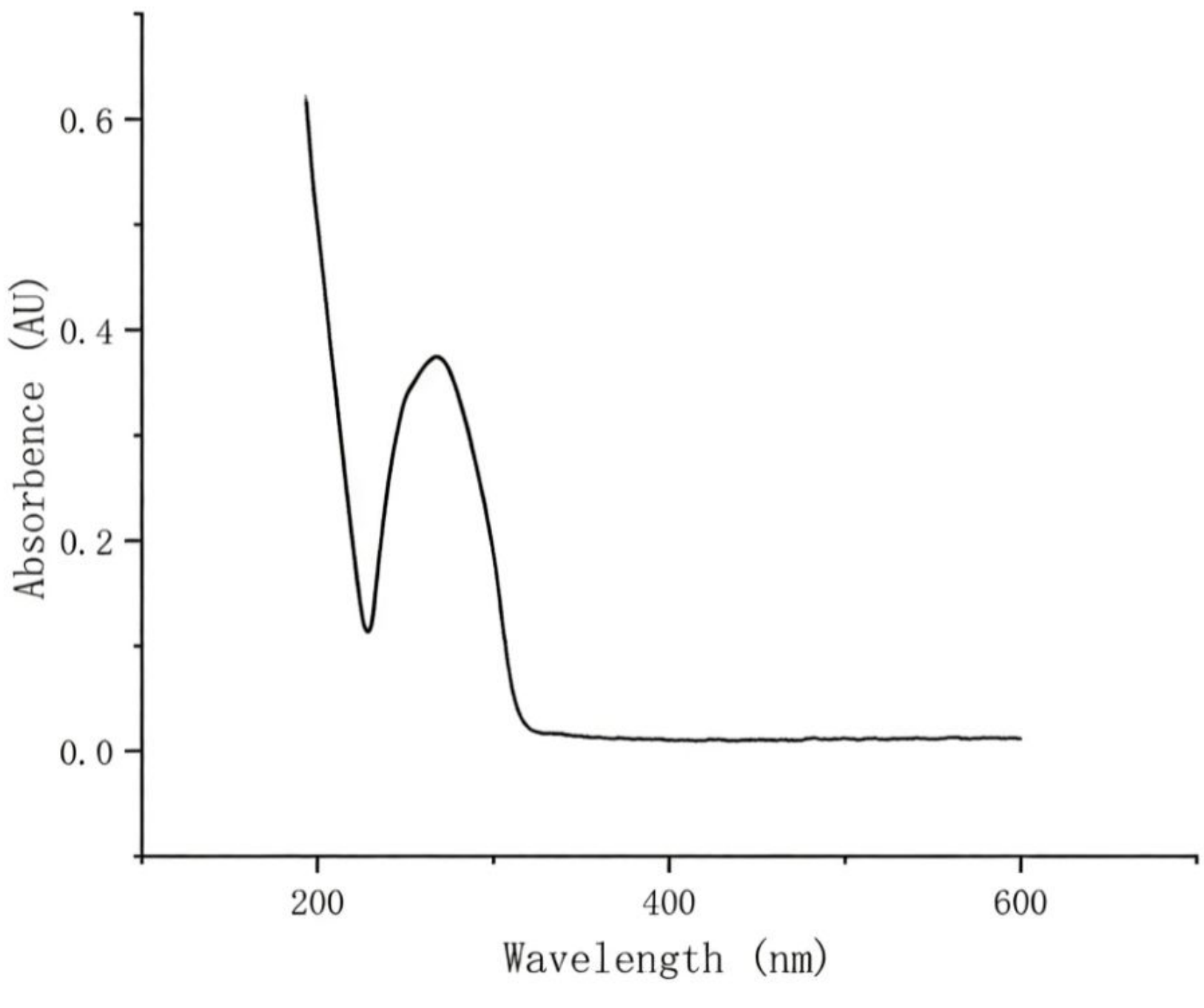

**Figure S10.** UV spectrum of **1**

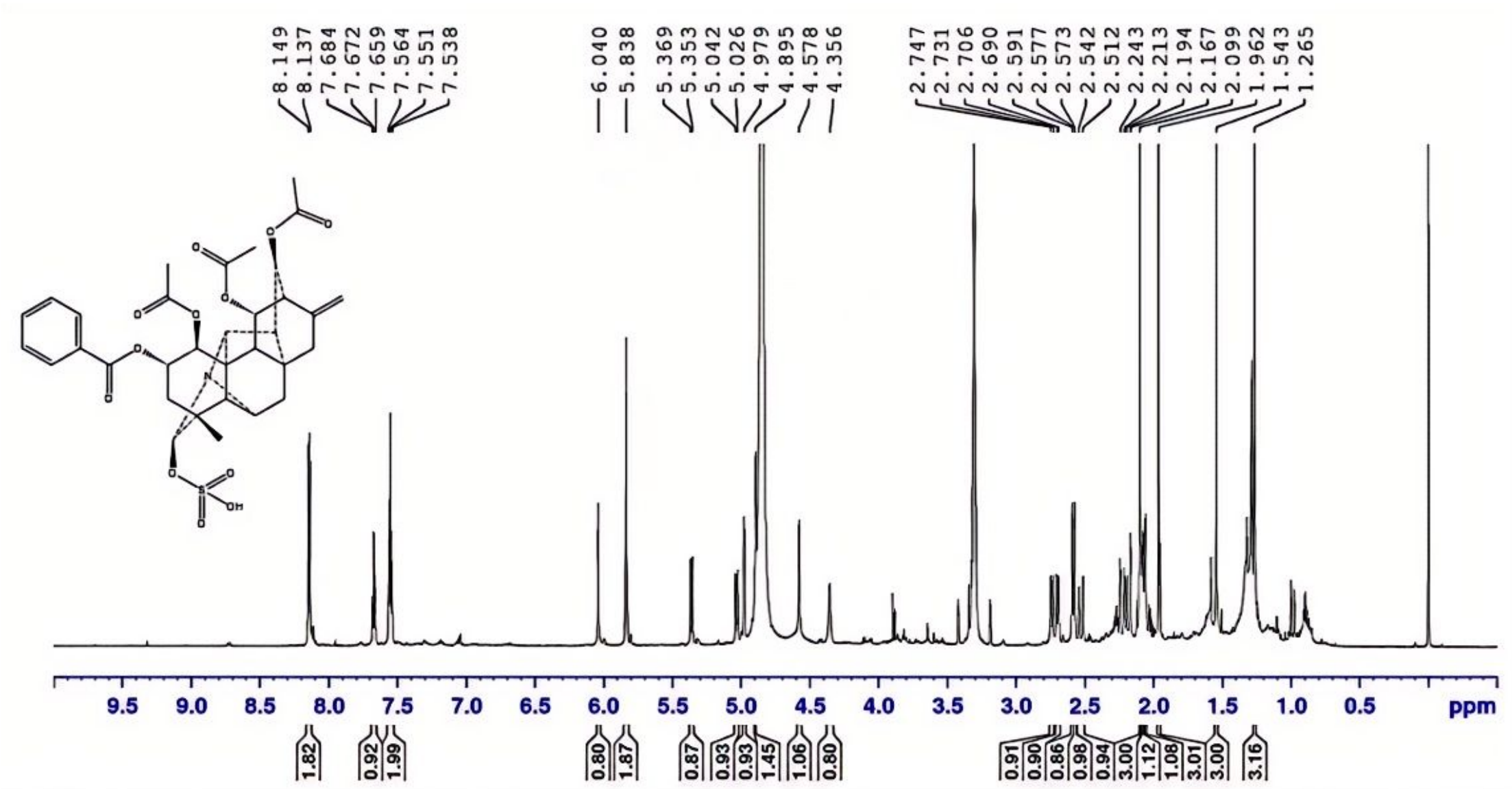


**Figure S11.** $^{1}$H NMR spectrum of **2** in methanol-$d_4$

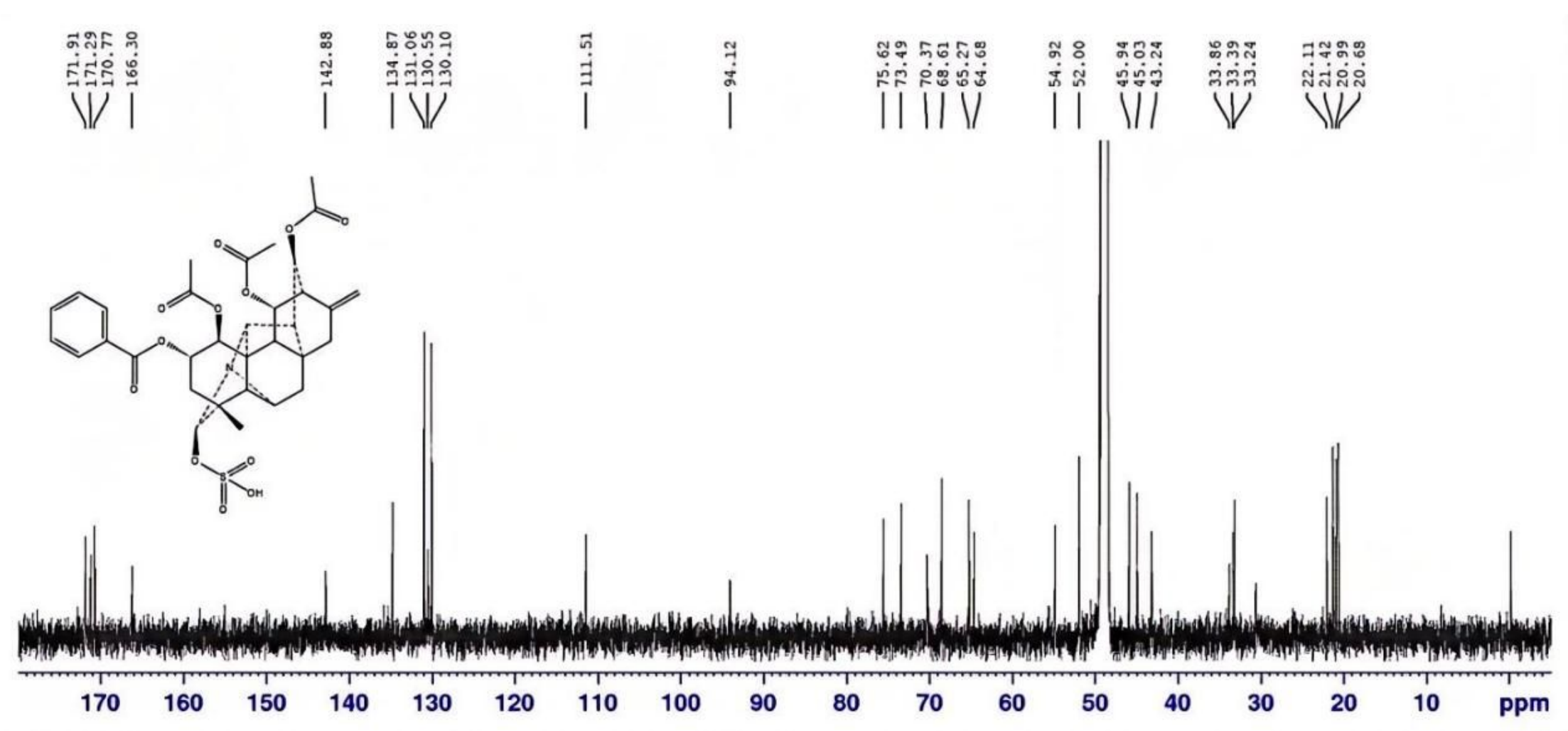


**Figure S12.** $^{13}$C NMR spectrum of **2** in methanol-$d_4$

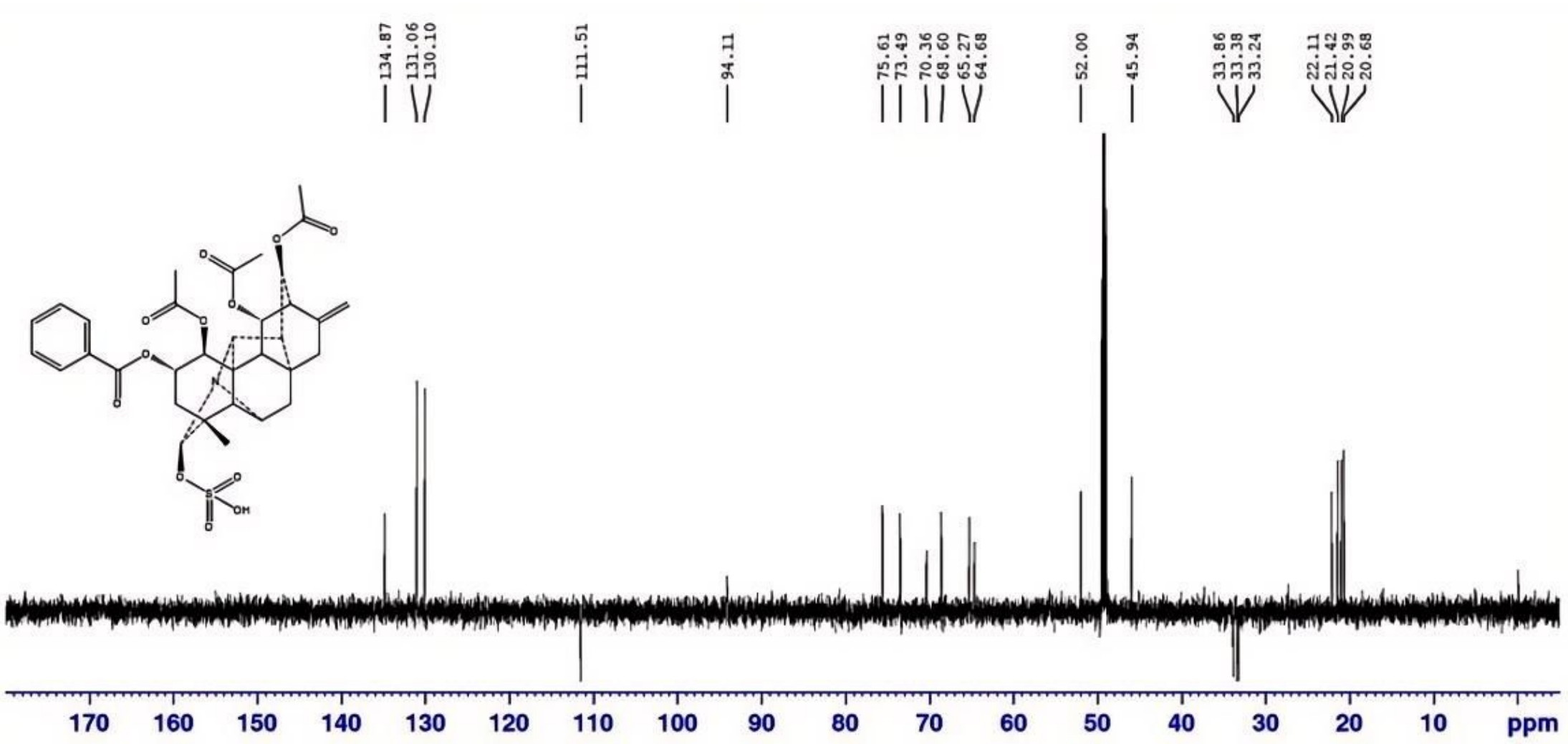


**Figure S13.** DEPT NMR spectrum of **2** in methanol-$d_4$

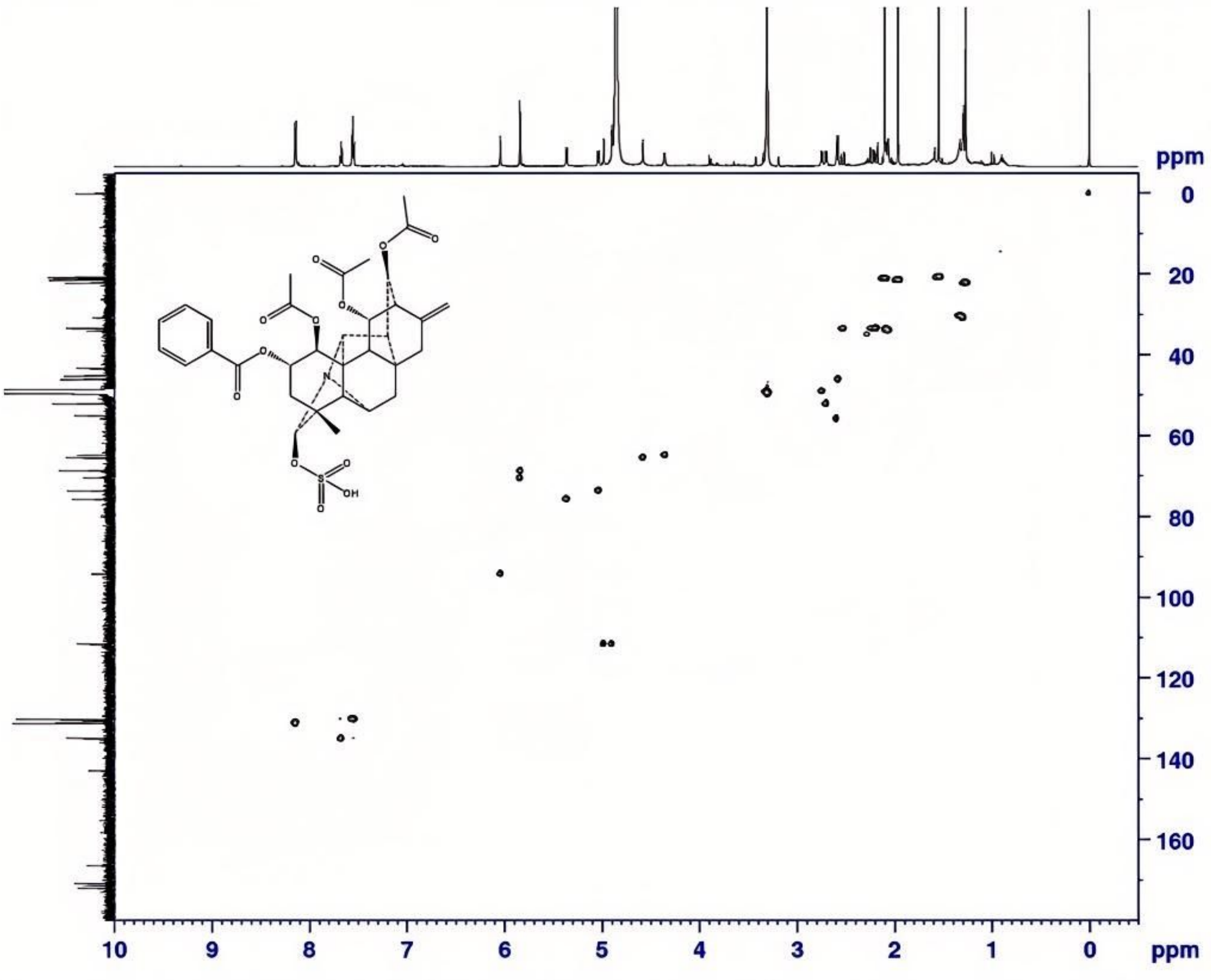


**Figure S14.** HSQC spectrum of **2**

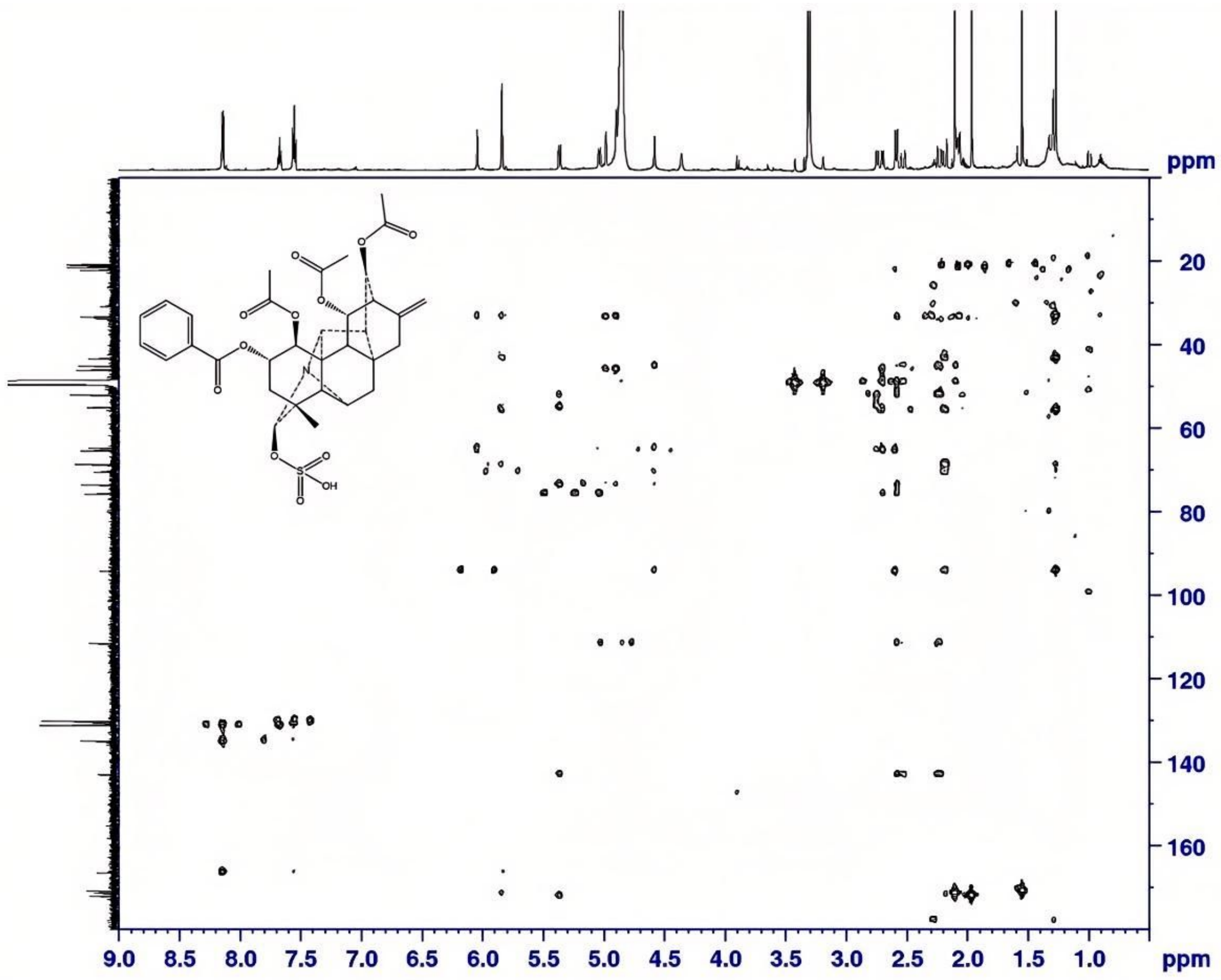


**Figure S15.** HMBC spectrum of **2**

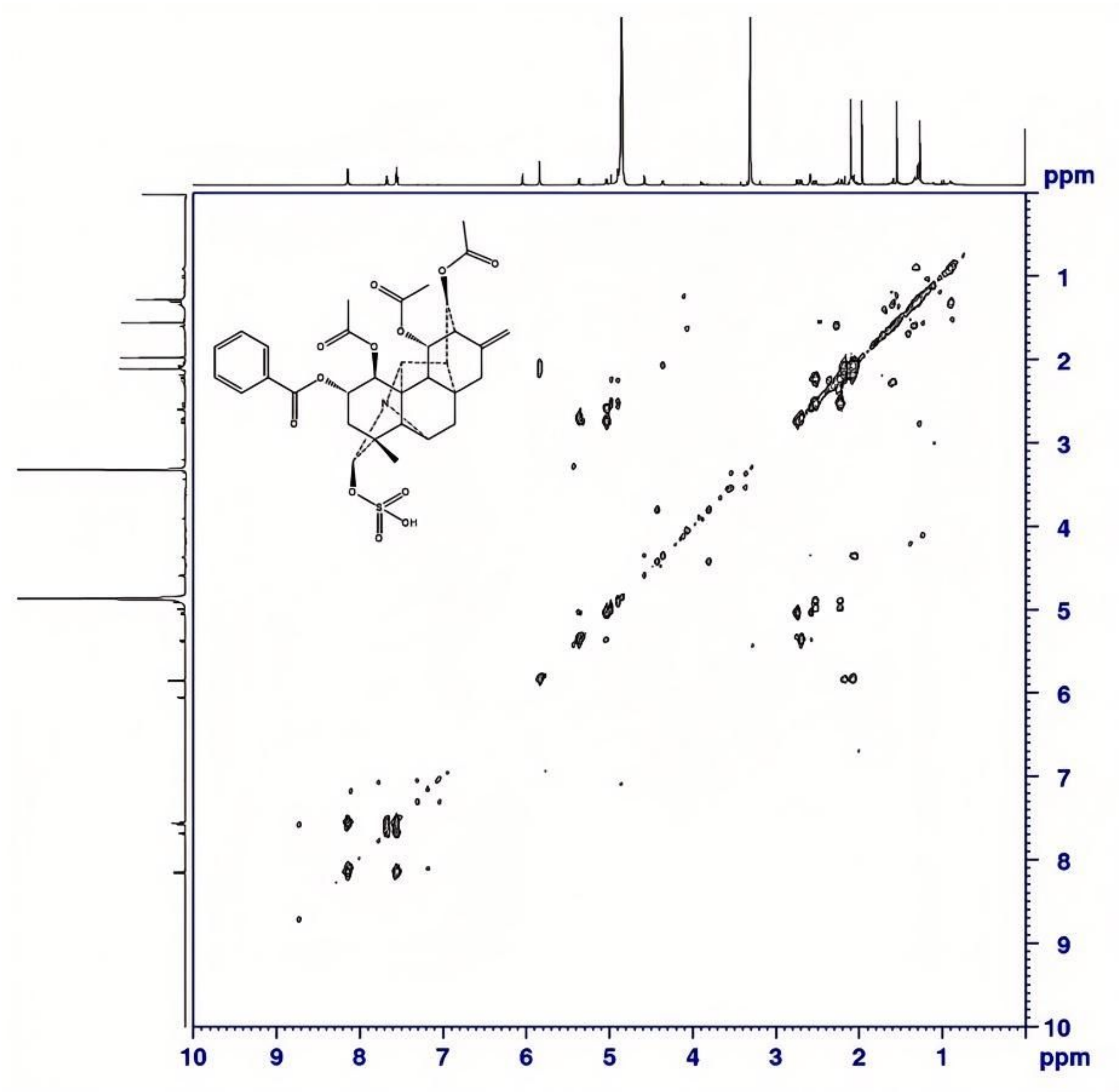


**Figure S16.** $^{1}H$-$^{1}H$ COSY spectrum of **2**

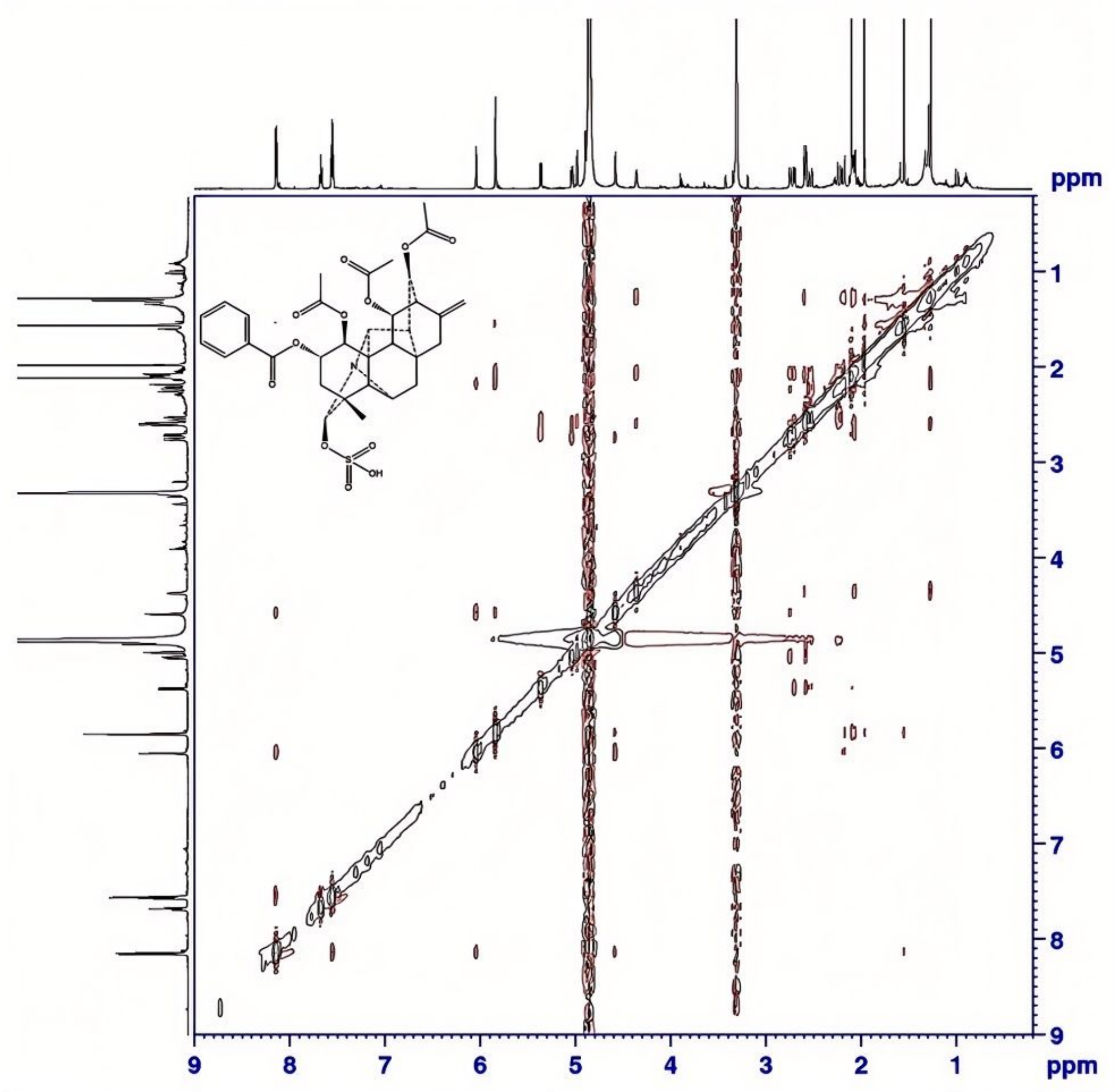

**Figure S17.** NOESY spectrum of **2**

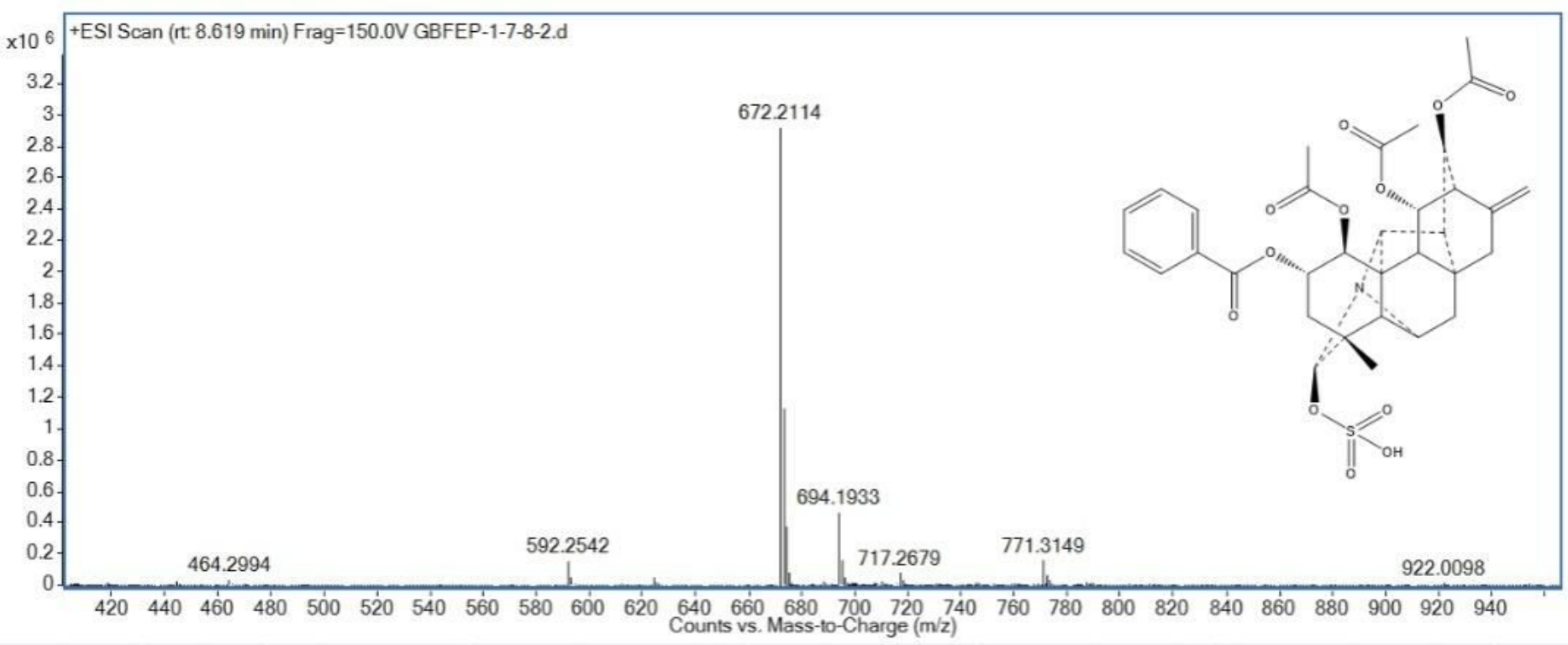


| Formula | Score | Mass | Mass (MFG) | m/z (Calc) | Diff (ppm) | Ion Formula | m/z |
|---|---|---|---|---|---|---|---|
| $C_{33}H_{37}NO_{12}S$ | 99.67 | 671.2041 | 671.2036 | 672.2109 | -0.71 | $C_{33}H_{38}NO_{12}S$ | 672.2114 |
| $C_{33}H_{37}NO_{12}S$ | 99.73 | 671.2041 | 671.2036 | 694.1929 | -0.64 | $C_{33}H_{37}NO_{12}SNa$ | 694.1933 |

**Figure S18.** HRESIMS spectrum of **2**

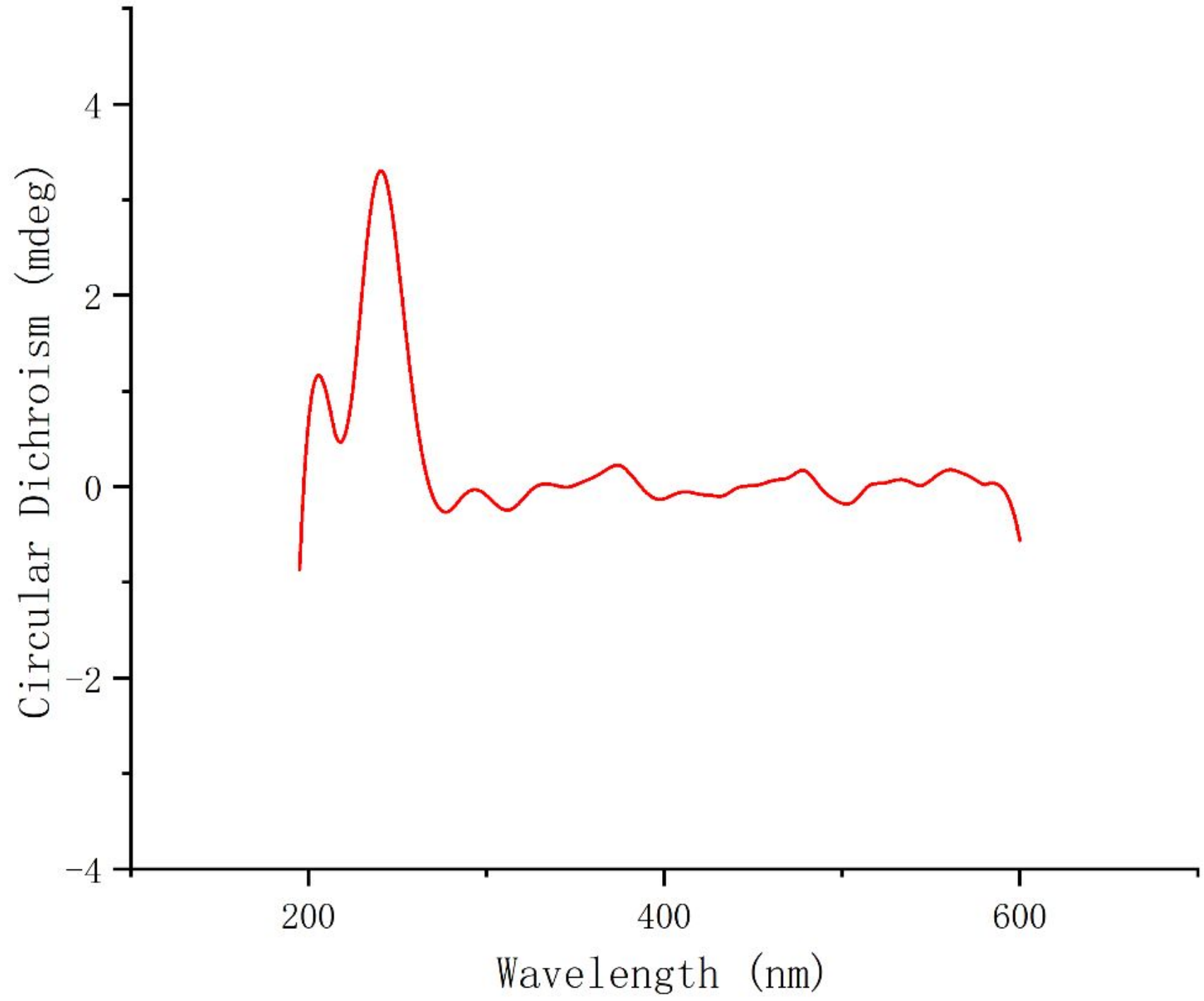


**Figure S19.** ECD spectrum of **2**

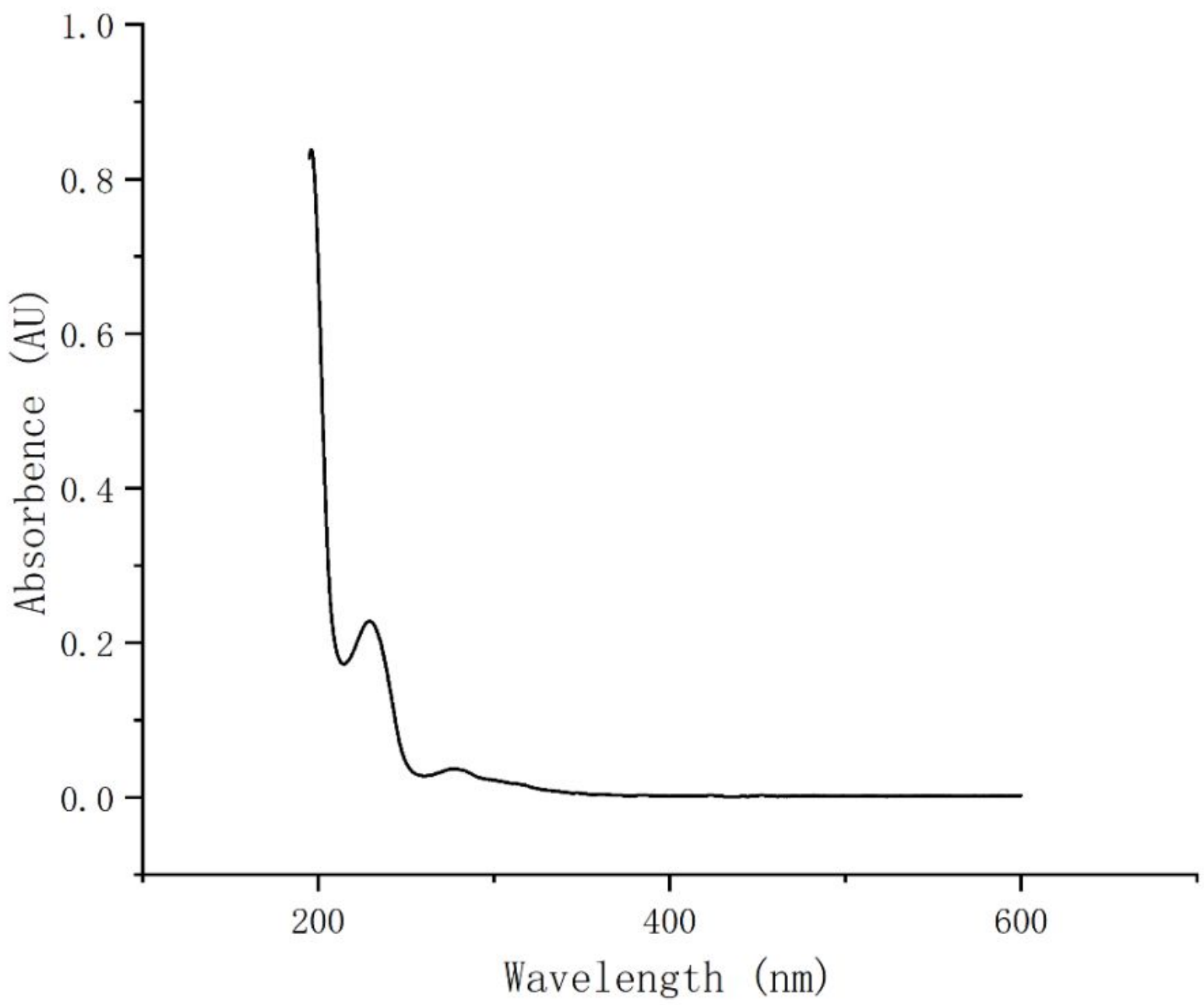


**Figure S20.** UV spectrum of **2**

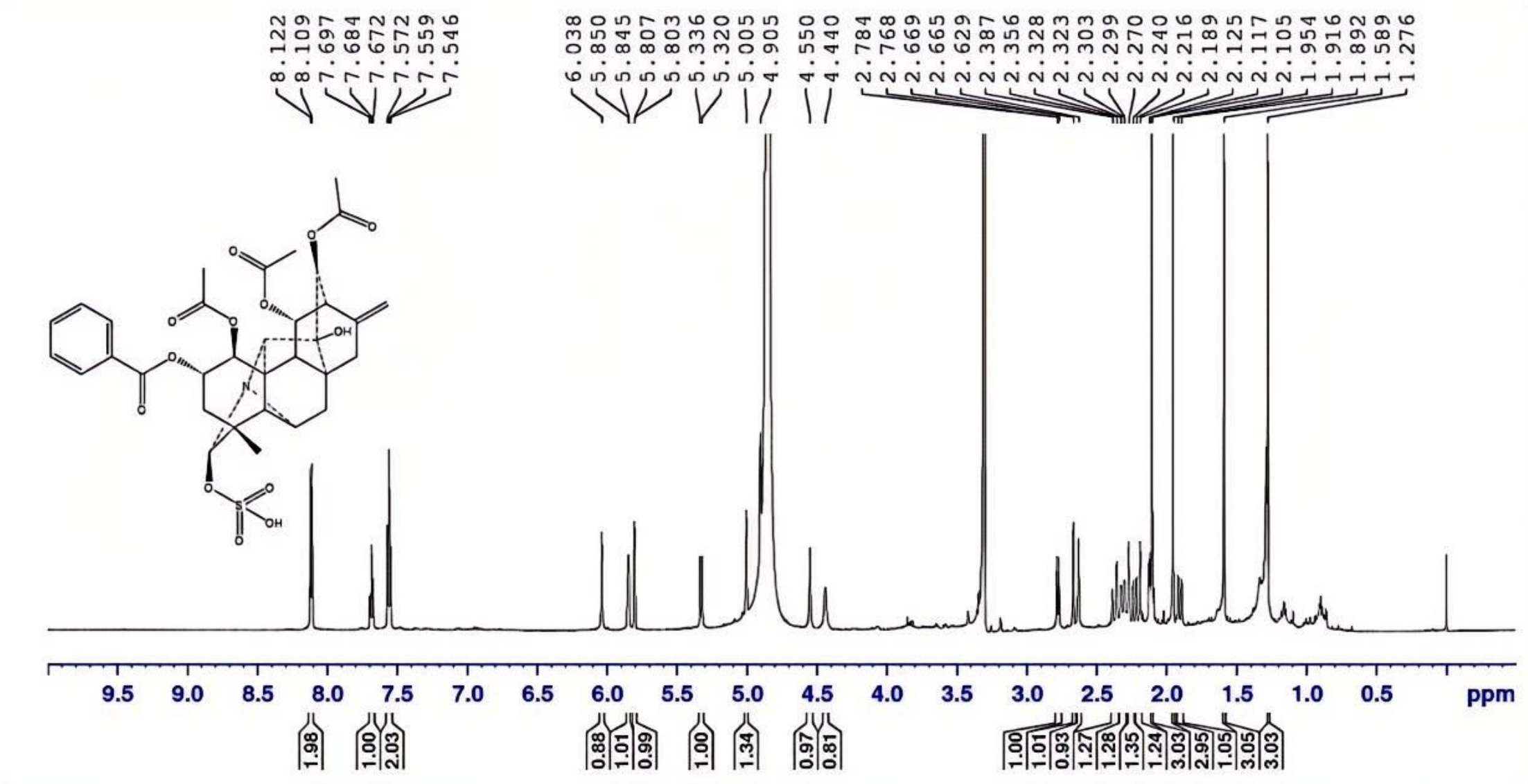


**Figure S21.** $^{1}$H NMR spectrum of **3** in methanol-$d_4$

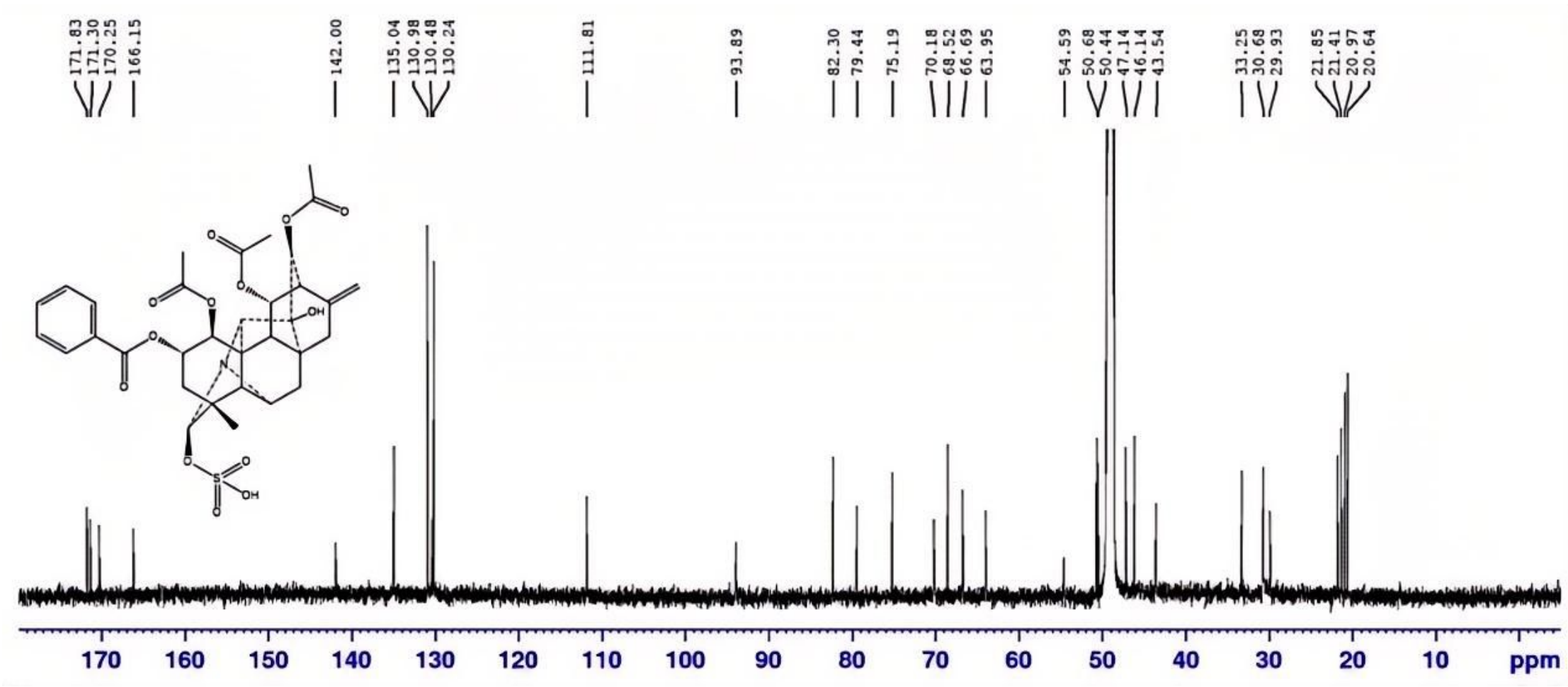


**Figure S22.** $^{13}$C NMR spectrum of **3** in methanol-$d_4$

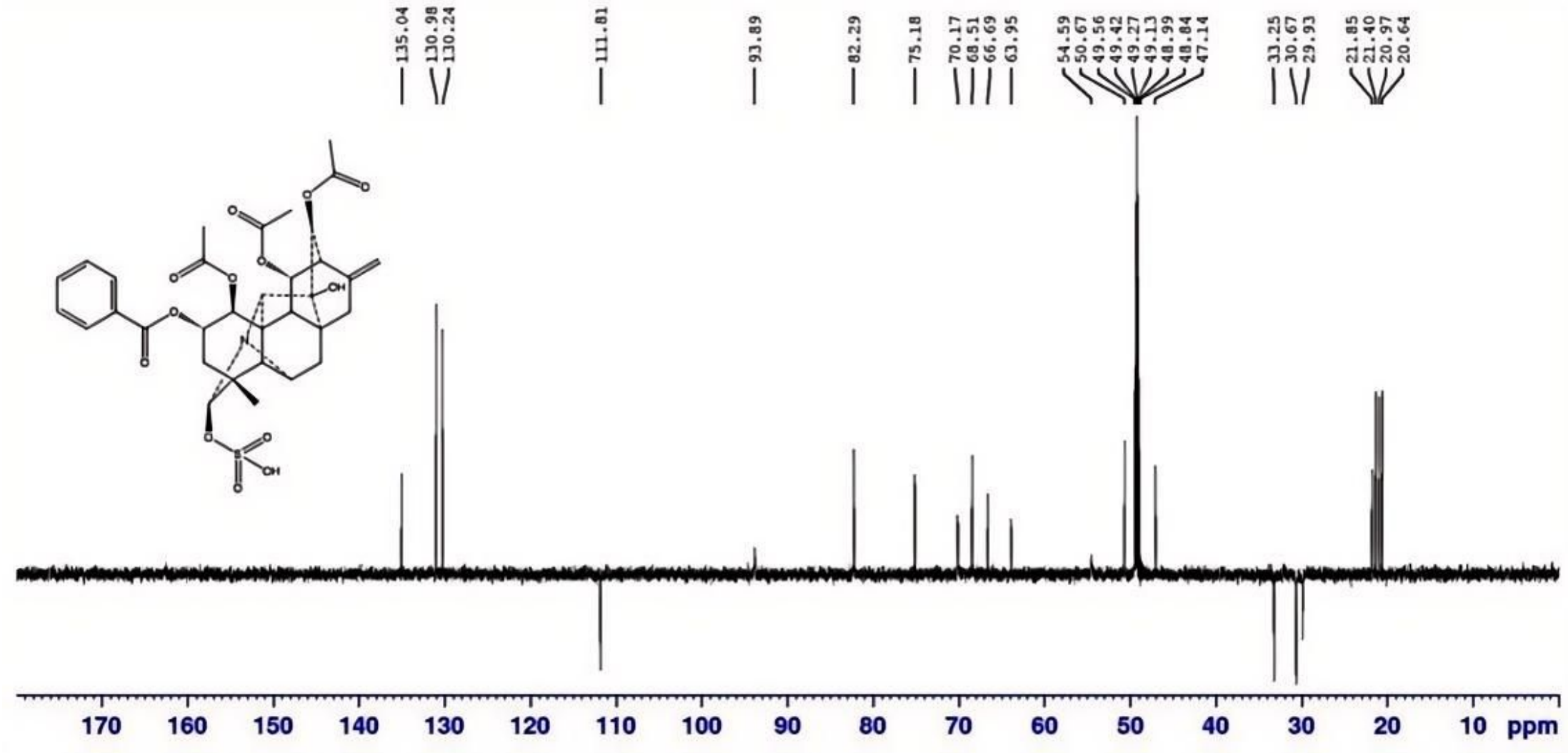

**Figure S23.** DEPT NMR spectrum of **3** in methanol-$d_4$

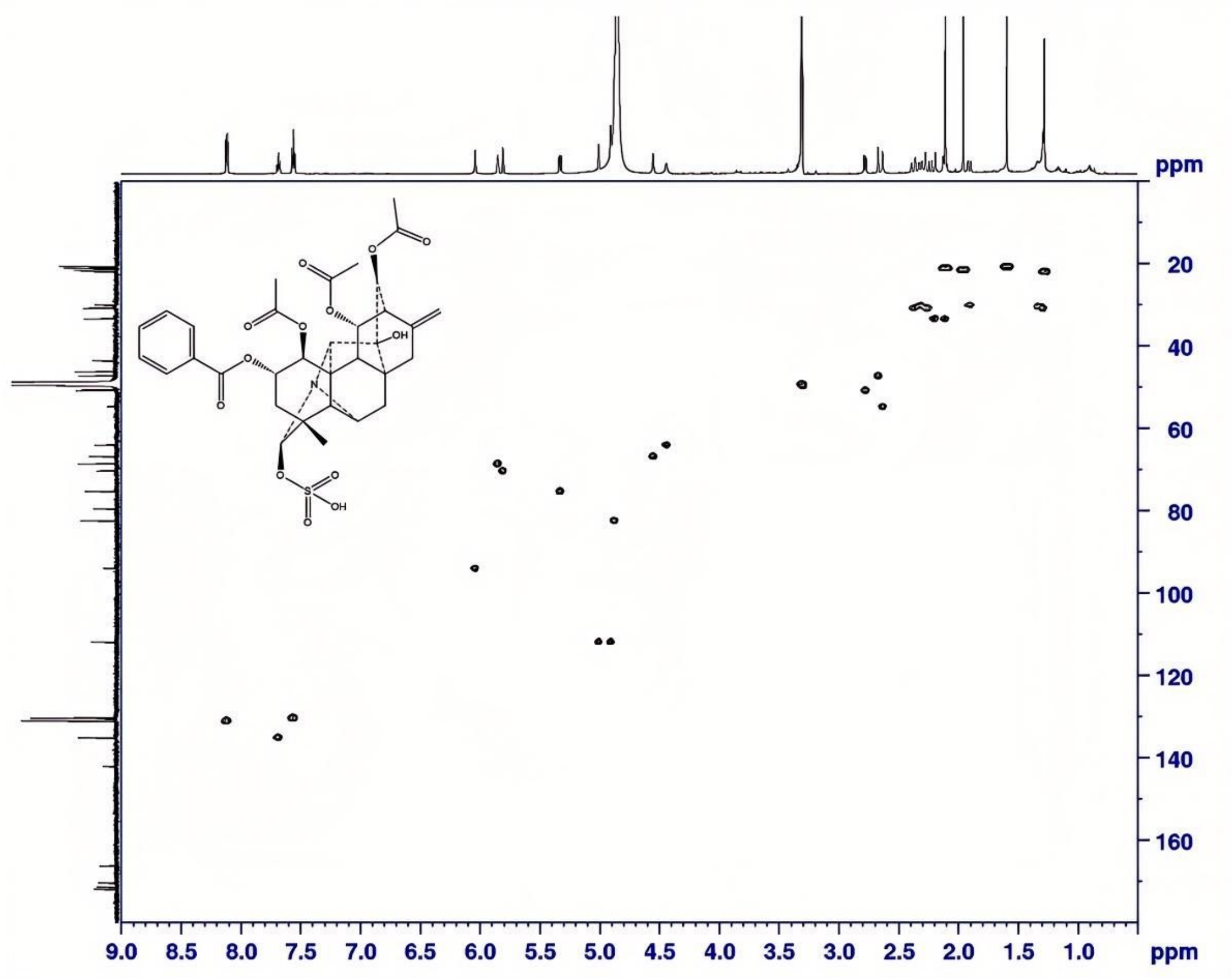


**Figure S24.** HSQC spectrum of **3**

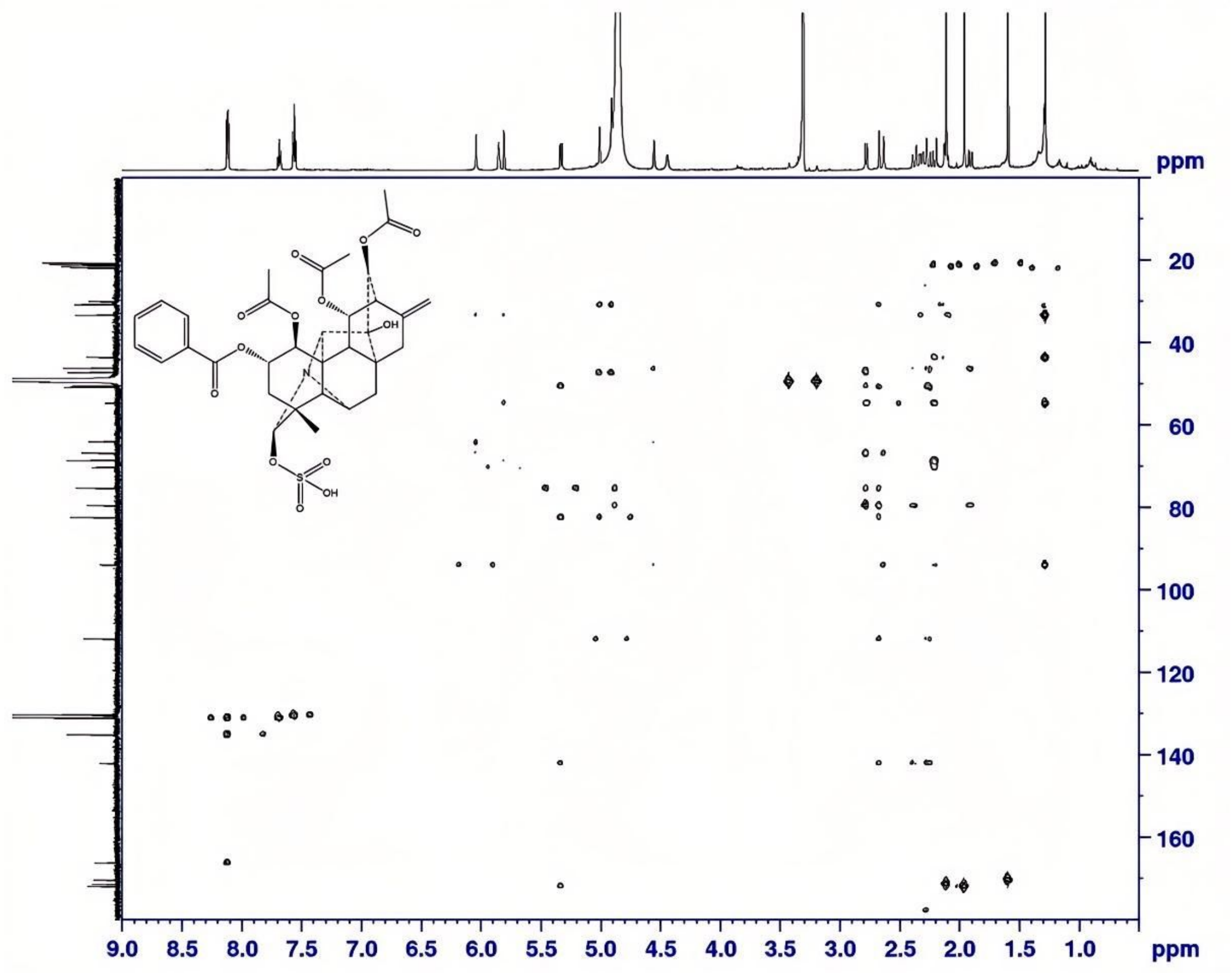


**Figure S25.** HMBC spectrum of **3**

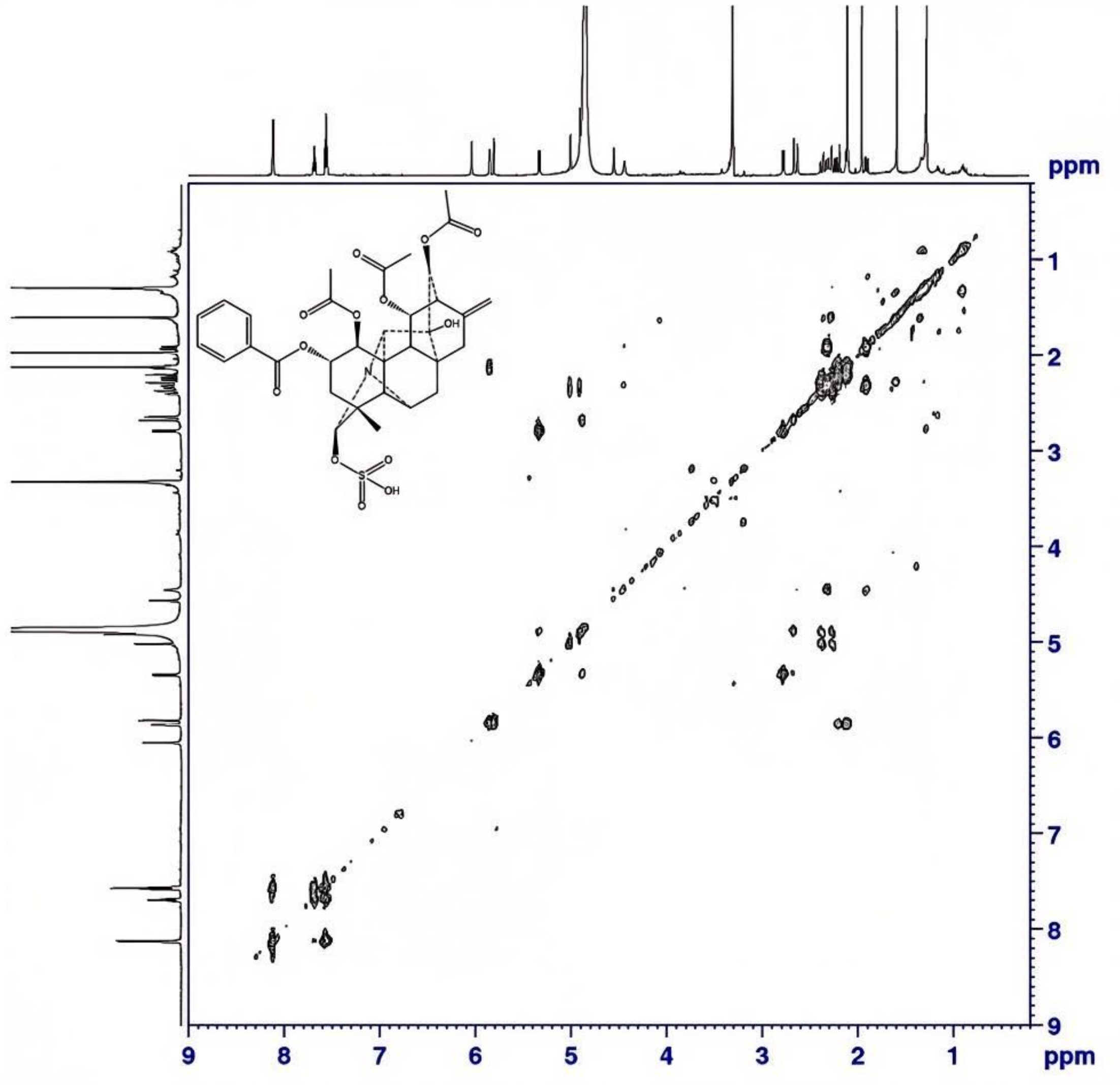


**Figure S26.** $^1H$-$^1H$ COSY spectrum of **3**

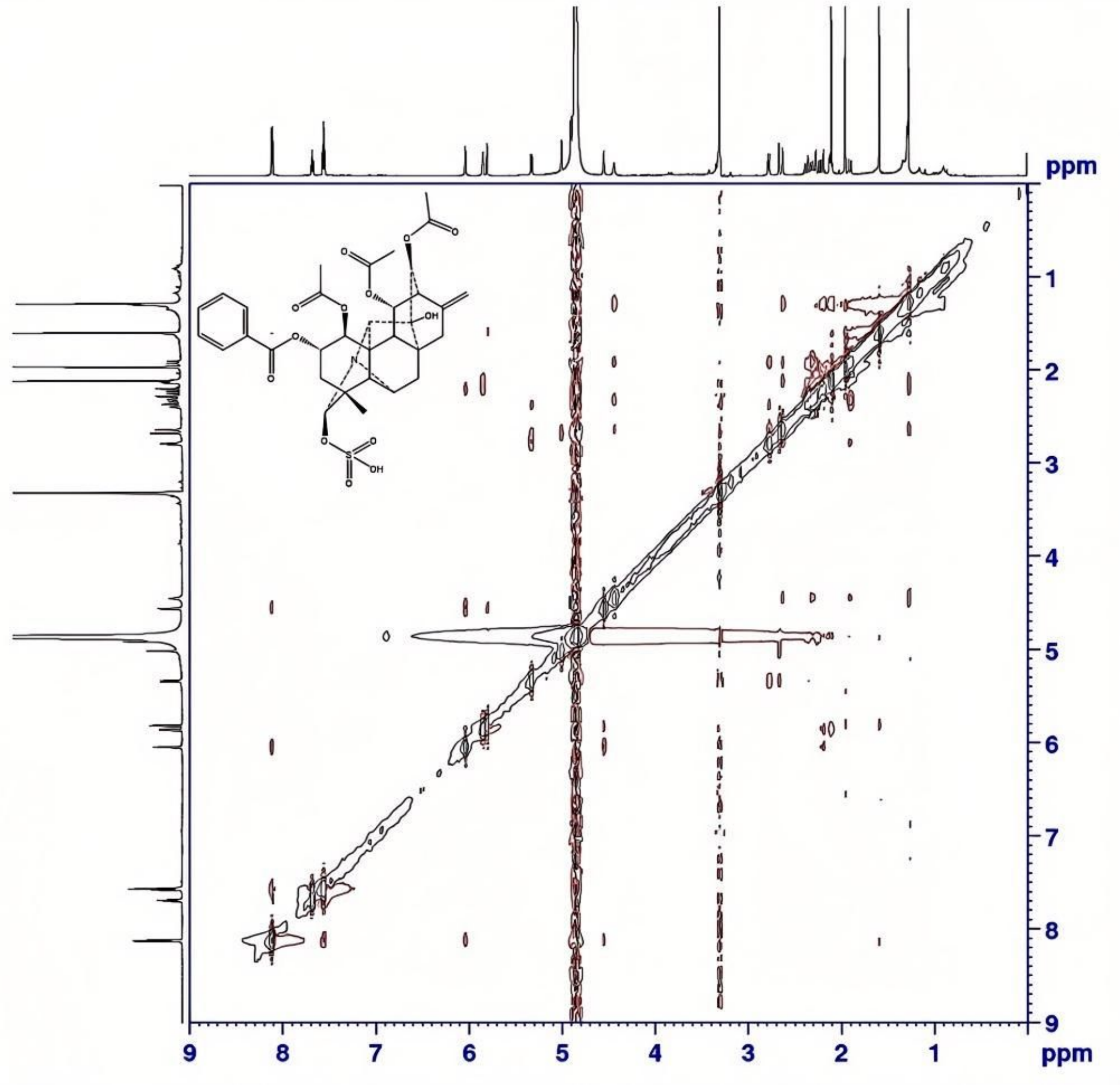

**Figure S27.** NOESY spectrum of **3**

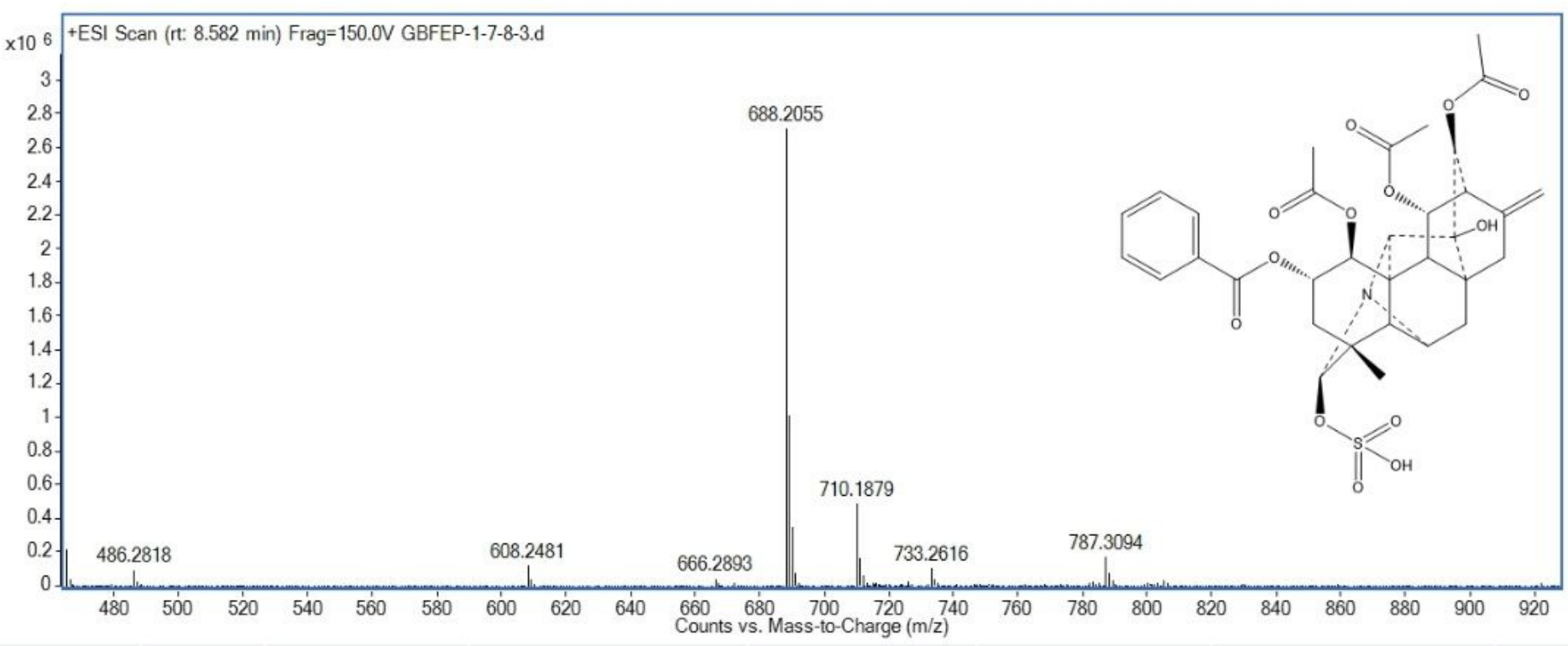

| Formula | Score | Mass | Mass (MFG) | m/z (Calc) | Diff (ppm) | Ion Formula | m/z |
|---|---|---|---|---|---|---|---|
| $C_{33}H_{37}NO_{13}S$ | 99.84 | 687.1982 | 687.1986 | 688.2058 | 0.49 | $C_{33}H_{38}NO_{13}S$ | 688.2055 |
| $C_{33}H_{37}NO_{13}S$ | 99.98 | 687.1987 | 687.1986 | 710.1878 | -0.17 | $C_{33}H_{37}NO_{13}SNa$ | 710.1879 |

**Figure S28.** HRESIMS spectrum of **3**

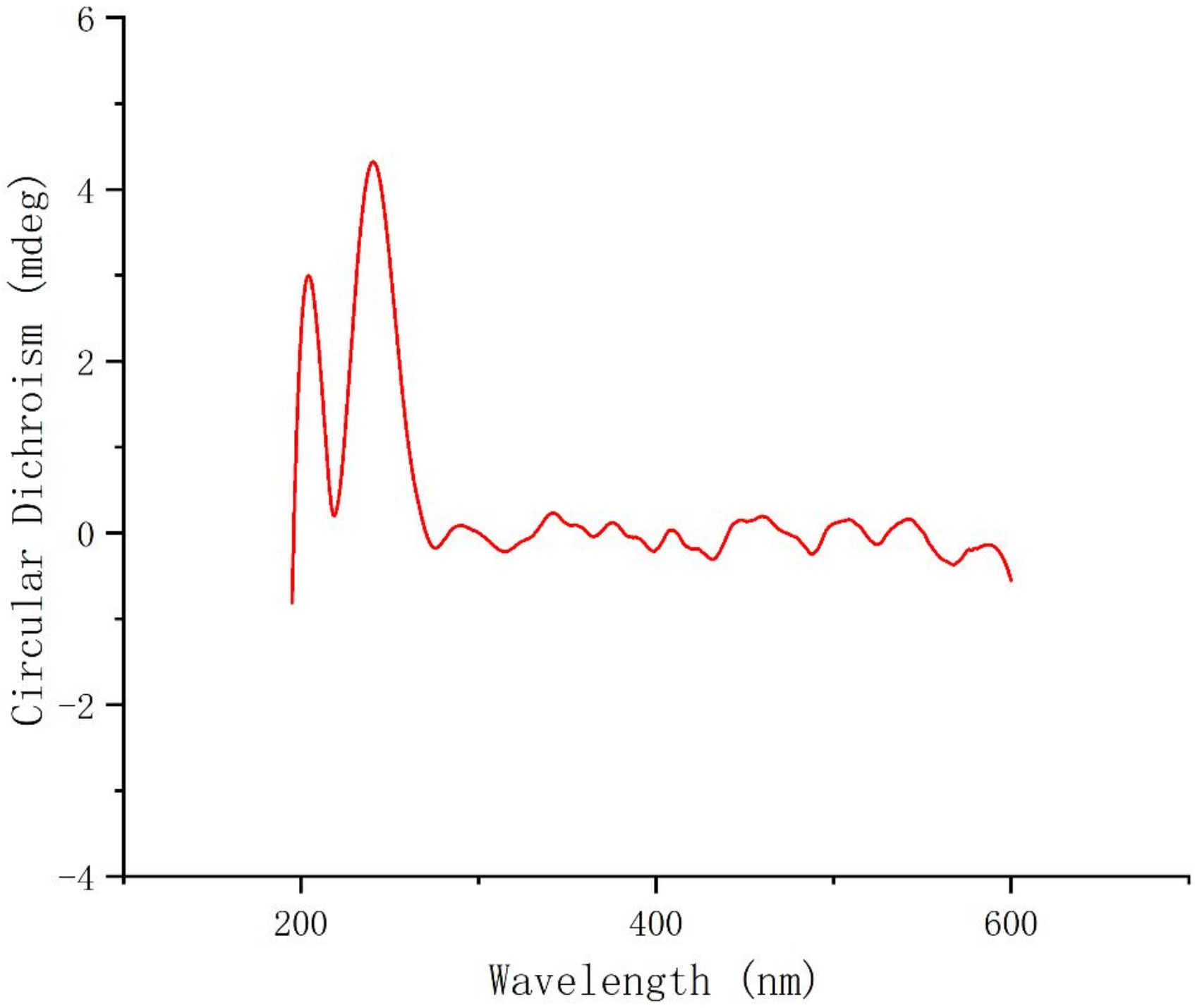


**Figure S29.** ECD spectrum of **3**

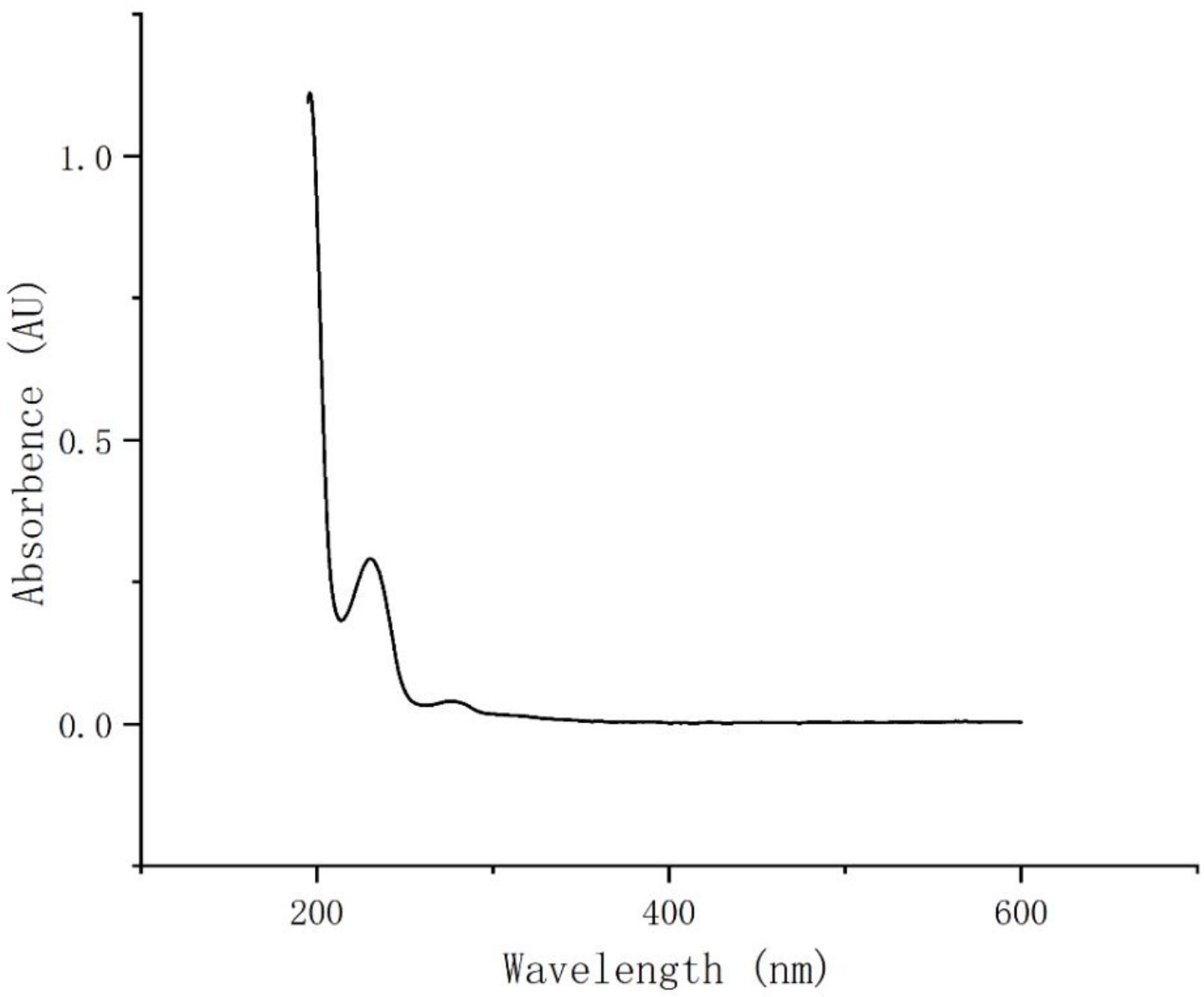

**Figure S30.** UV spectrum of **3**

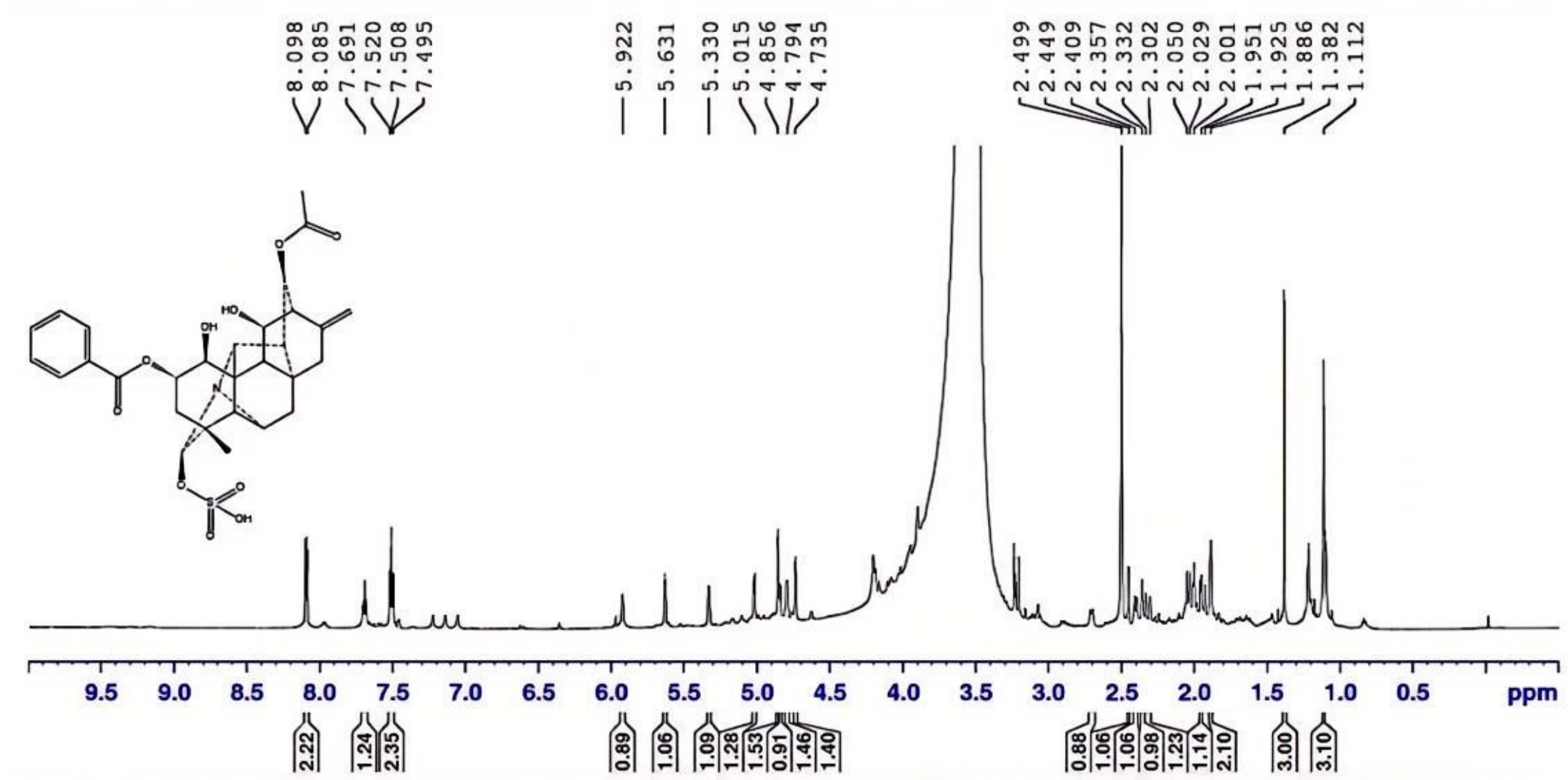


**Figure S31.** $^{1}$H NMR spectrum of **4** in DMSO-$d_6$

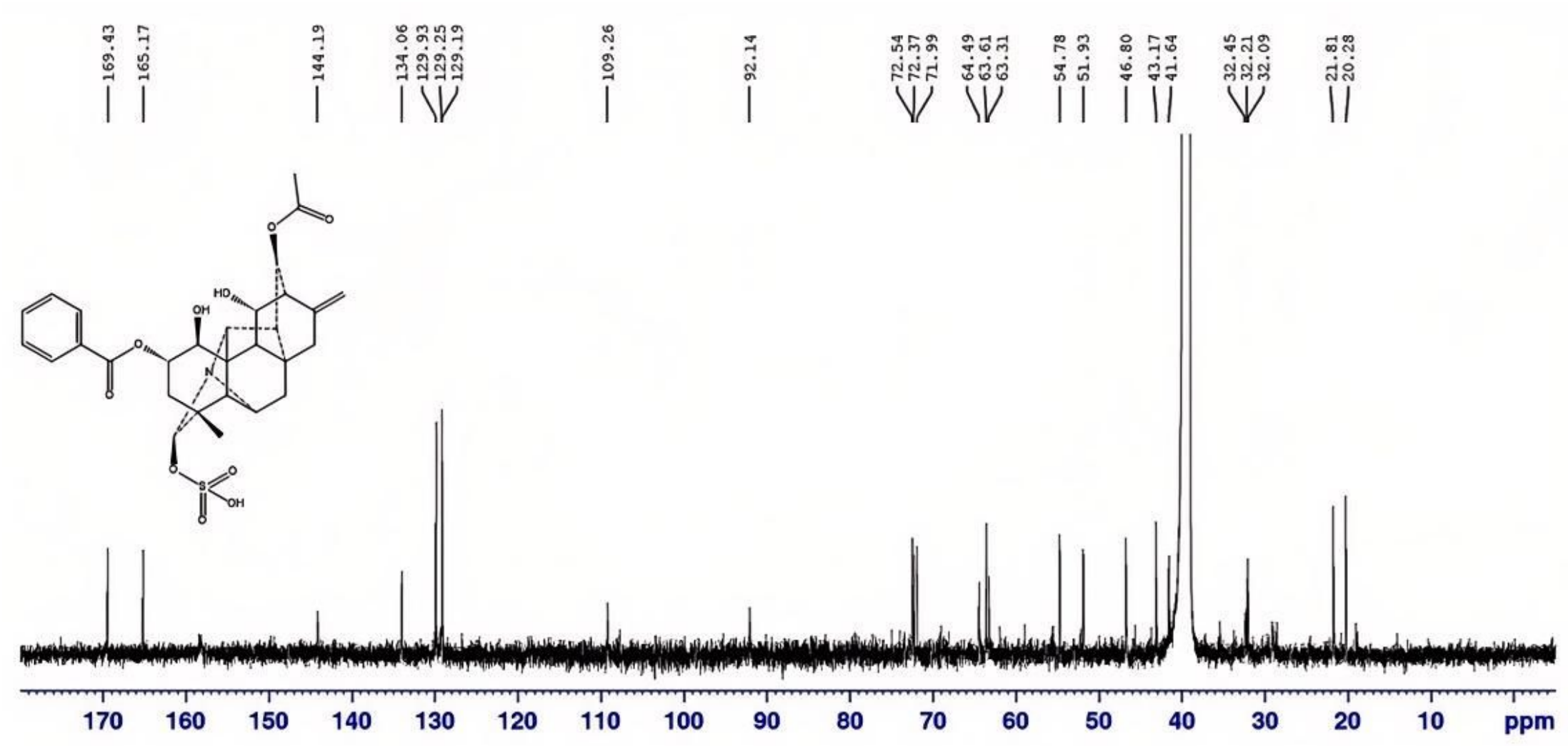


**Figure S32.** $^{13}$C NMR spectrum of **4** in DMSO-$d_6$

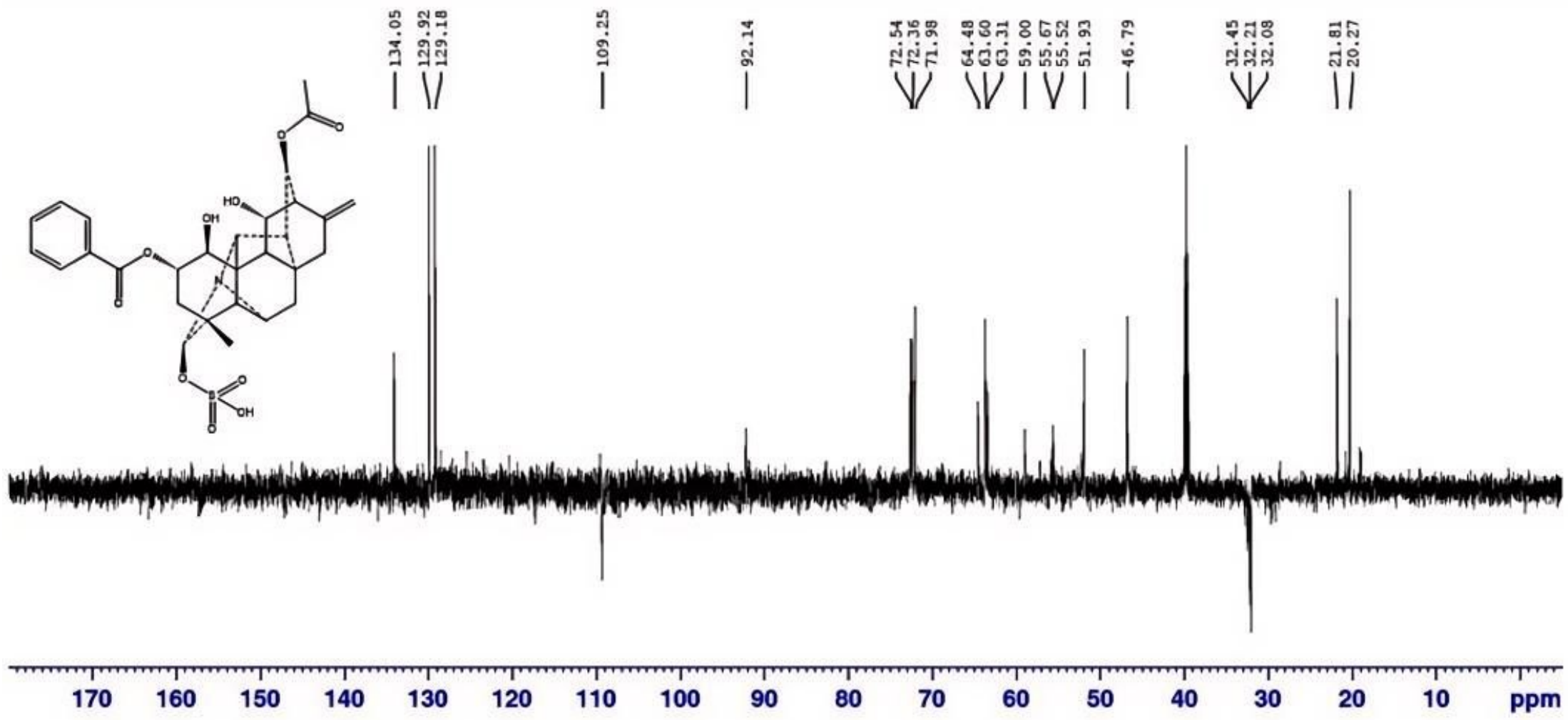


**Figure S33.** DEPT NMR spectrum of **4** in DMSO-$d_6$

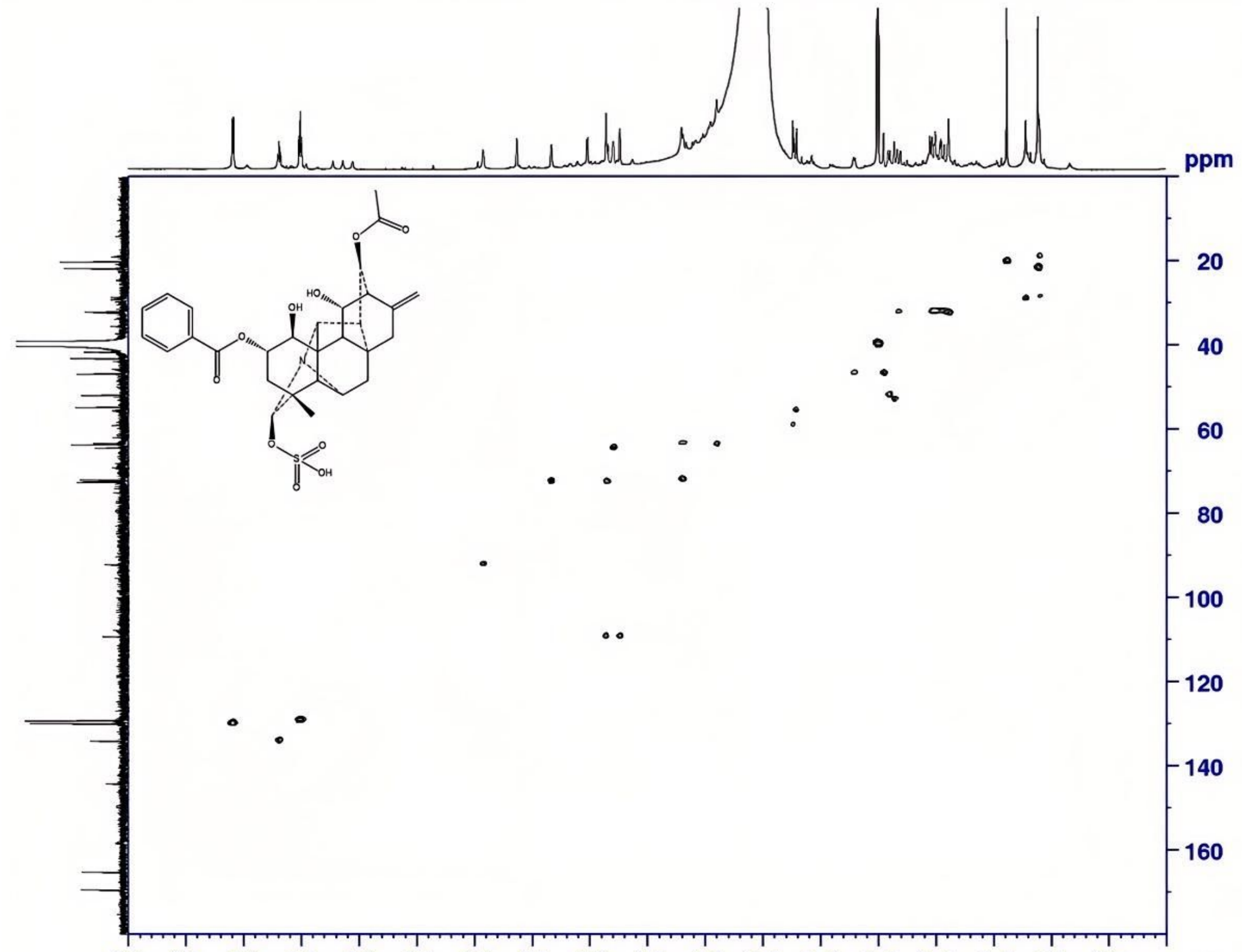


**Figure S34.** HSQC spectrum of **4**

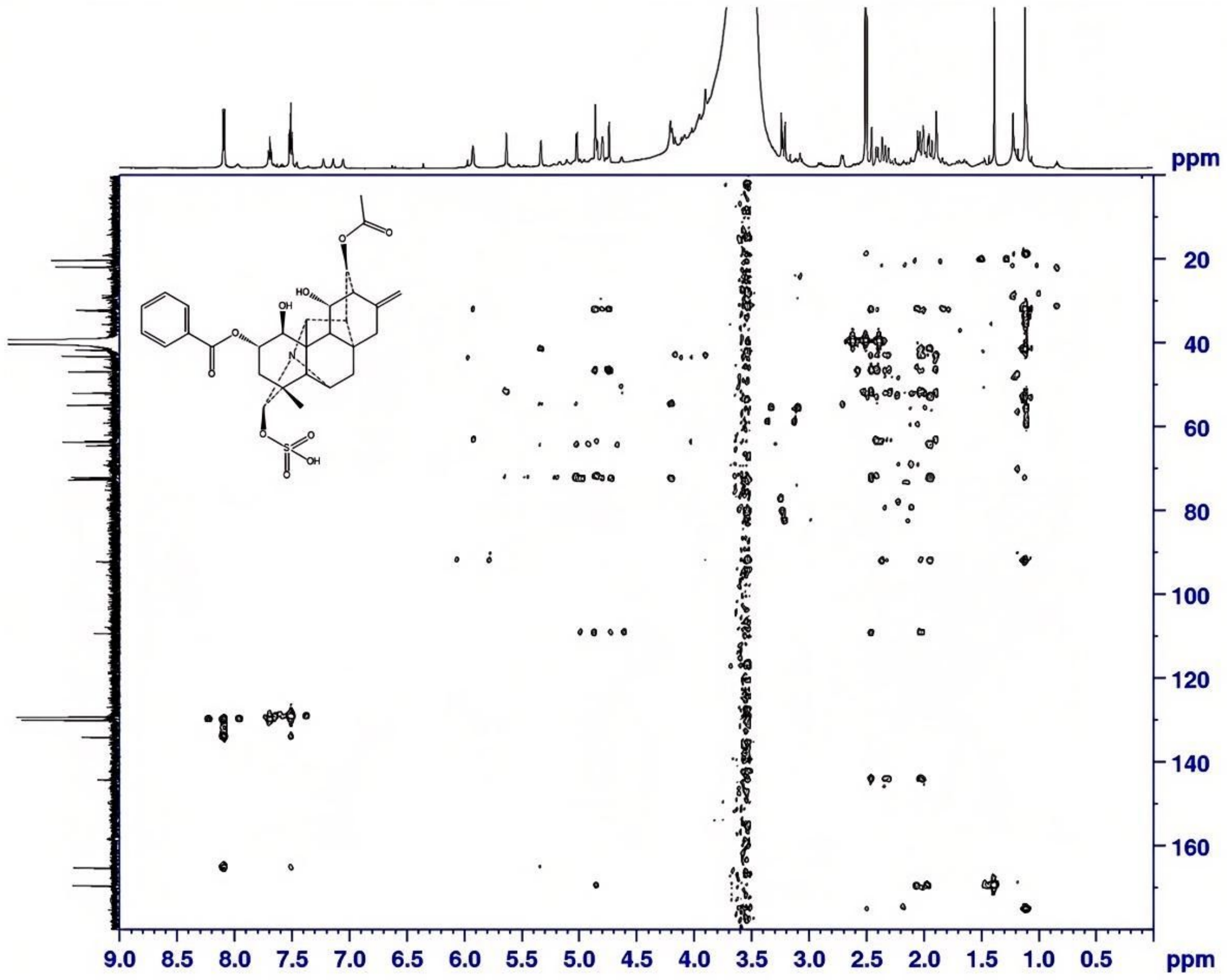


**Figure S35.** HMBC spectrum of **4**

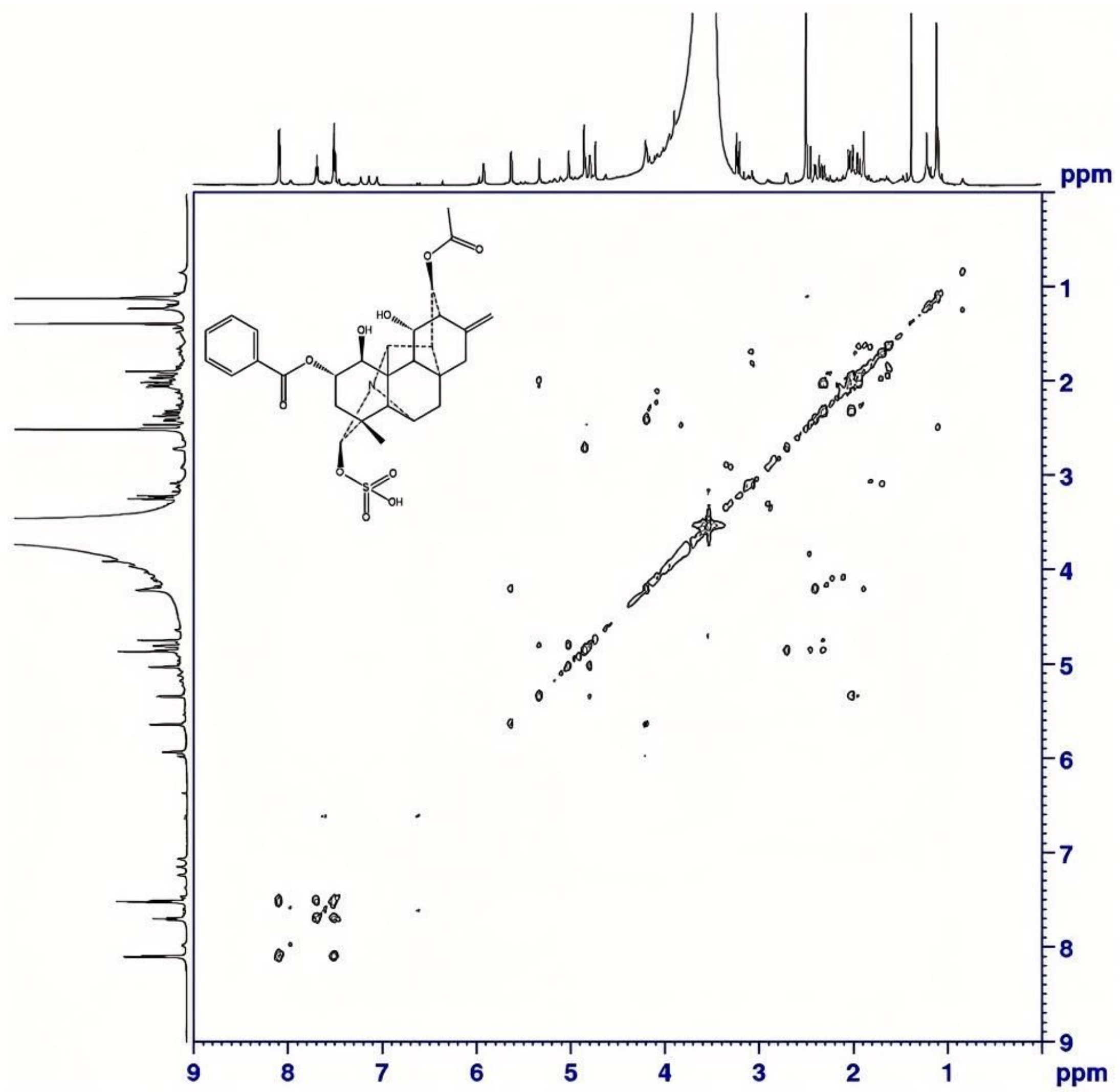


**Figure S36.** $^{1}H$-$^{1}H$ COSY spectrum of **4**

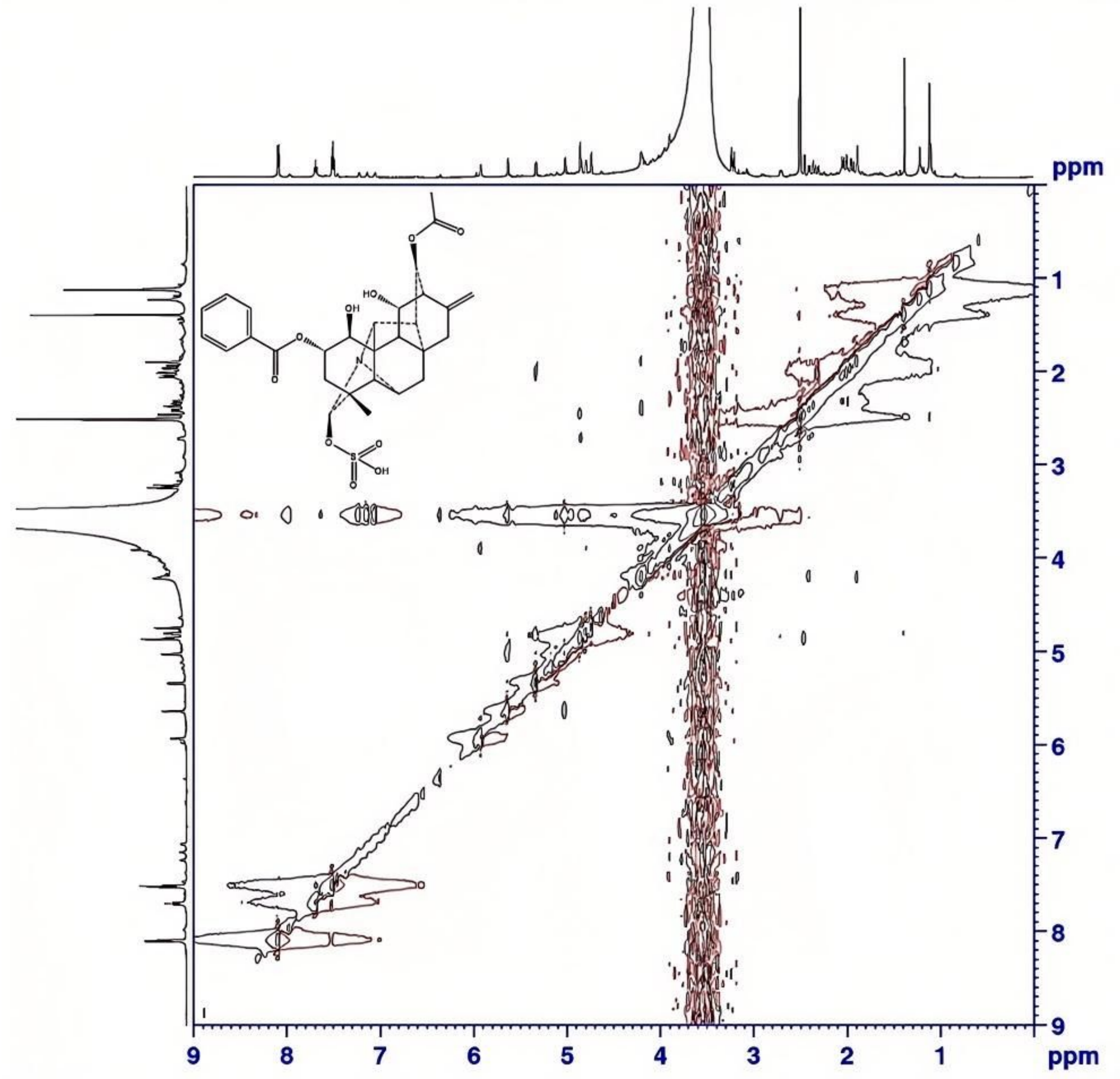


**Figure S37.** NOESY spectrum of **4**

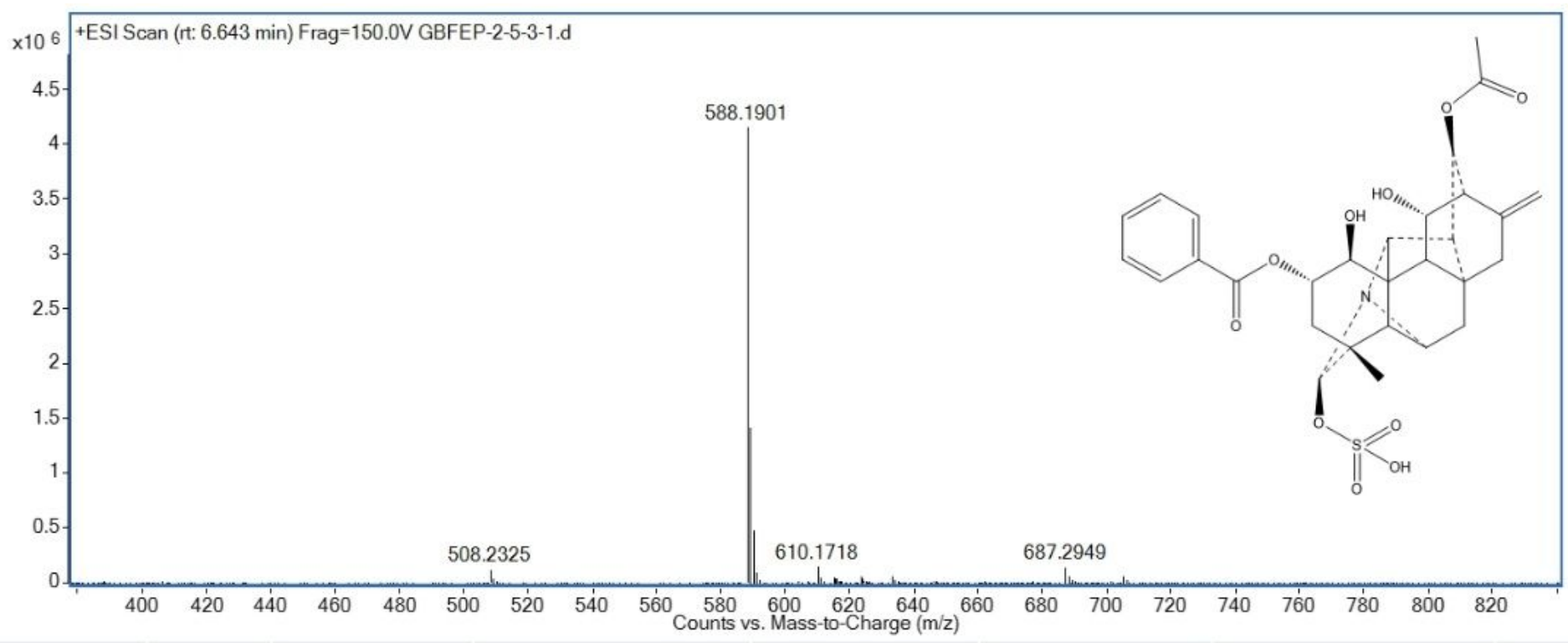


| Formula | Score | Mass | Mass (MFG) | m/z (Calc) | Diff (ppm) | Ion Formula | m/z |
|---|---|---|---|---|---|---|---|
| $C_{29}H_{33}NO_{10}S$ | 99.84 | 587.1828 | 587.1825 | 588.1898 | -0.52 | $C_{29}H_{34}NO_{10}S$ | 588.1901 |
| $C_{29}H_{33}NO_{10}S$ | 99.99 | 587.1826 | 587.1825 | 610.1717 | -0.11 | $C_{29}H_{33}NO_{10}SNa$ | 610.1718 |

**Figure S38.** HRESIMS spectrum of **4**

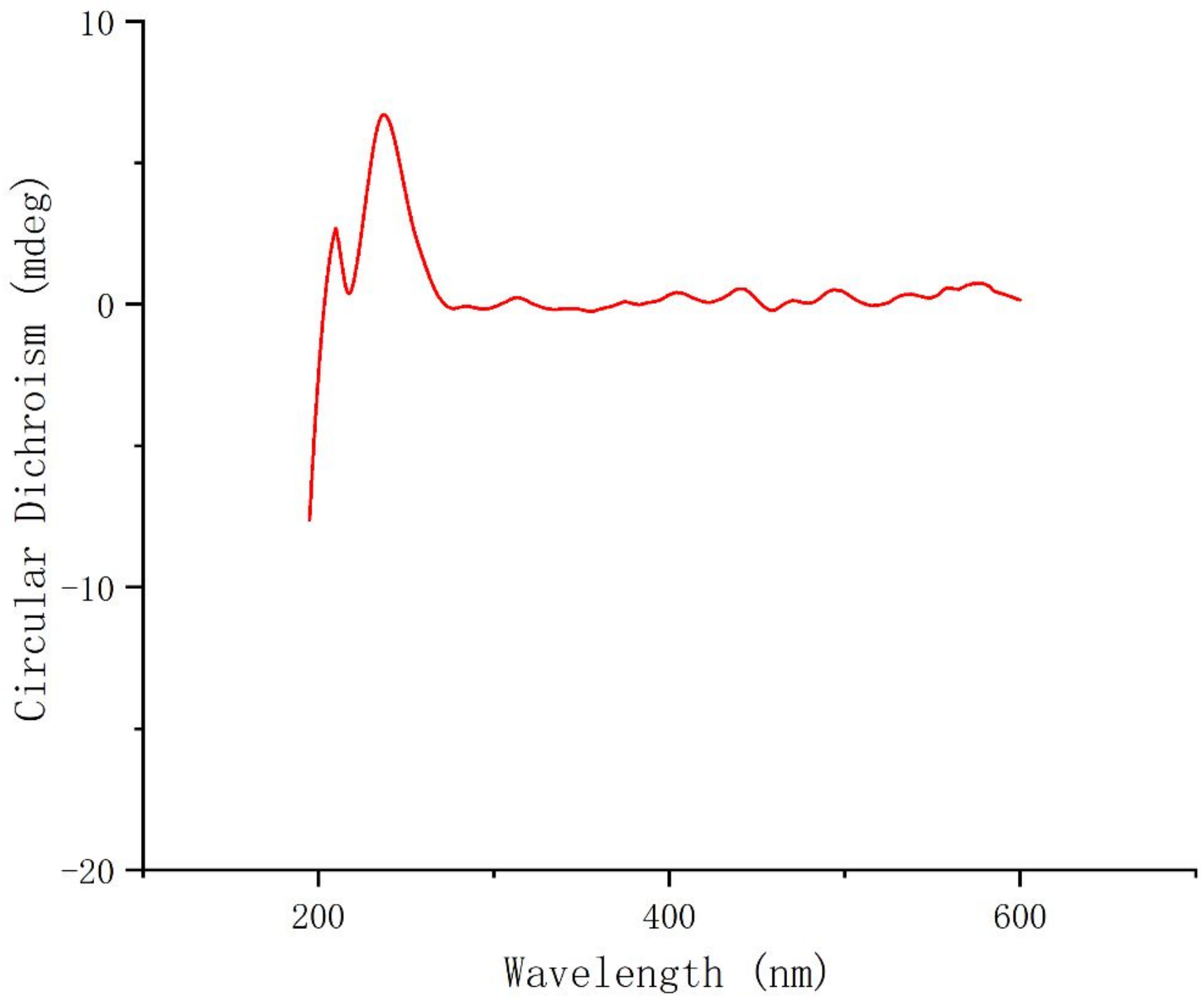


**Figure S39.** ECD spectrum of **4**

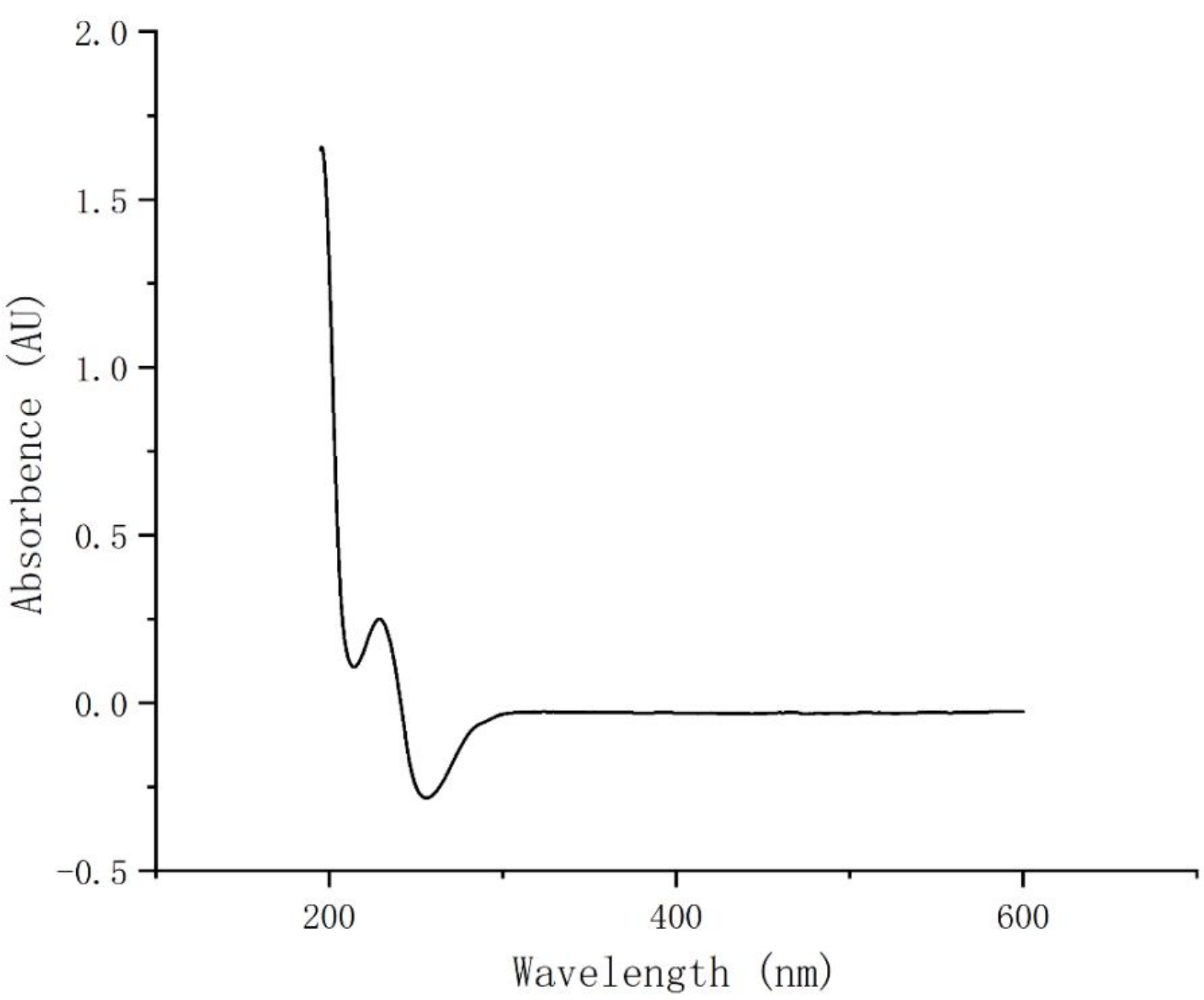


**Figure S40.** UV spectrum of **4**

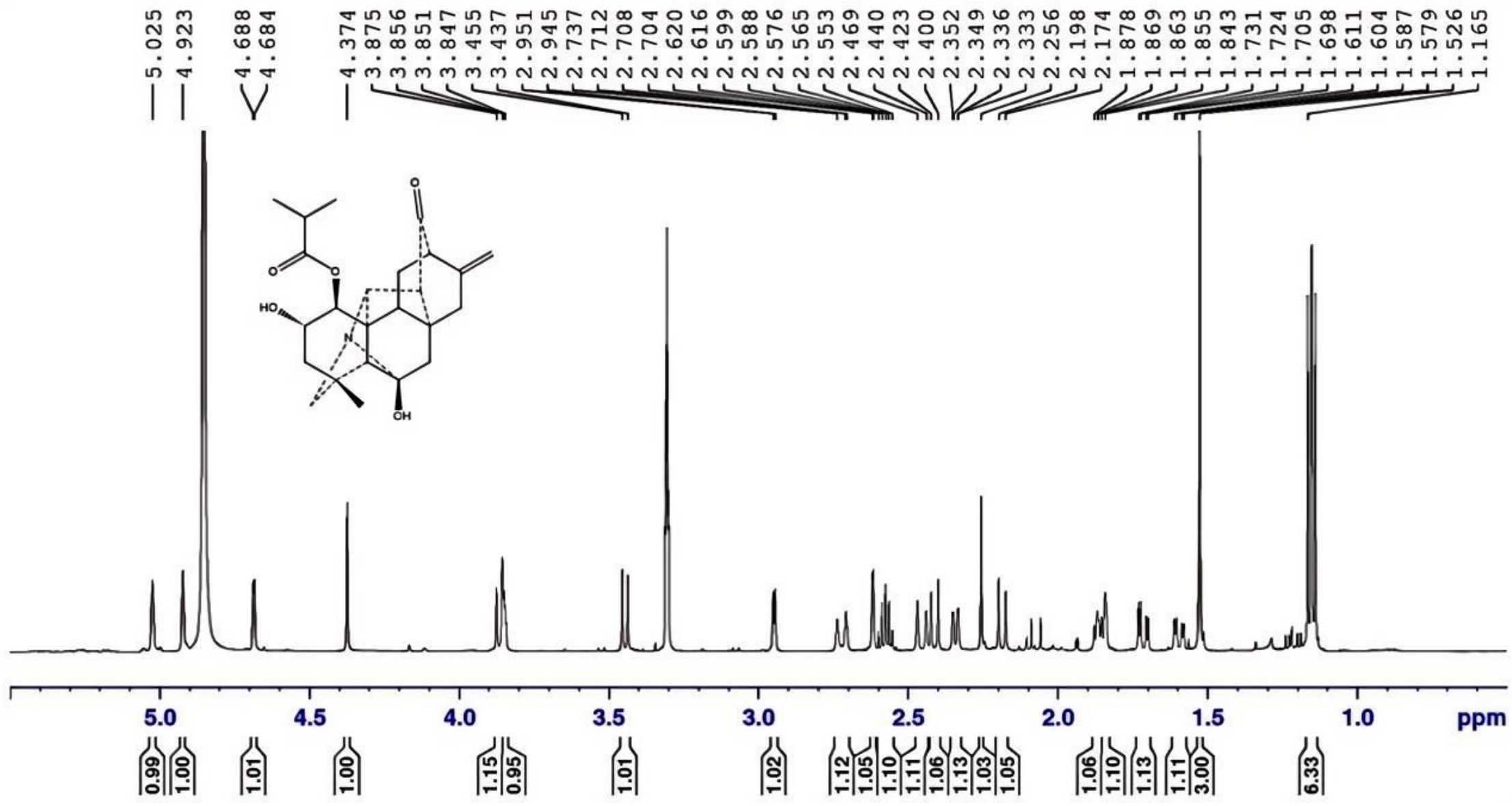


**Figure S41.** $^{1}$H NMR spectrum of **5** in methanol-$d_4$

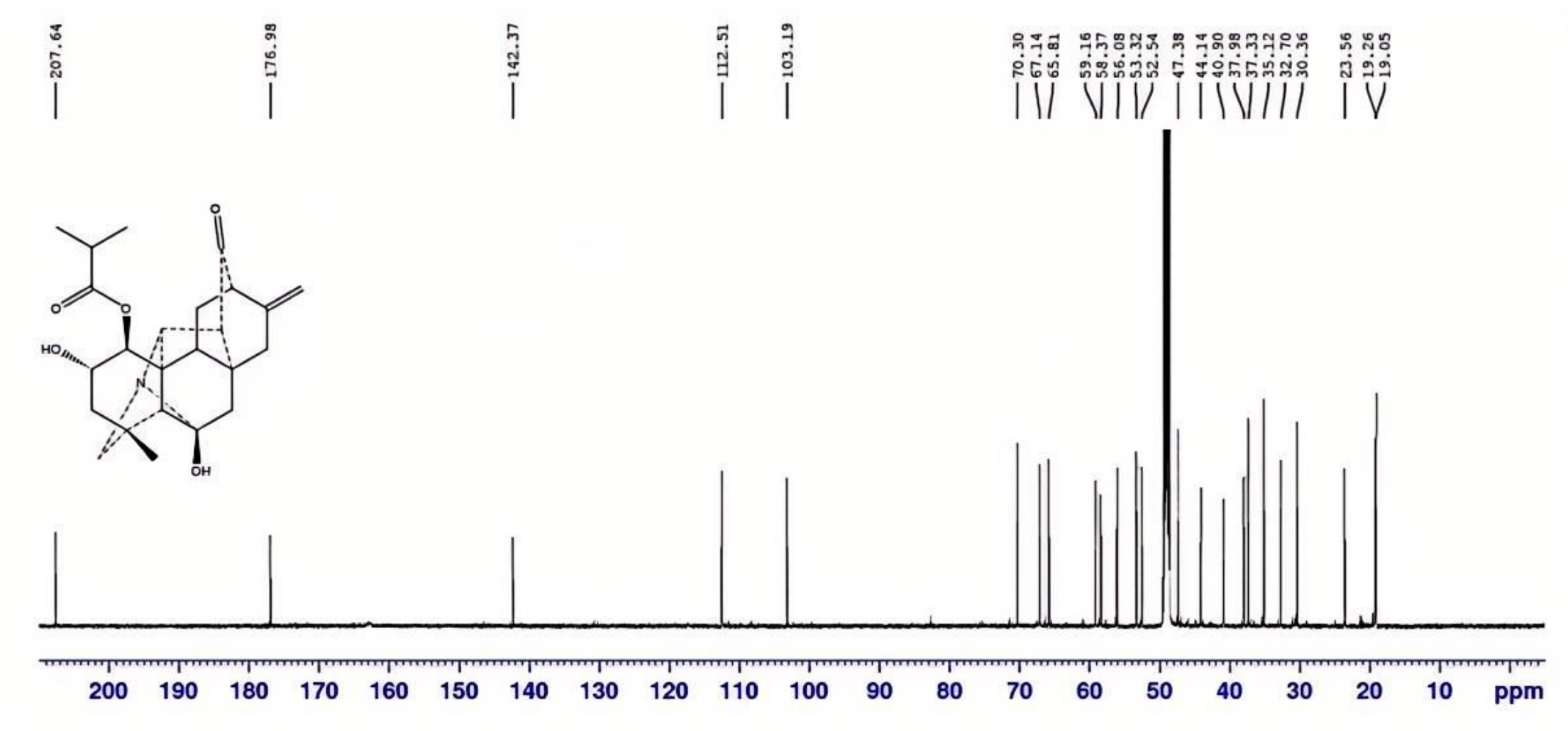


**Figure S42.** $^{13}$C NMR spectrum of **5** in methanol-$d_4$

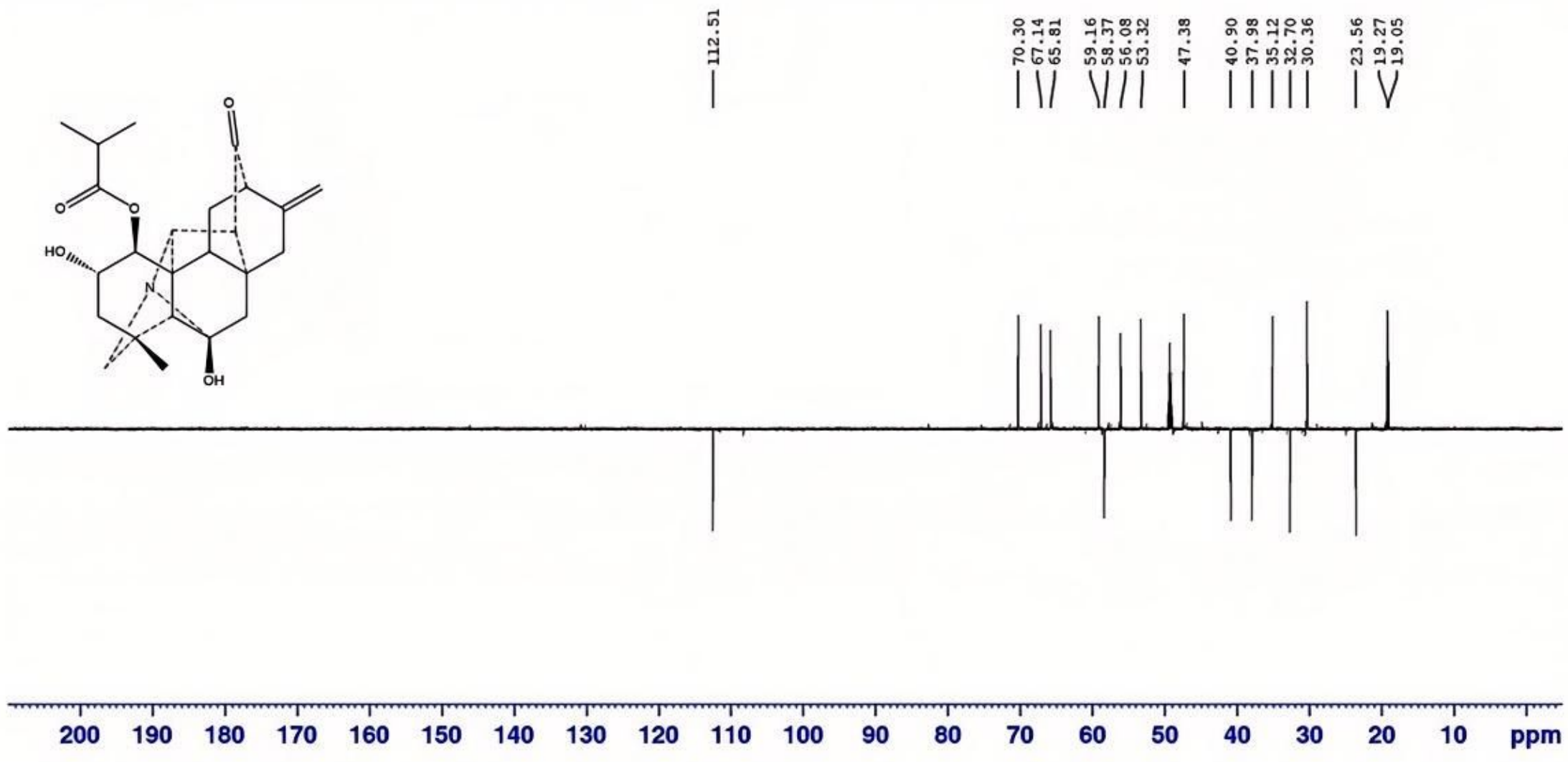


**Figure S43.** DEPT NMR spectrum of **5** in methanol-$d_4$

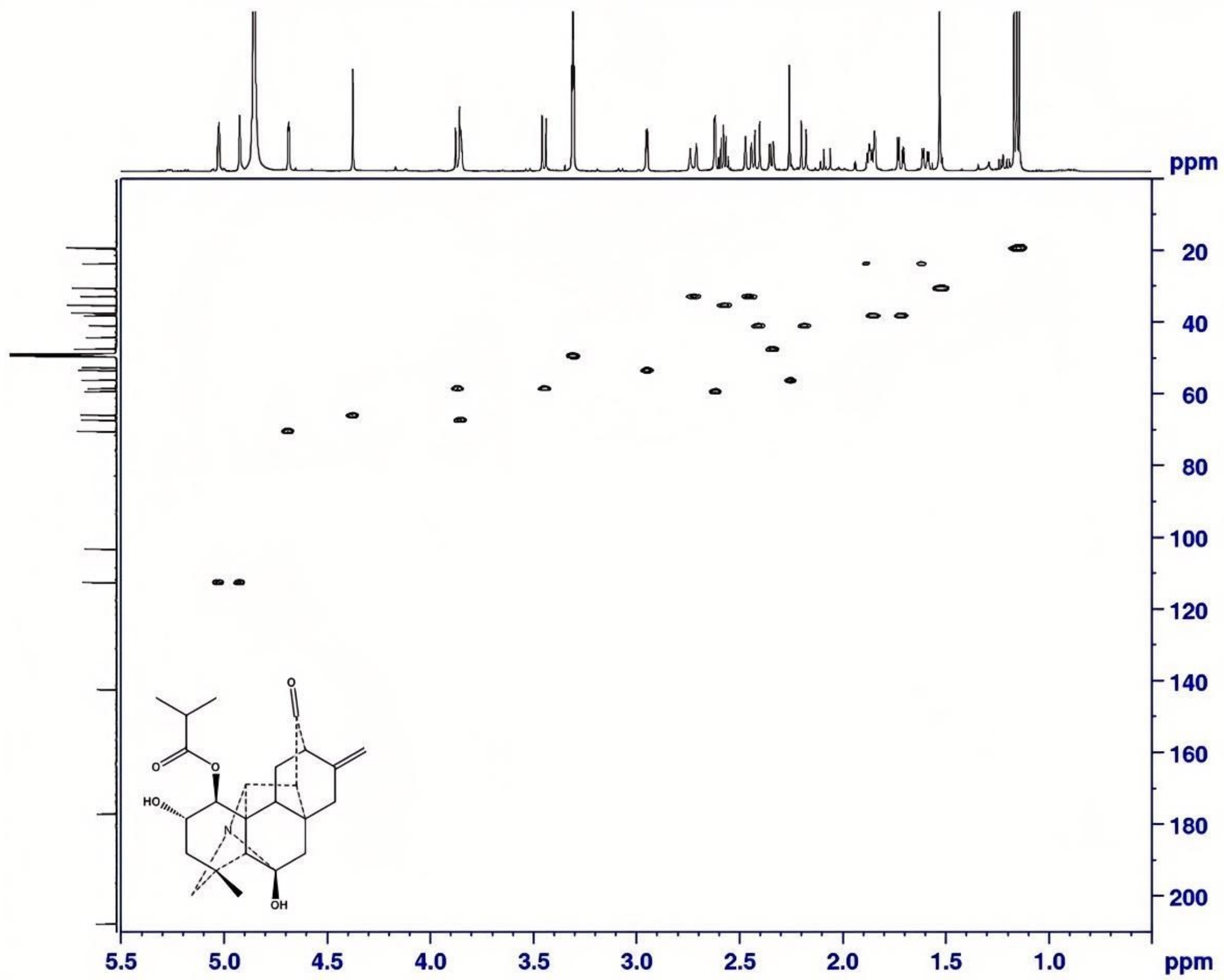


**Figure S44.** HSQC spectrum of **5**

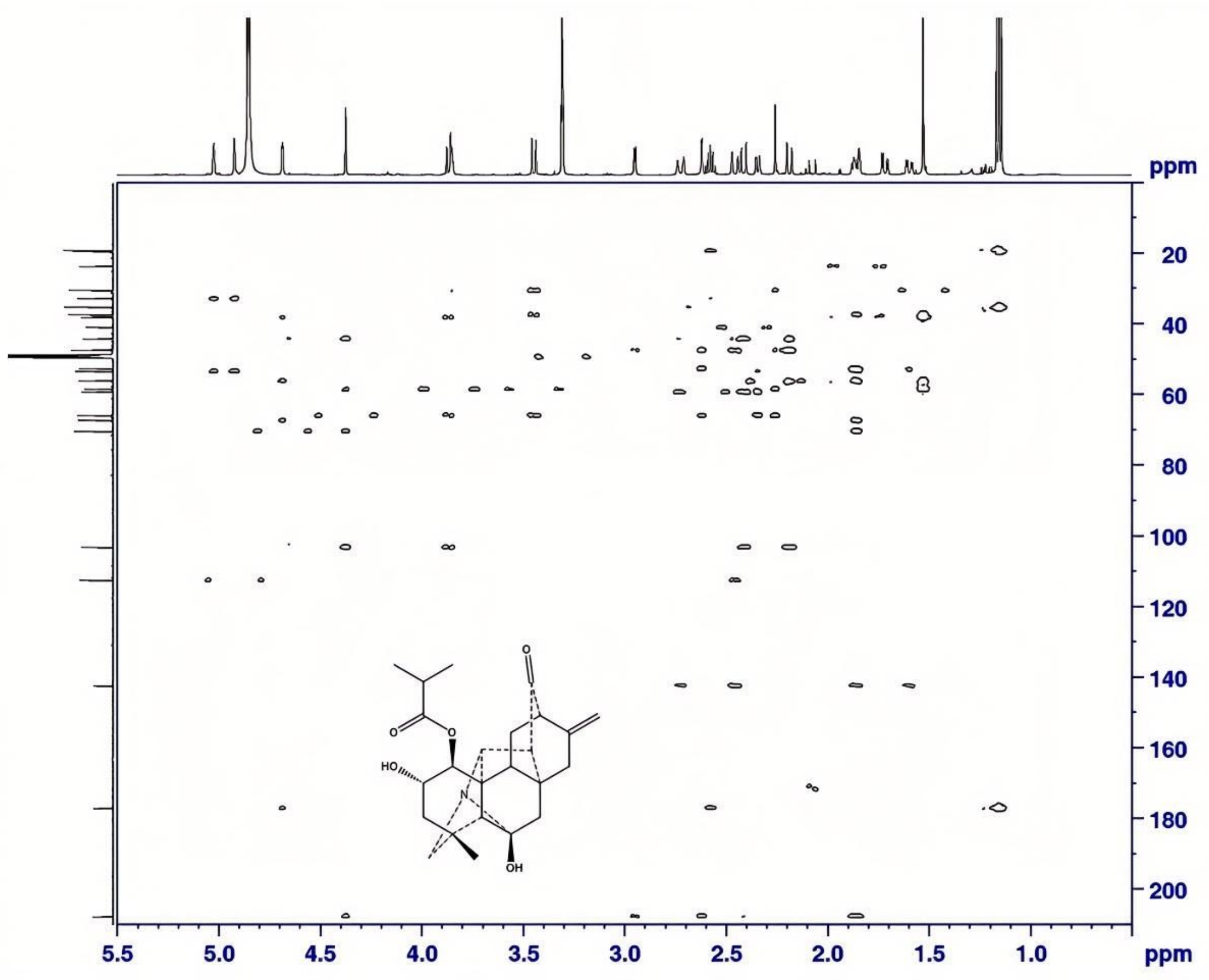


**Figure S45.** HMBC spectrum of **5**

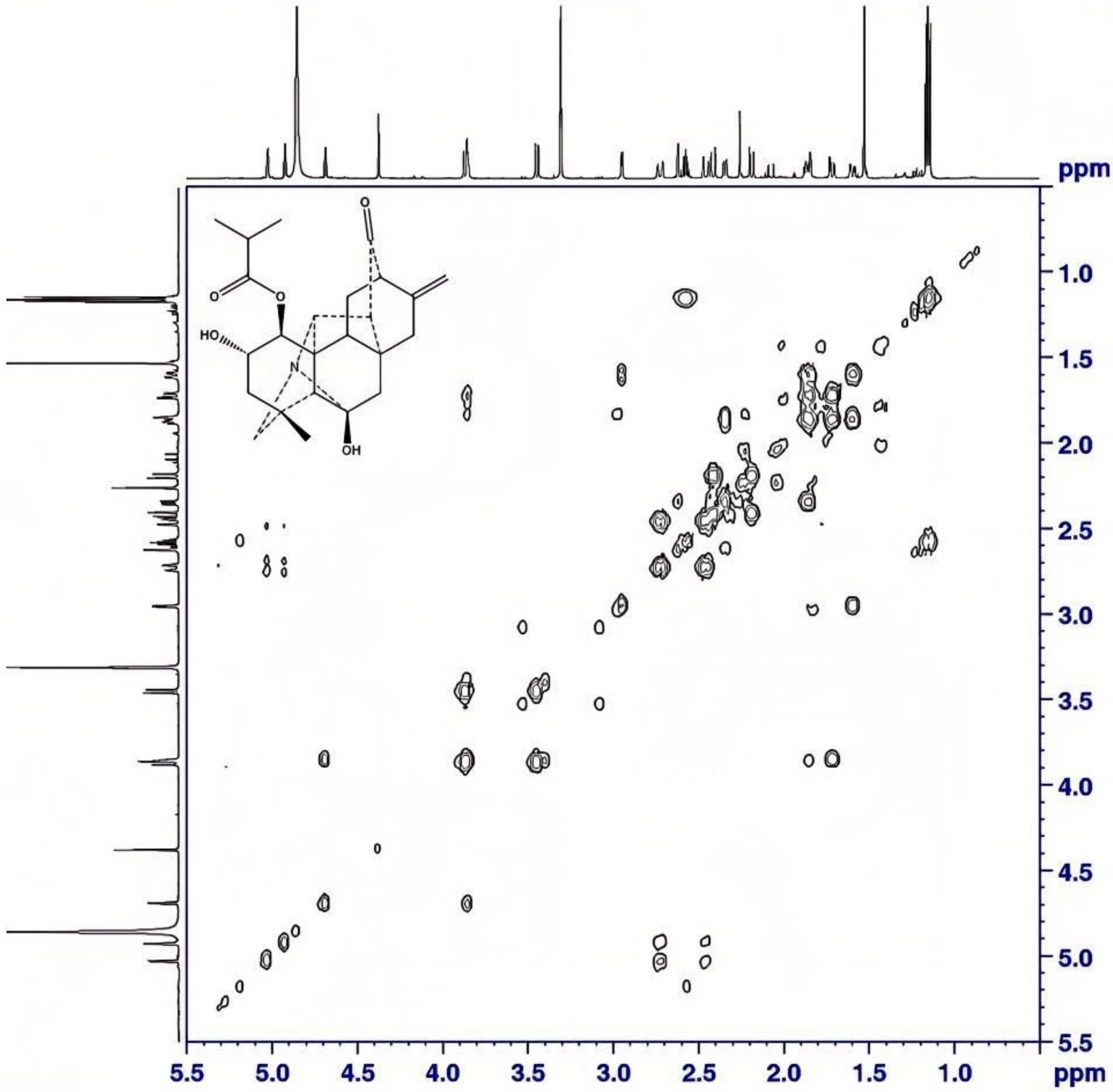


**Figure S46.** $^{1}$H-$^{1}$H COSY spectrum of **5**

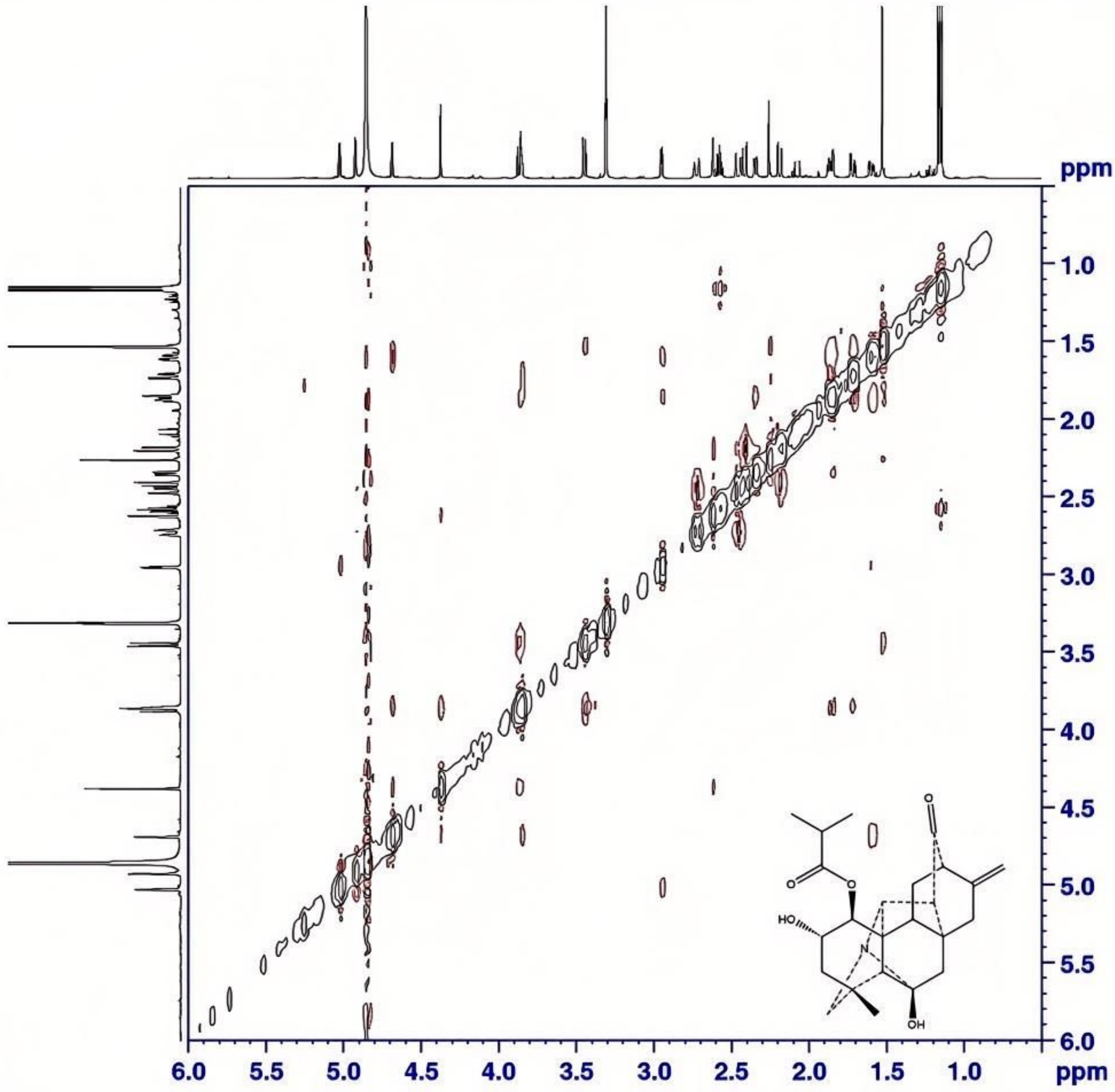

**Figure S47.** NOESY spectrum of **5**

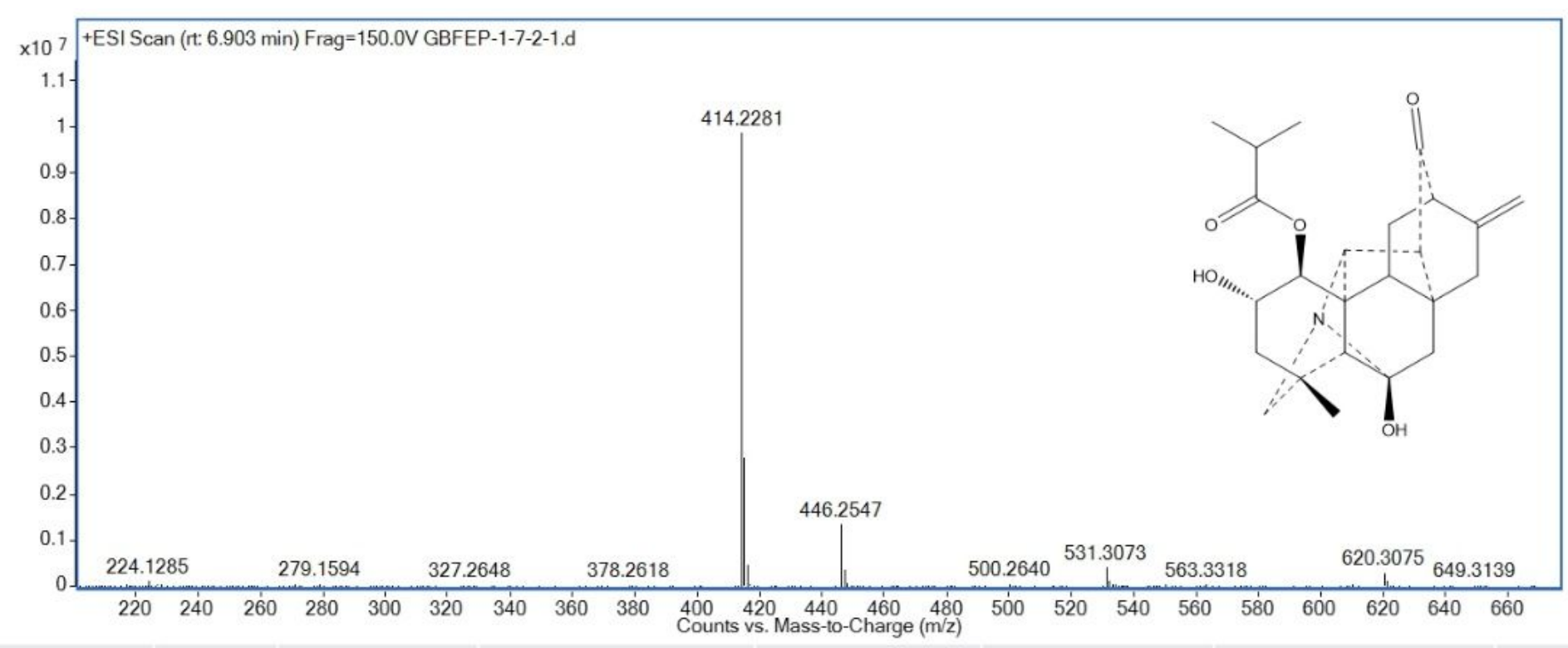

| Formula | Score | Mass | Mass (MFG) | m/z (Calc) | Diff (ppm) | Ion Formula | m/z |
|---|---|---|---|---|---|---|---|
| $C_{24}H_{31}NO_5$ | 99.09 | 413.2208 | 413.2202 | 414.2275 | -1.45 | $C_{24}H_{32}NO_5$ | 414.2281 |

**Figure S48.** HRESIMS spectrum of **5**

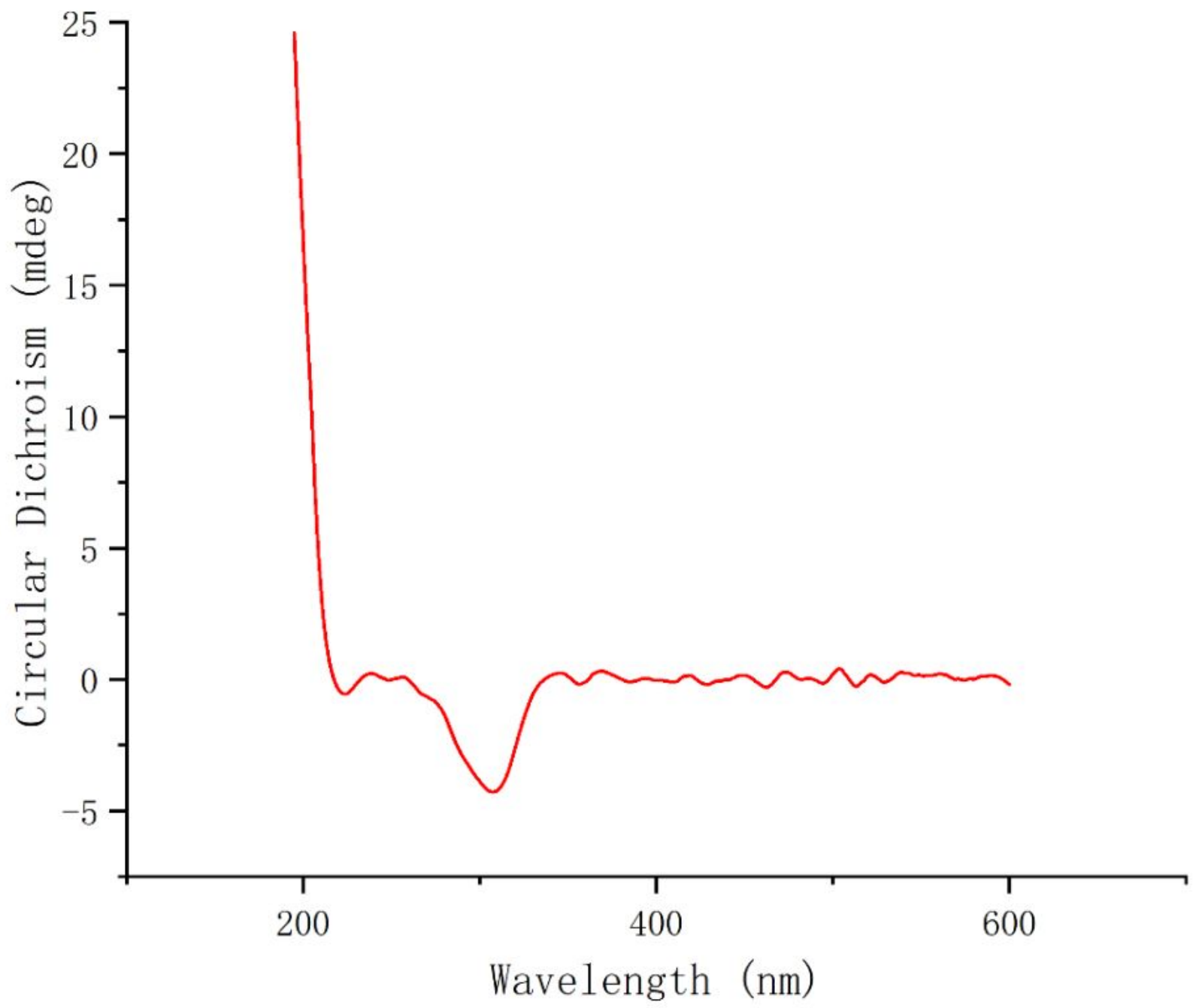


**Figure S49.** ECD spectrum of **5**

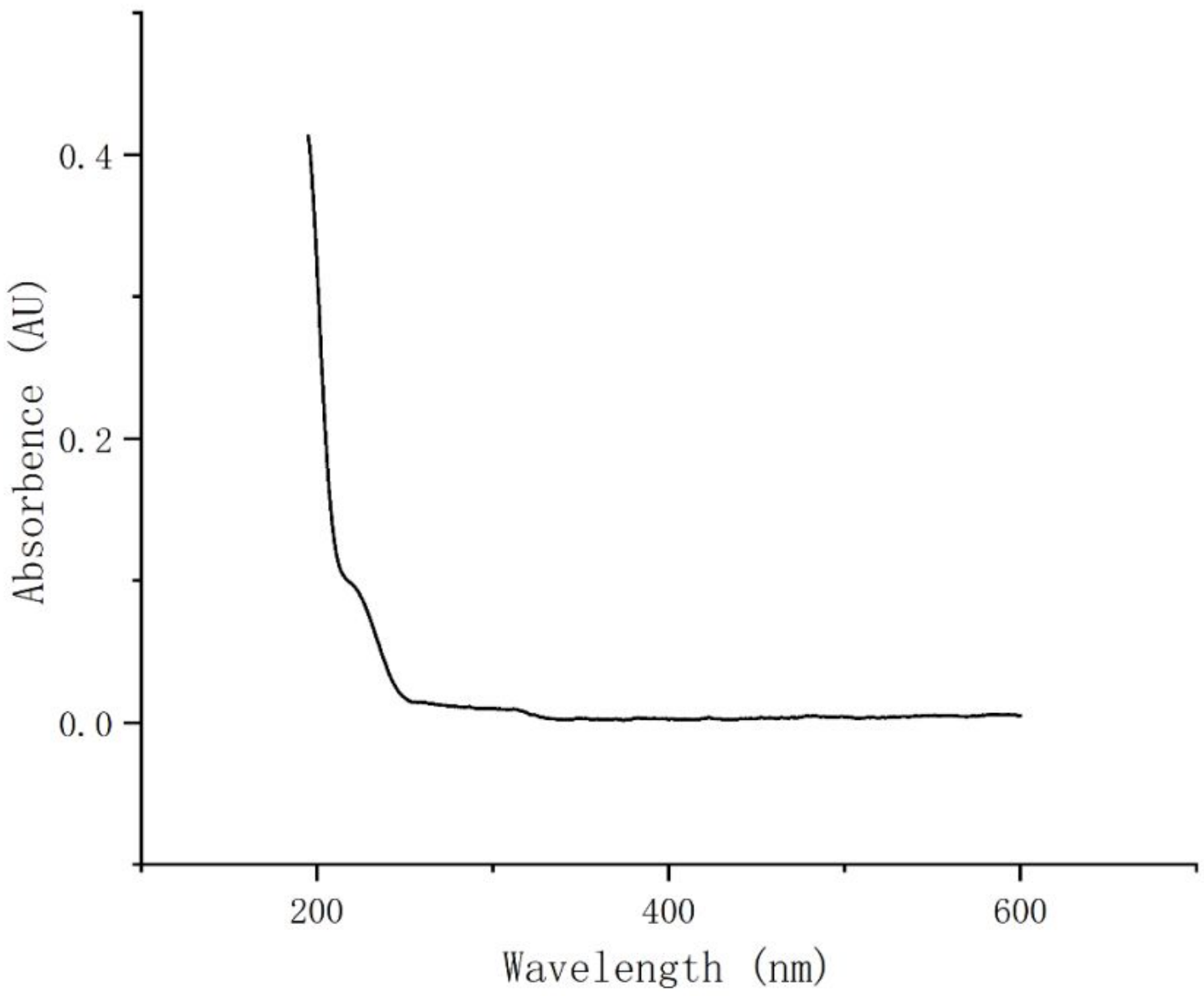


**Figure S50.** UV spectrum of **5**

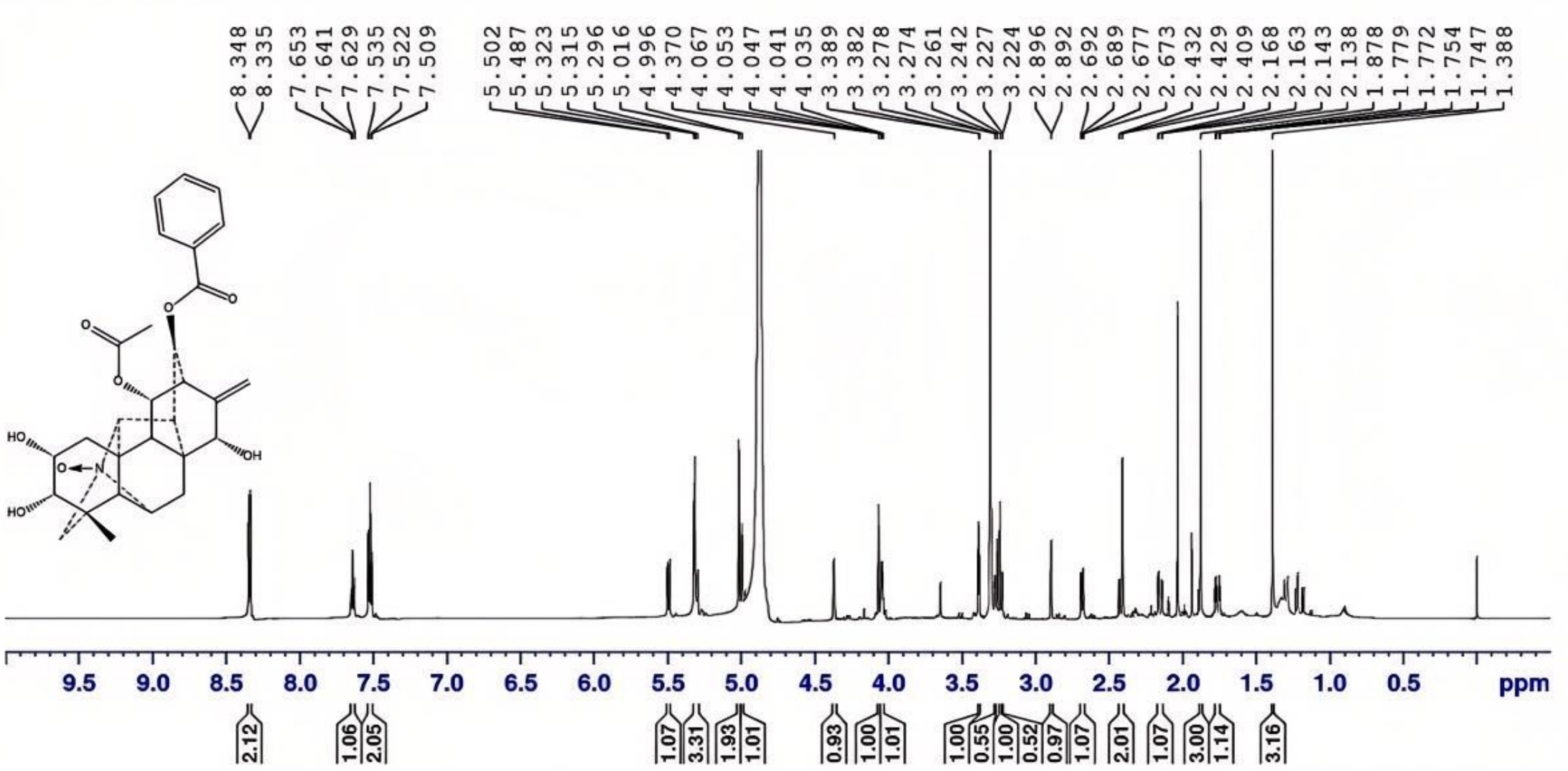


**Figure S51.** $^{1}$H NMR spectrum of **6** in methanol-$d_4$

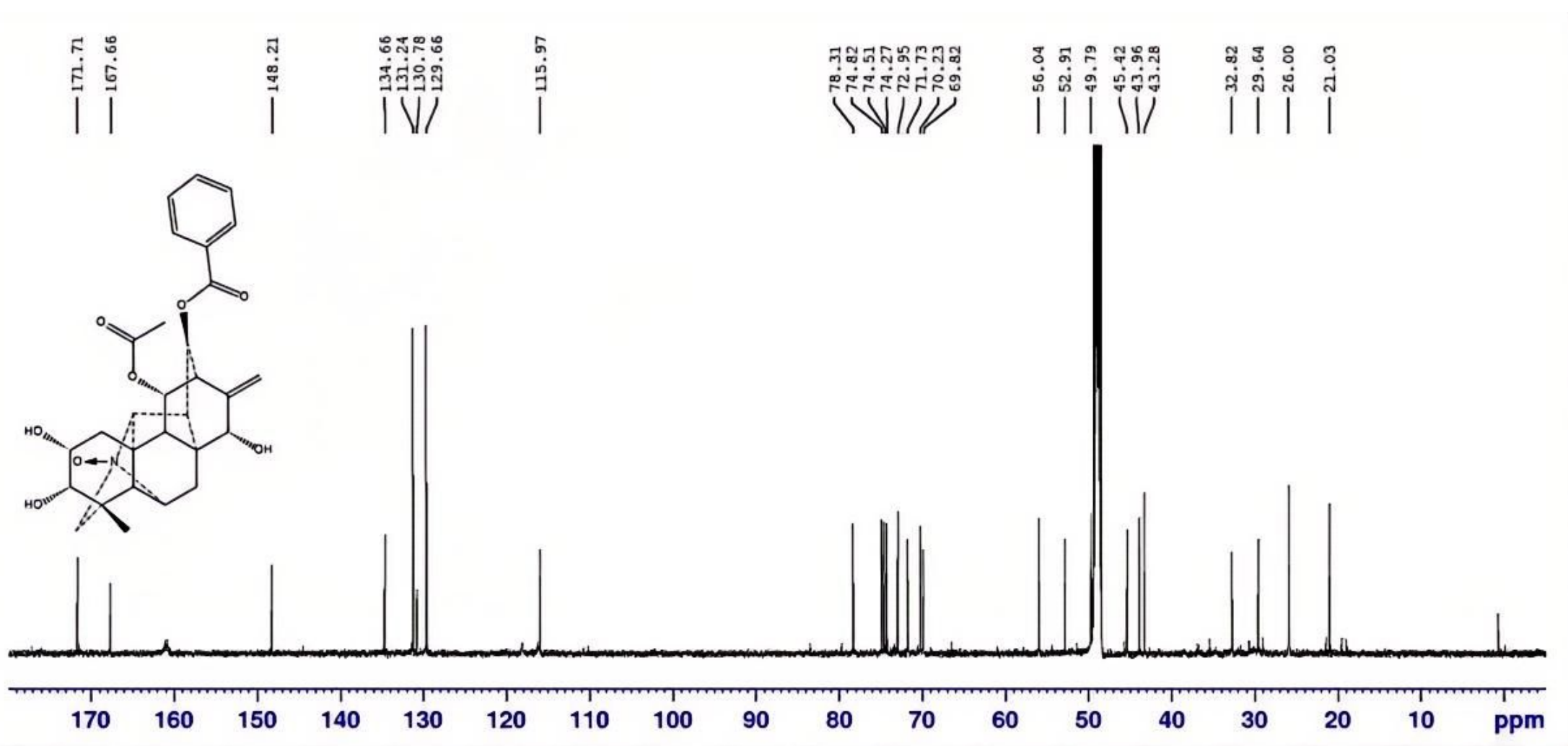


**Figure S52.** $^{13}$C NMR spectrum of **6** in methanol-$d_4$

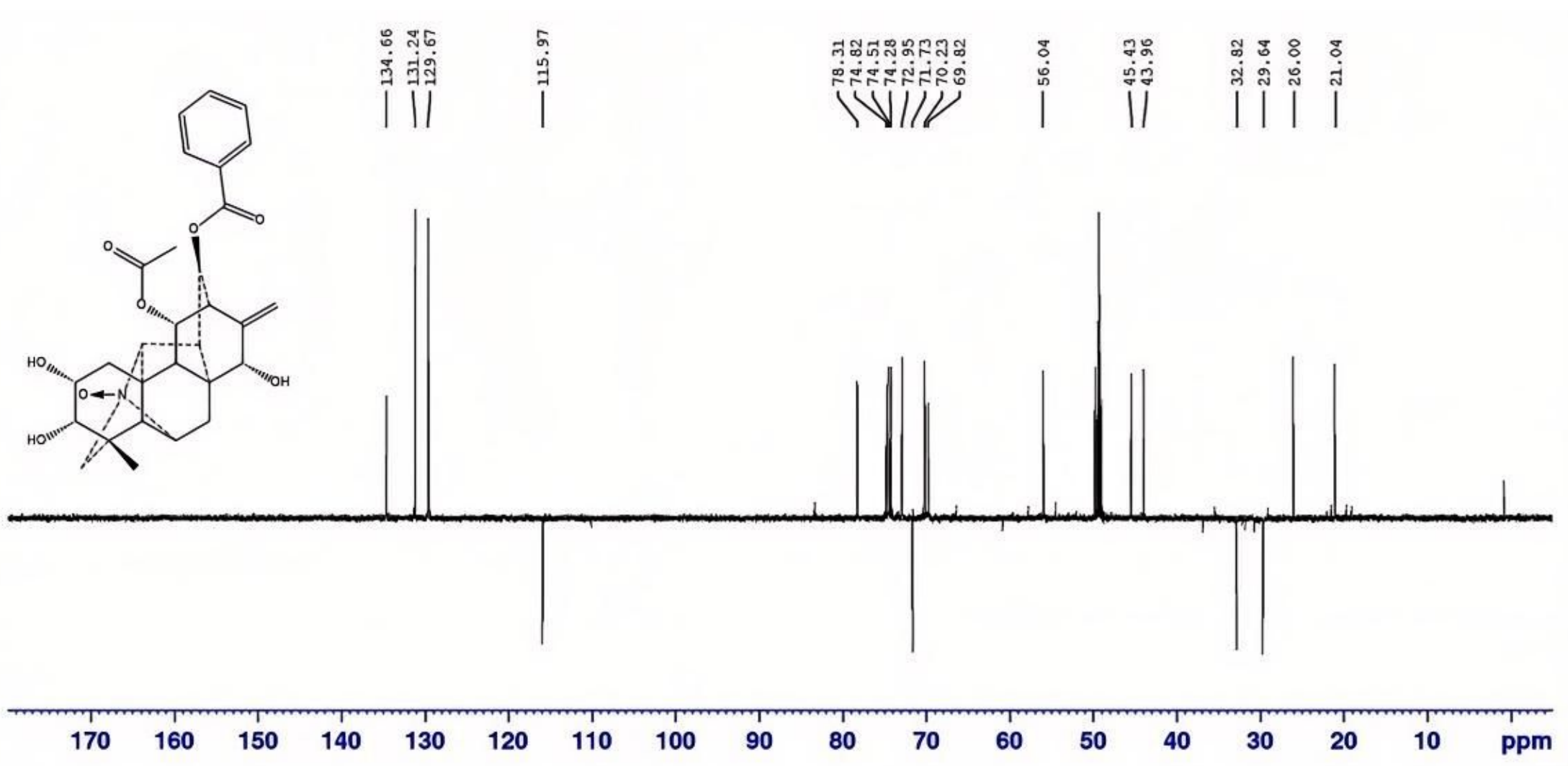

**Figure S53.** DEPT NMR spectrum of **6** in methanol-$d_4$

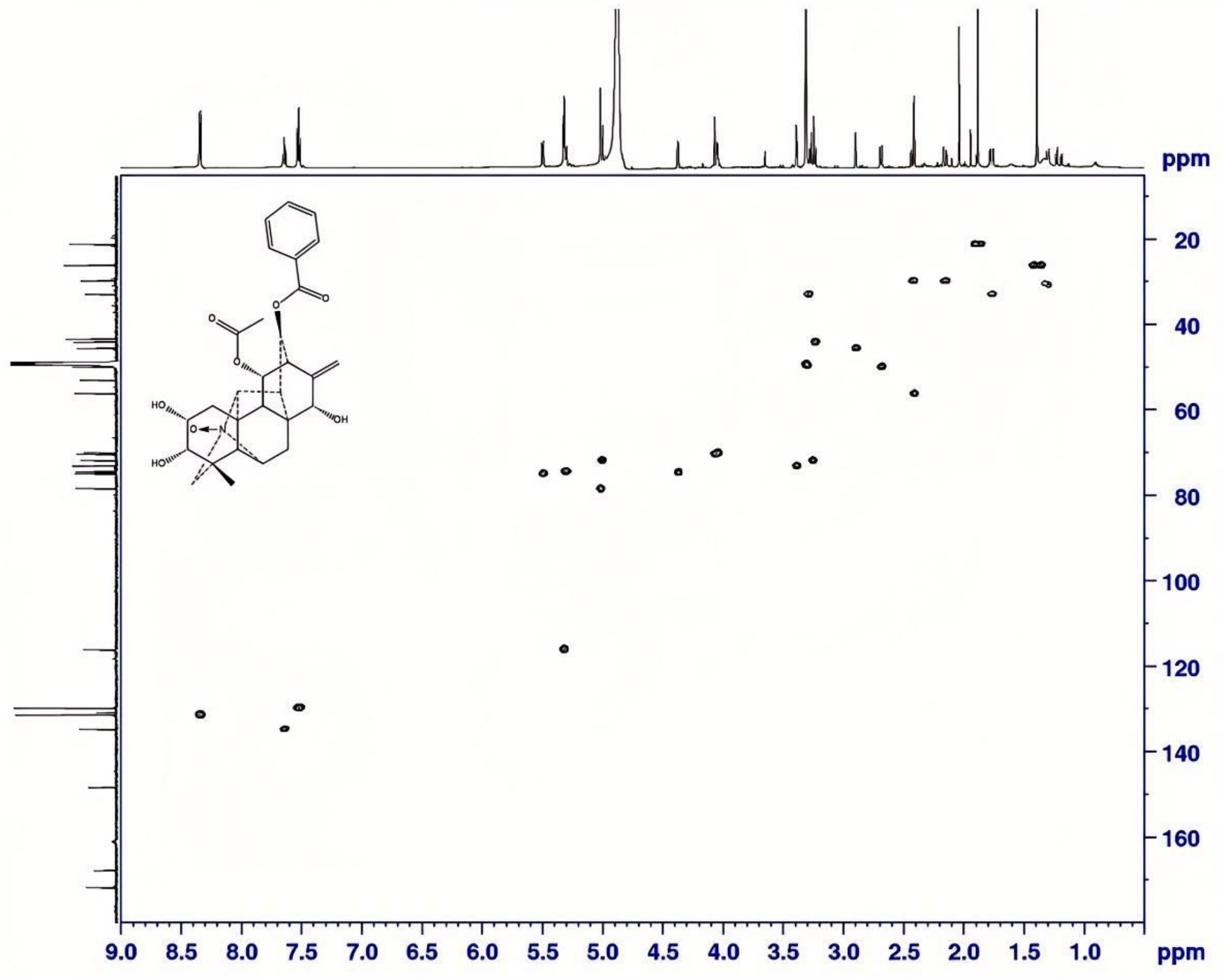


**Figure S54.** HSQC spectrum of **6**

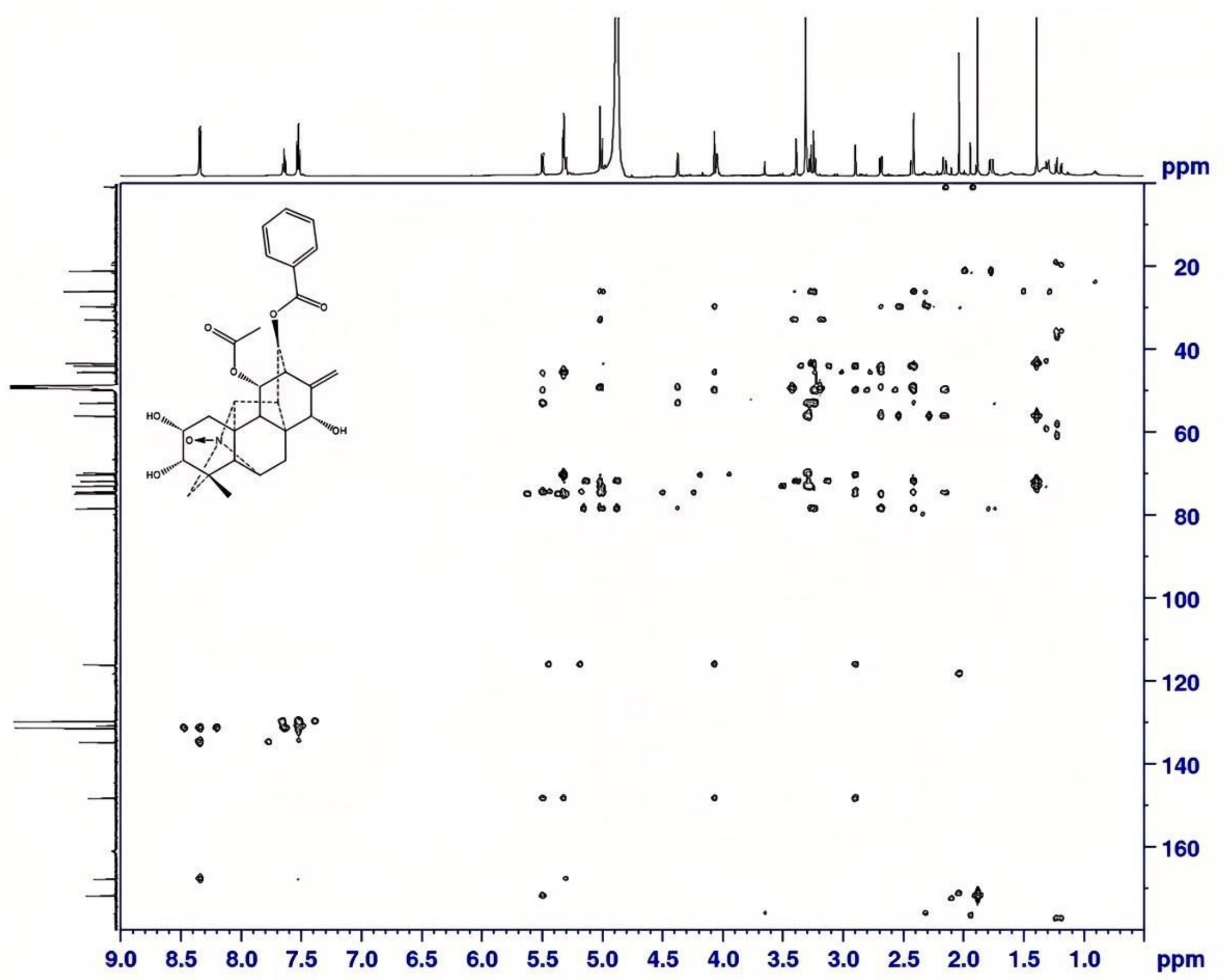


**Figure S55.** HMBC spectrum of **6**

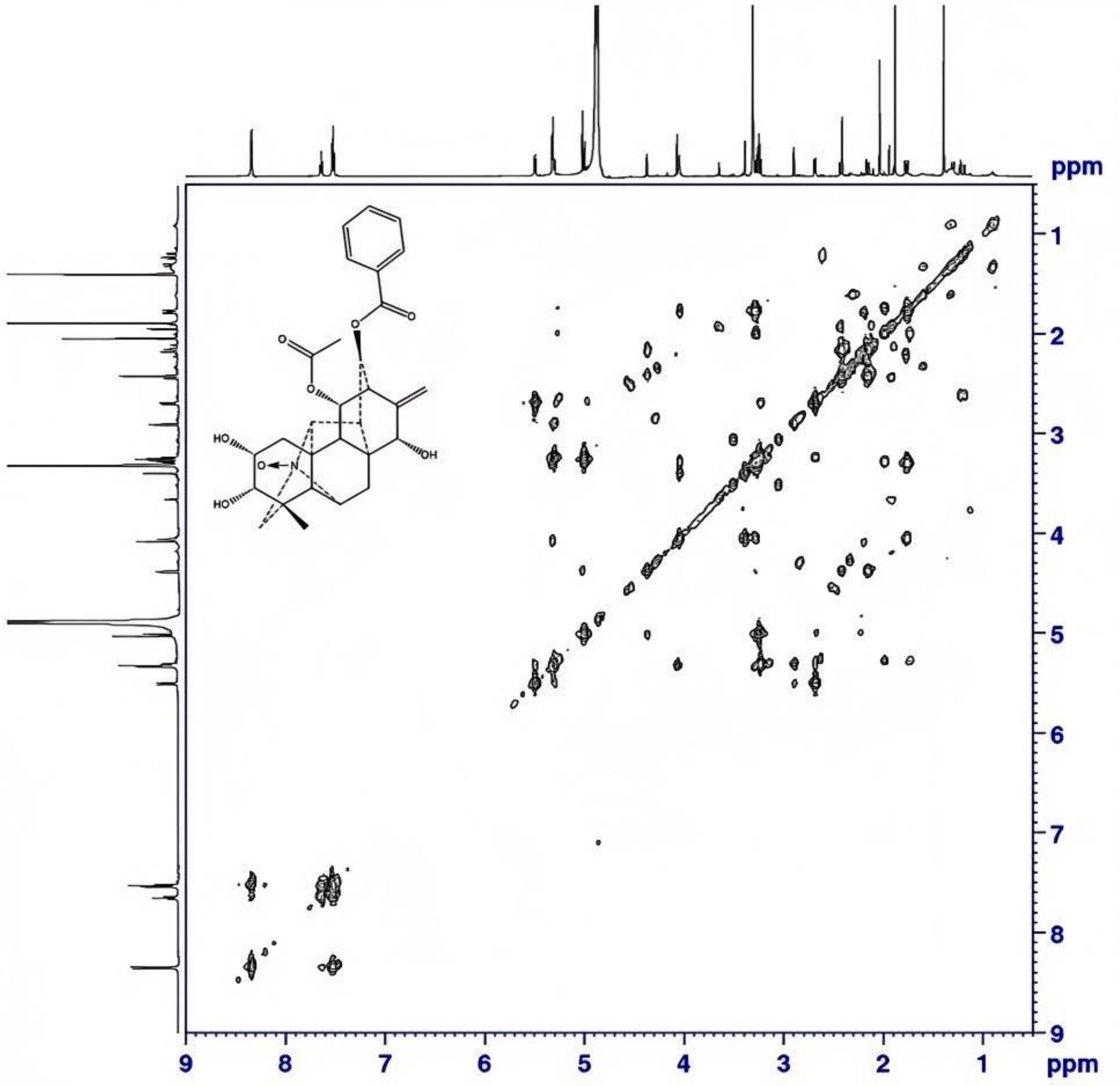


**Figure S56.** $^{1}H$-$^{1}H$ COSY spectrum of **6**

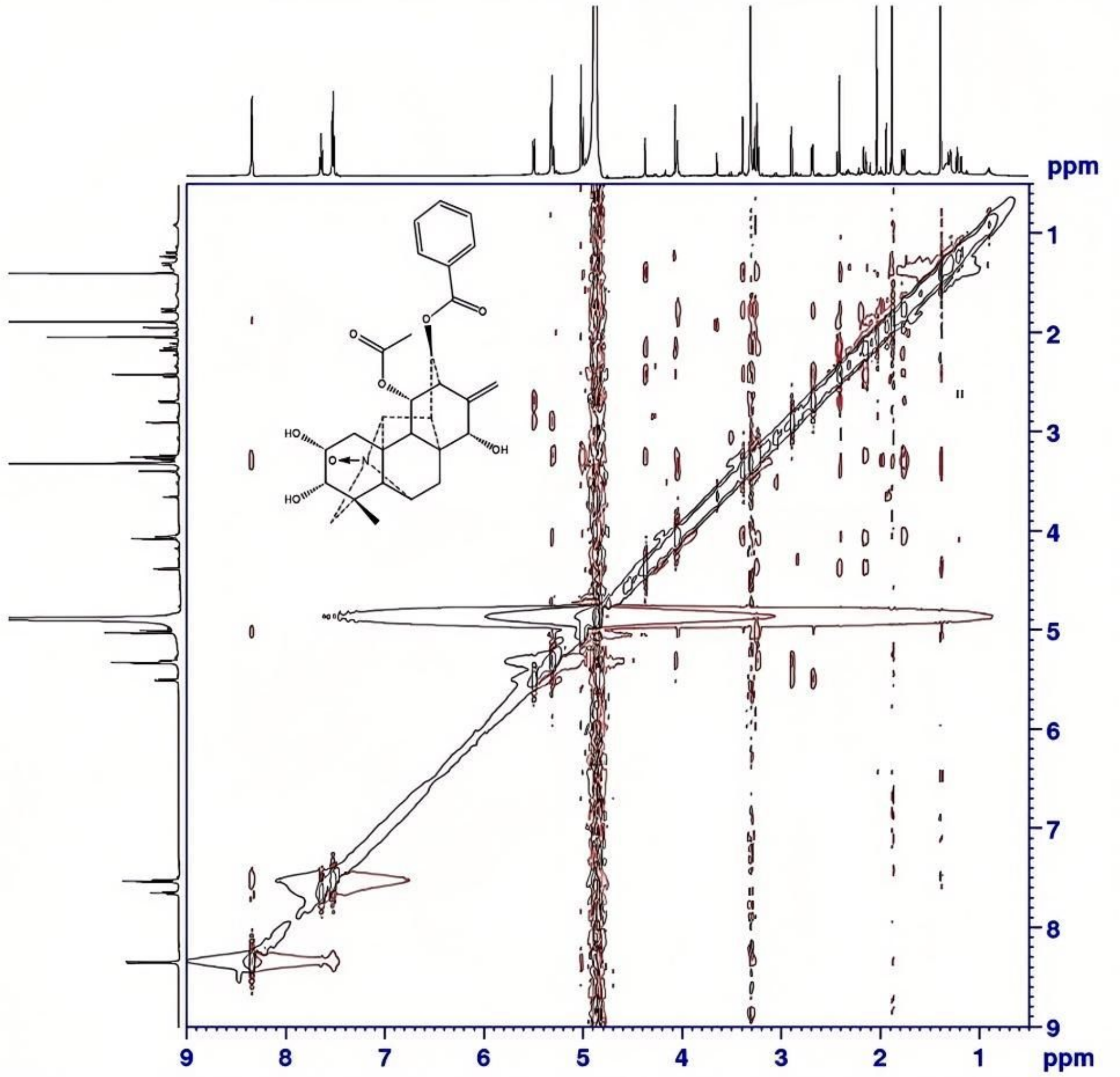

**Figure S57.** NOESY spectrum of **6**

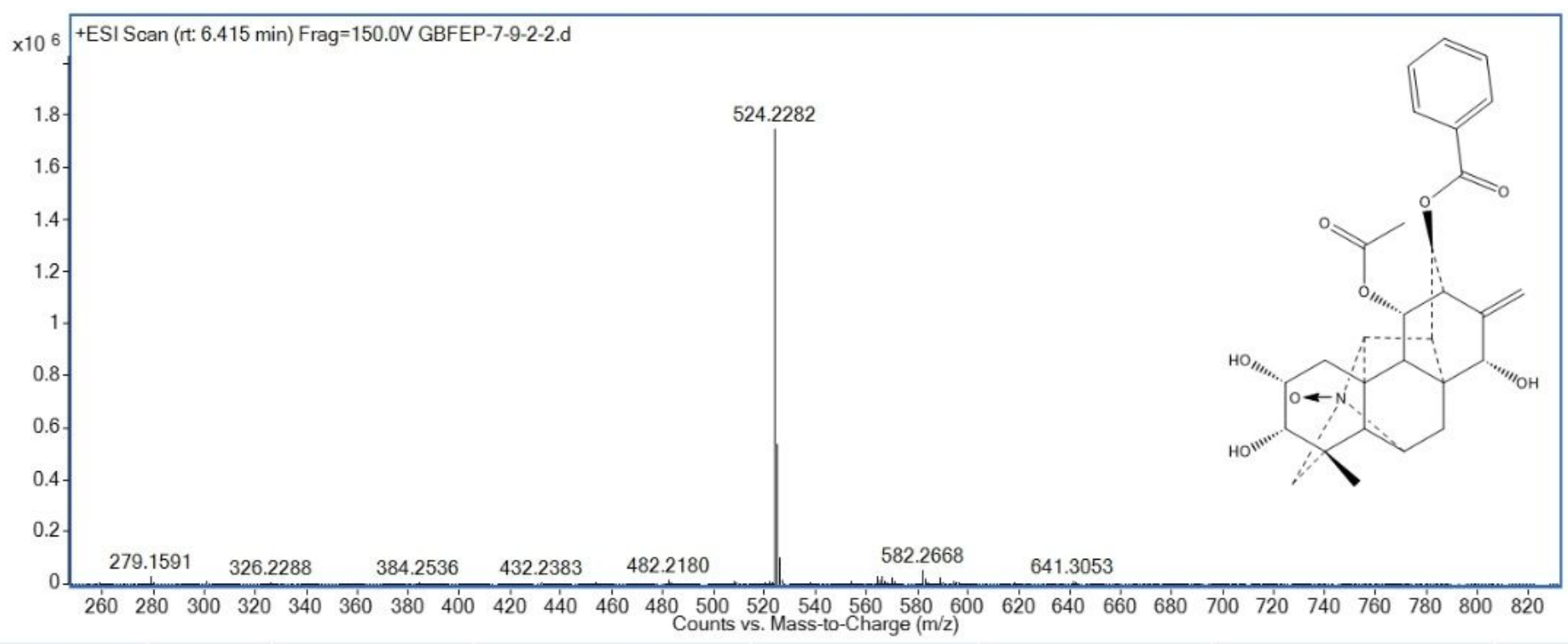


| Formula | Score | Mass | Mass (MFG) | m/z (Calc) | Diff (ppm) | Ion Formula | m/z |
|---|---|---|---|---|---|---|---|
| $C_{29}H_{33}NO_8$ | 99.82 | 523.2209 | 523.2206 | 524.2279 | -0.59 | $C_{29}H_{34}NO_8$ | 524.2282 |

**Figure S58.** HRESIMS spectrum of **6**

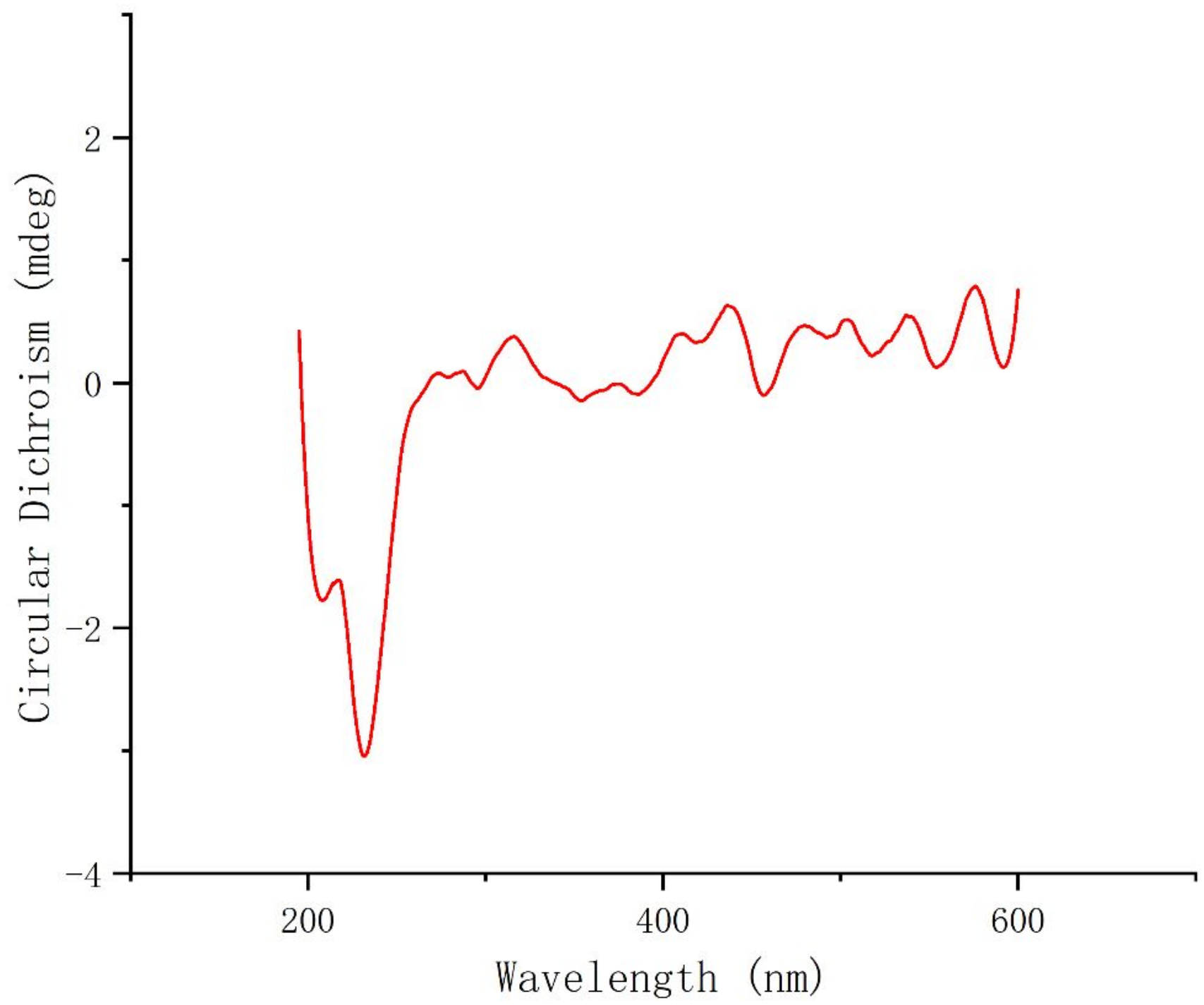


**Figure S59.** ECD spectrum of **6**

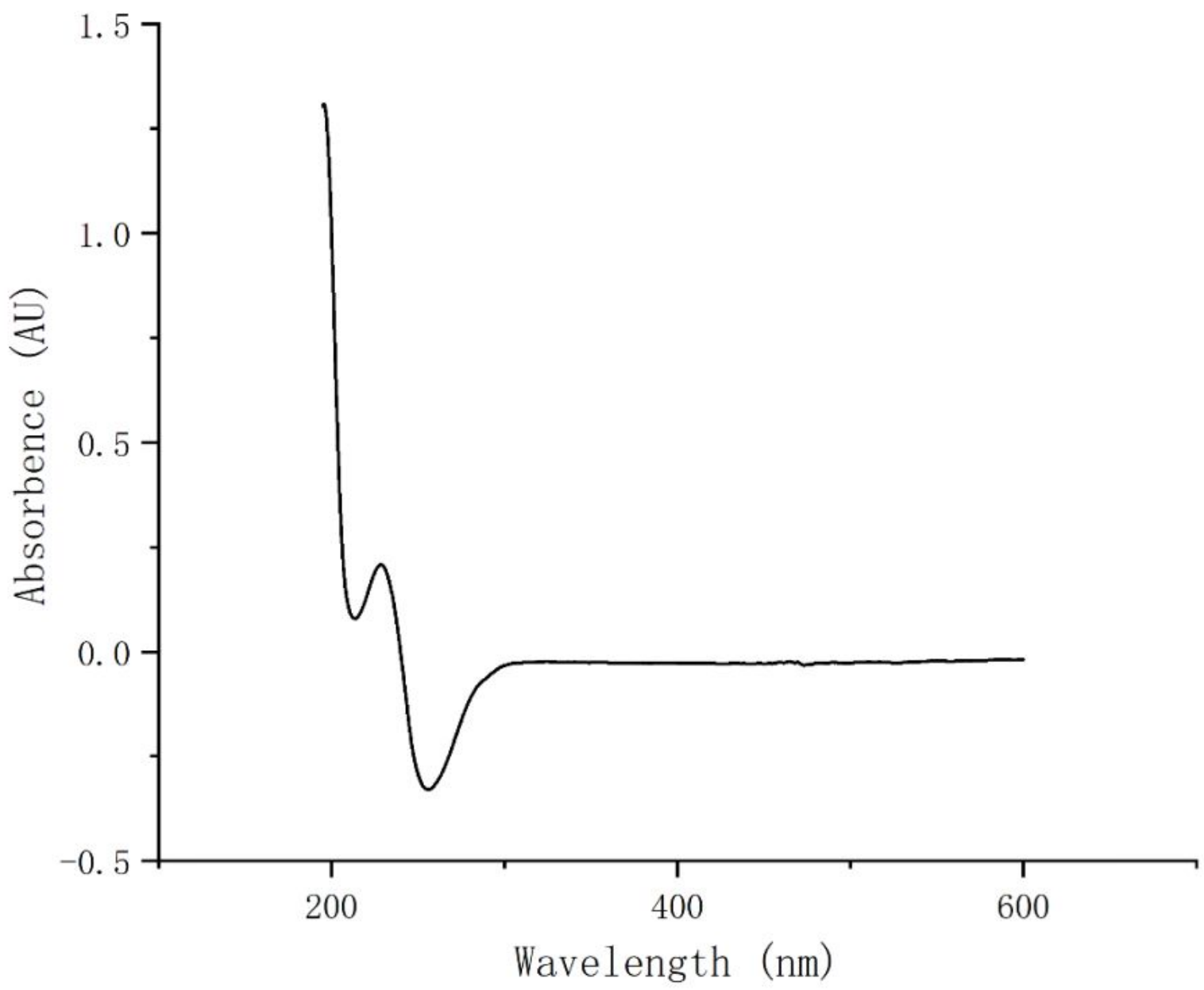


**Figure S60.** UV spectrum of **6**

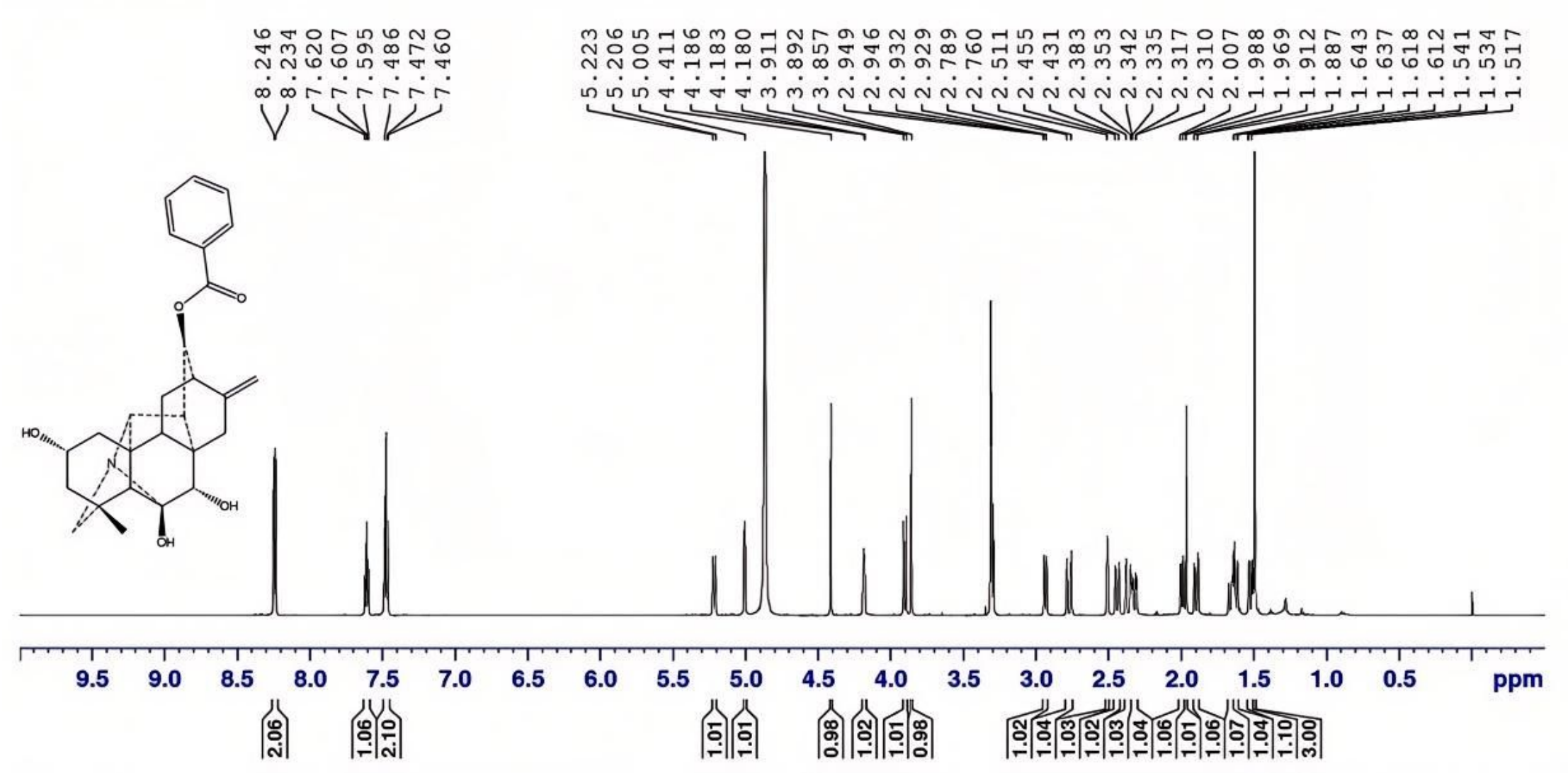


**Figure S61.** $^{1}$H NMR spectrum of **7** in methanol-$d_4$

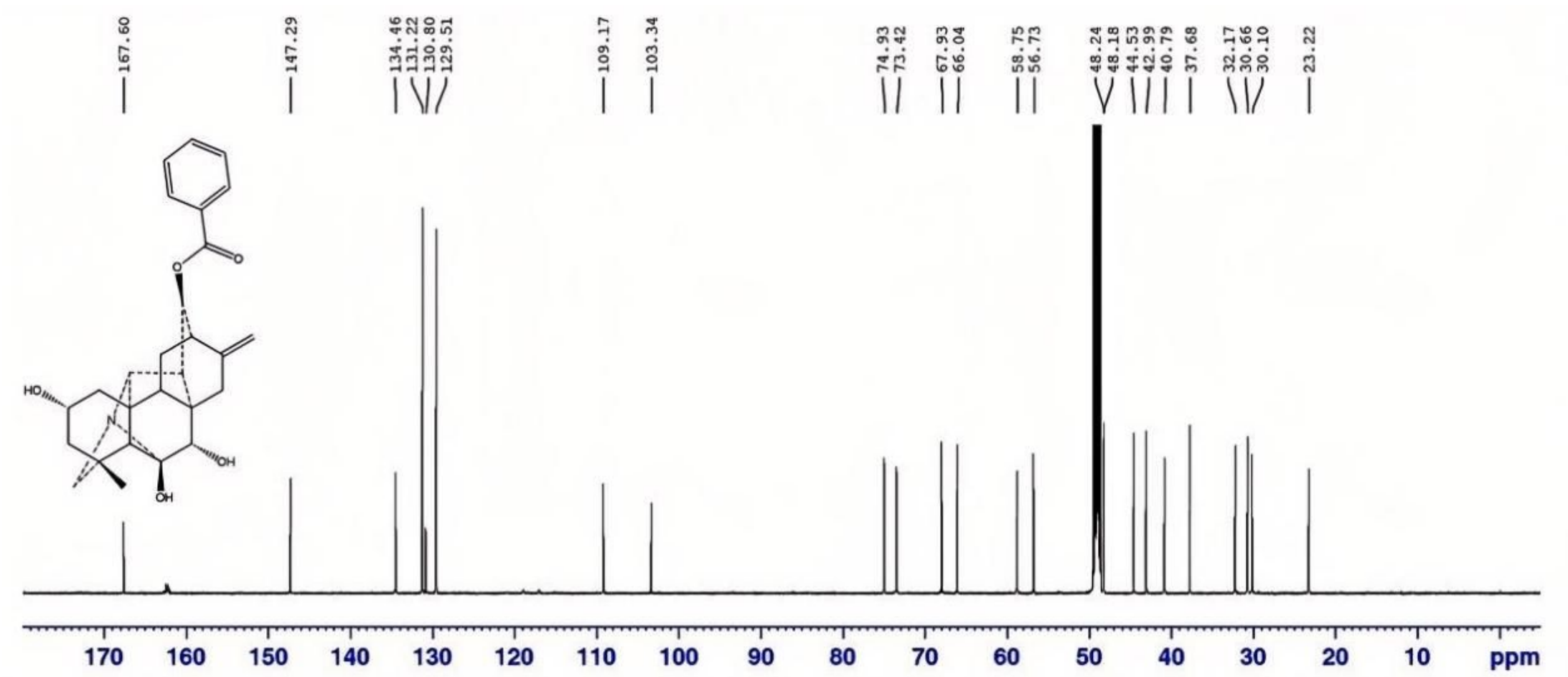


**Figure S62.** $^{13}$C NMR spectrum of **7** in methanol-$d_4$

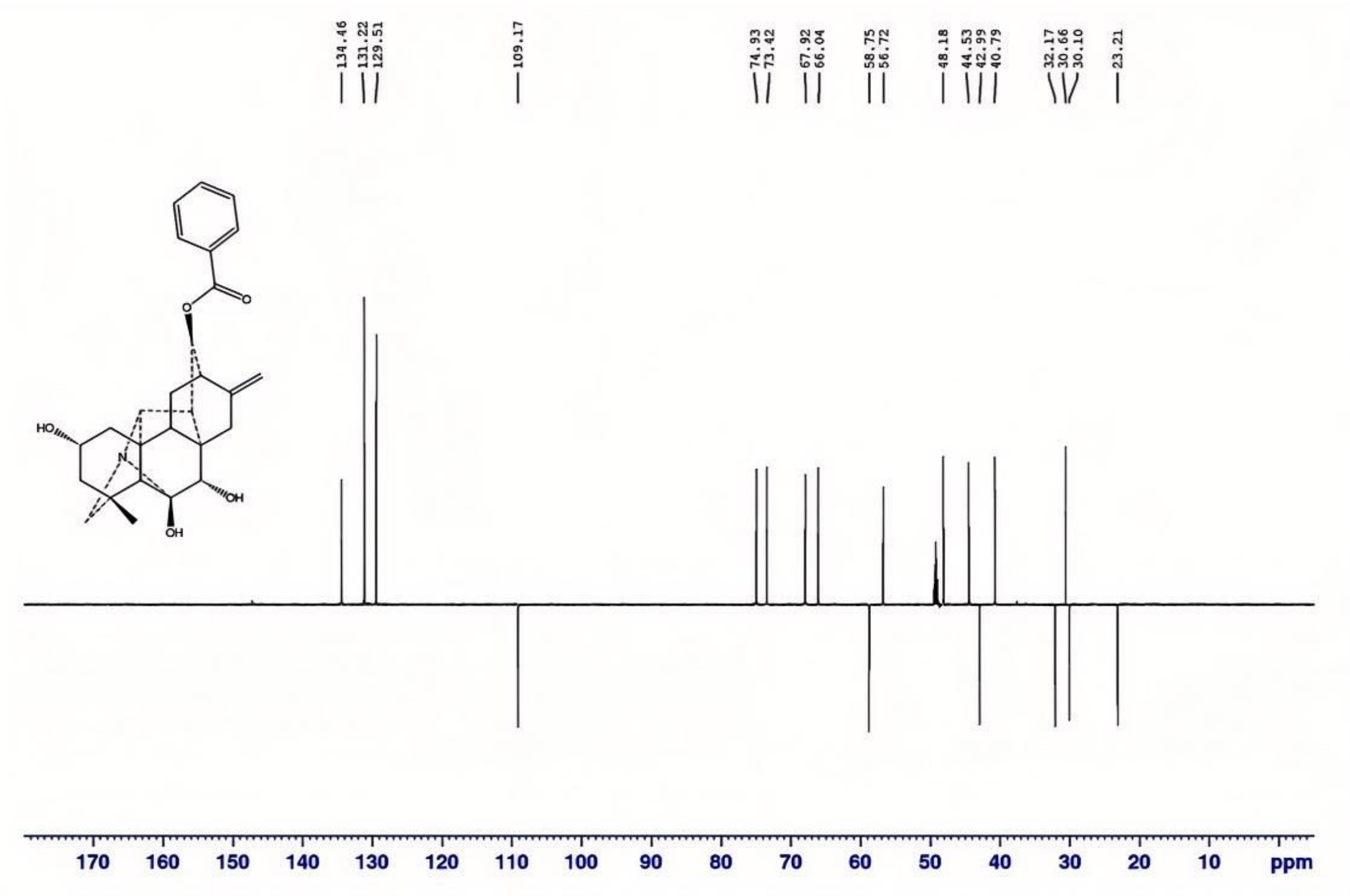


**Figure S63.** DEPT NMR spectrum of **7** in methanol-$d_4$

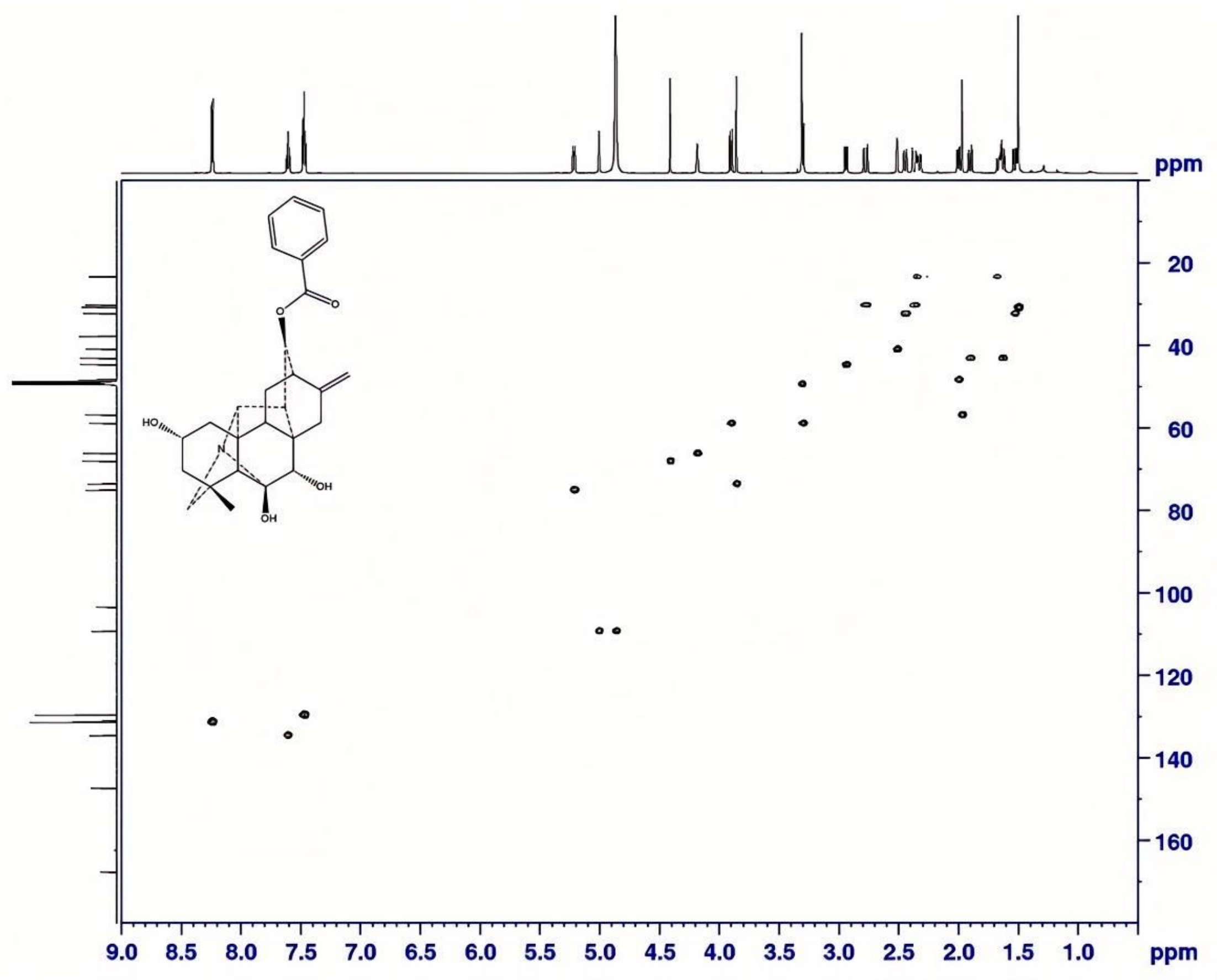


**Figure S64.** HSQC spectrum of **7**

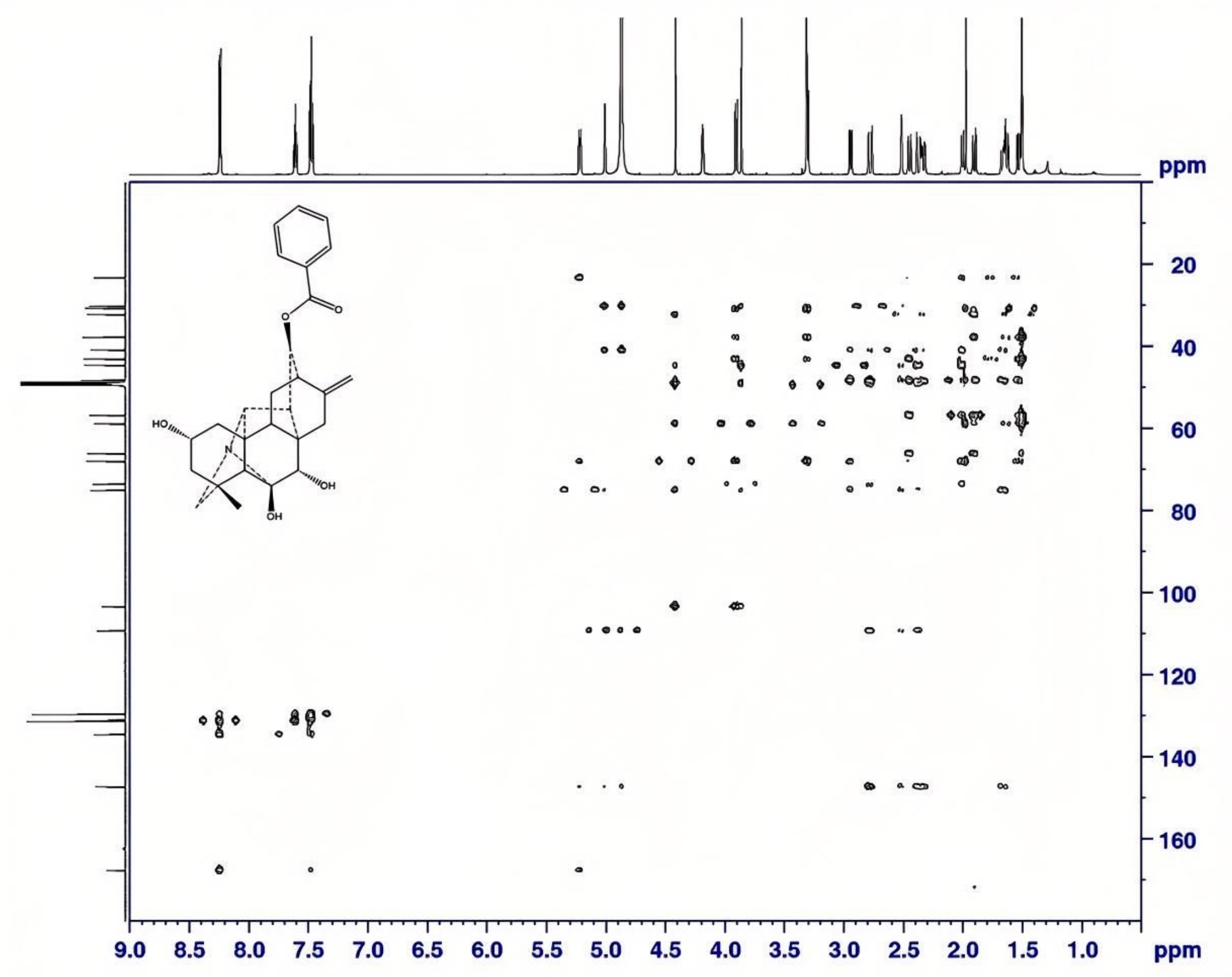


**Figure S65.** HMBC spectrum of **7**

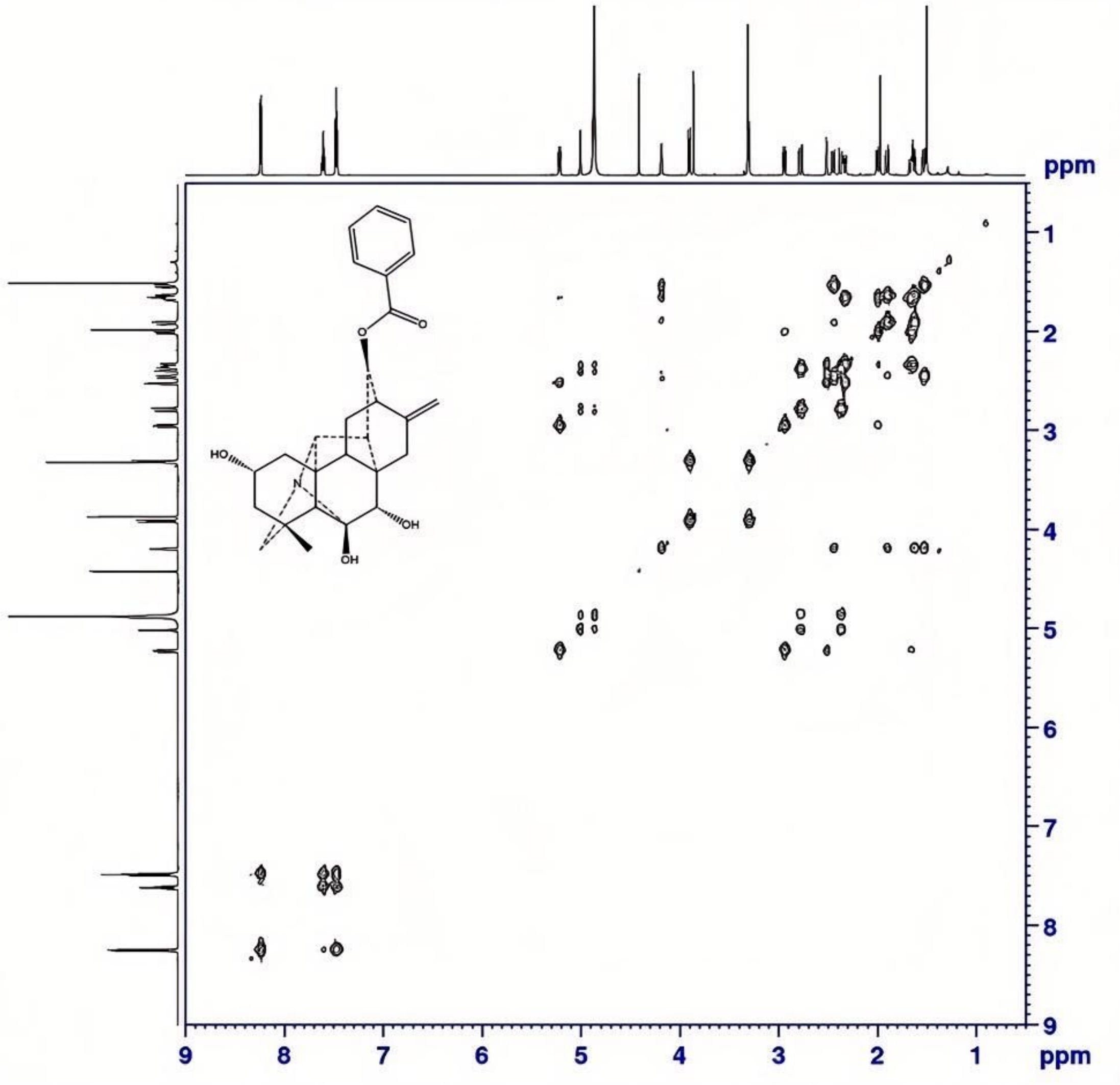


**Figure S66.** $^{1}H$-$^{1}H$ COSY spectrum of **7**

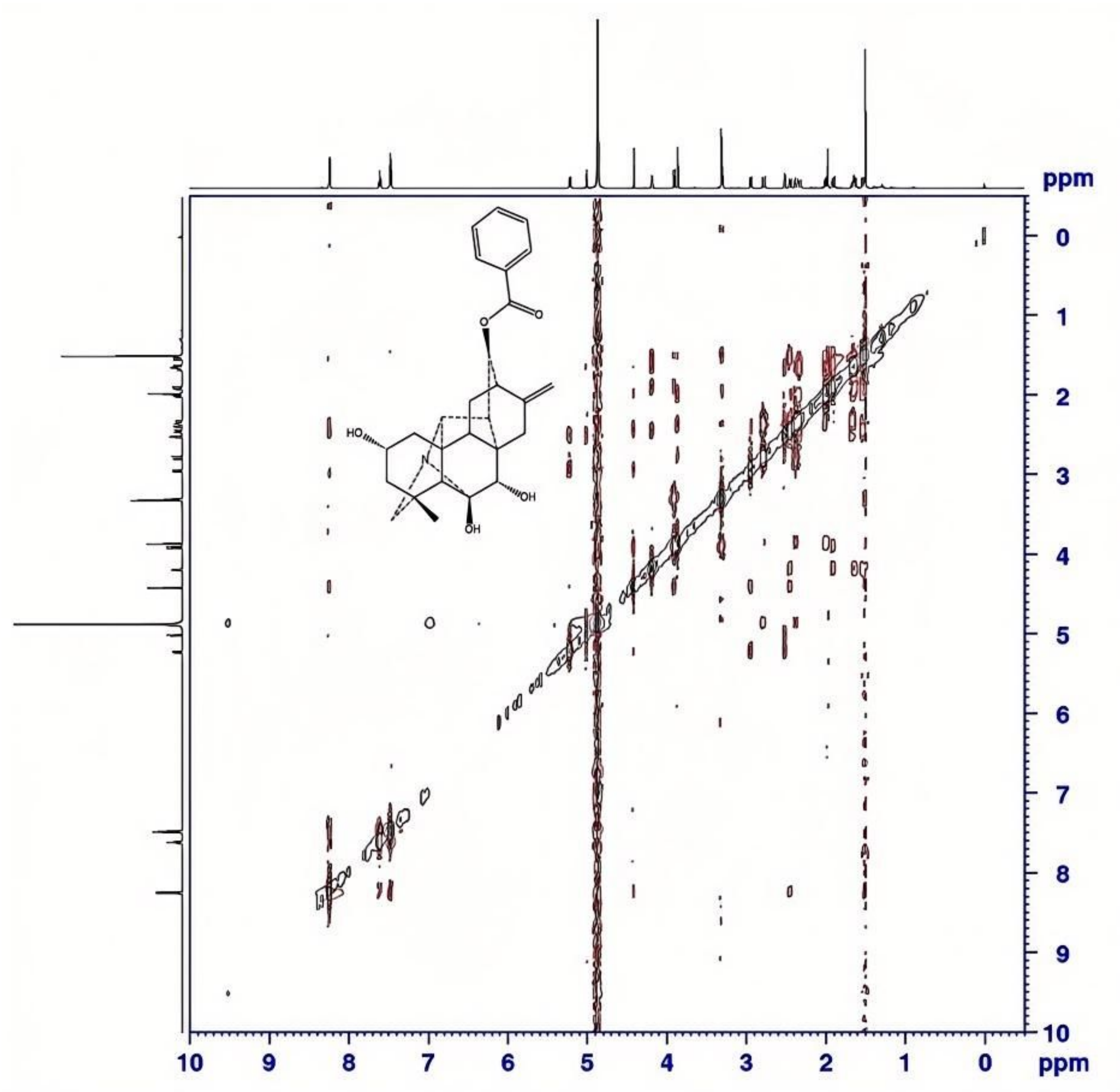


**Figure S67.** NOESY spectrum of **7**

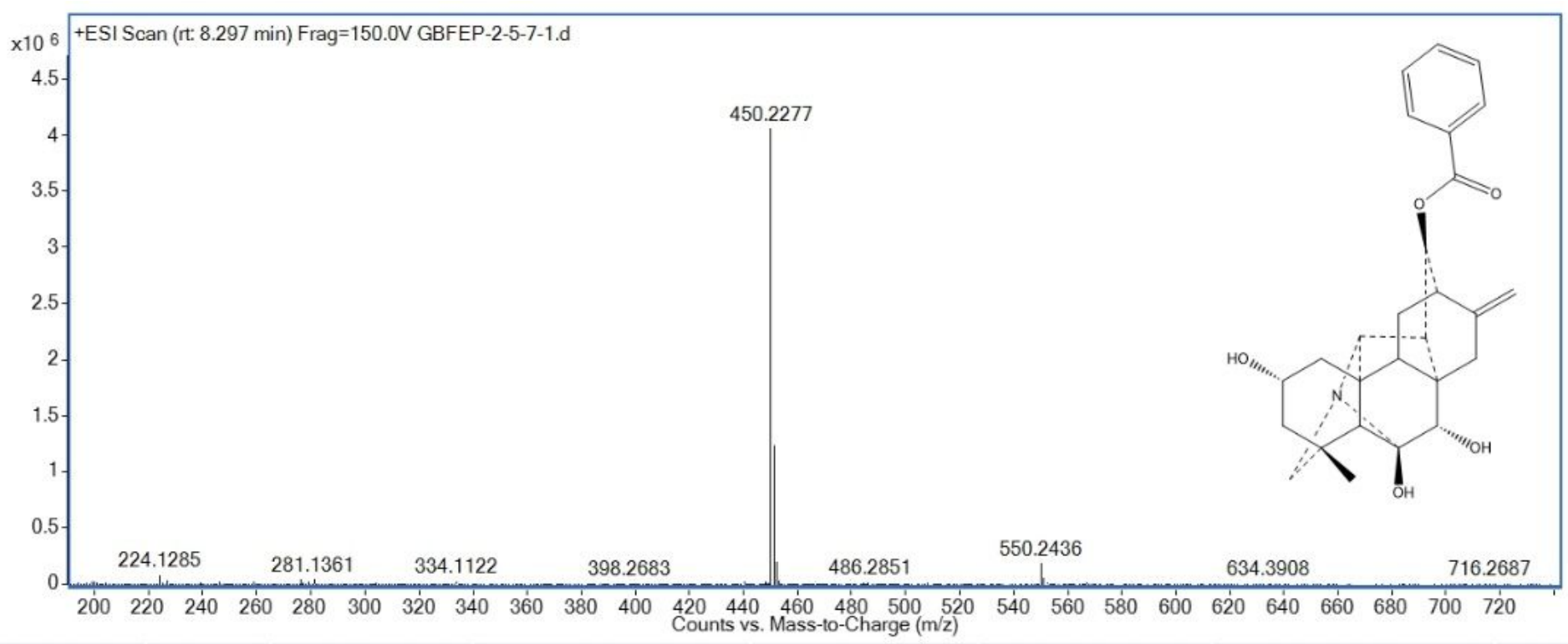


| Formula | Score | Mass | Mass (MFG) | m/z (Calc) | Diff (ppm) | Ion Formula | m/z |
|---|---|---|---|---|---|---|---|
| $C_{27}H_{31}NO_5$ | 99.91 | 449.2204 | 449.2202 | 450.2275 | -0.45 | $C_{27}H_{32}NO_5$ | 450.2277 |

**Figure S68.** HRESIMS spectrum of **7**

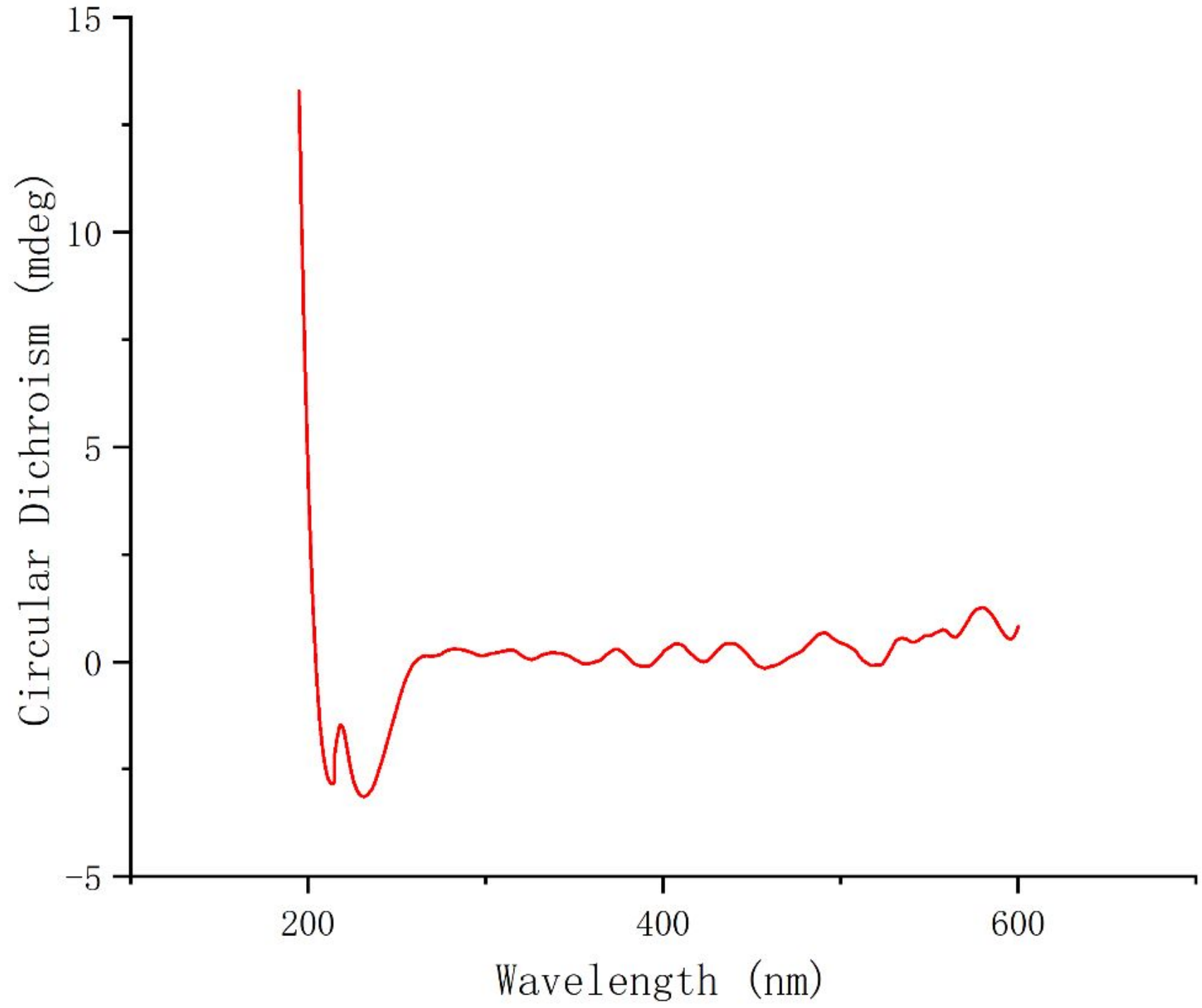


**Figure S69.** ECD spectrum of **7**

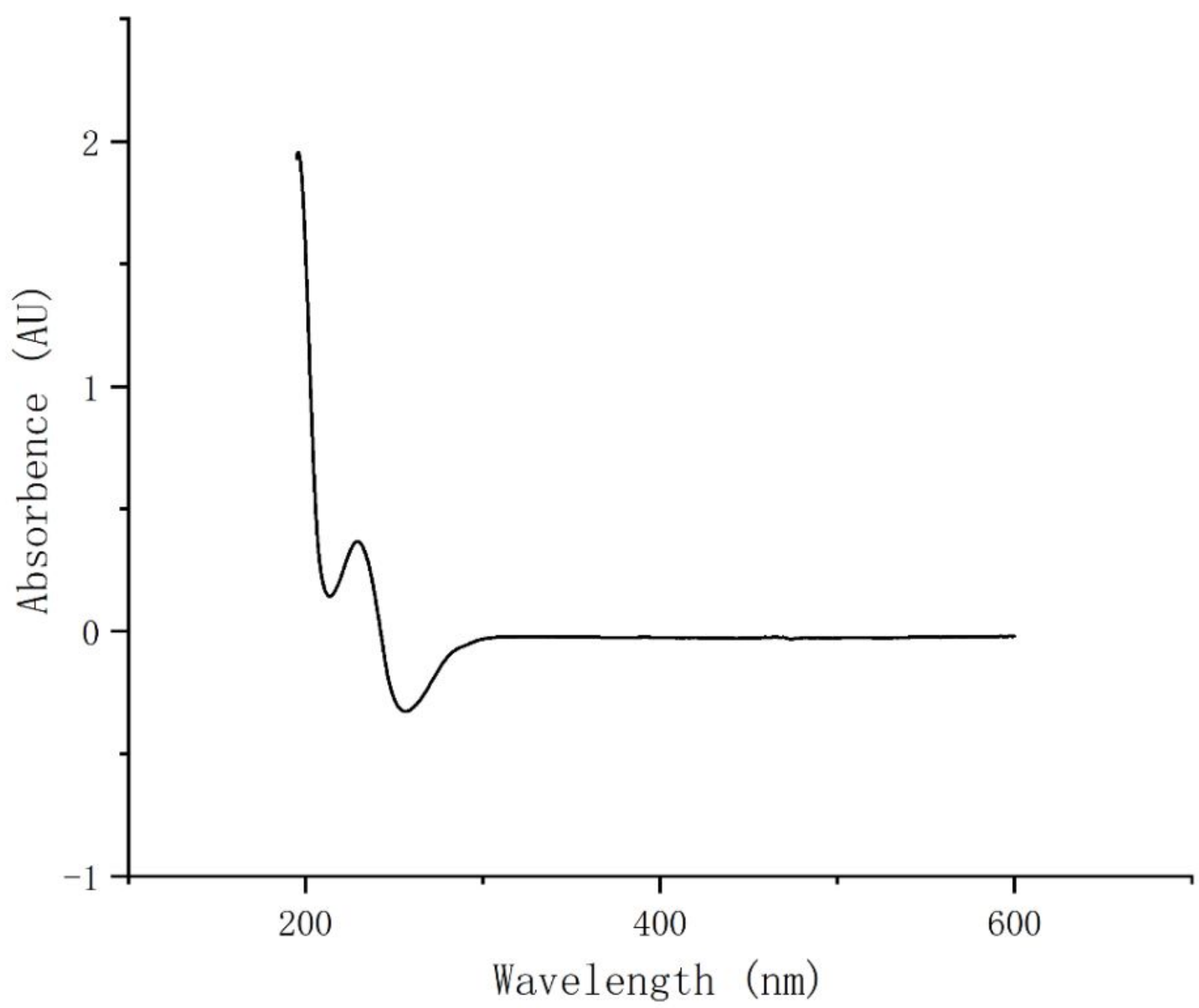

**Figure S70.** UV spectrum of **7**

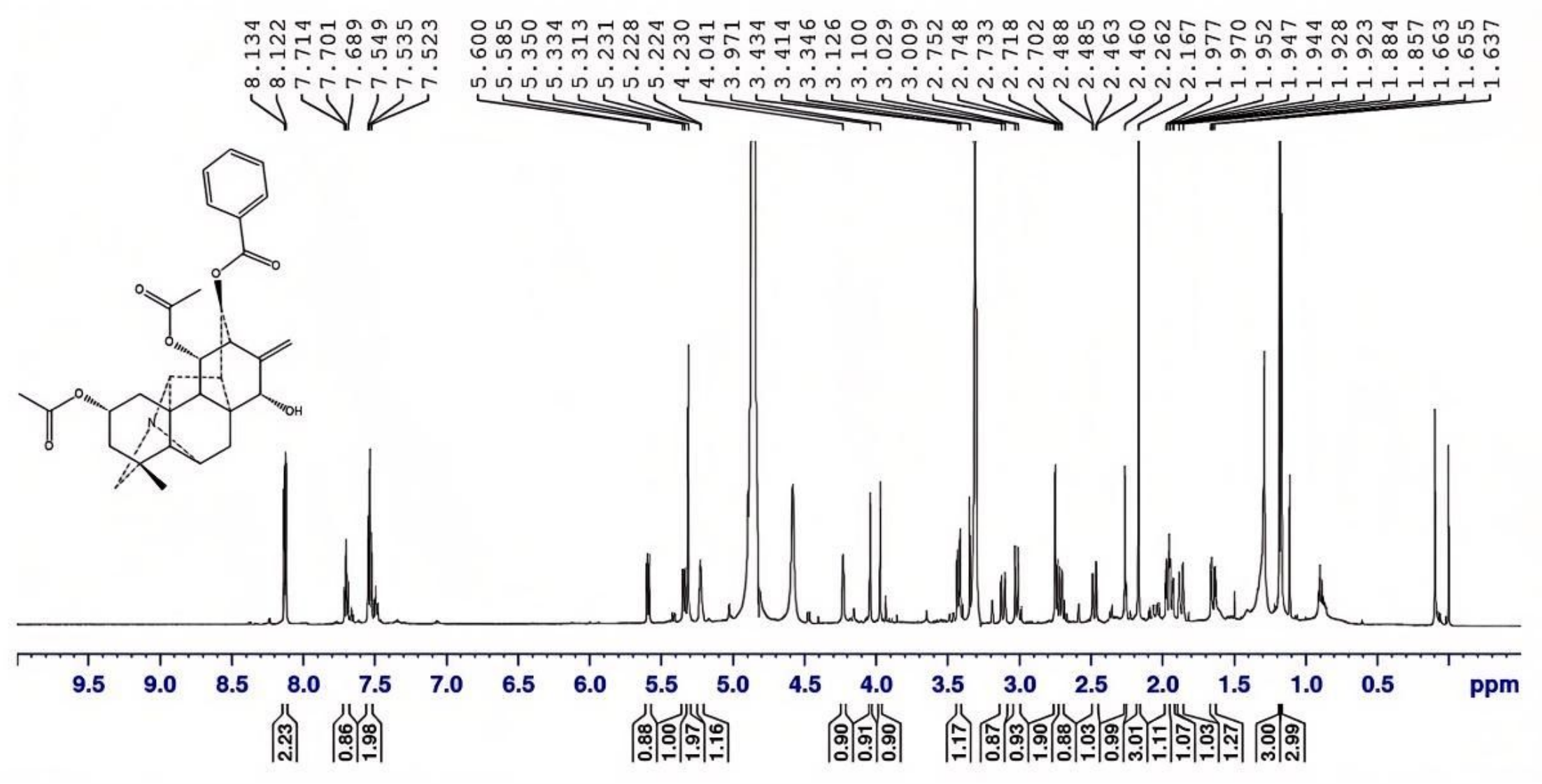

**Figure S71.** $^{1}$H NMR spectrum of **8** in methanol-$d_4$

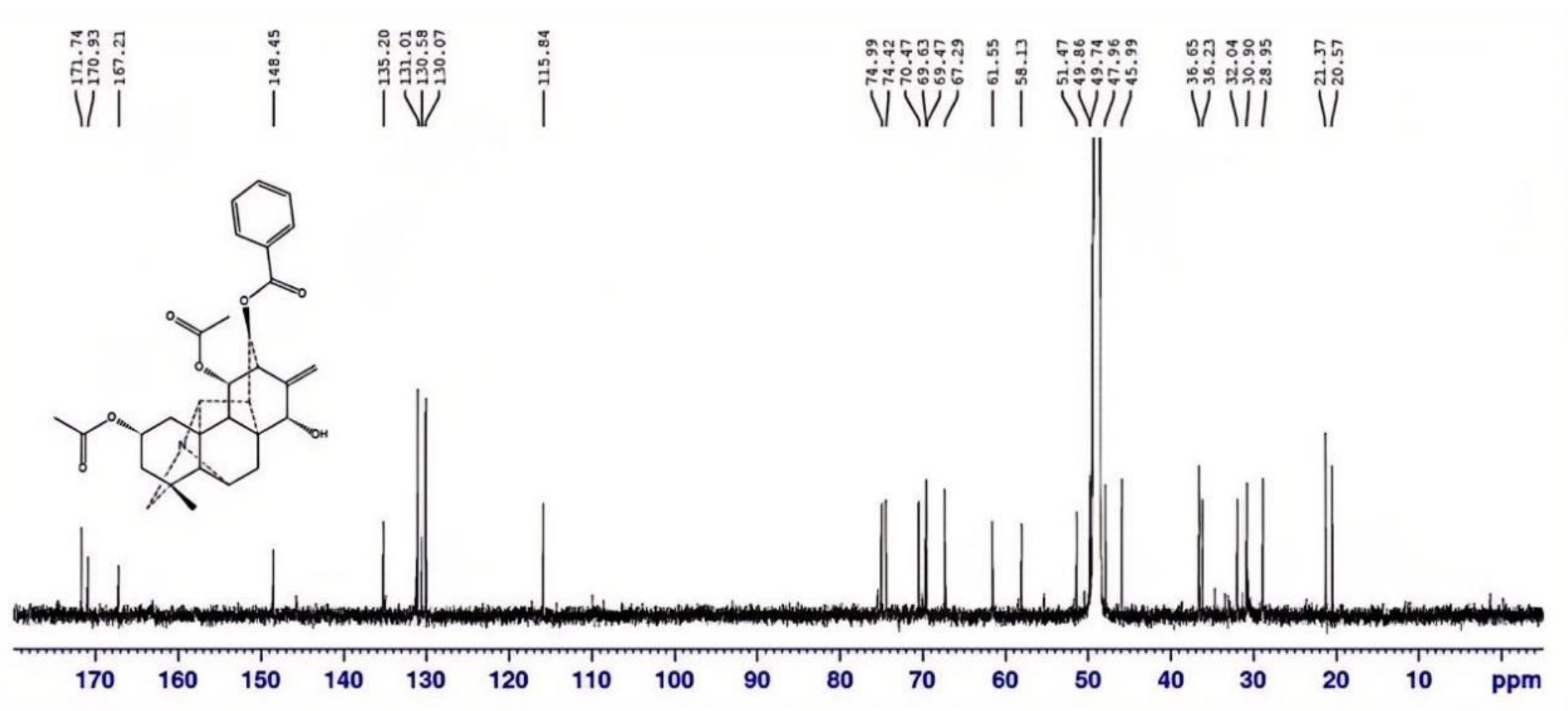

**Figure S72.** $^{13}$C NMR spectrum of **8** in methanol-$d_4$

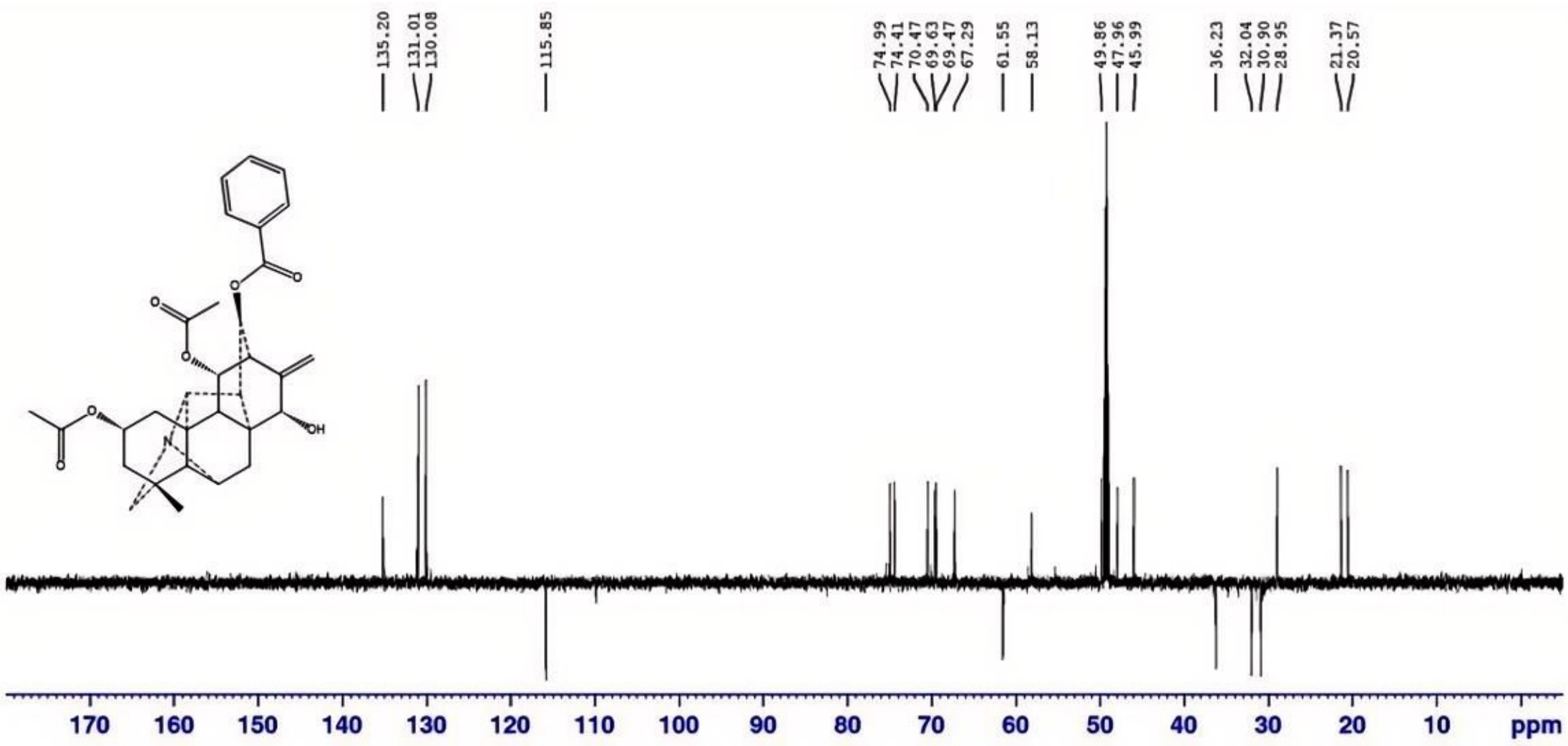


**Figure S73.** DEPT NMR spectrum of **8** in methanol-$d_4$

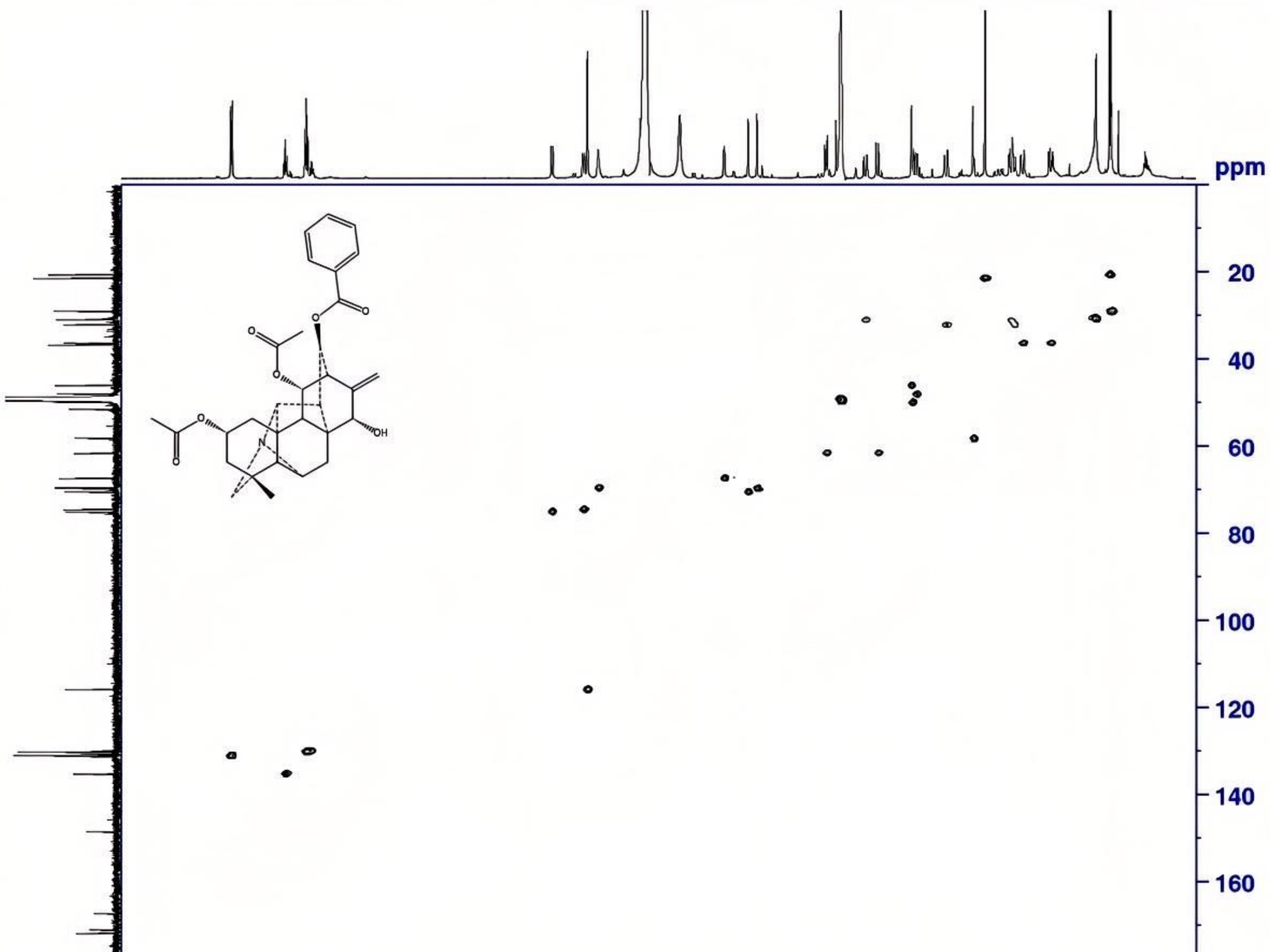


**Figure S74.** HSQC spectrum of **8**

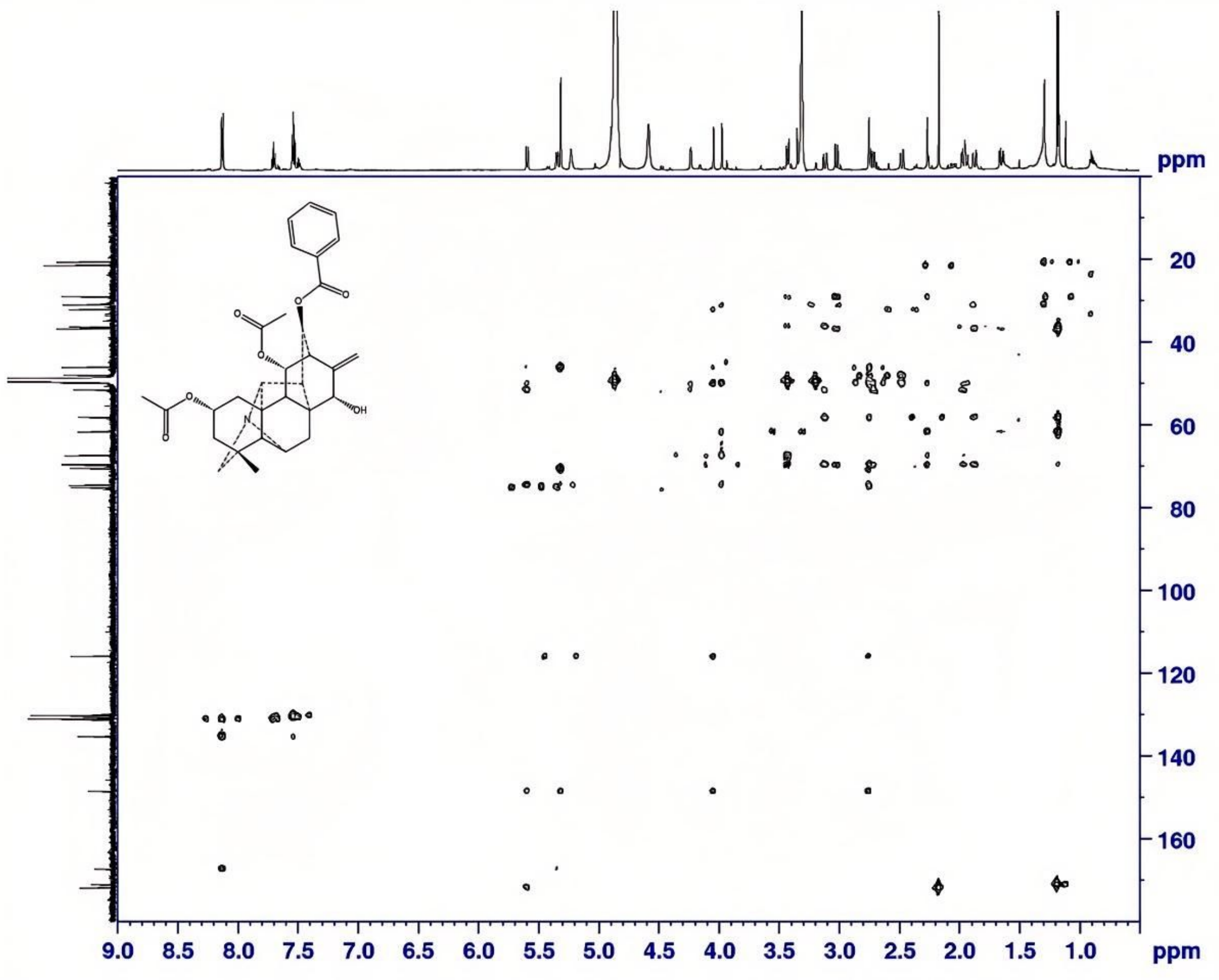


**Figure S75.** HMBC spectrum of **8**

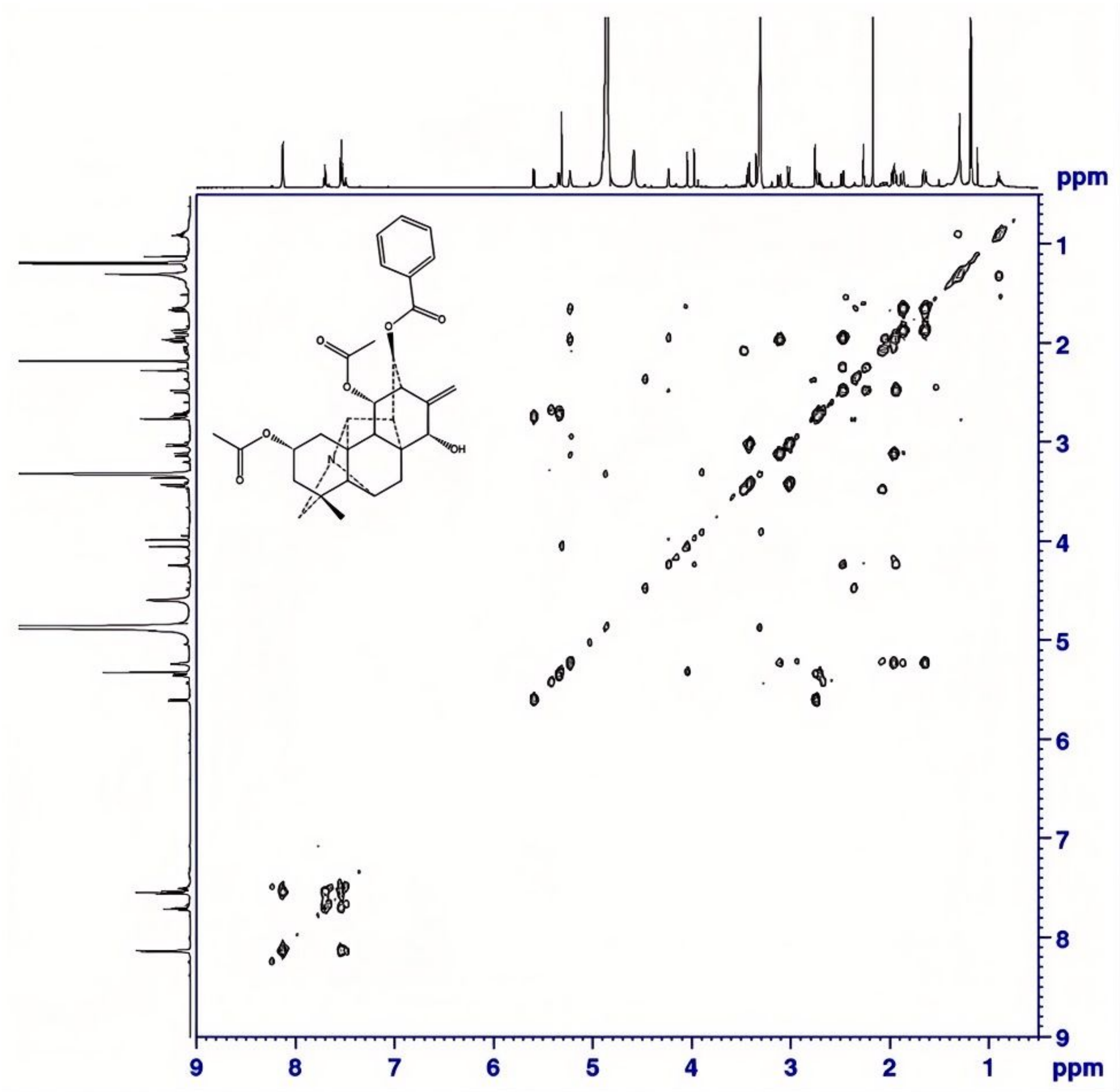


**Figure S76.** $^{1}$H-$^{1}$H COSY spectrum of **8**

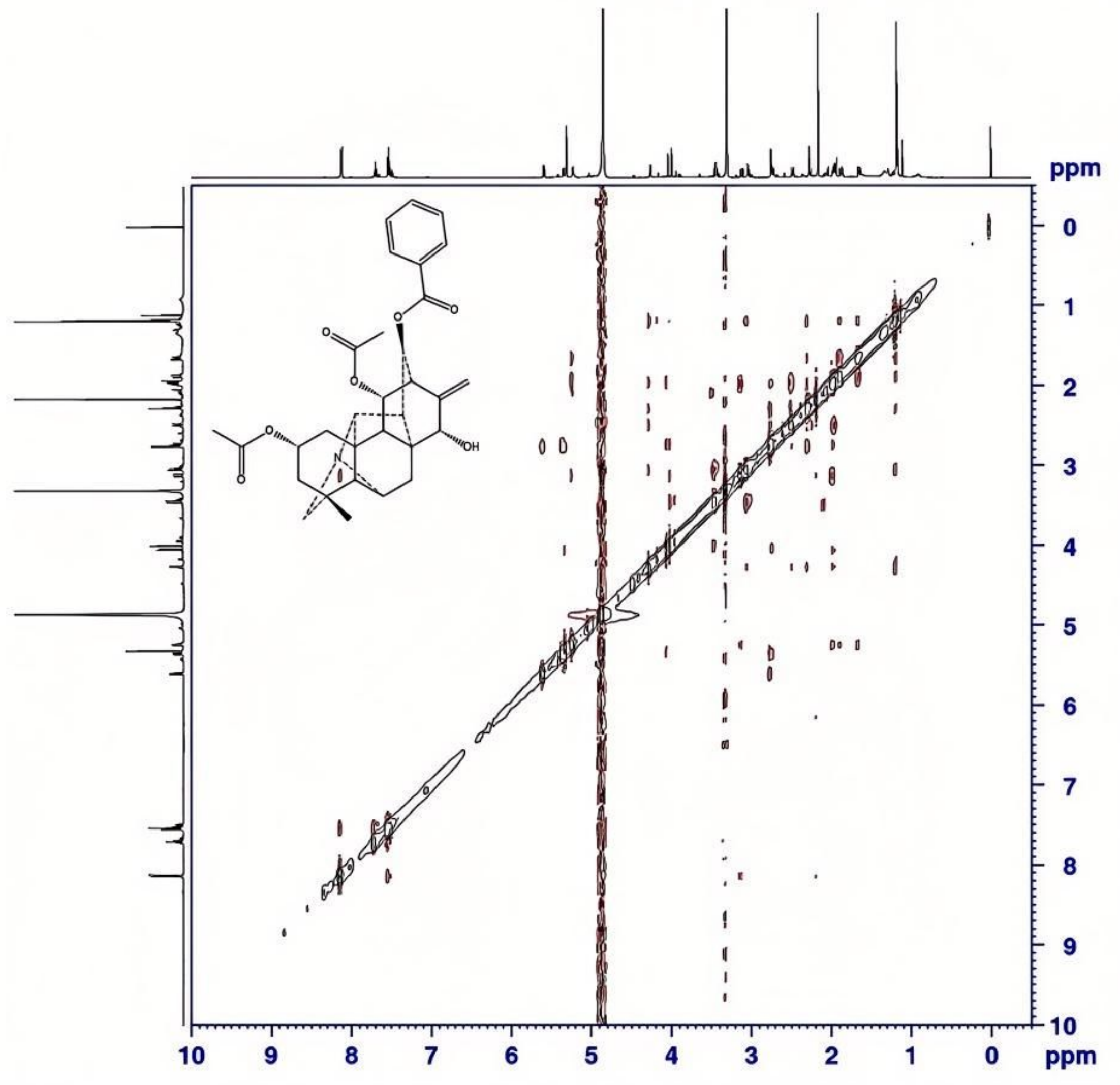

**Figure S77.** NOESY spectrum of **8**

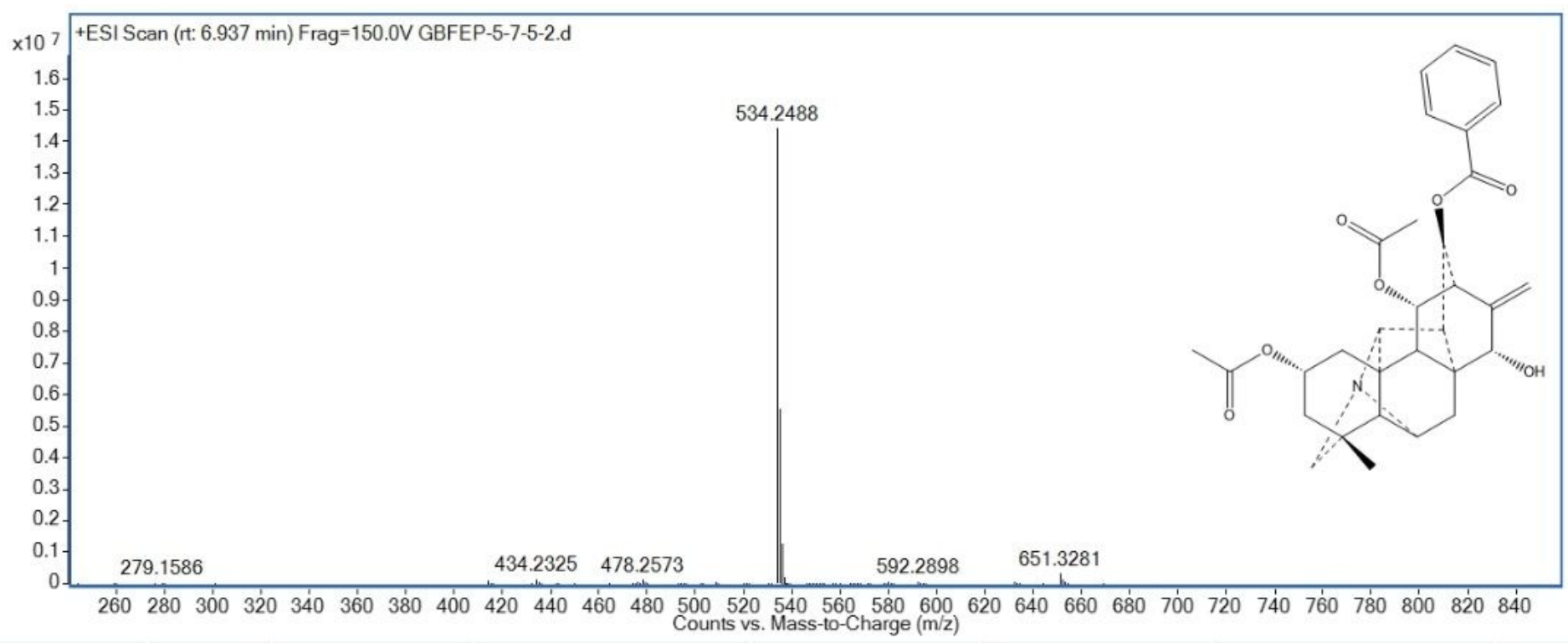


| Formula | Score | Mass | Mass (MFG) | m/z (Calc) | Diff (ppm) | Ion Formula | m/z |
|---|---|---|---|---|---|---|---|
| $C_{31}H_{35}NO_7$ | 99.94 | 533.2415 | 533.2414 | 534.2486 | -0.32 | $C_{31}H_{36}NO_7$ | 534.2488 |

**Figure S78.** HRESIMS spectrum of **8**

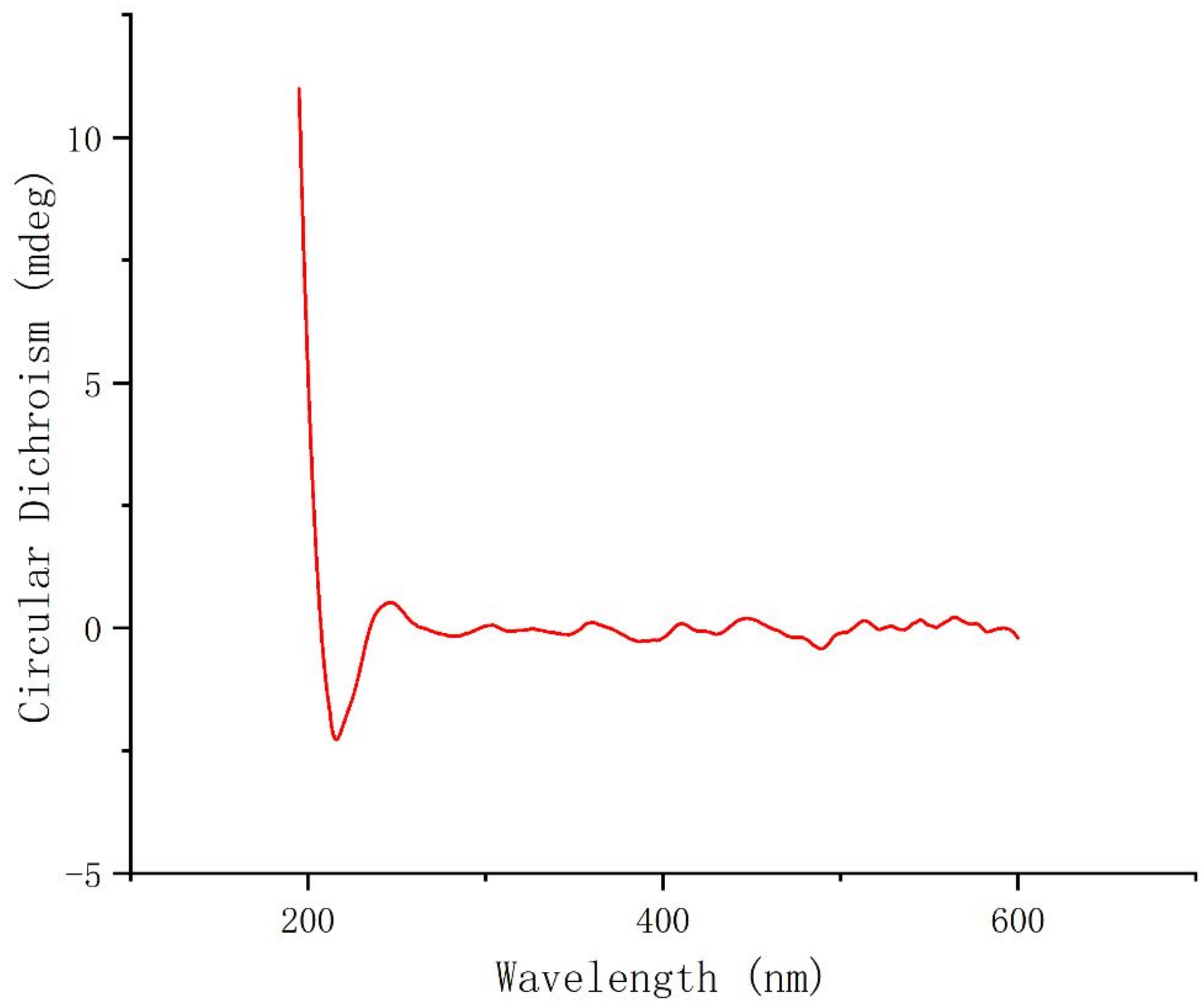


**Figure S79.** ECD spectrum of **8**

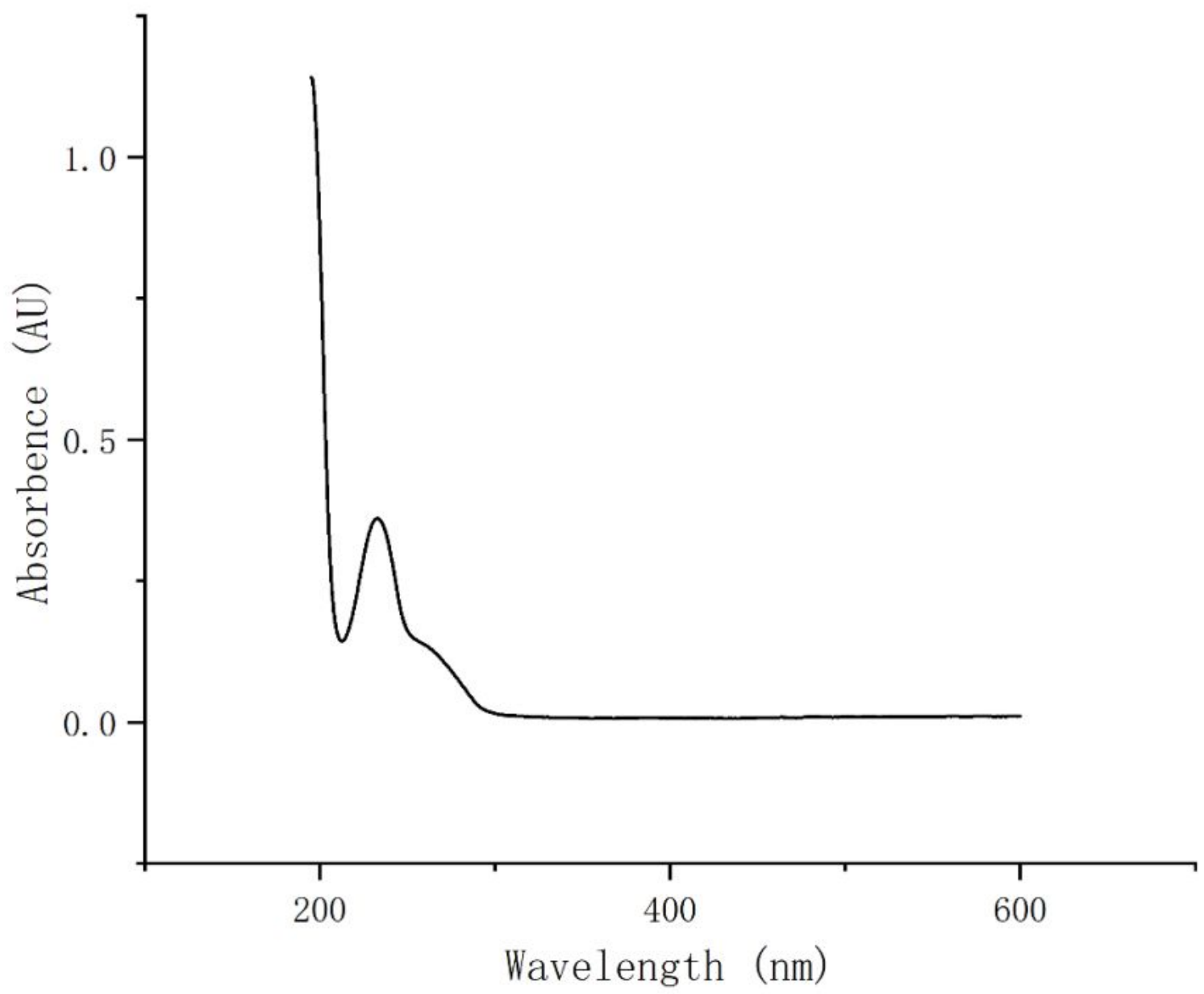


**Figure S80.** UV spectrum of **8**

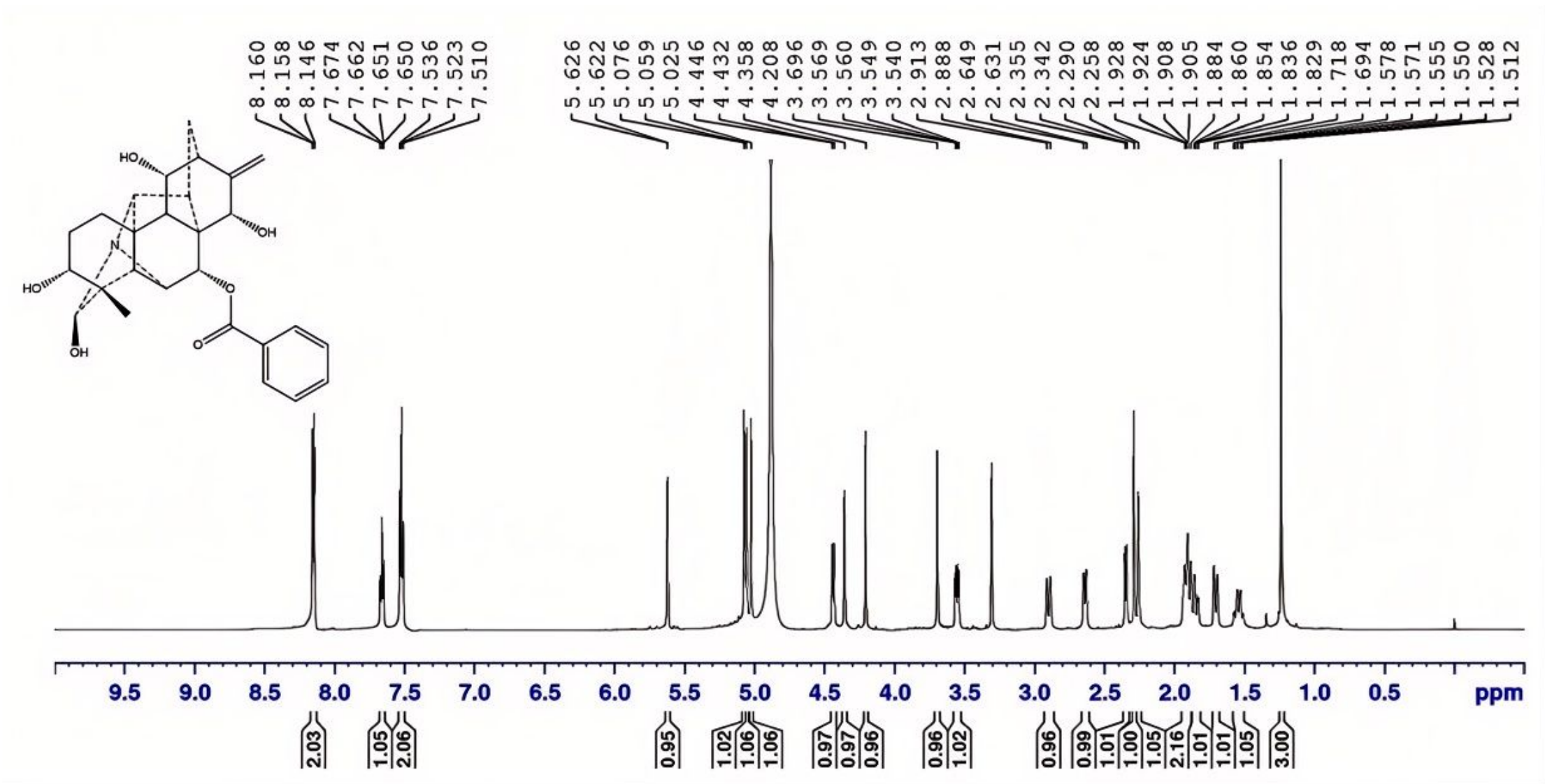

**Figure S81.** $^{1}H$ NMR spectrum of **9** in methanol-$d_4$

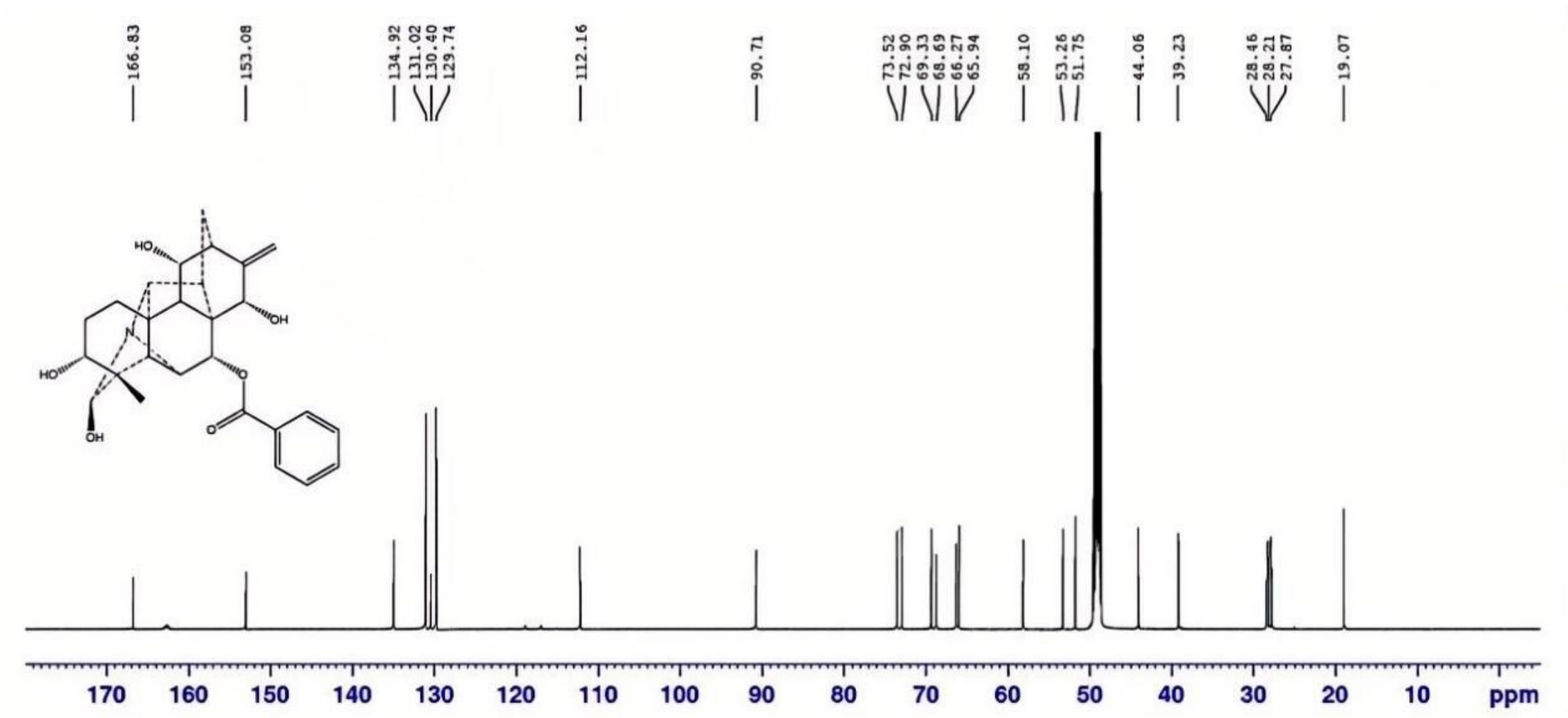

**Figure S82.** $^{13}C$ NMR spectrum of **9** in methanol-$d_4$

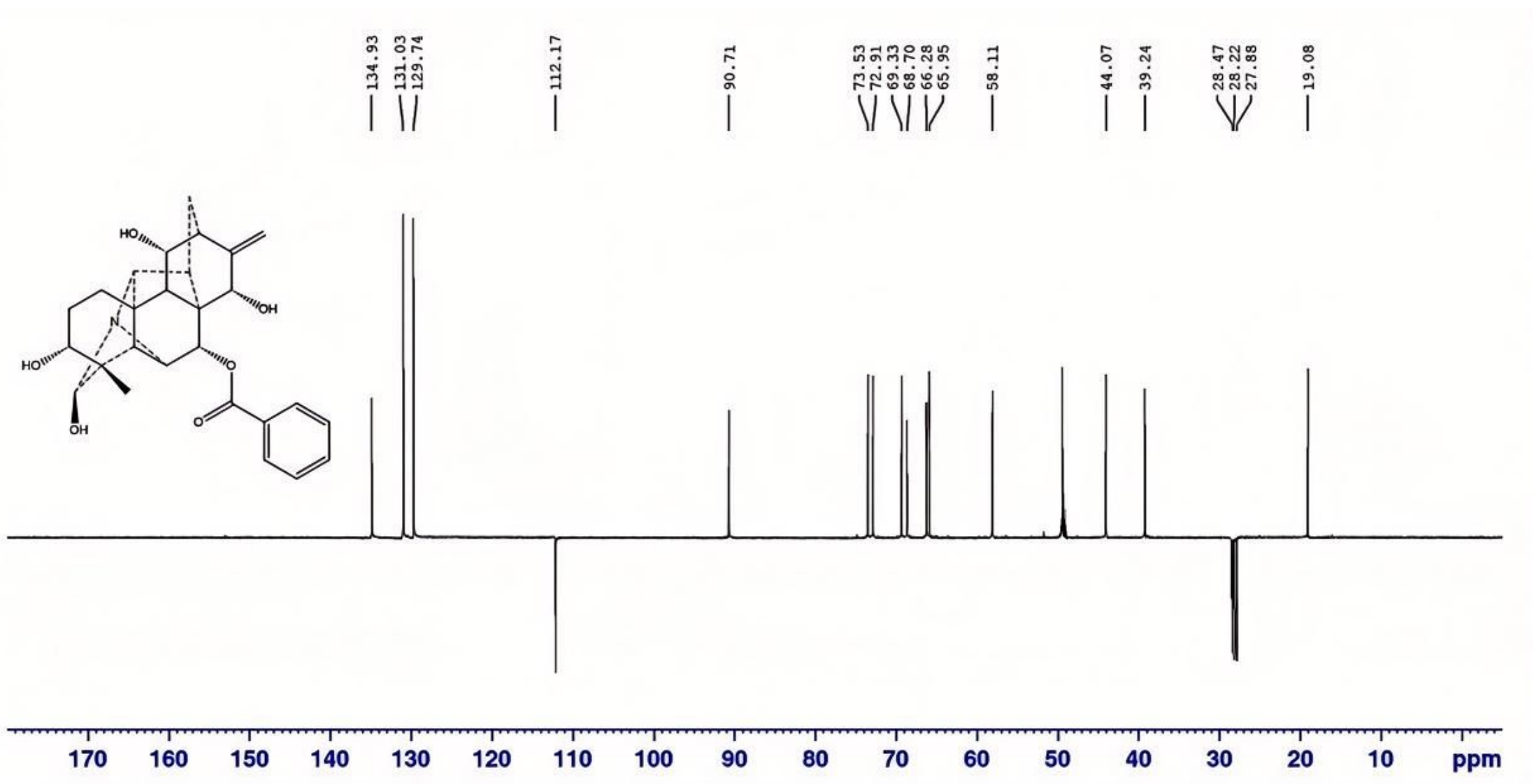


**Figure S83.** DEPT NMR spectrum of **9** in methanol-$d_4$

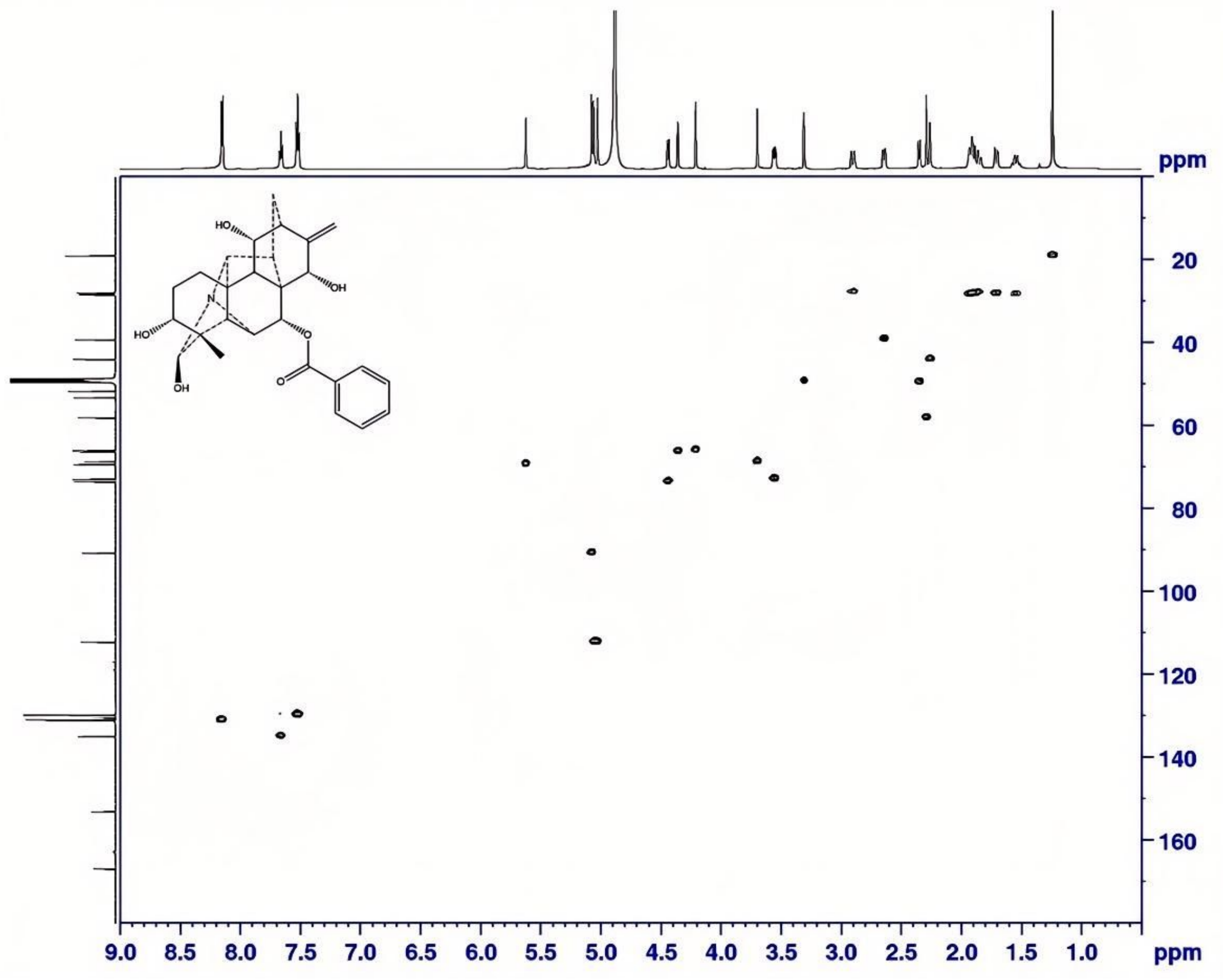


**Figure S84.** HSQC spectrum of **9**

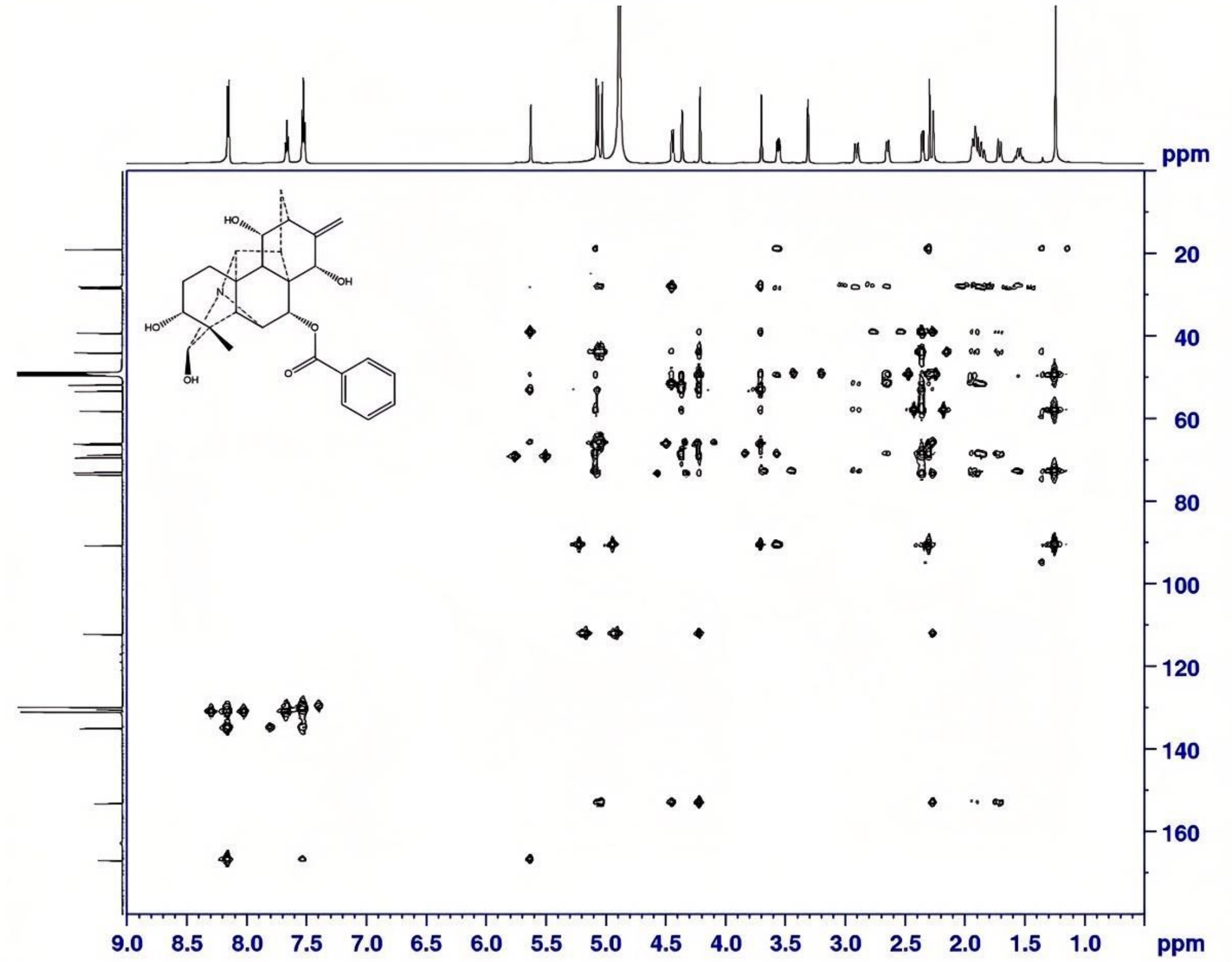


**Figure S85.** HMBC spectrum of **9**

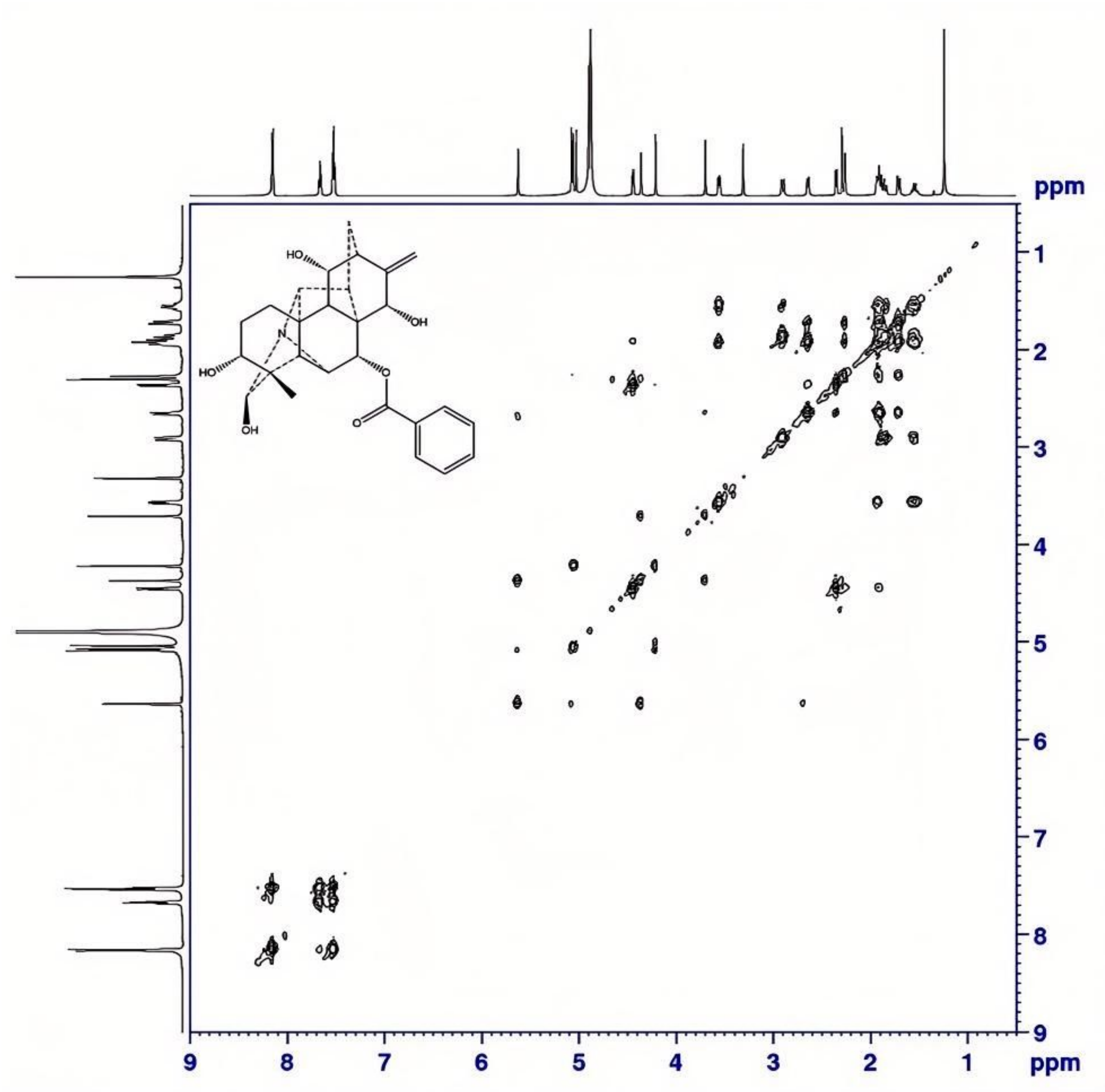


**Figure S86.** $^{1}H$-$^{1}H$ COSY spectrum of **9**

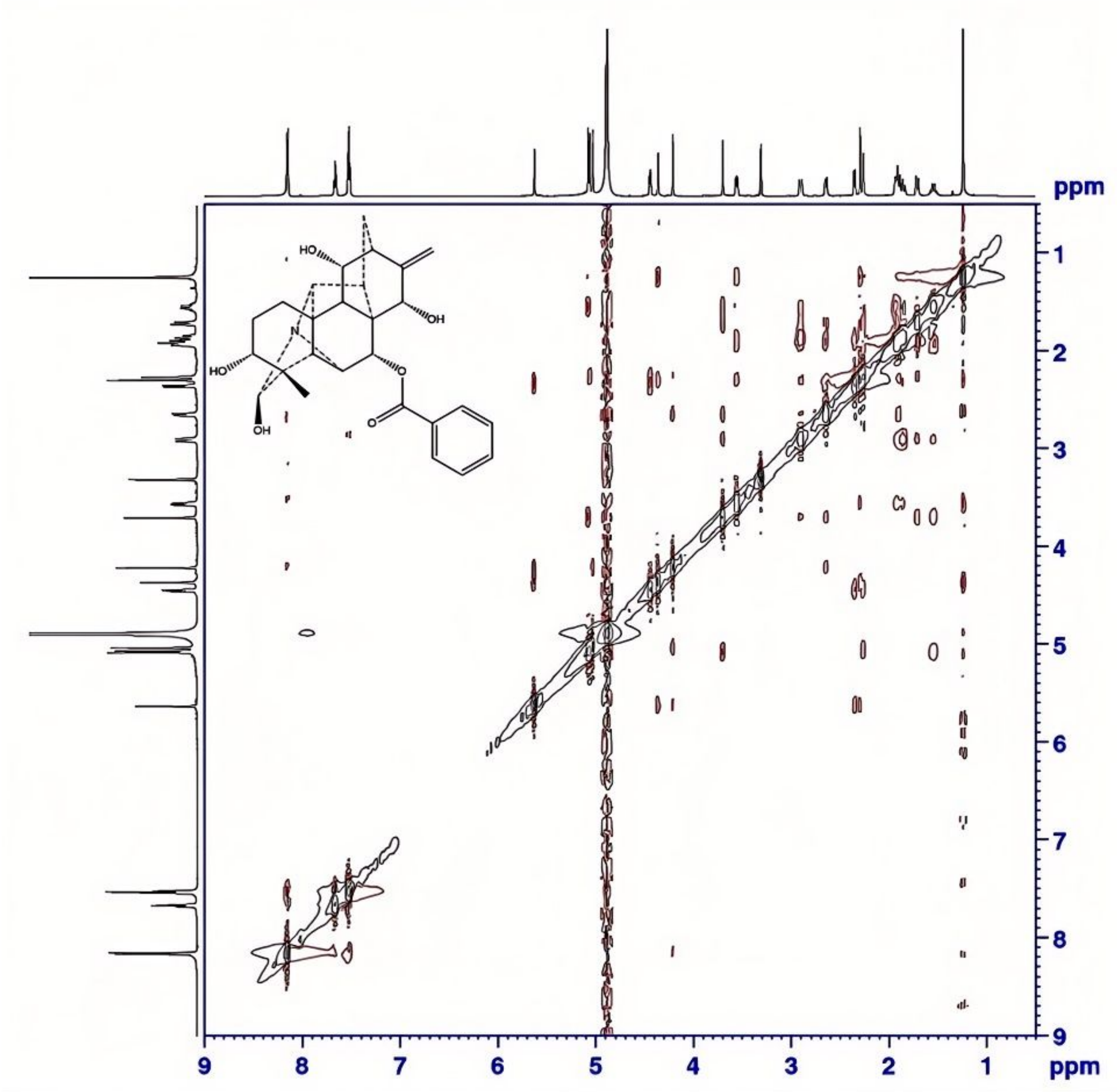

**Figure S87.** NOESY spectrum of **9**

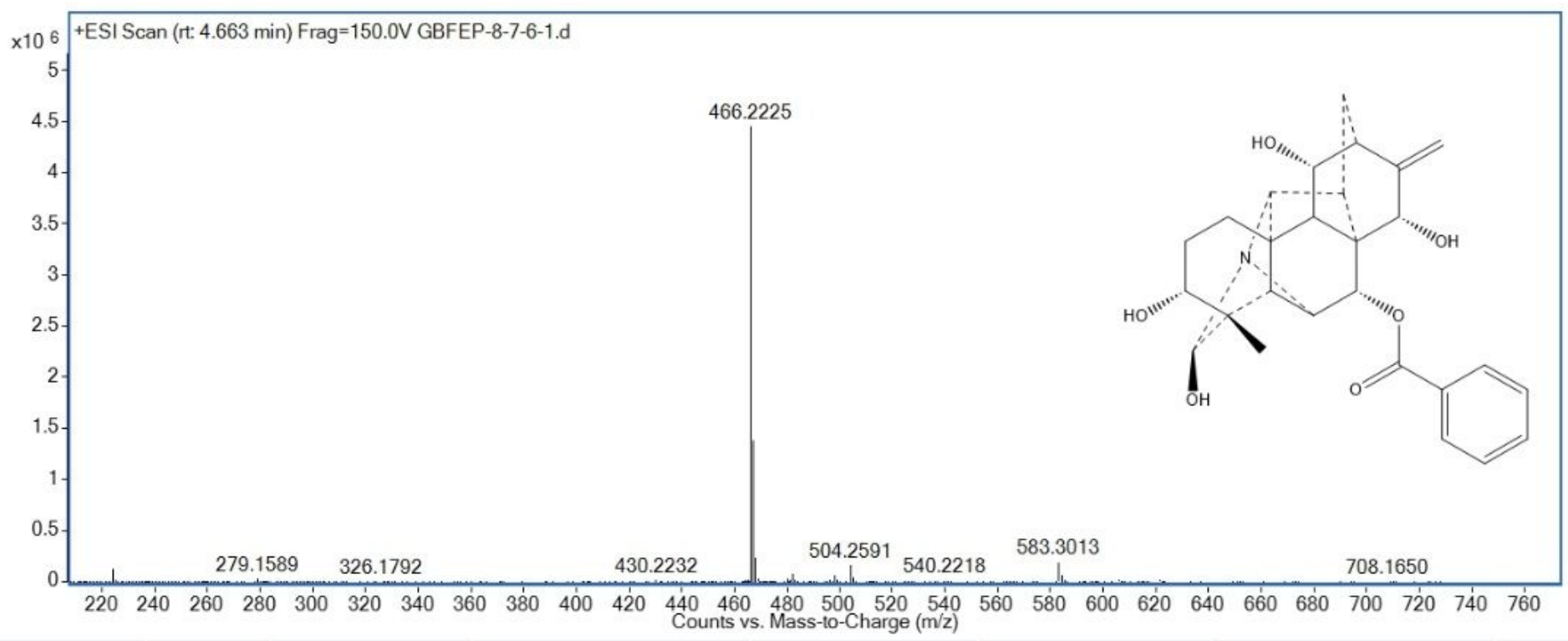

| Formula | Score | Mass | Mass (MFG) | m/z (Calc) | Diff (ppm) | Ion Formula | m/z |
|---|---|---|---|---|---|---|---|
| $C_{27}H_{31}NO_6$ | 99.98 | 465.2152 | 465.2151 | 466.2224 | -0.18 | $C_{27}H_{32}NO_6$ | 466.2225 |

**Figure S88.** HRESIMS spectrum of **9**

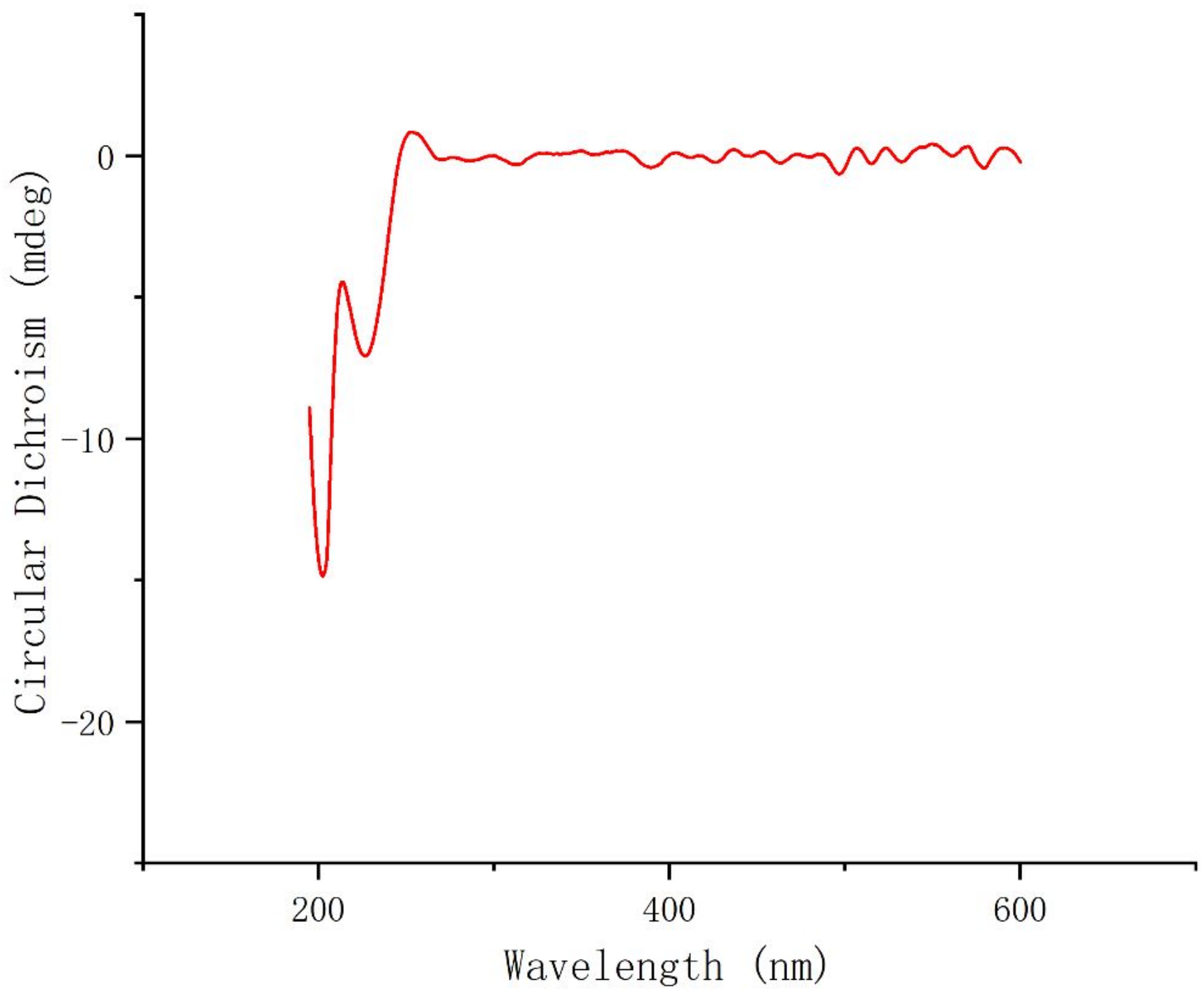


**Figure S89.** ECD spectrum of **9**

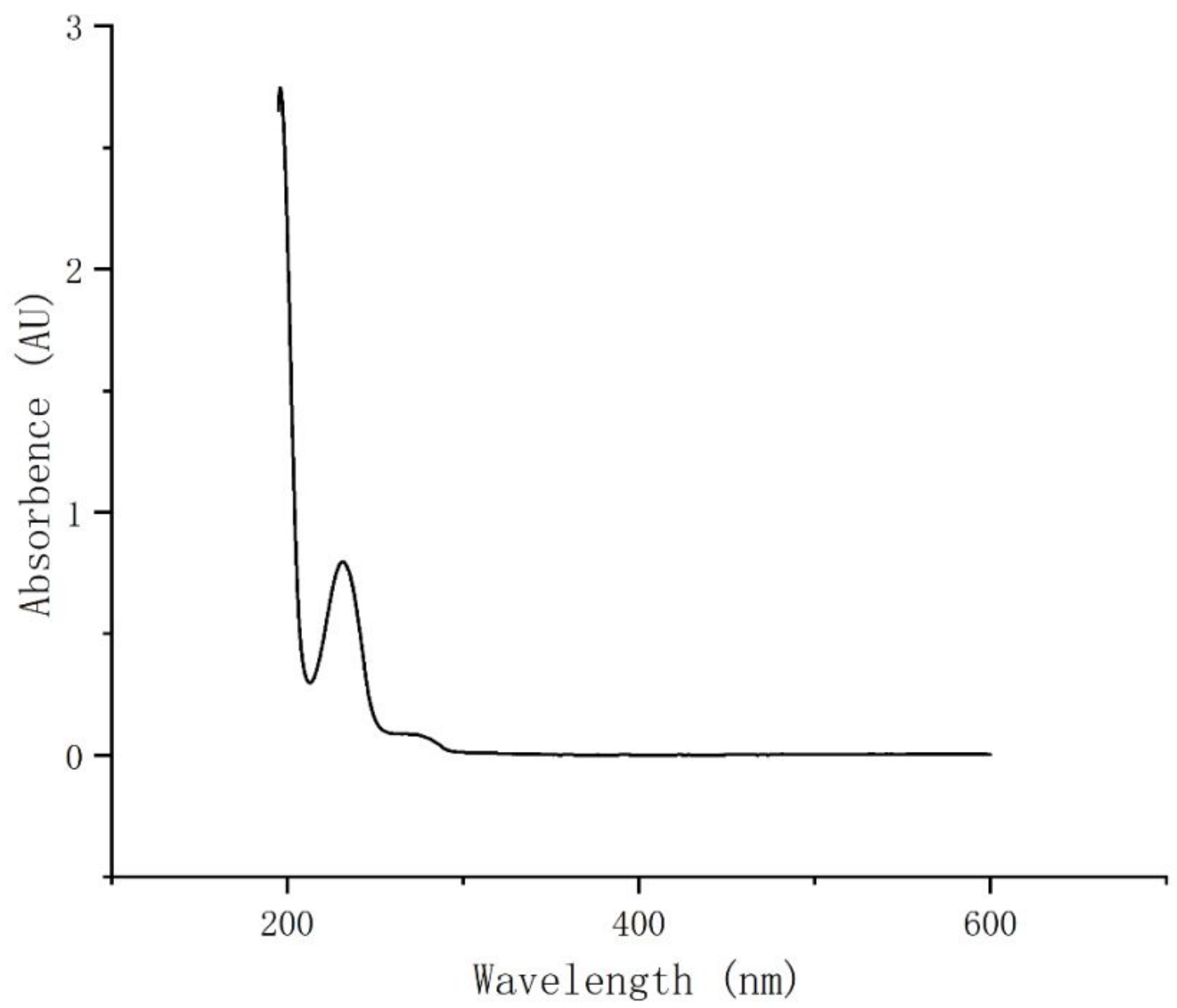


**Figure S90.** UV spectrum of **9**

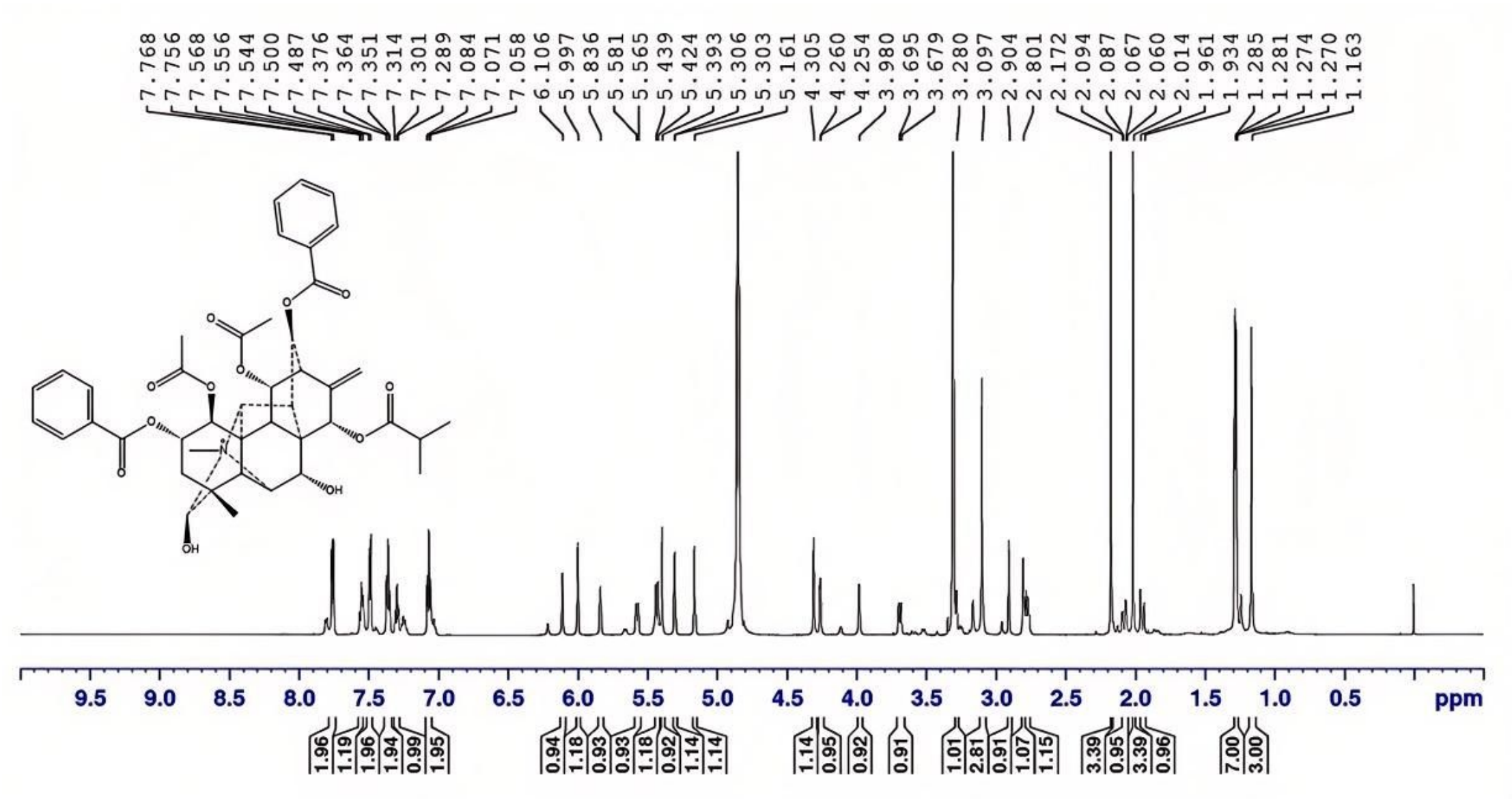


**Figure S91.** $^{1}H$ NMR spectrum of **10** in methanol-$d_4$

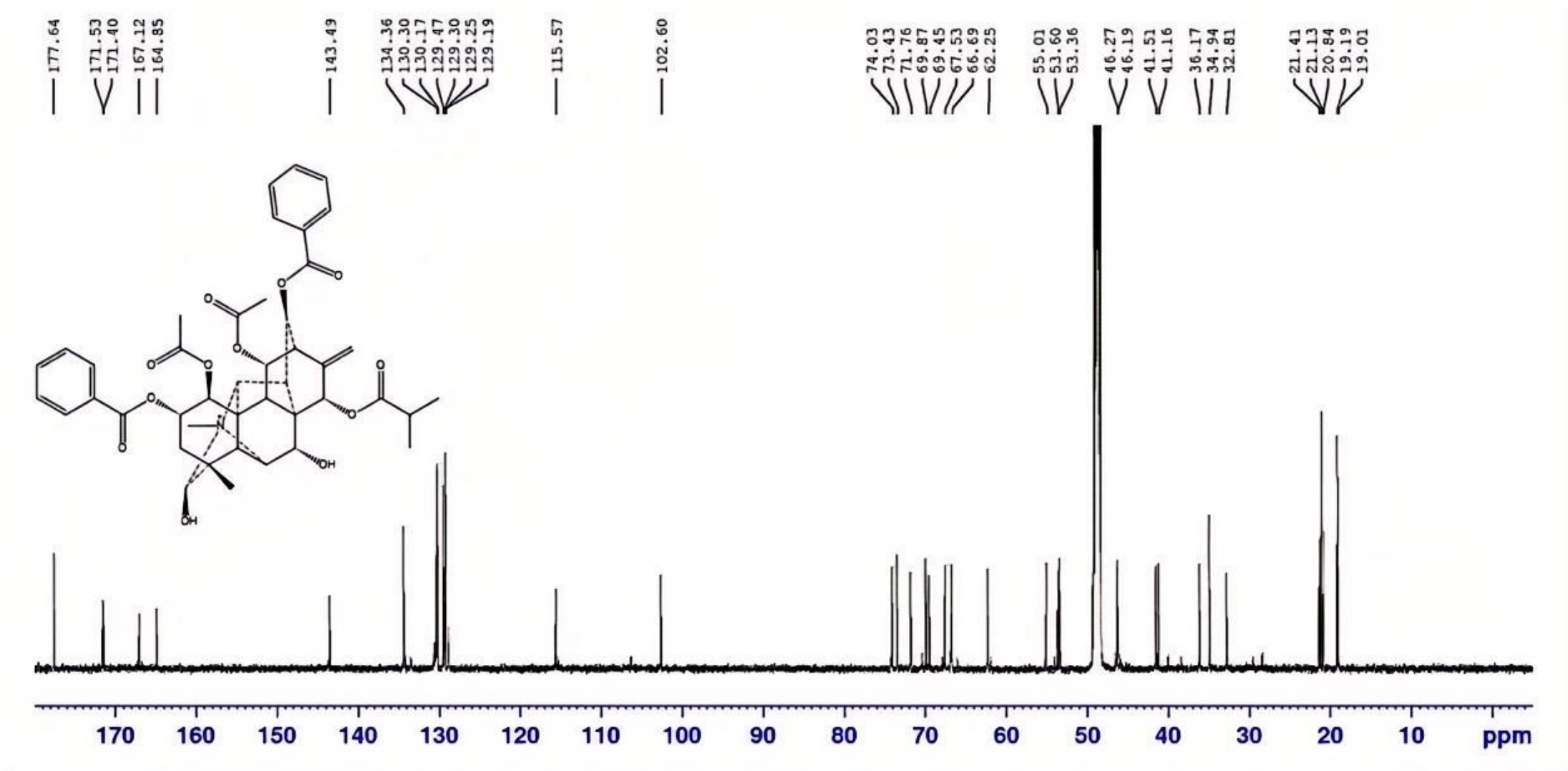


**Figure S92.** $^{13}C$ NMR spectrum of **10** in methanol-$d_4$

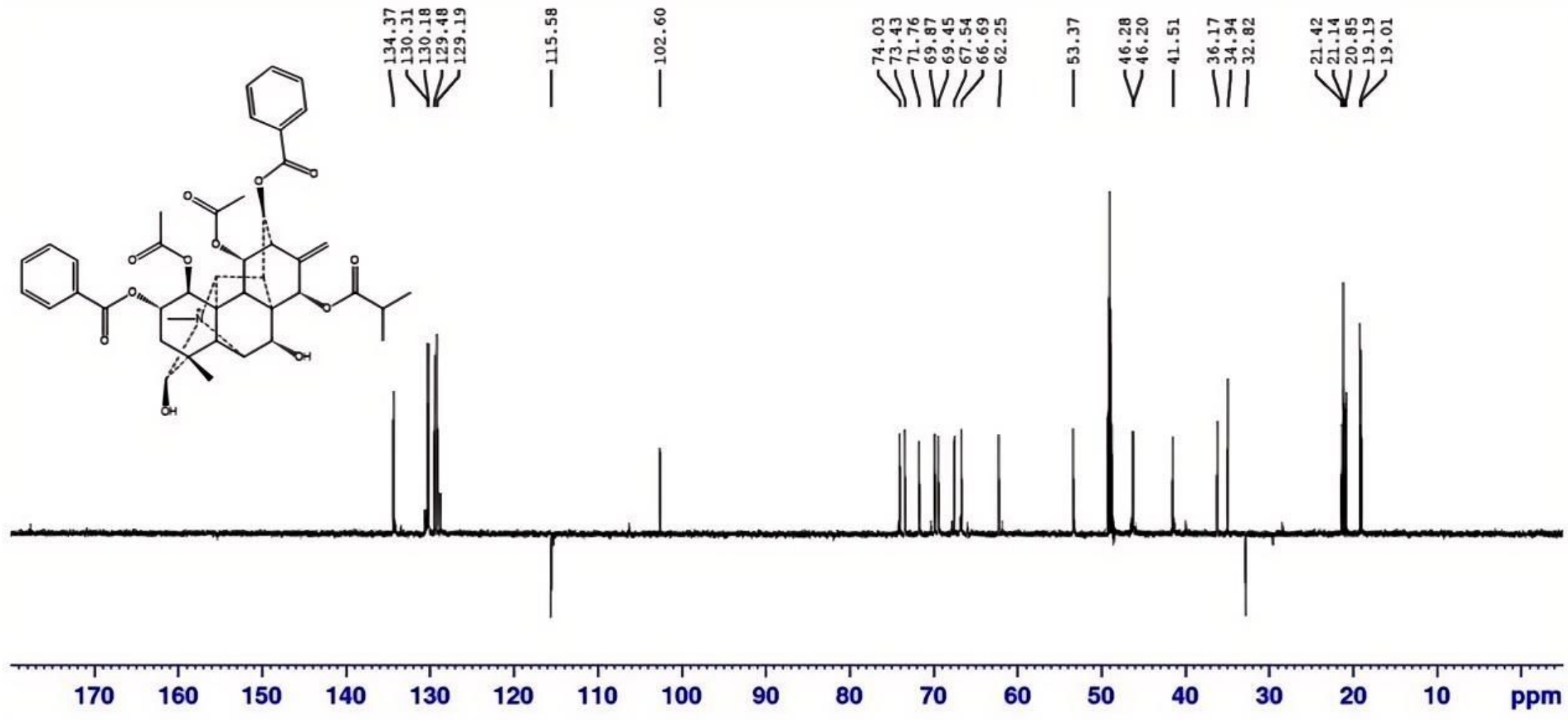


**Figure S93.** DEPT NMR spectrum of **10** in methanol-$d_4$

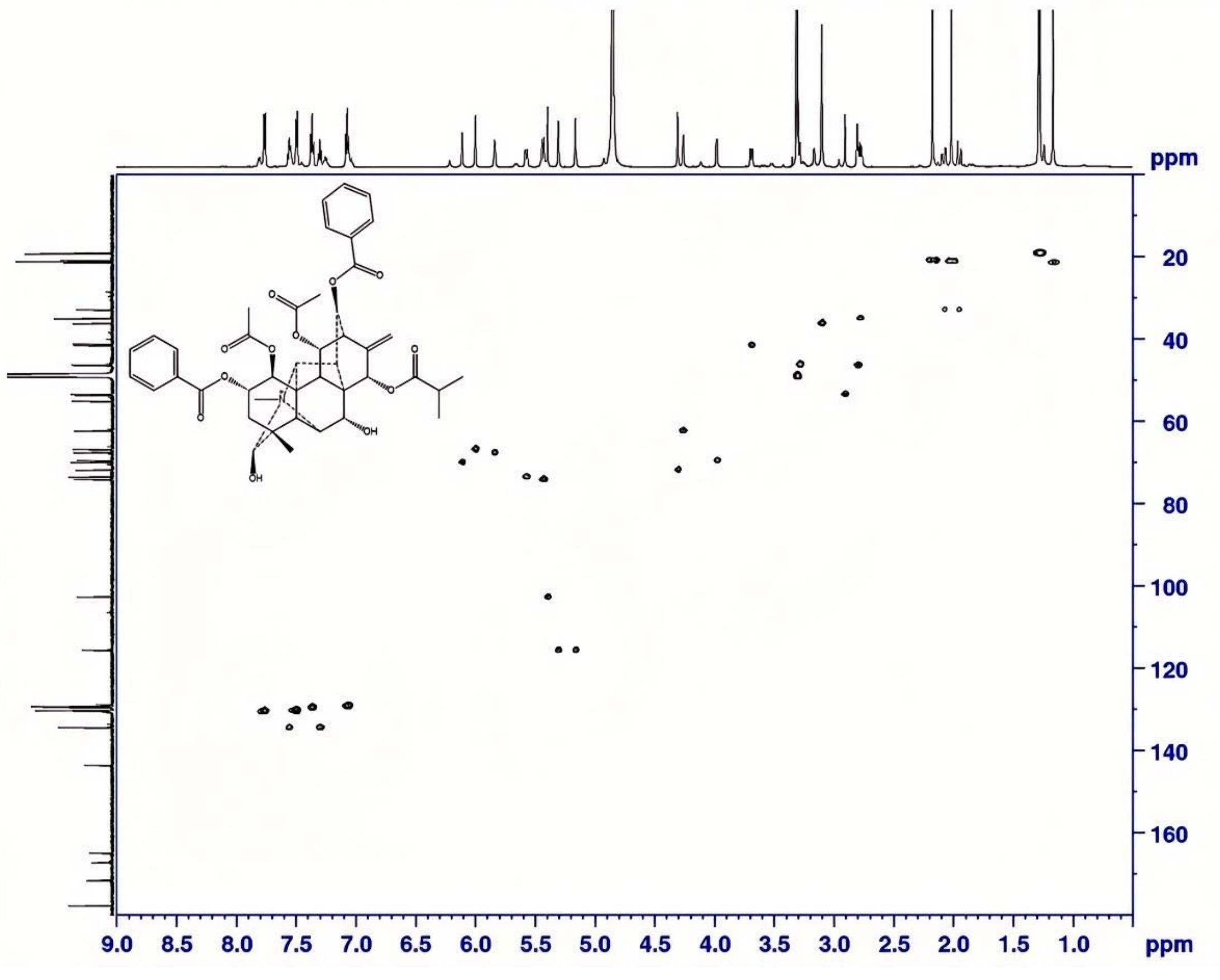


**Figure S94.** HSQC spectrum of **10**

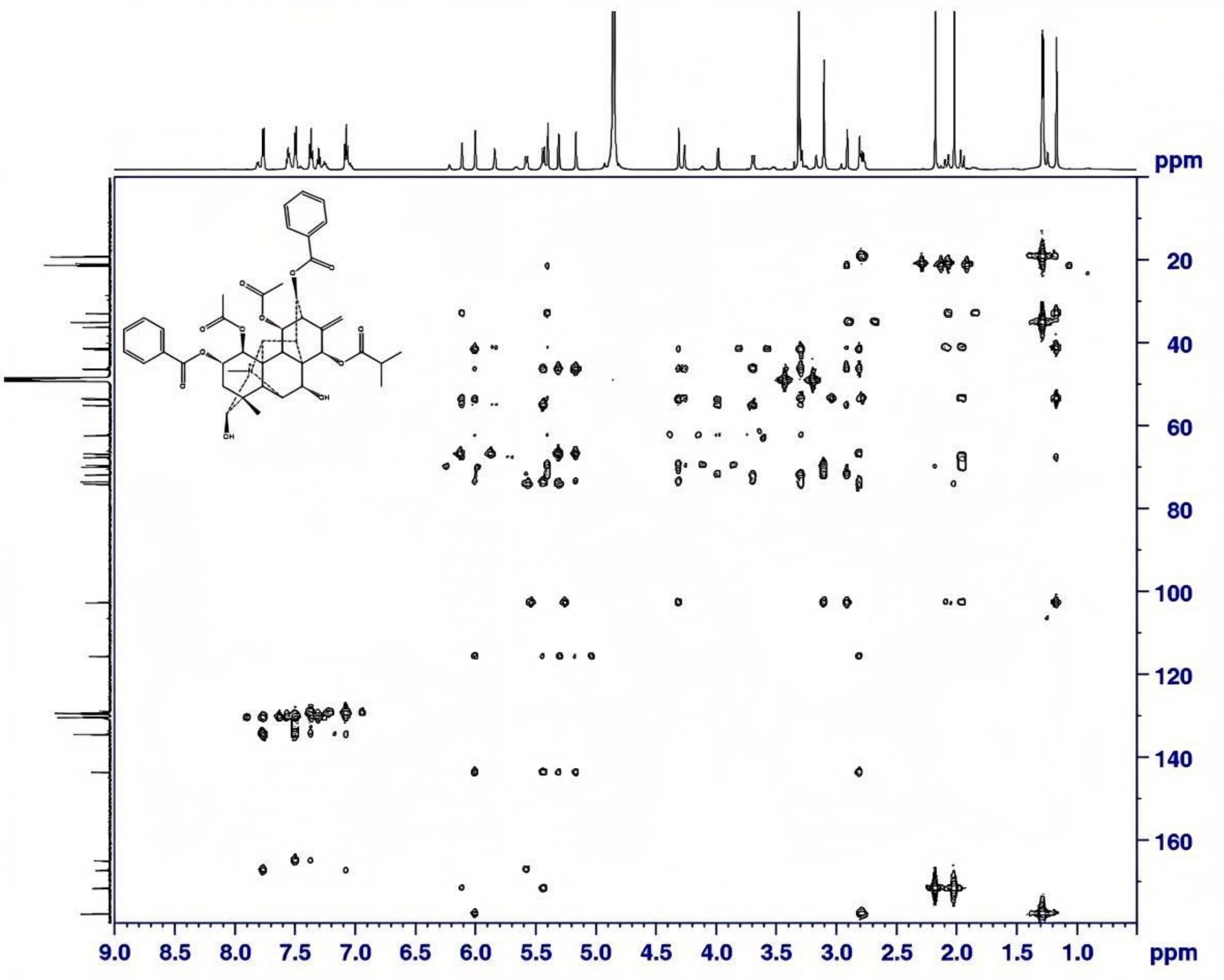


**Figure S95.** HMBC spectrum of **10**

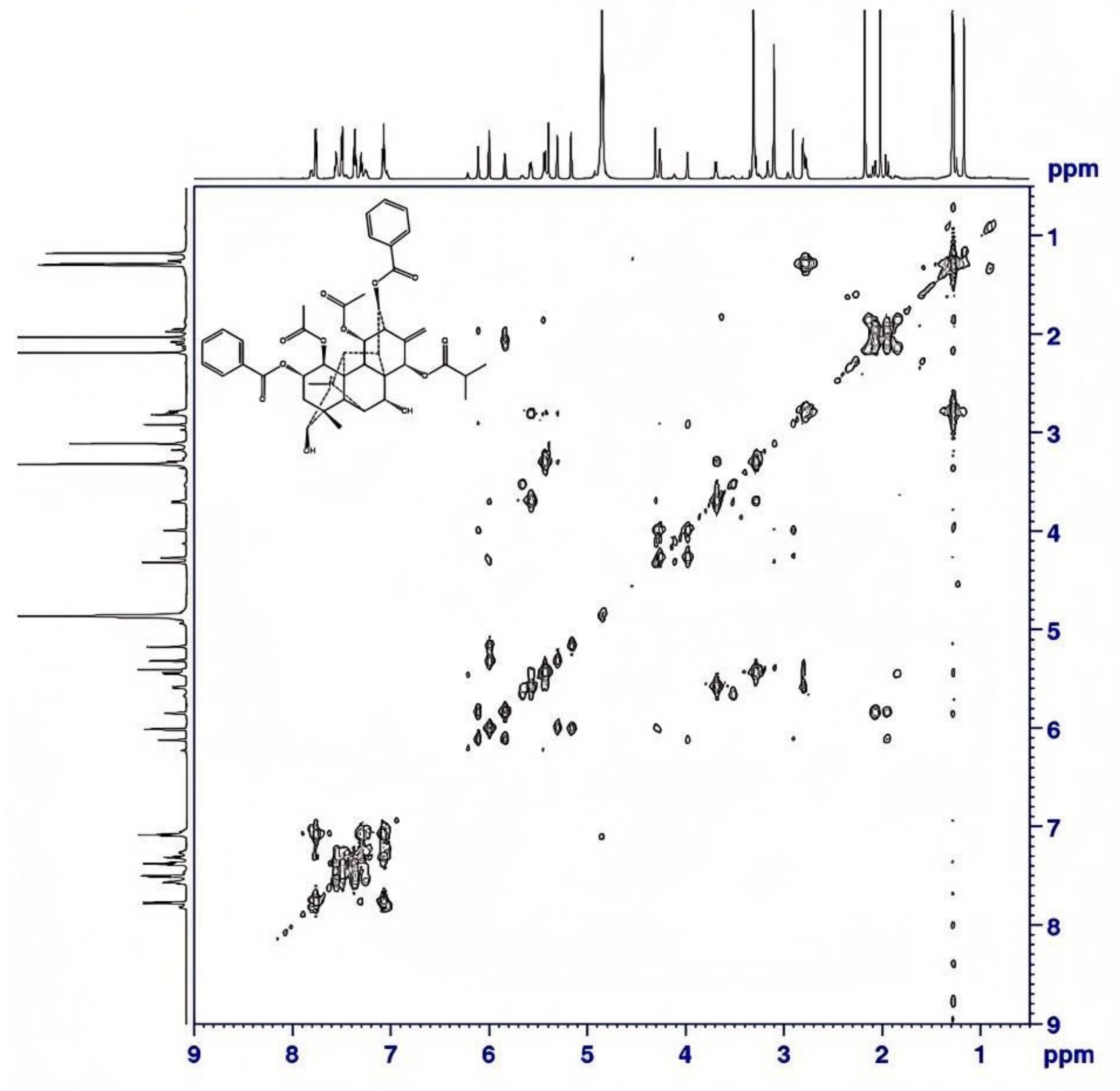


**Figure S96.** $^1H$-$^1H$ COSY spectrum of **10**

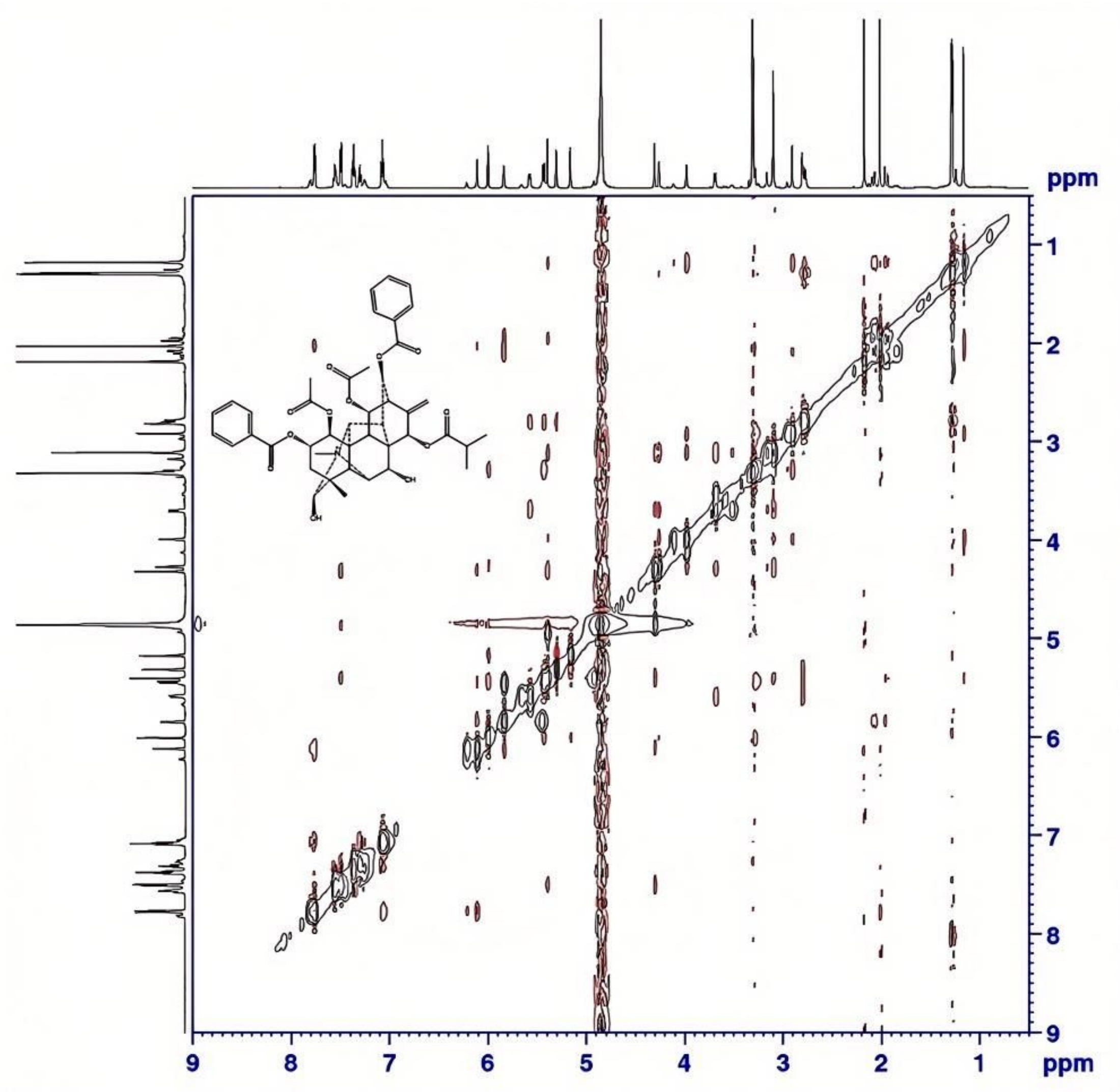


**Figure S97.** NOESY spectrum of **10**

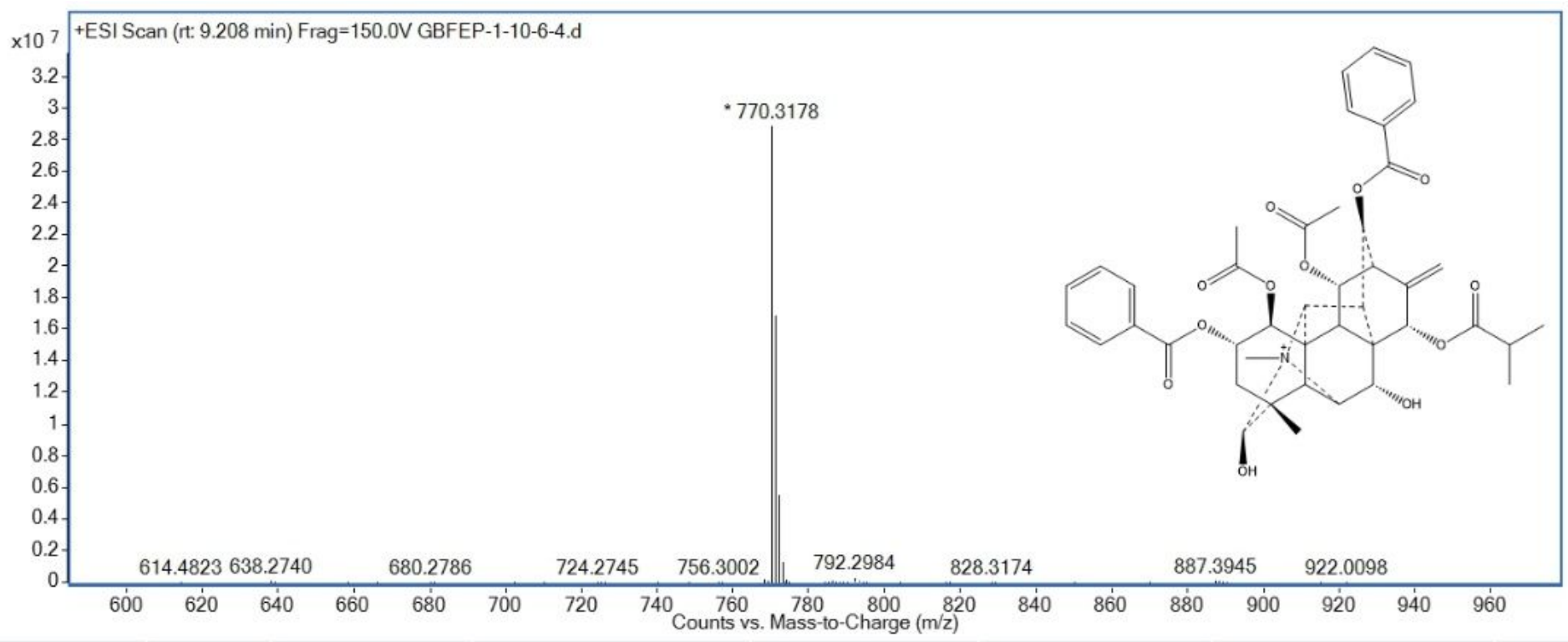


| Formula | Score | Mass | Mass (MFG) | m/z (Calc) | Diff (ppm) | Ion Formula | m/z |
|---|---|---|---|---|---|---|---|
| $C_{43}H_{48}NO_{12}$ | 99.42 | 770.3183 | 770.3177 | 770.3171 | -0.91 | $C_{43}H_{48}NO_{12}$ | 770.3178 |

**Figure S98.** HRESIMS spectrum of **10**

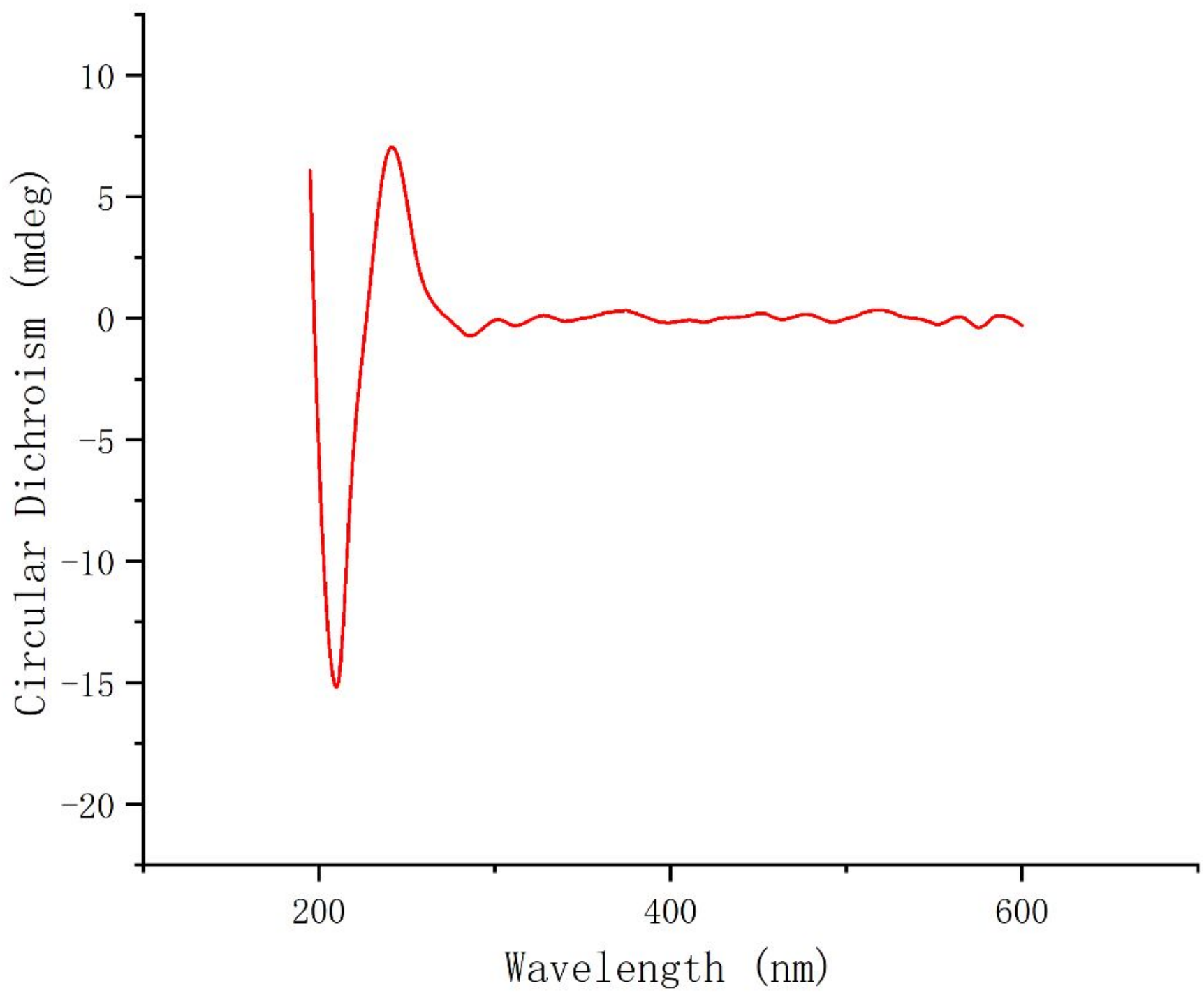


**Figure S99.** ECD spectrum of **10**

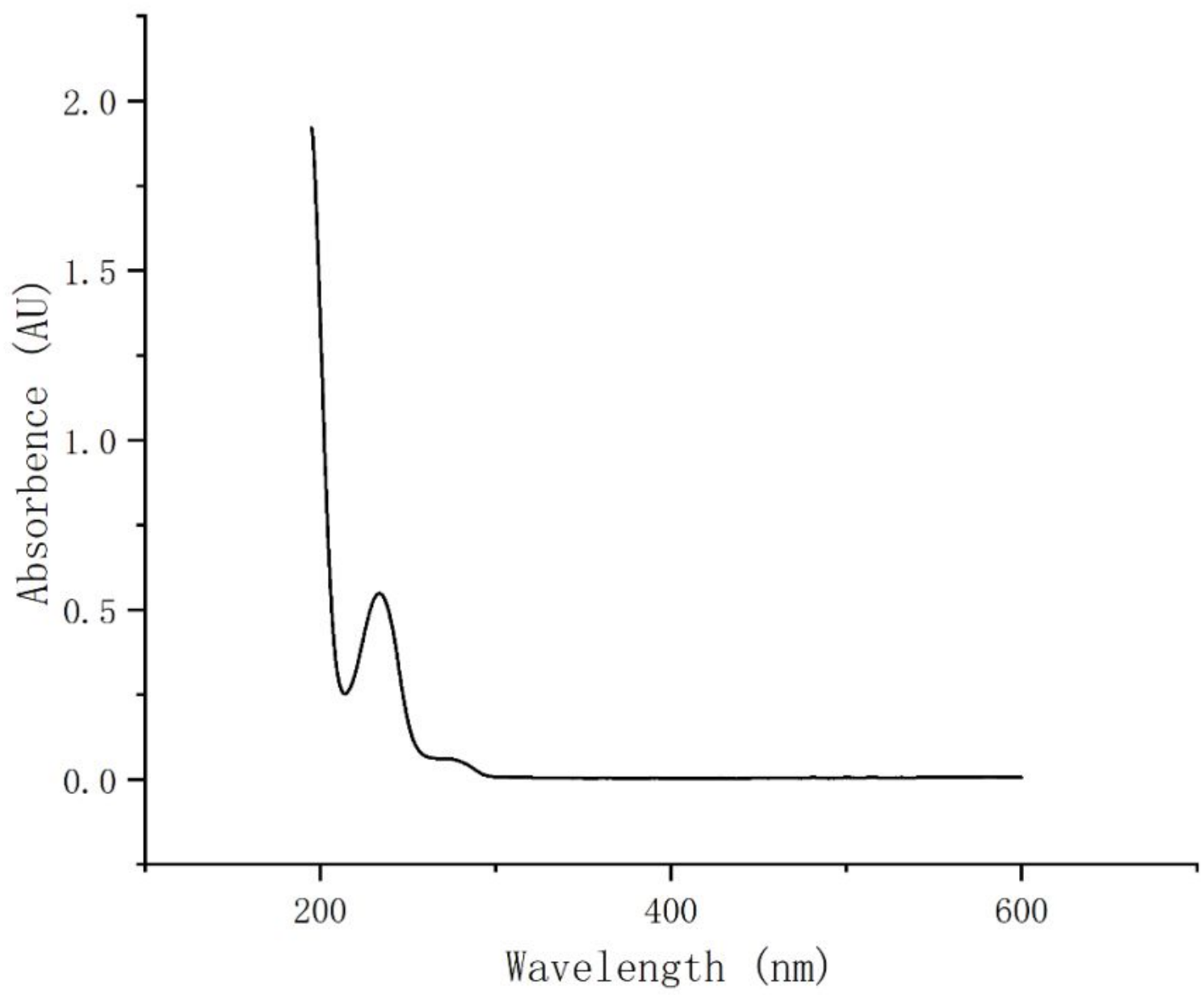


**Figure S100.** UV spectrum of **10**

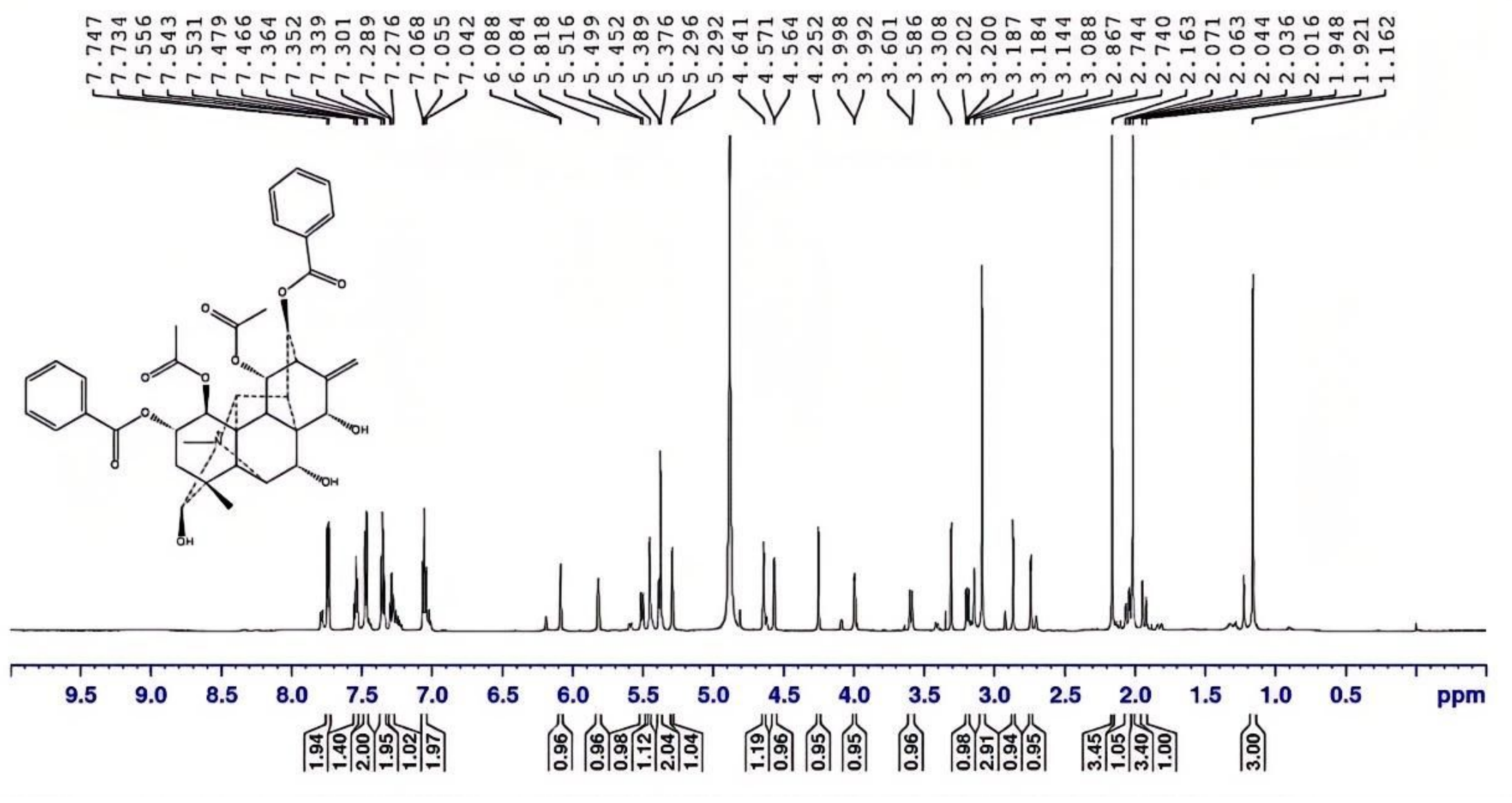

**Figure S101.** $^{1}$H NMR spectrum of **11** in methanol-$d_4$

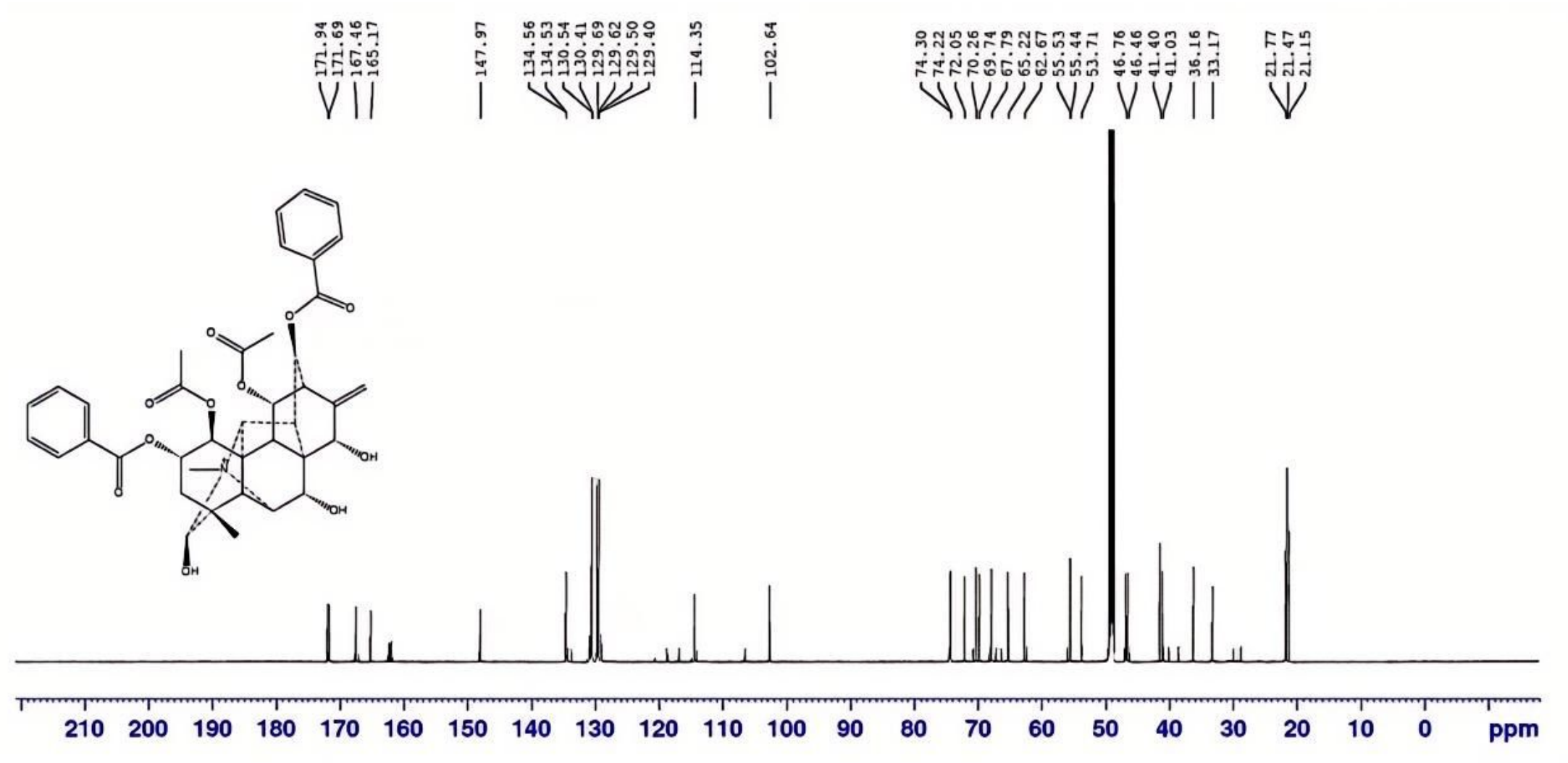

**Figure S102.** $^{13}$C NMR spectrum of **11** in methanol-$d_4$

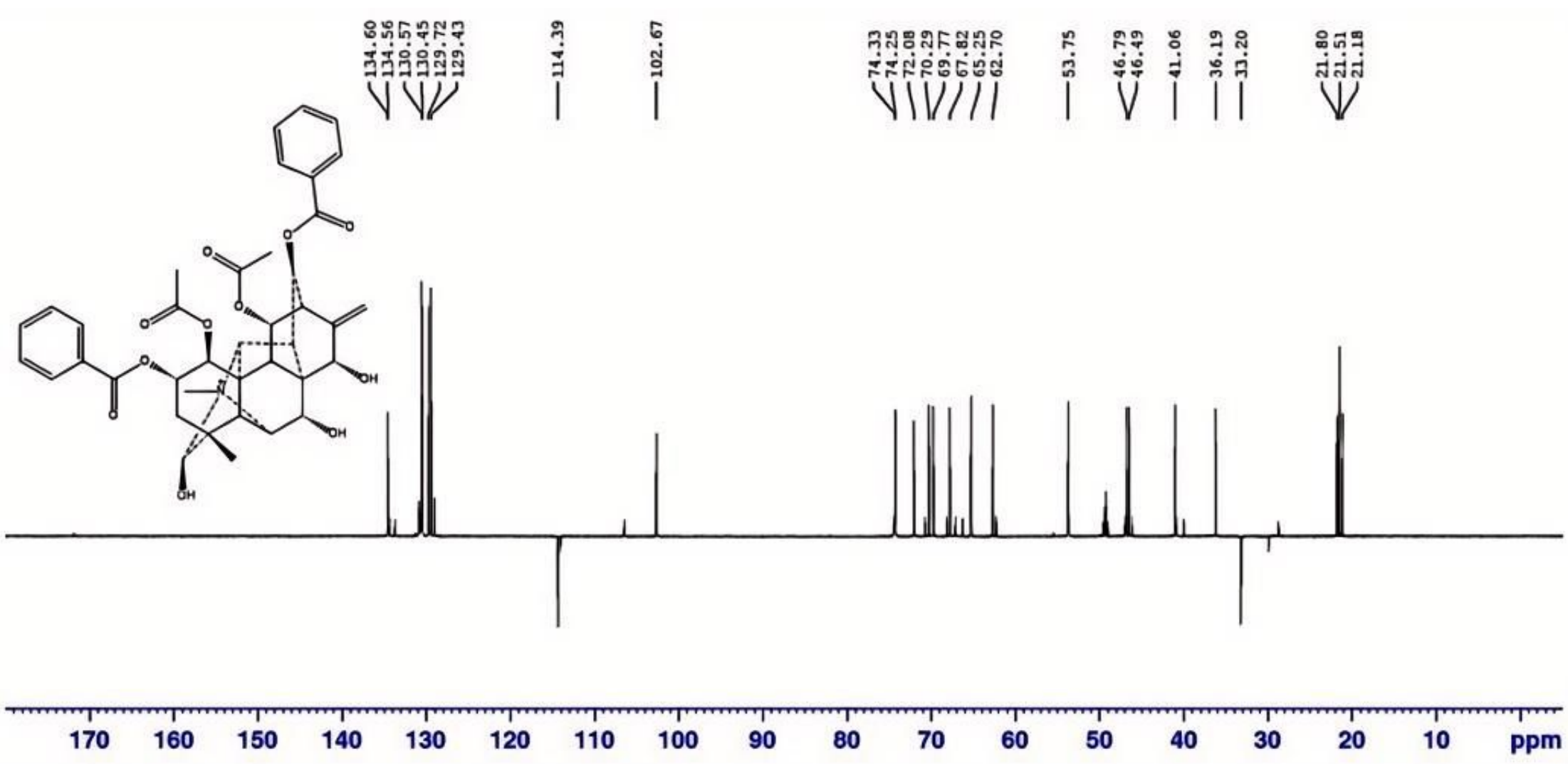


**Figure S103.** DEPT NMR spectrum of **11** in methanol-$d_4$

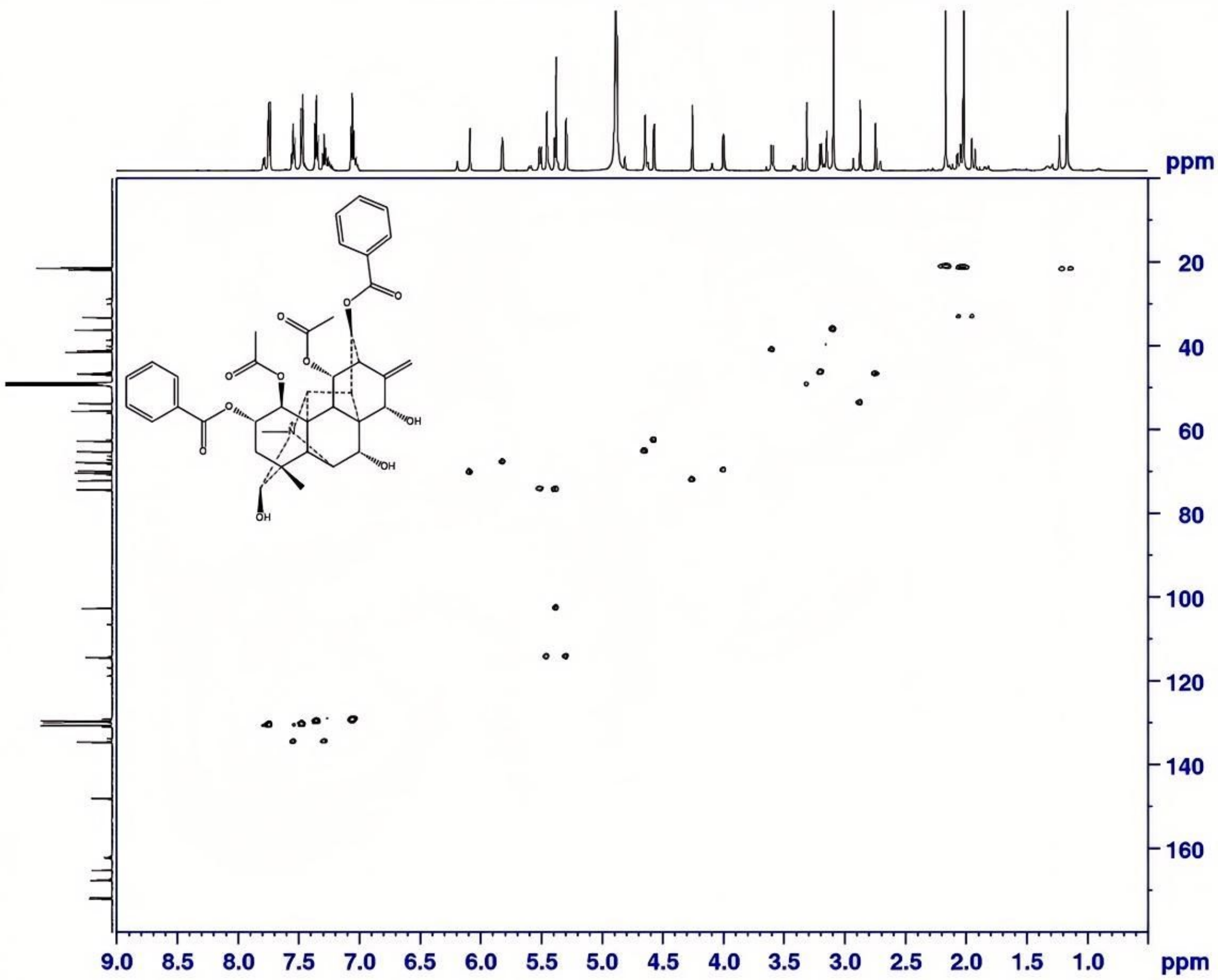


**Figure S104.** HSQC spectrum of **11**

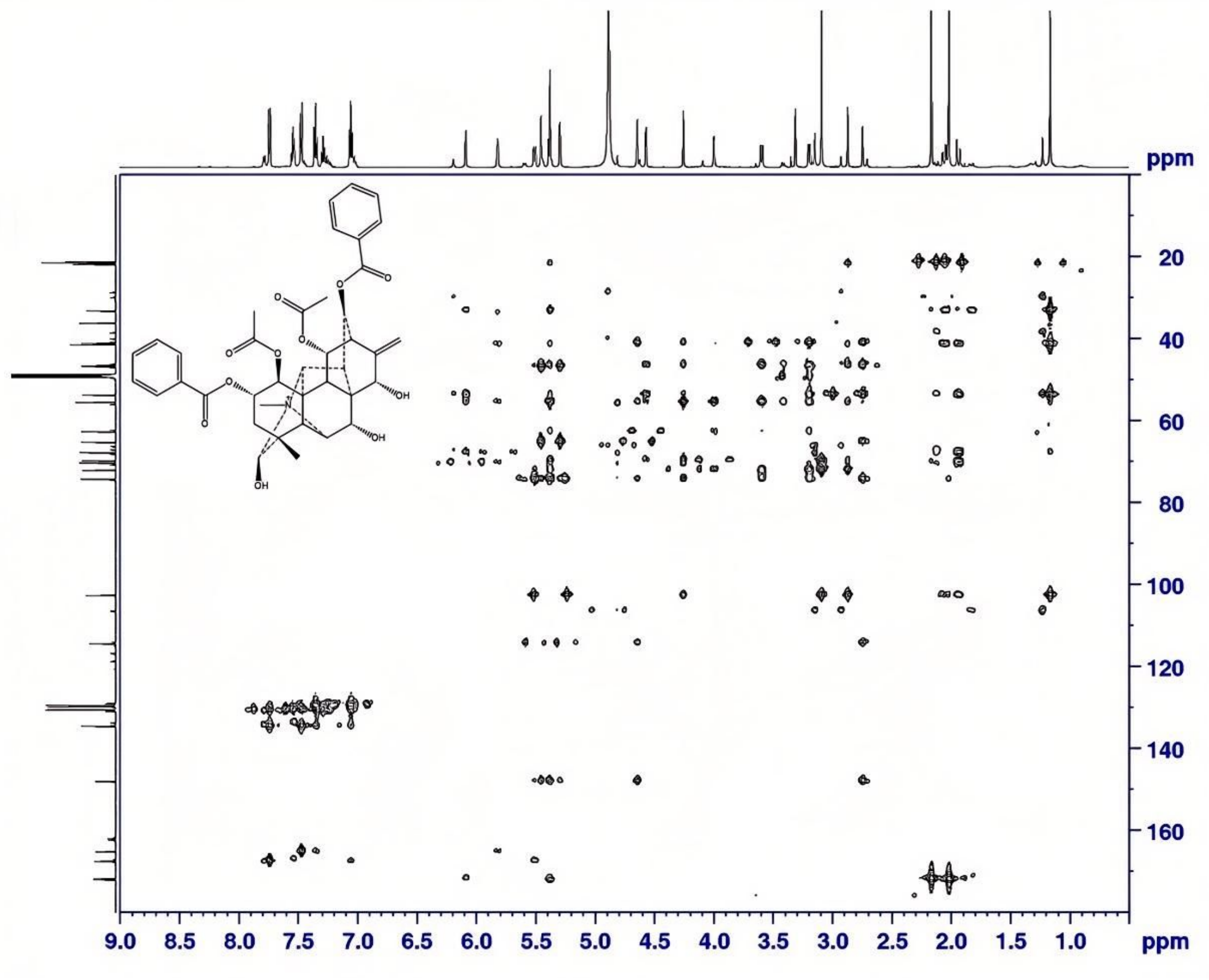


**Figure S105.** HMBC spectrum of **11**

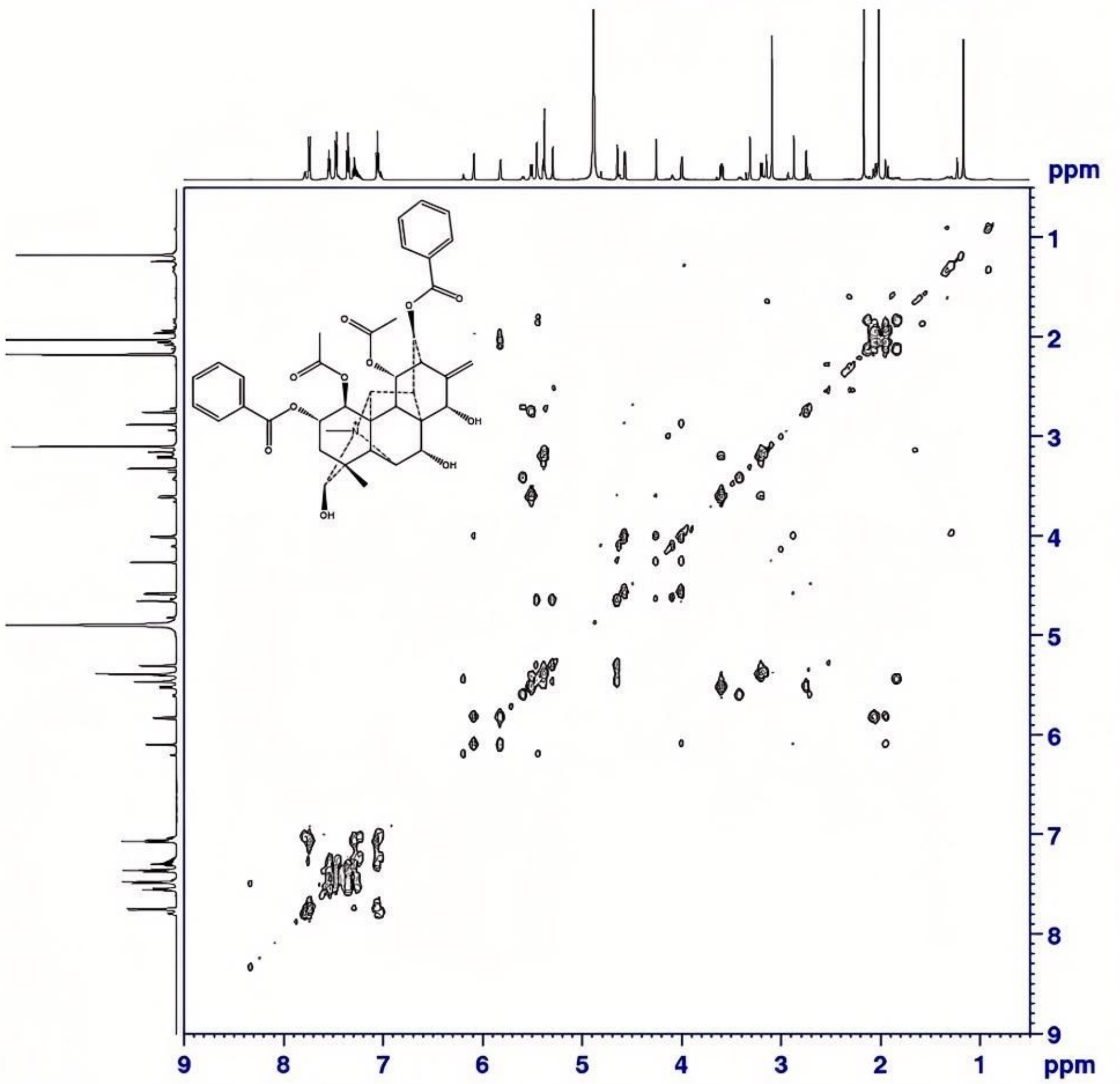

**Figure S106.** $^{1}H$-$^{1}H$ COSY spectrum of **11**

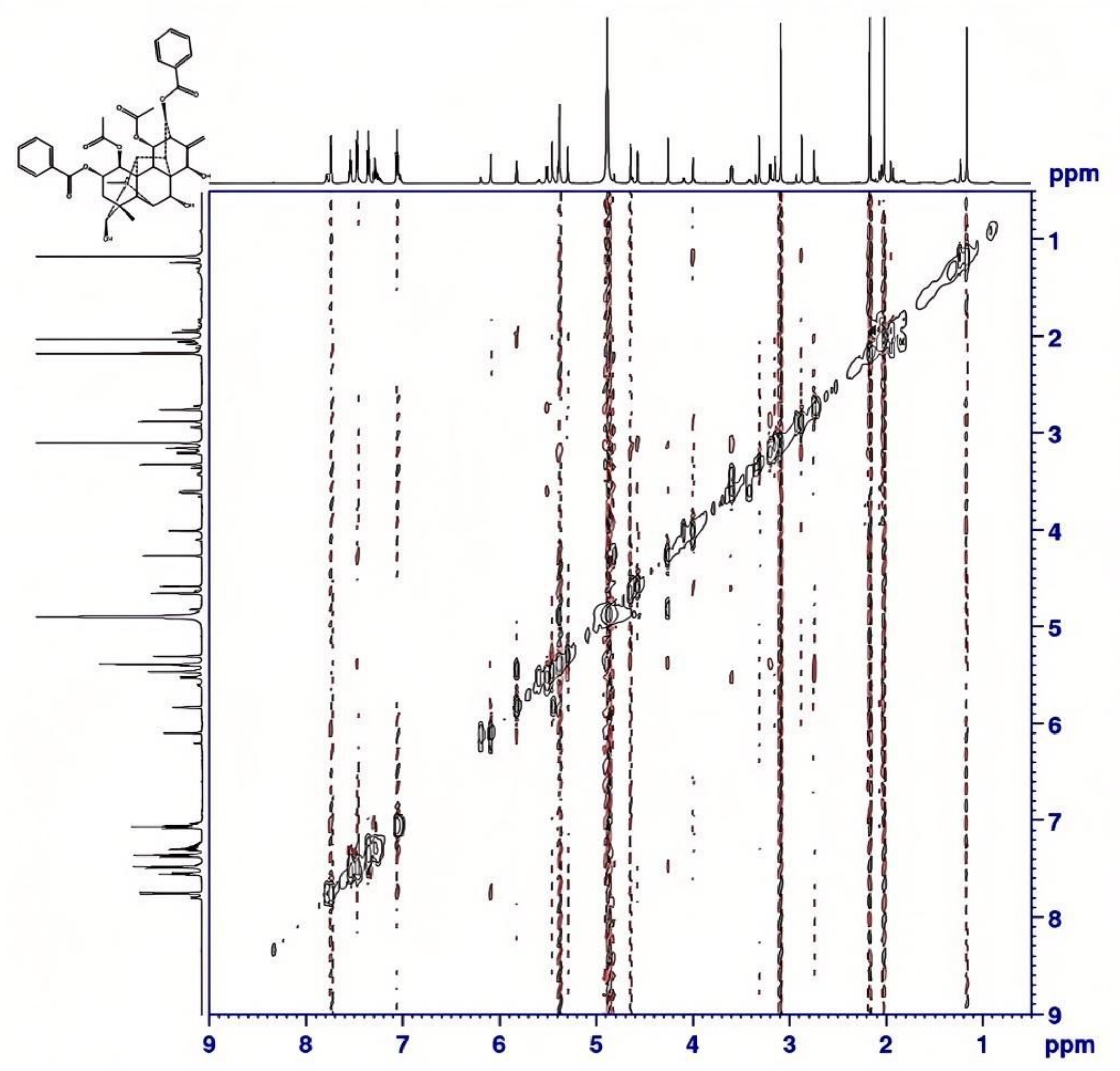

**Figure S107.** NOESY spectrum of **11**

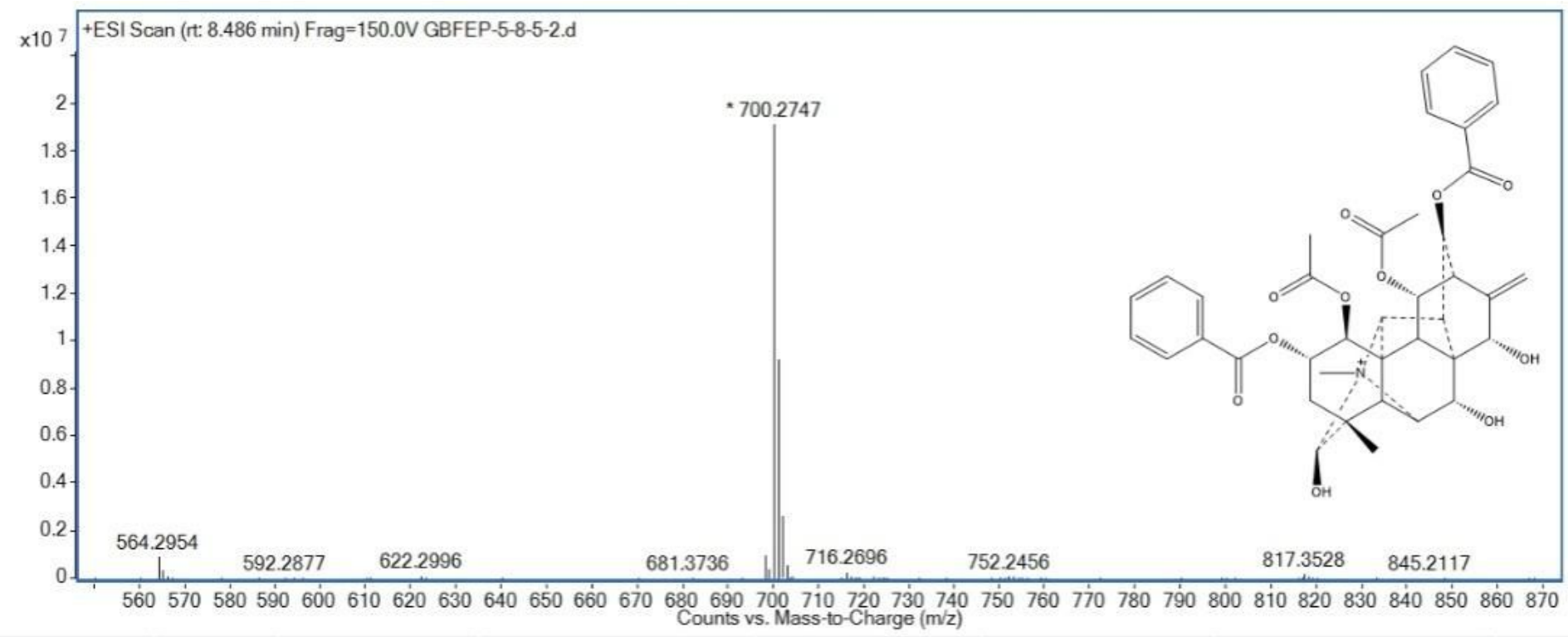


| Formula | Score | Mass | Mass (MFG) | m/z (Calc) | Diff (ppm) | Ion Formula | m/z |
|---|---|---|---|---|---|---|---|
| $C_{39}H_{42}NO_{11}$ | 99.61 | 700.2752 | 700.2758 | 700.2752 | 0.77 | $C_{39}H_{42}NO_{11}$ | 700.2747 |

**Figure S108.** HRESIMS spectrum of **11**

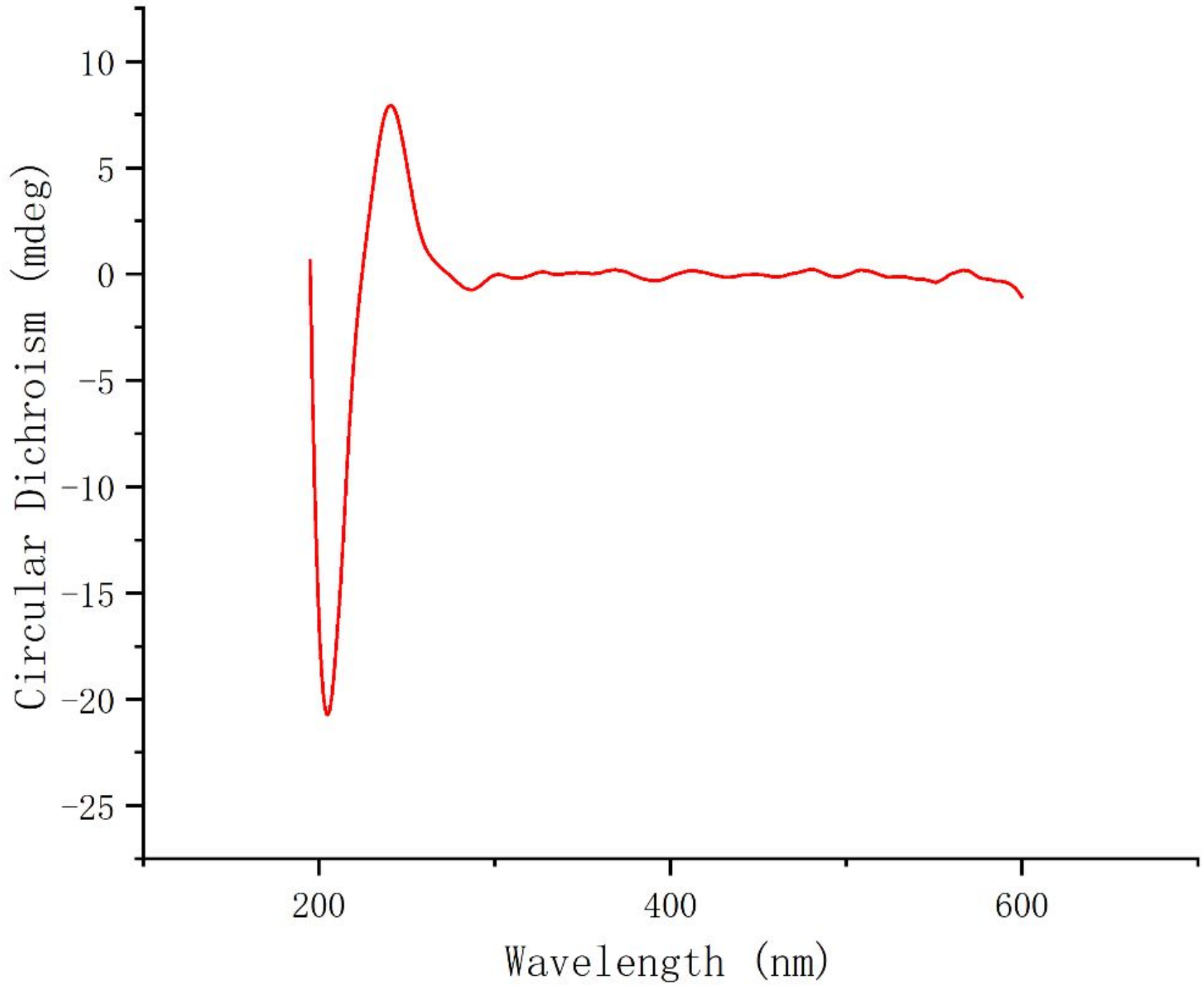


**Figure S109.** ECD spectrum of **11**

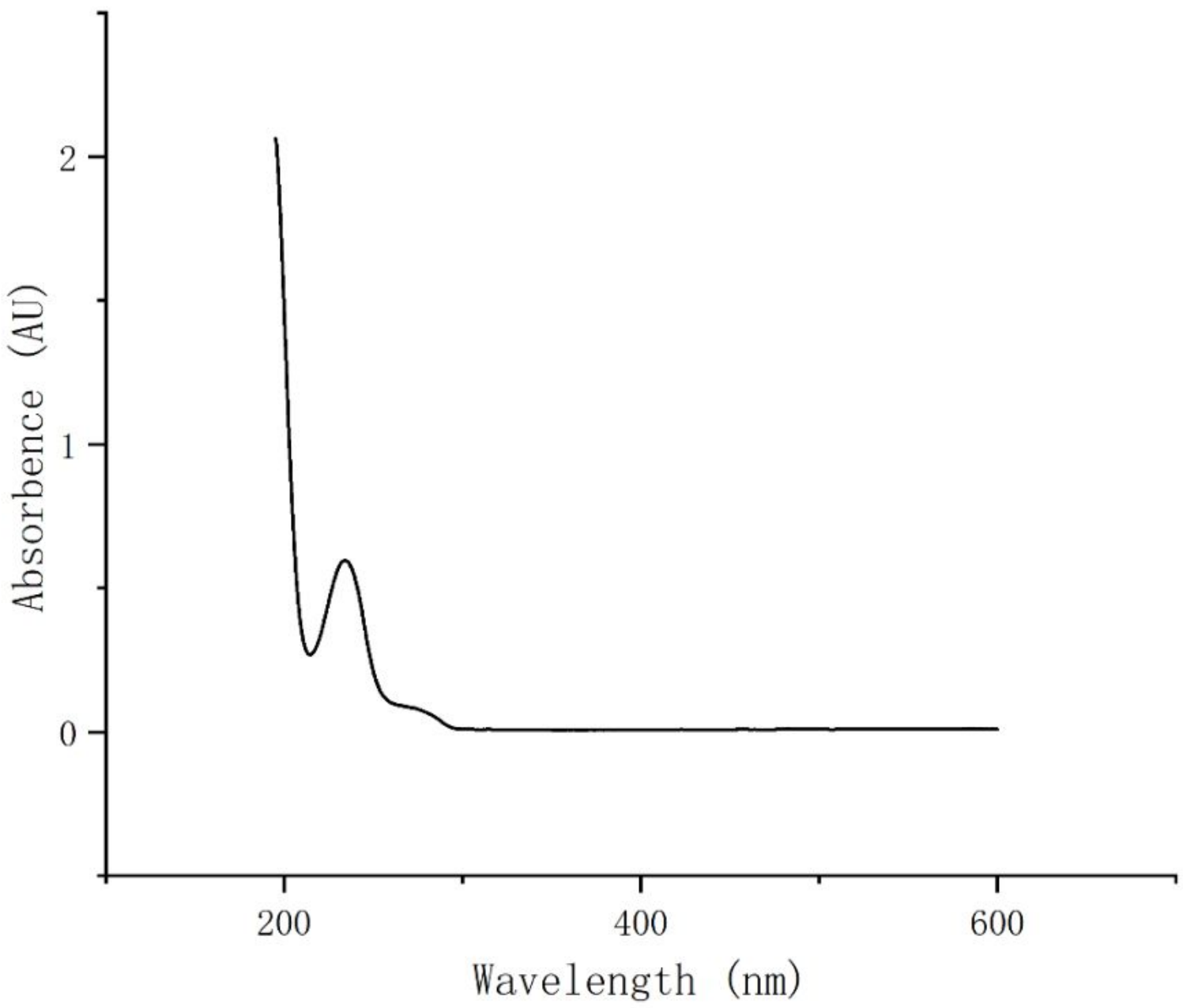


**Figure S110.** UV spectrum of **11**

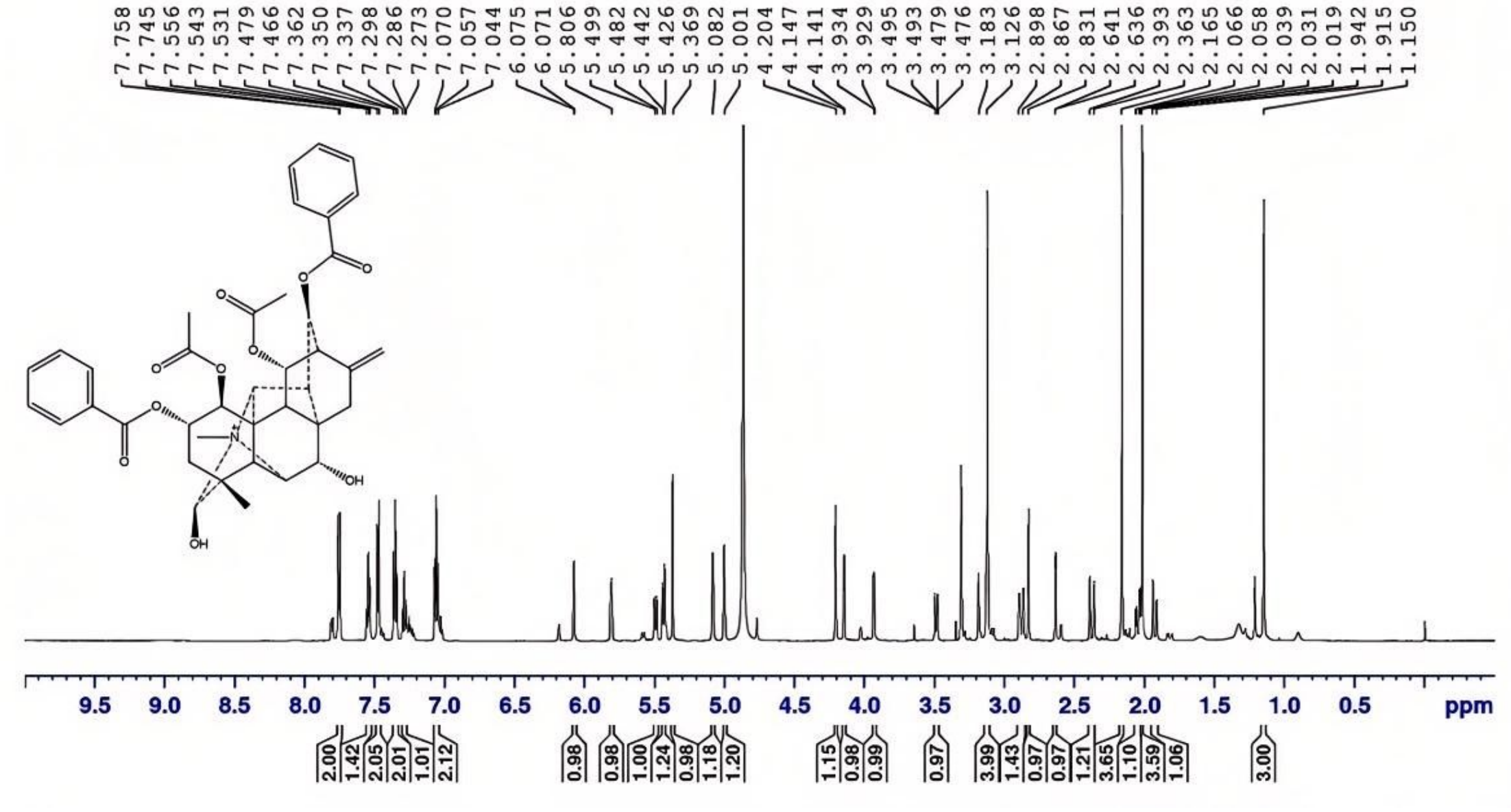

**Figure S111.** $^{1}$H NMR spectrum of **12** in methanol-$d_4$

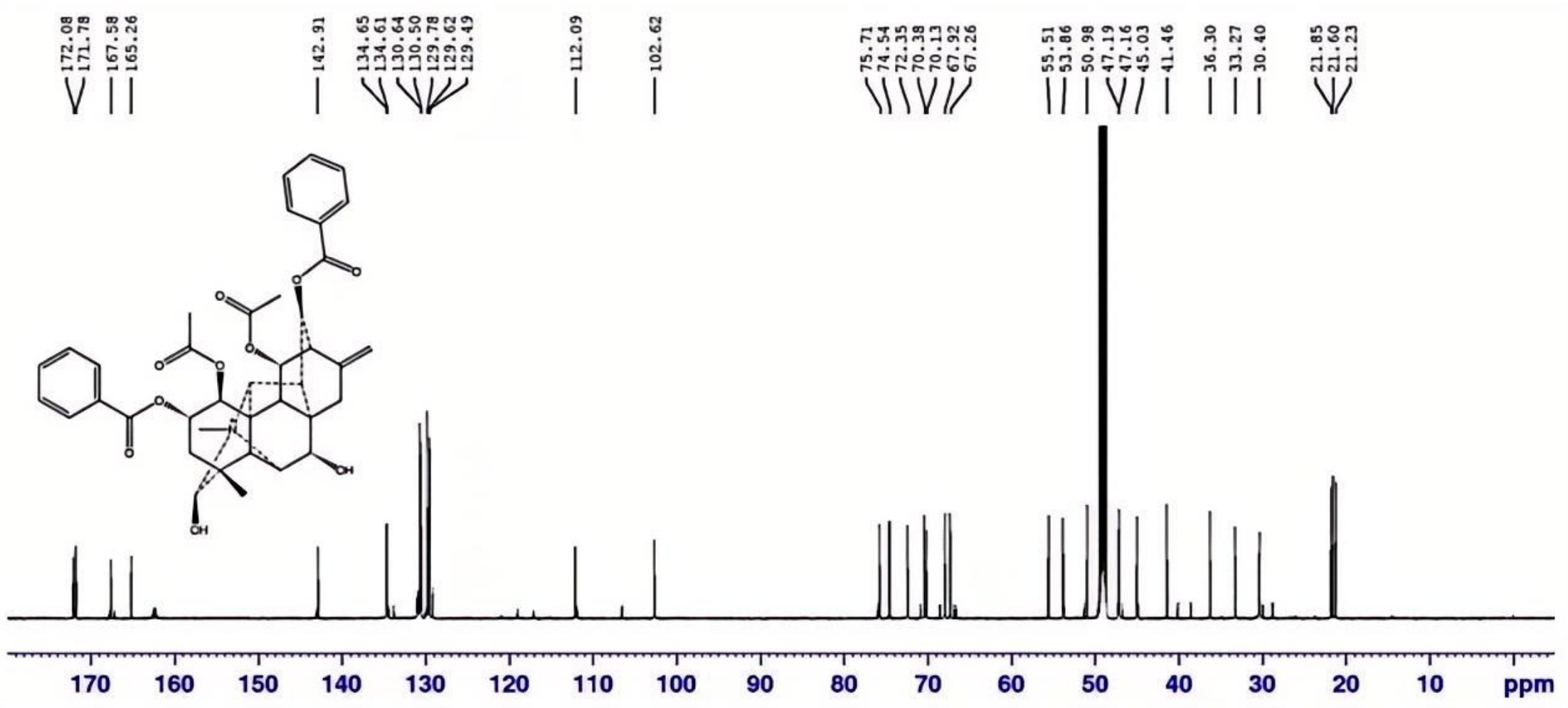

**Figure S112.** $^{13}$C NMR spectrum of **12** in methanol-$d_4$

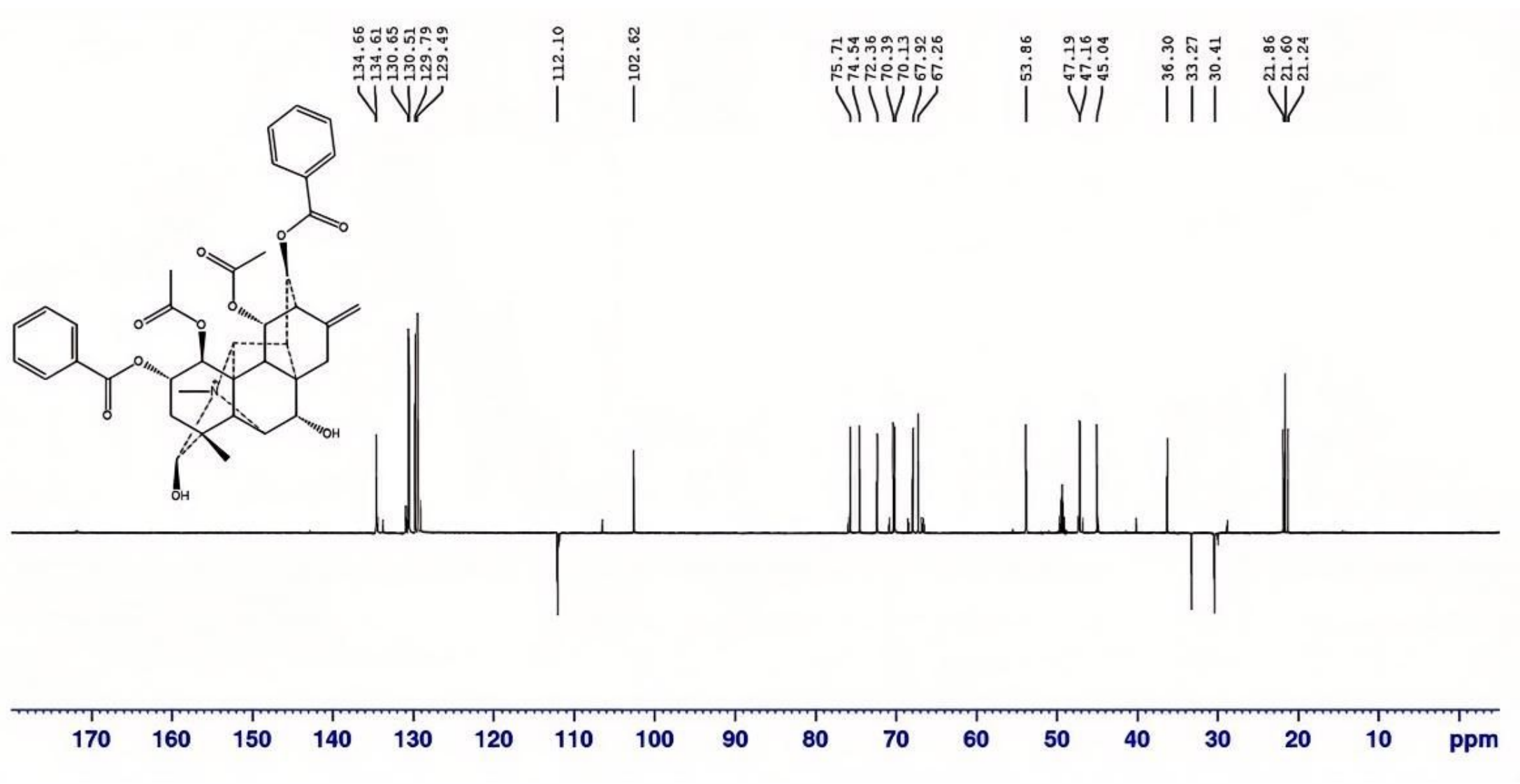


**Figure S113.** DEPT NMR spectrum of **12** in methanol-$d_4$

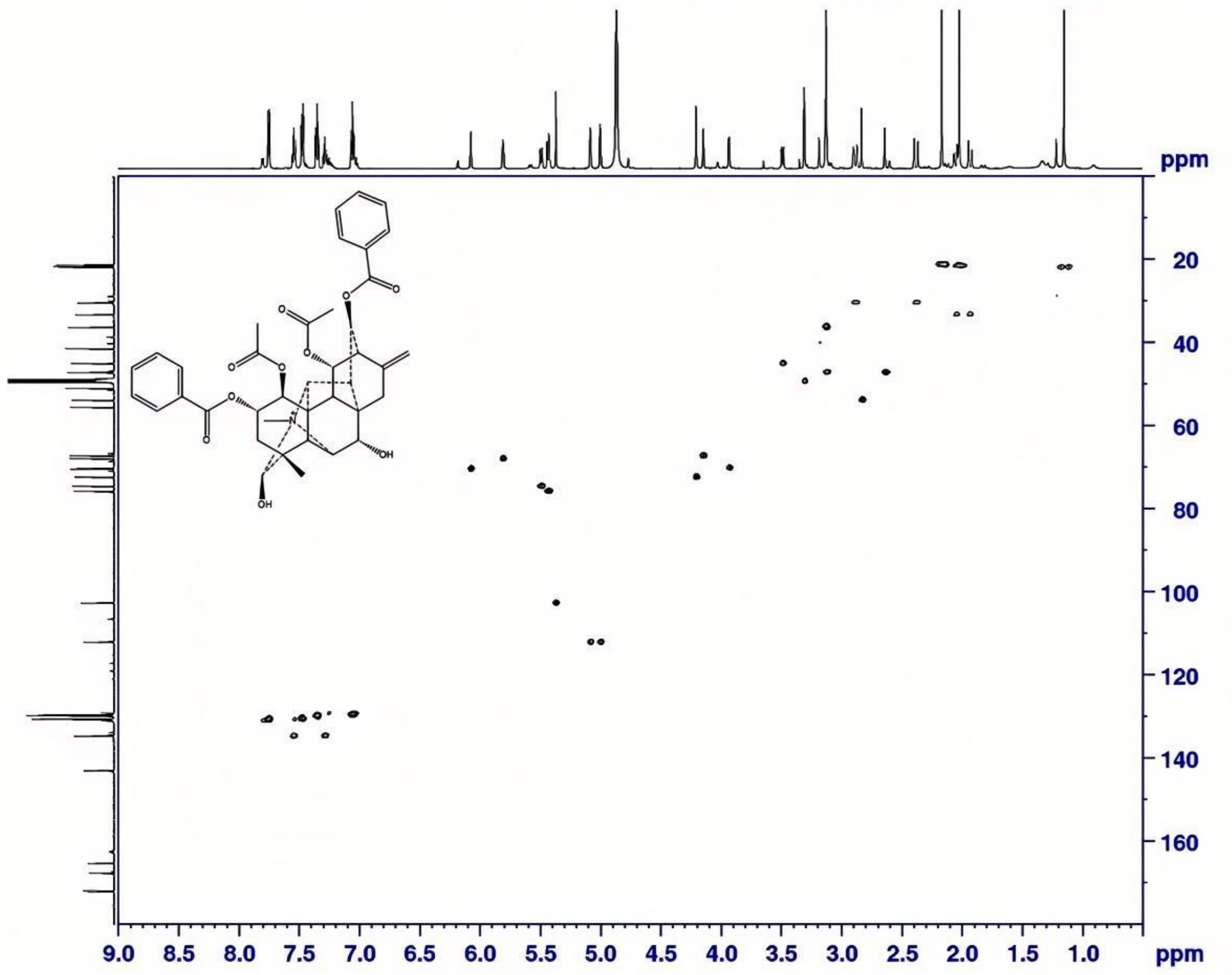


**Figure S114.** HSQC spectrum of **12**

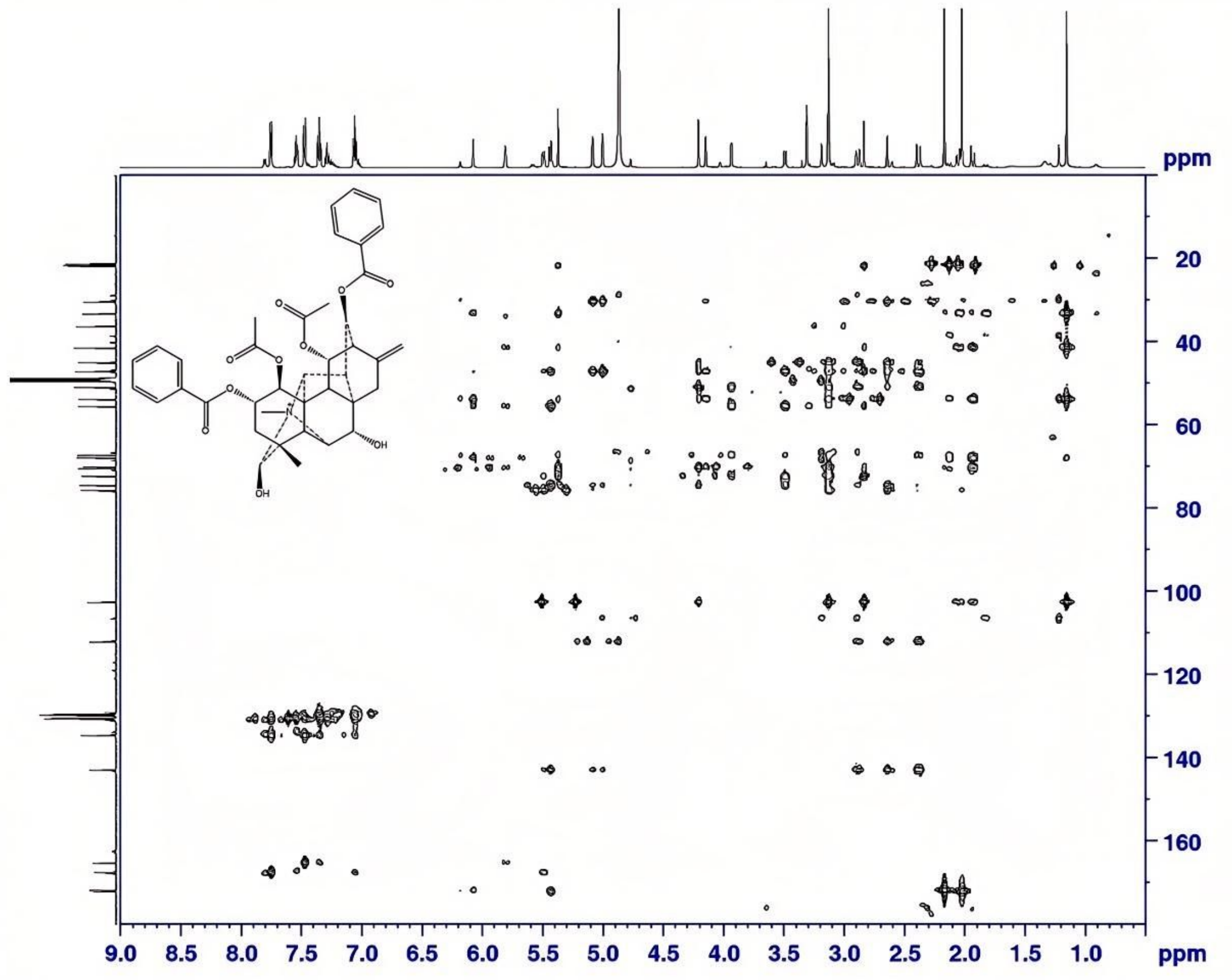


**Figure S115.** HMBC spectrum of **12**

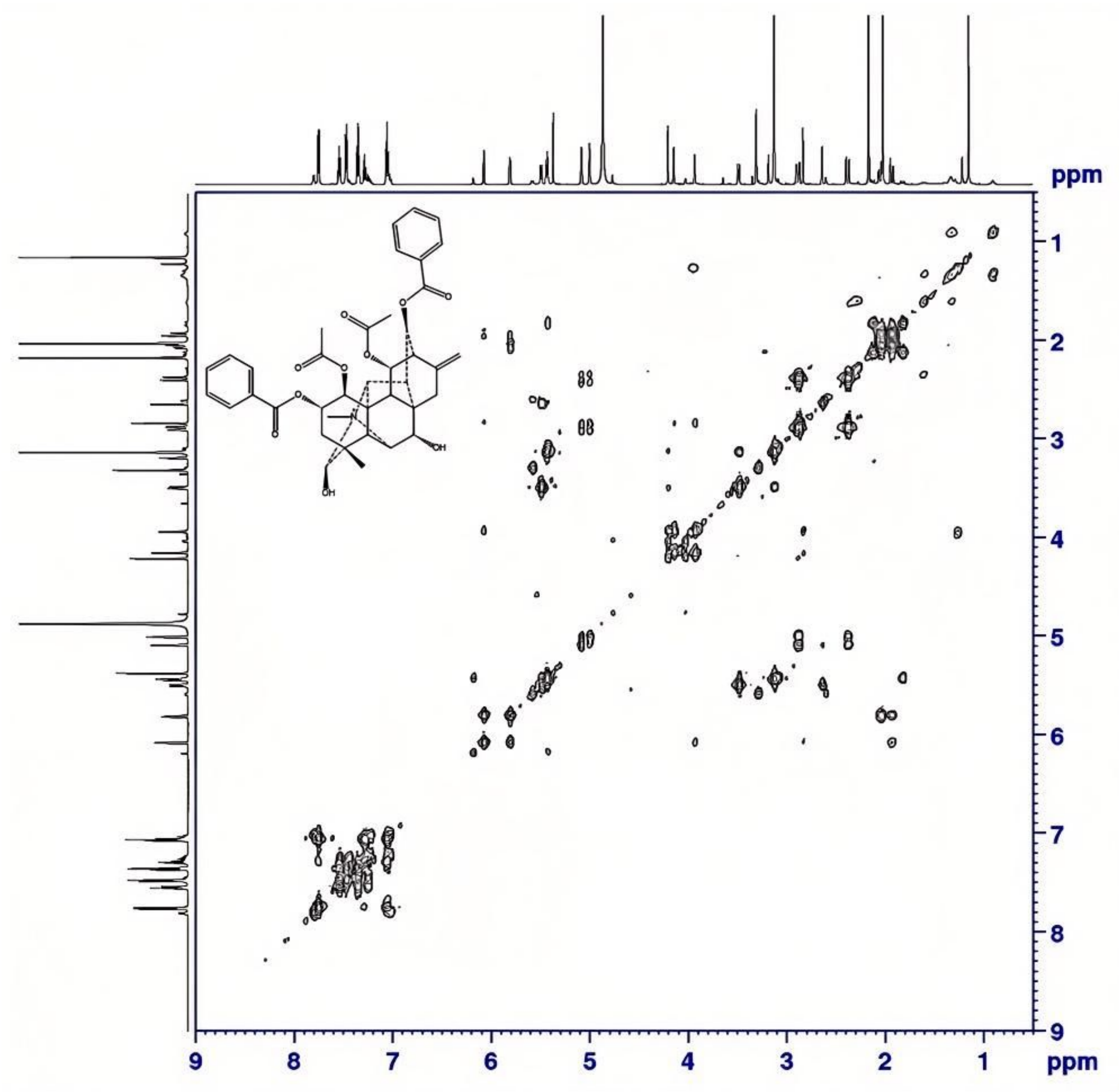


**Figure S116.** $^{1}H$-$^{1}H$ COSY spectrum of **12**

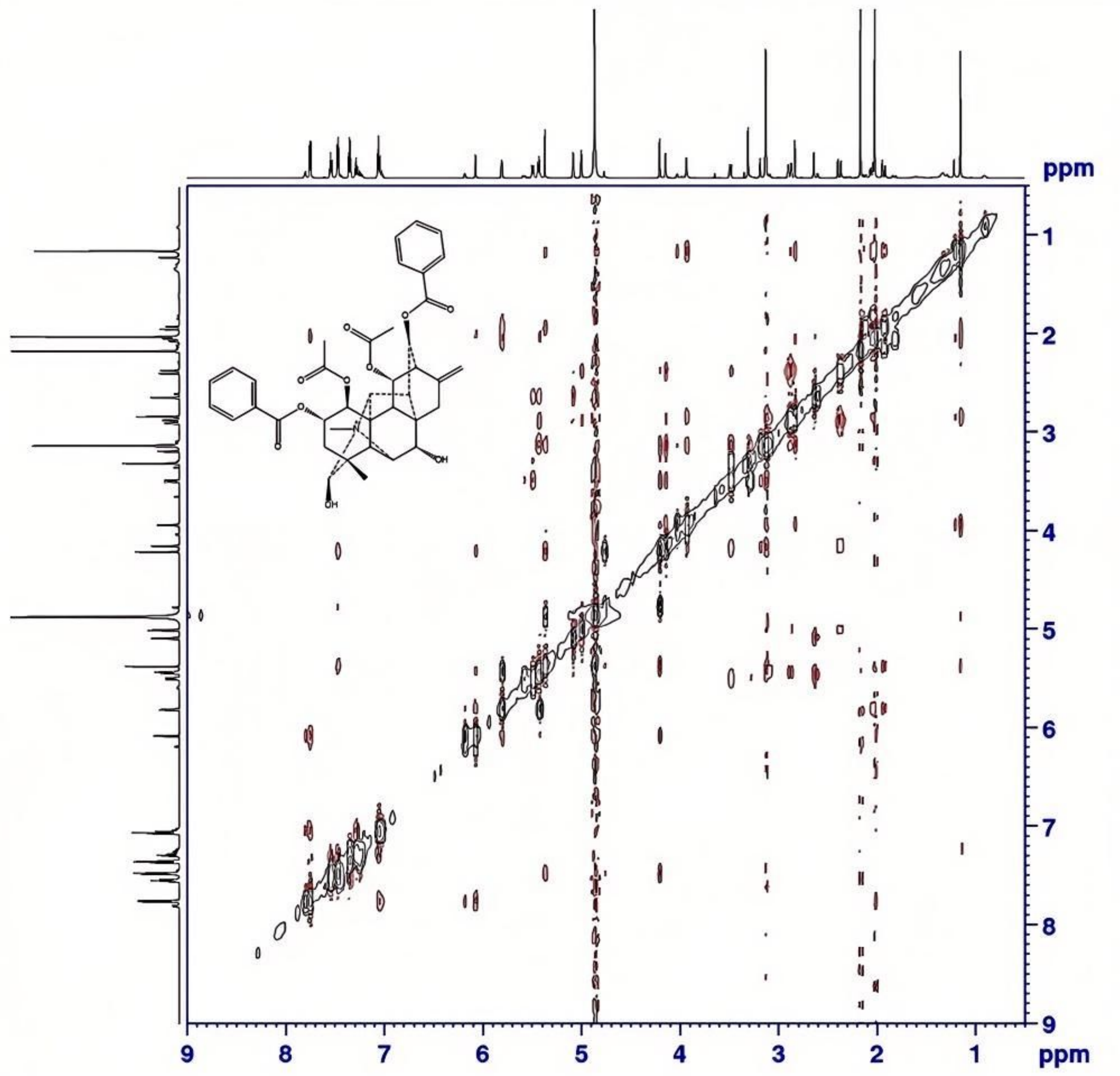

**Figure S117.** NOESY spectrum of **12**

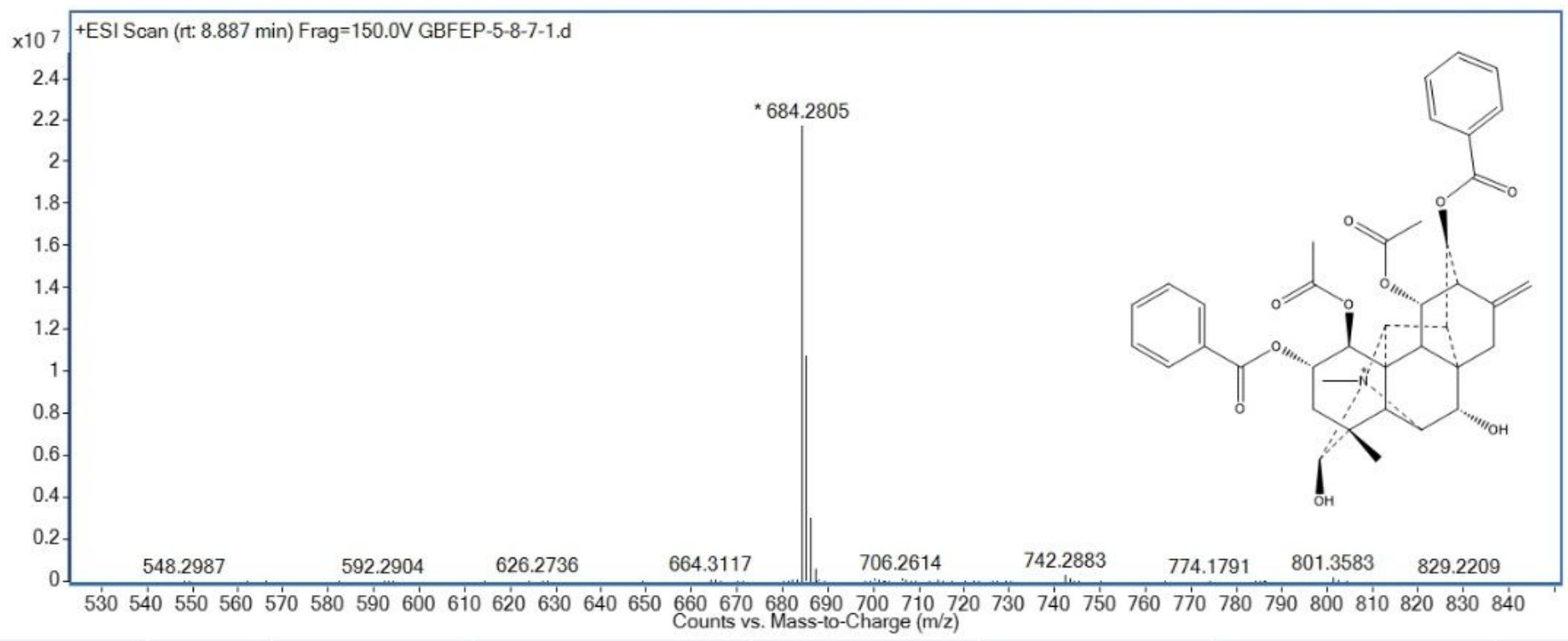


| Formula | Score | Mass | Mass (MFG) | m/z (Calc) | Diff (ppm) | Ion Formula | m/z |
|---|---|---|---|---|---|---|---|
| $C_{39}H_{42}NO_{10}$ | 99.96 | 684.2810 | 687.2809 | 684.2803 | -0.26 | $C_{39}H_{42}NO_{10}$ | 684.2805 |

**Figure S118.** HRESIMS spectrum of **12**

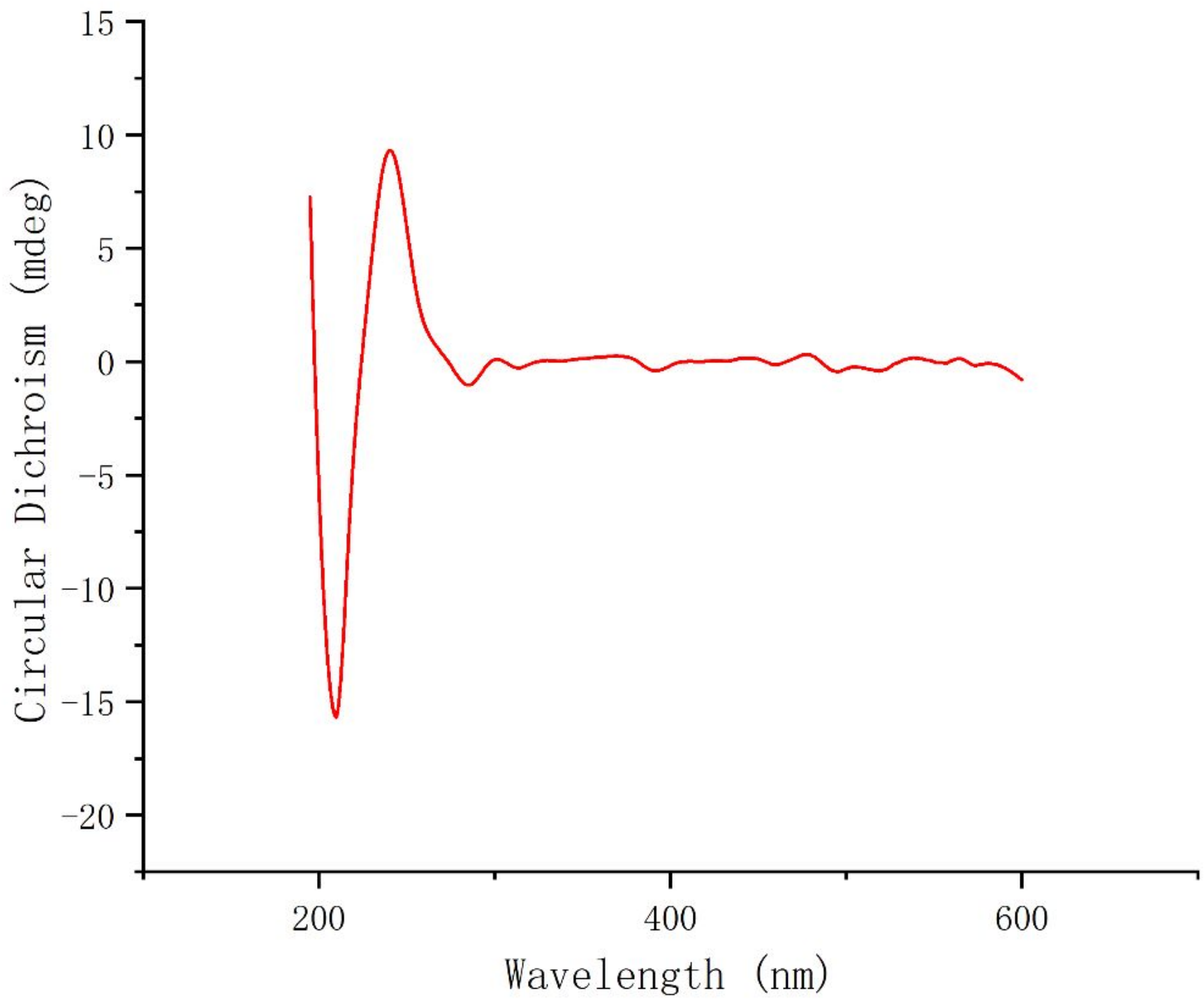


**Figure S119.** ECD spectrum of **12**

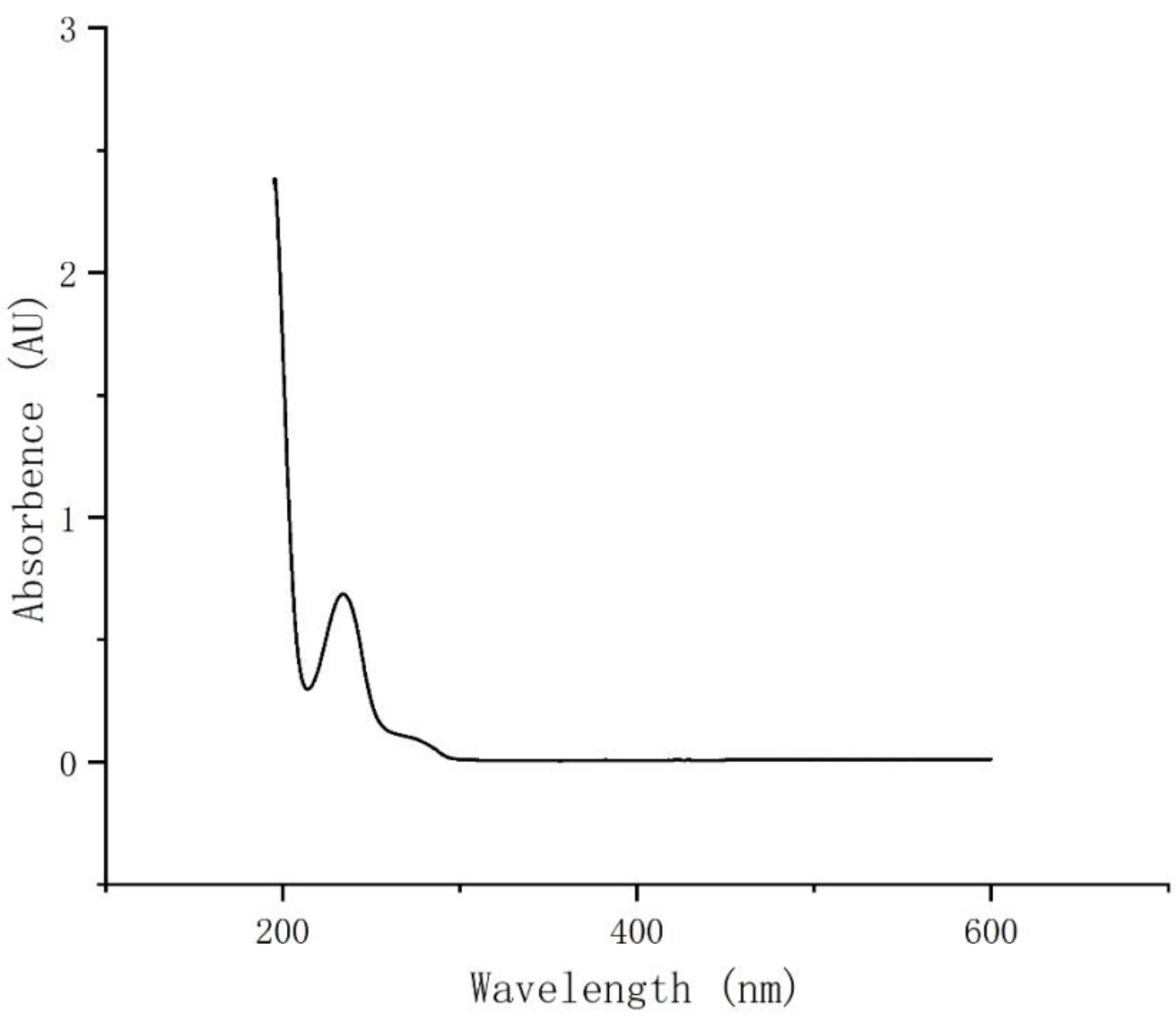


**Figure S120.** UV spectrum of **12**

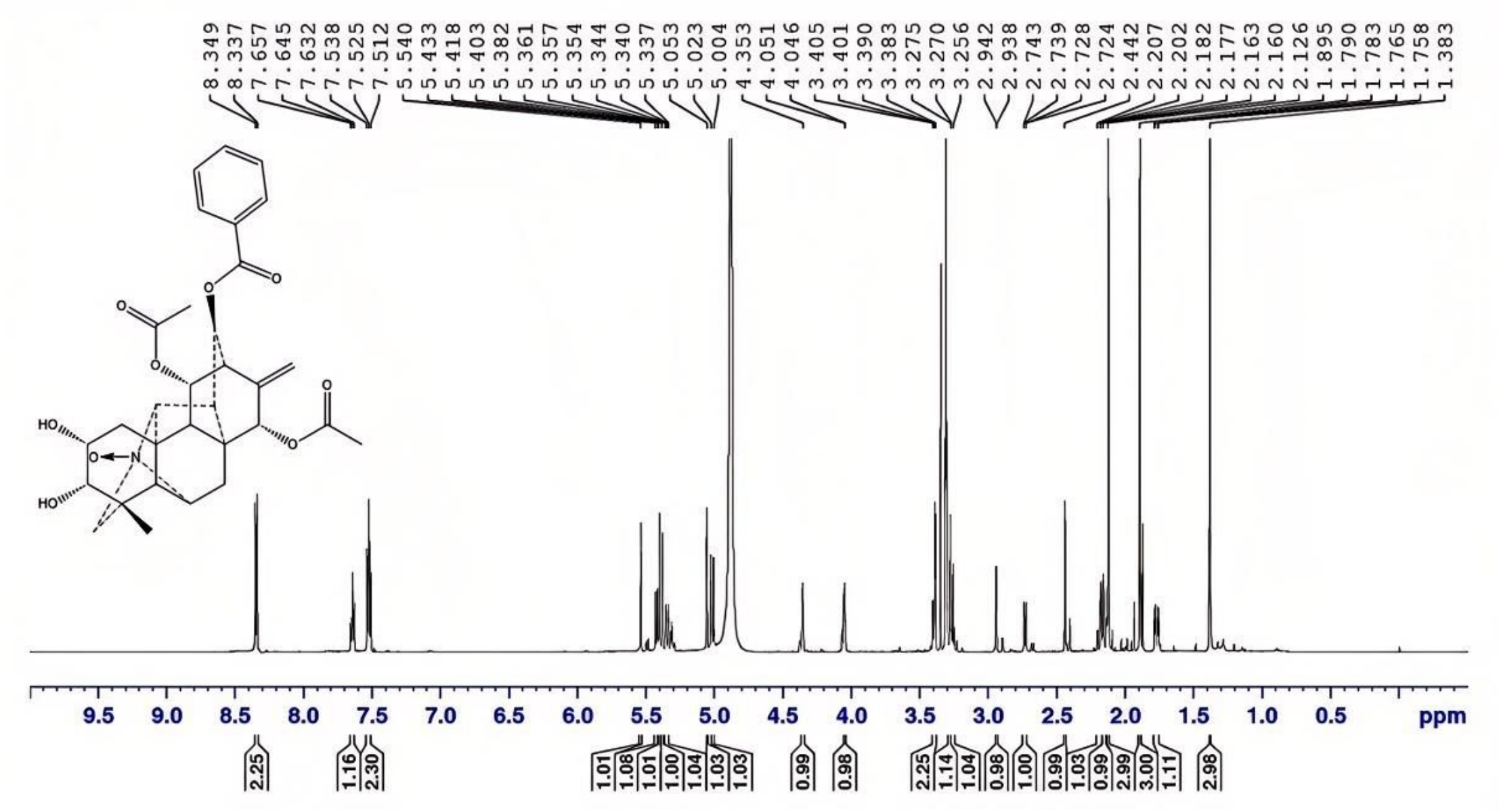

**Figure S121.** $^{1}$H NMR spectrum of **13** in methanol-$d_4$

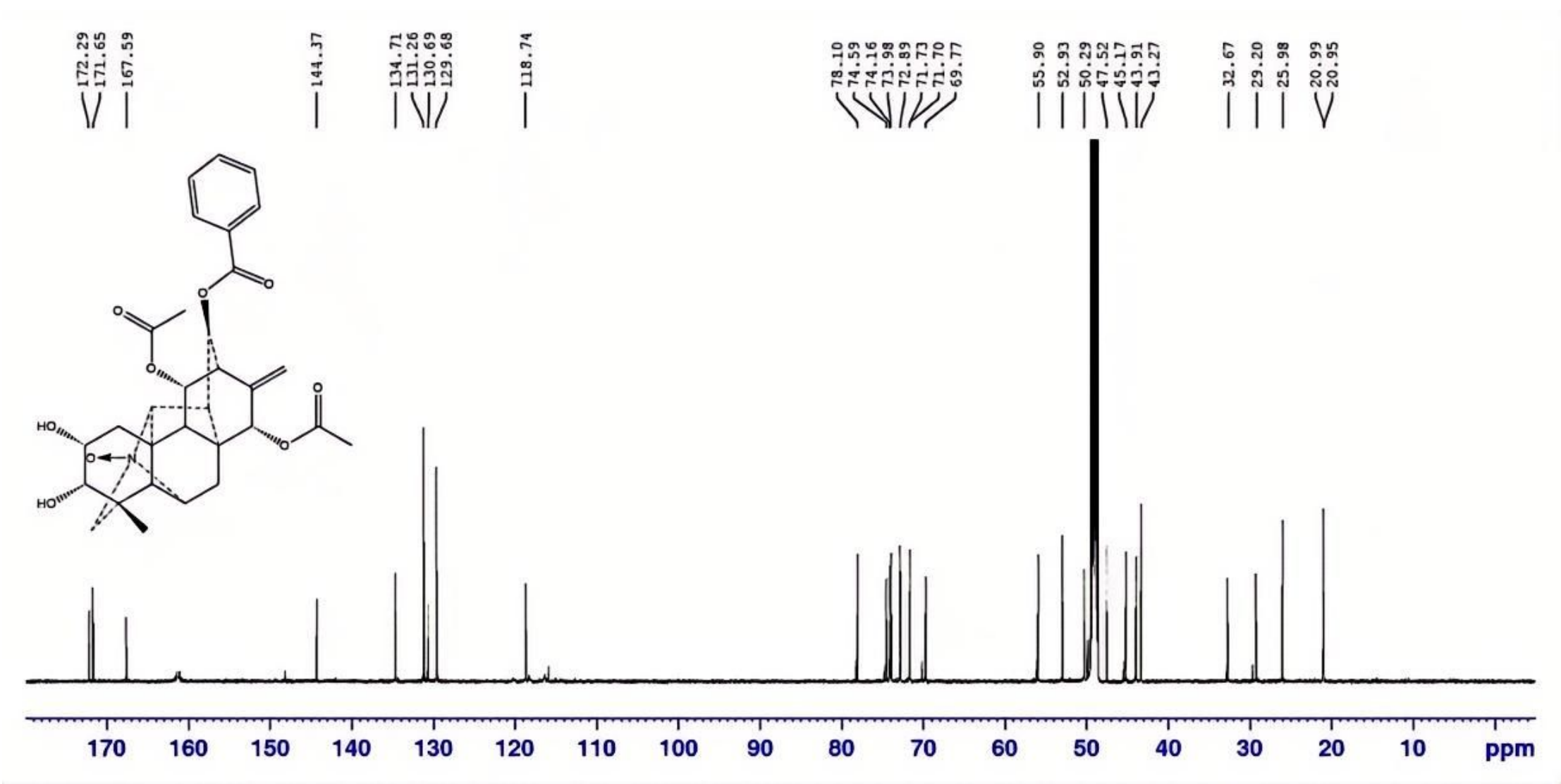

**Figure S122.** $^{13}$C NMR spectrum of **13** in methanol-$d_4$

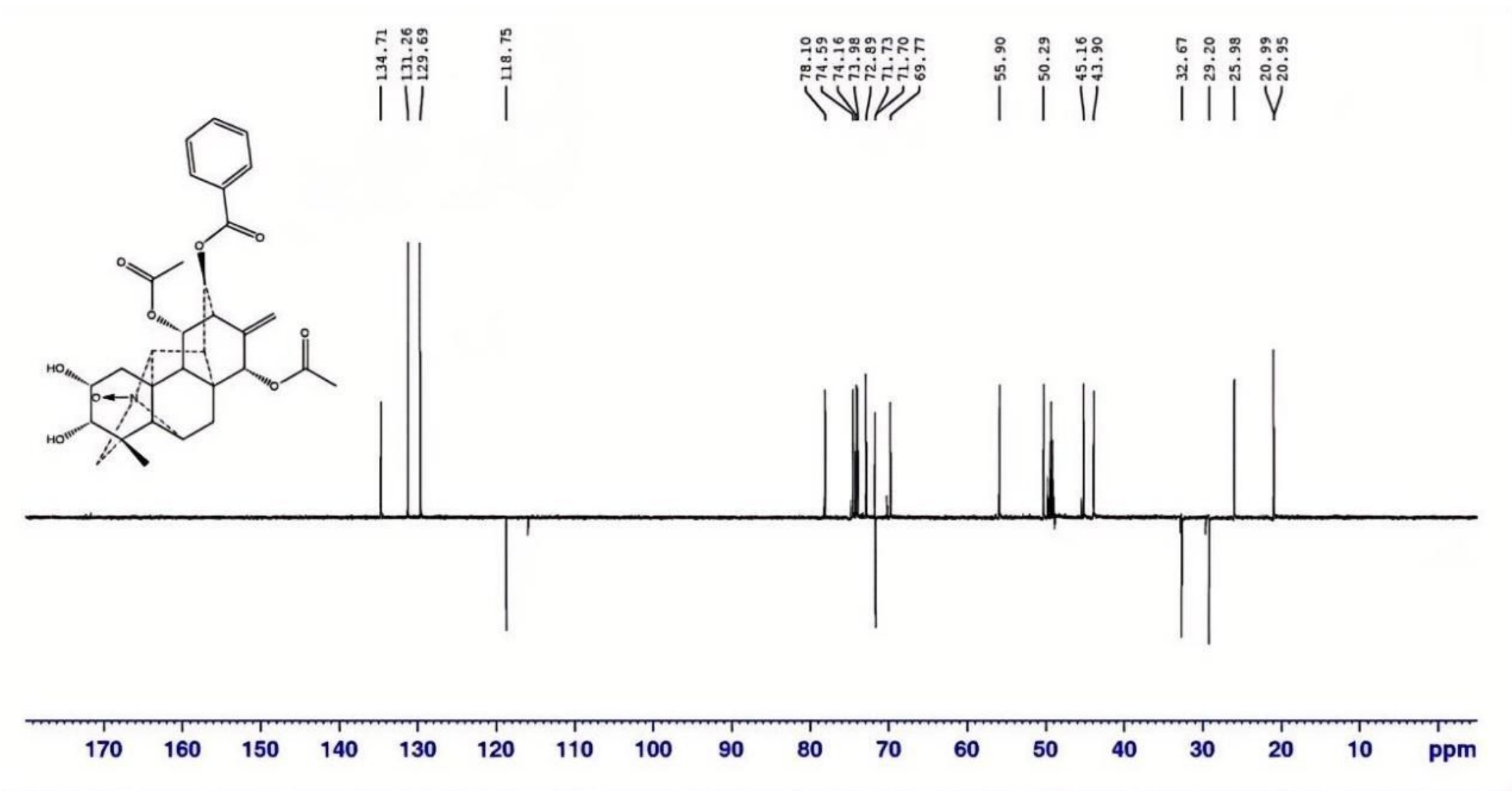


**Figure S123.** DEPT NMR spectrum of **13** in methanol-$d_4$

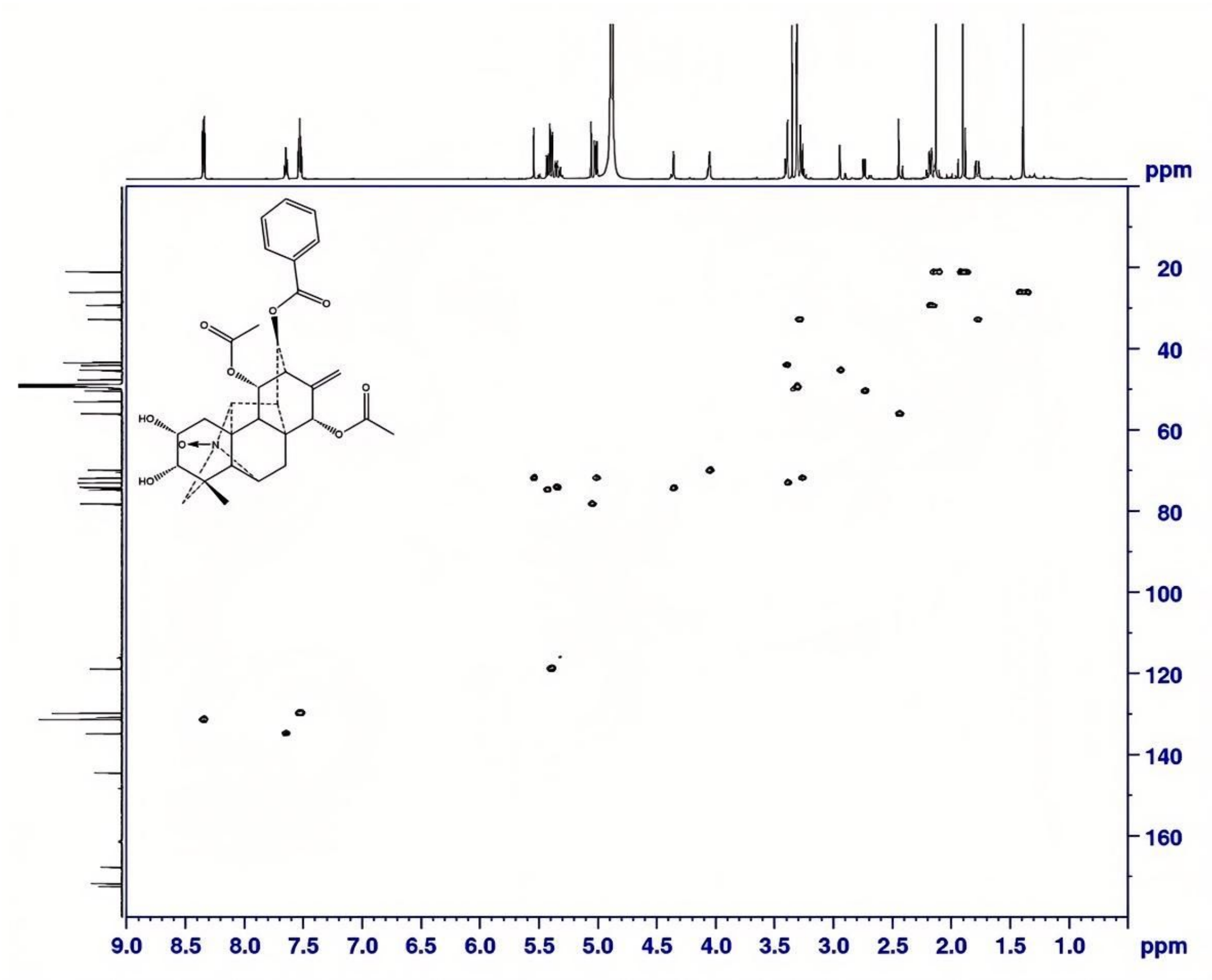


**Figure S124.** HSQC spectrum of **13**

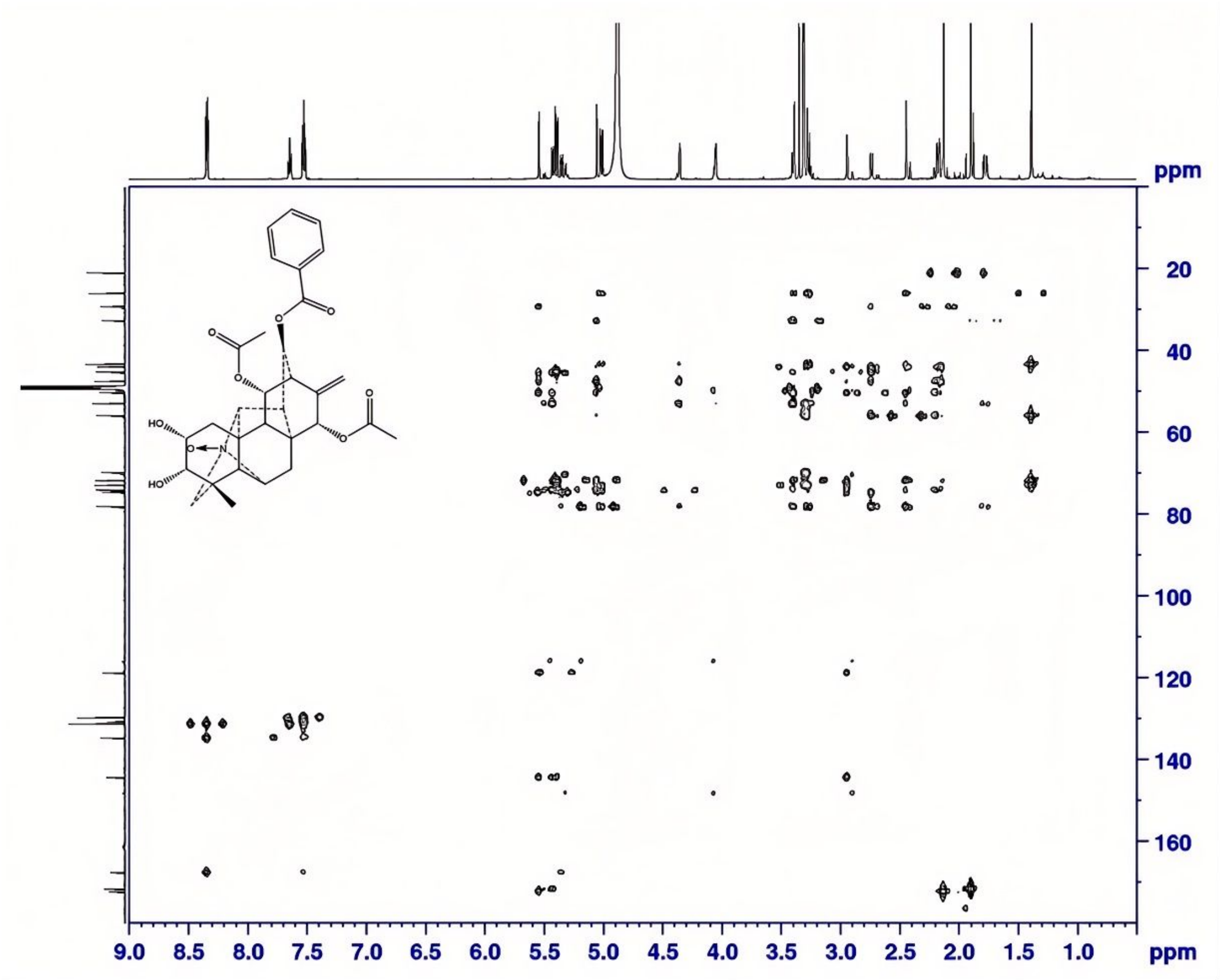


**Figure S125.** HMBC spectrum of **13**

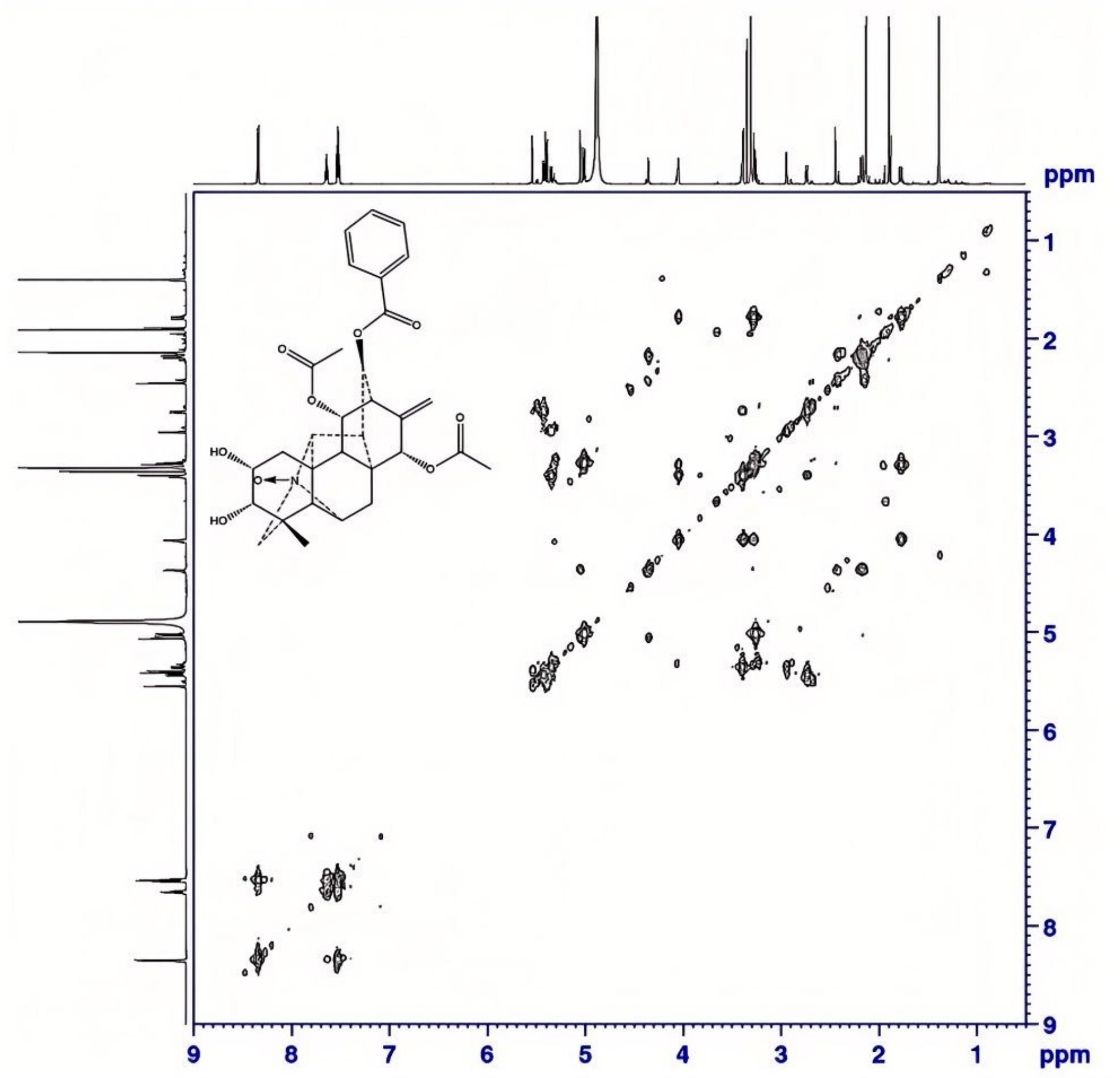


**Figure S126.** $^1H$-$^1H$ COSY spectrum of **13**

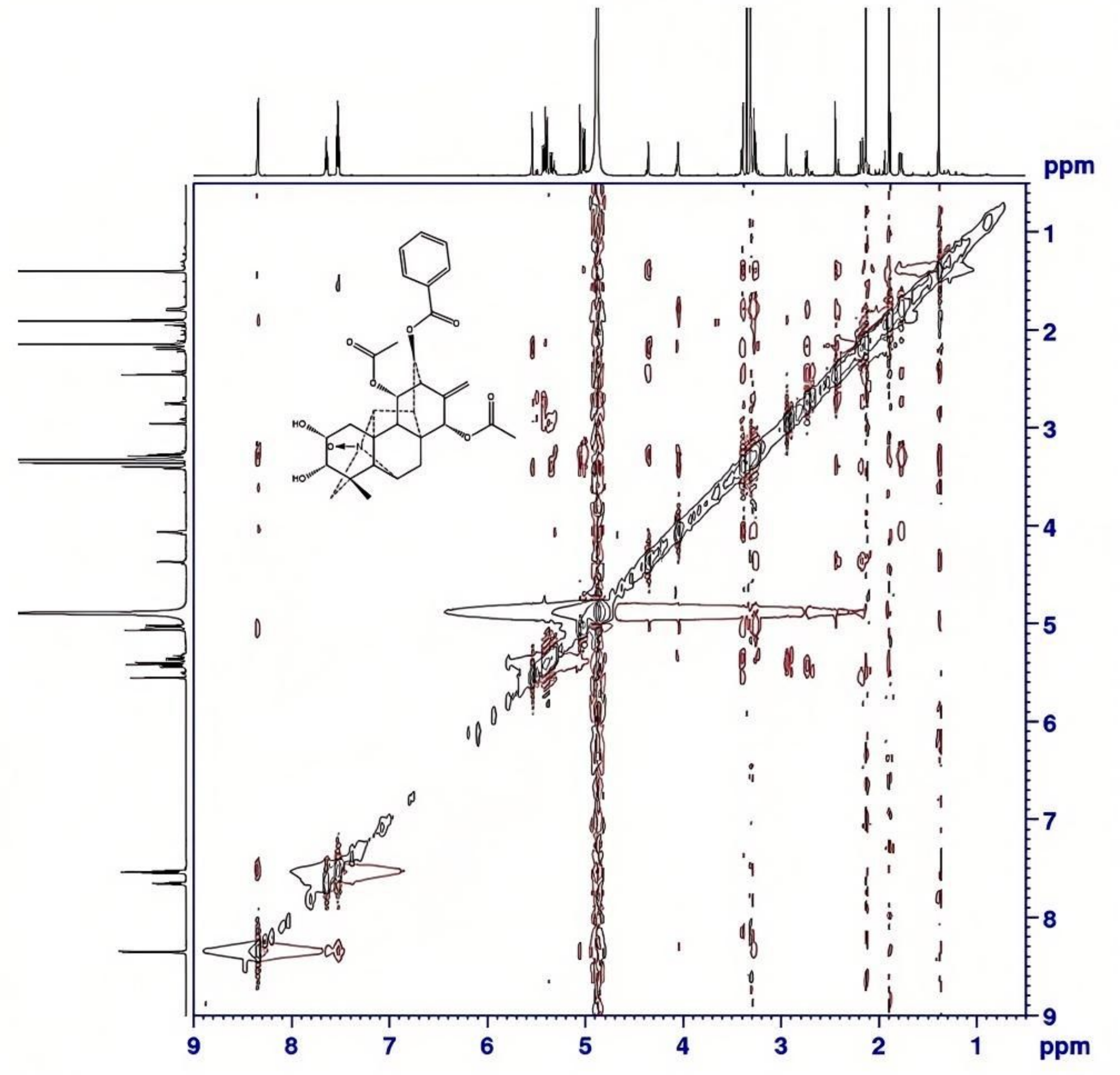

**Figure S127.** NOESY spectrum of **13**

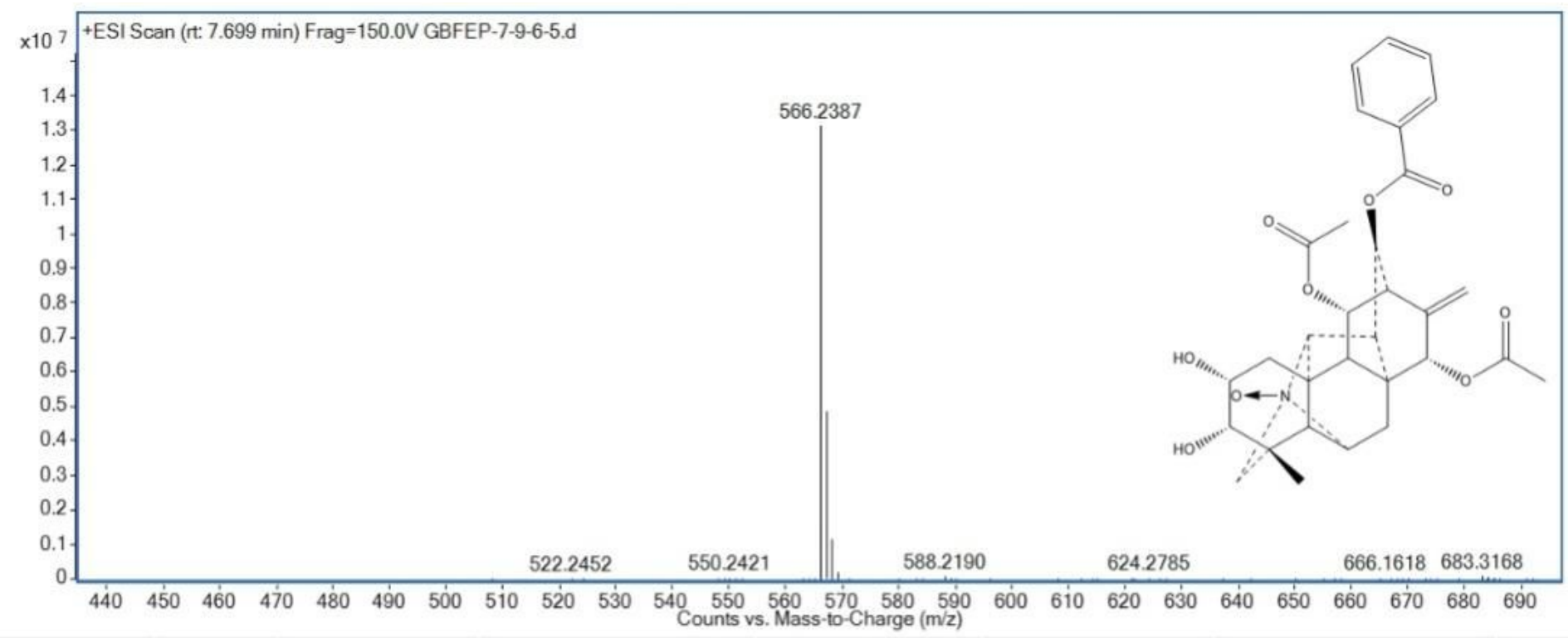

| Formula | Score | Mass | Mass (MFG) | m/z (Calc) | Diff (ppm) | Ion Formula | m/z |
|---|---|---|---|---|---|---|---|
| $C_{31}H_{35}NO_9$ | 99.90 | 565.2314 | 565.2312 | 566.2385 | -0.43 | $C_{31}H_{36}NO_9$ | 566.2387 |
| $C_{31}H_{35}NO_9$ | 96.61 | 565.2298 | 565.2312 | 588.2204 | 2.48 | $C_{31}H_{35}NO_9Na$ | 588.2190 |

**Figure S128.** HRESIMS spectrum of **13**

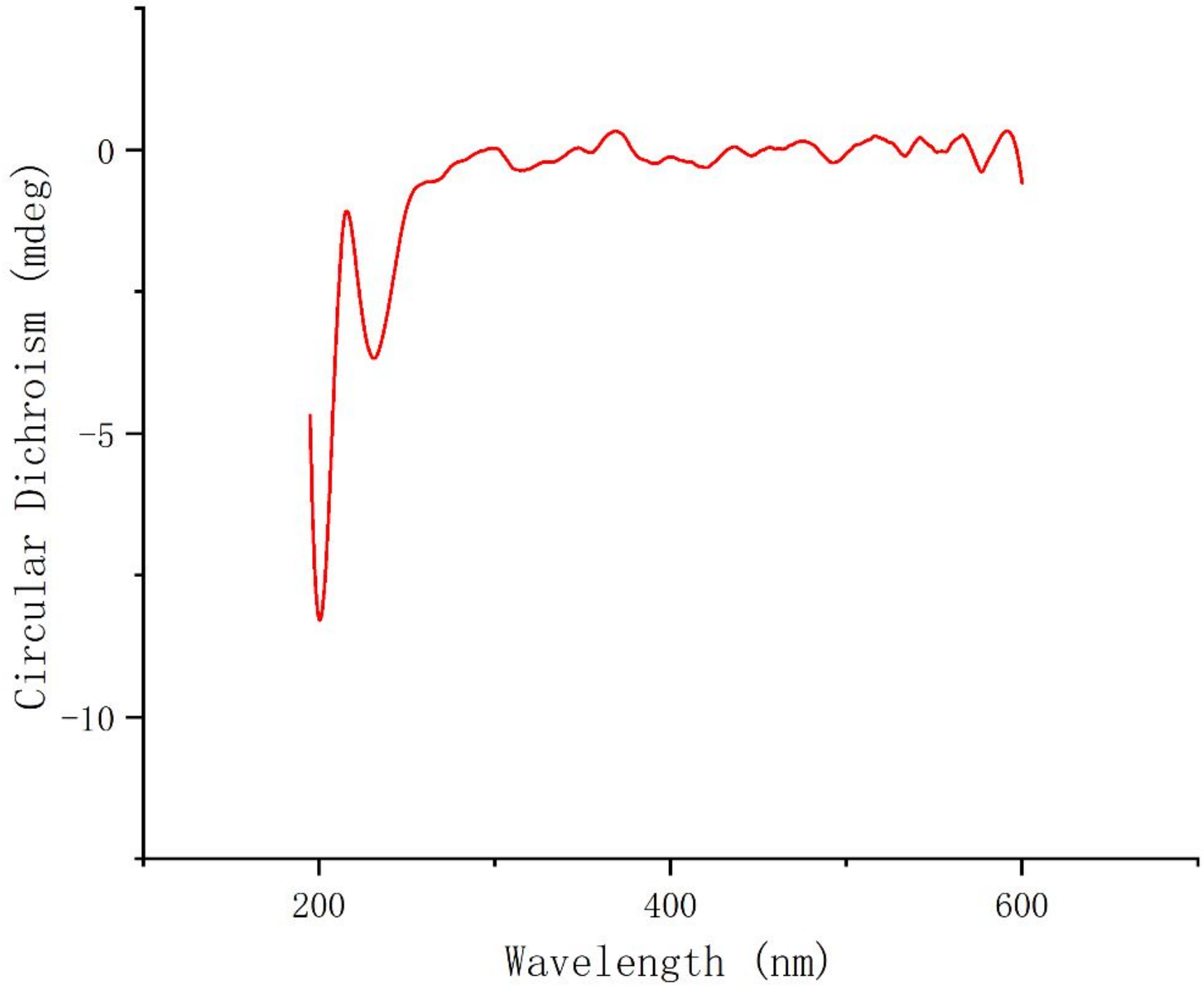


**Figure S129.** ECD spectrum of **13**

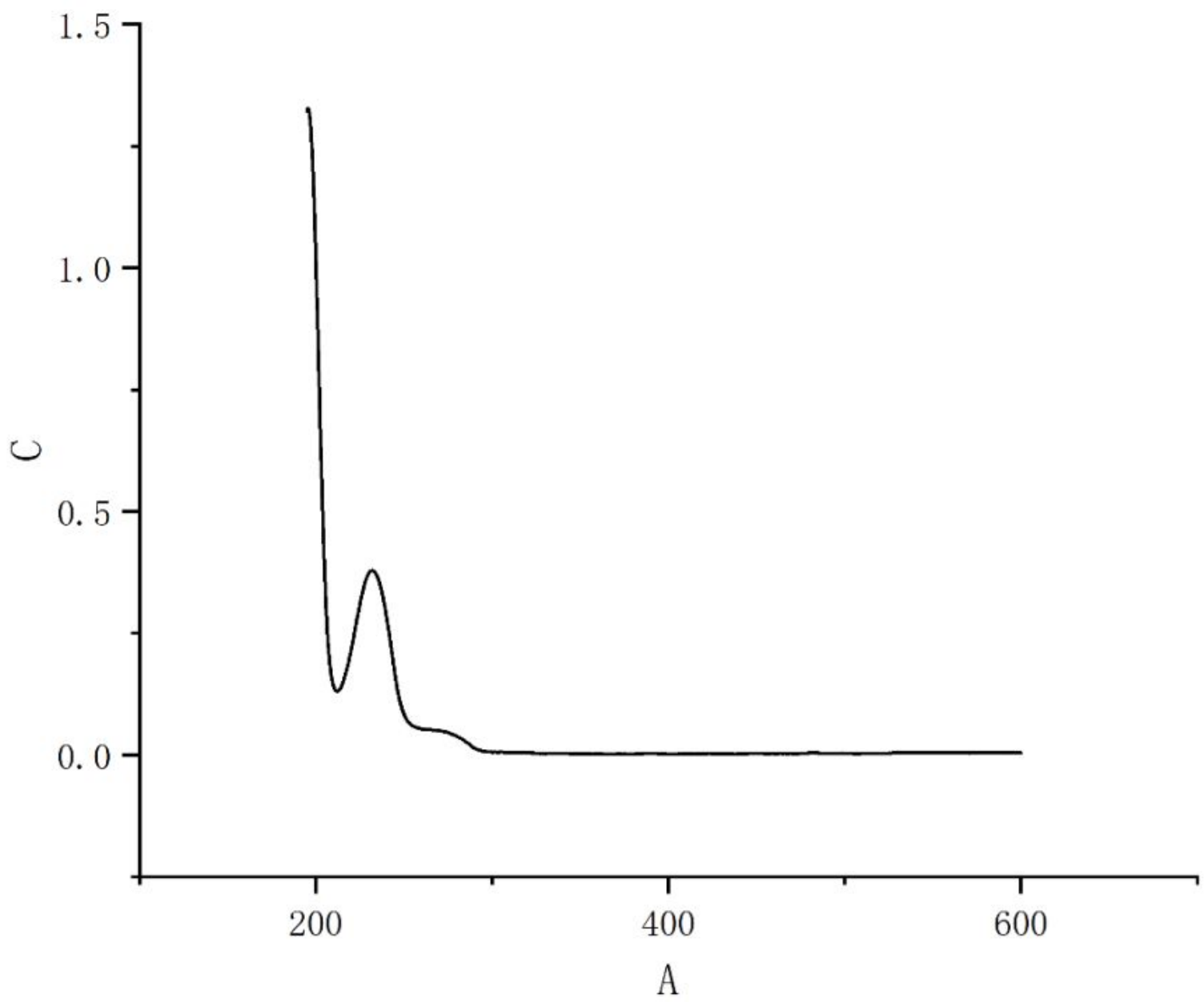


**Figure S130.** UV spectrum of **13**

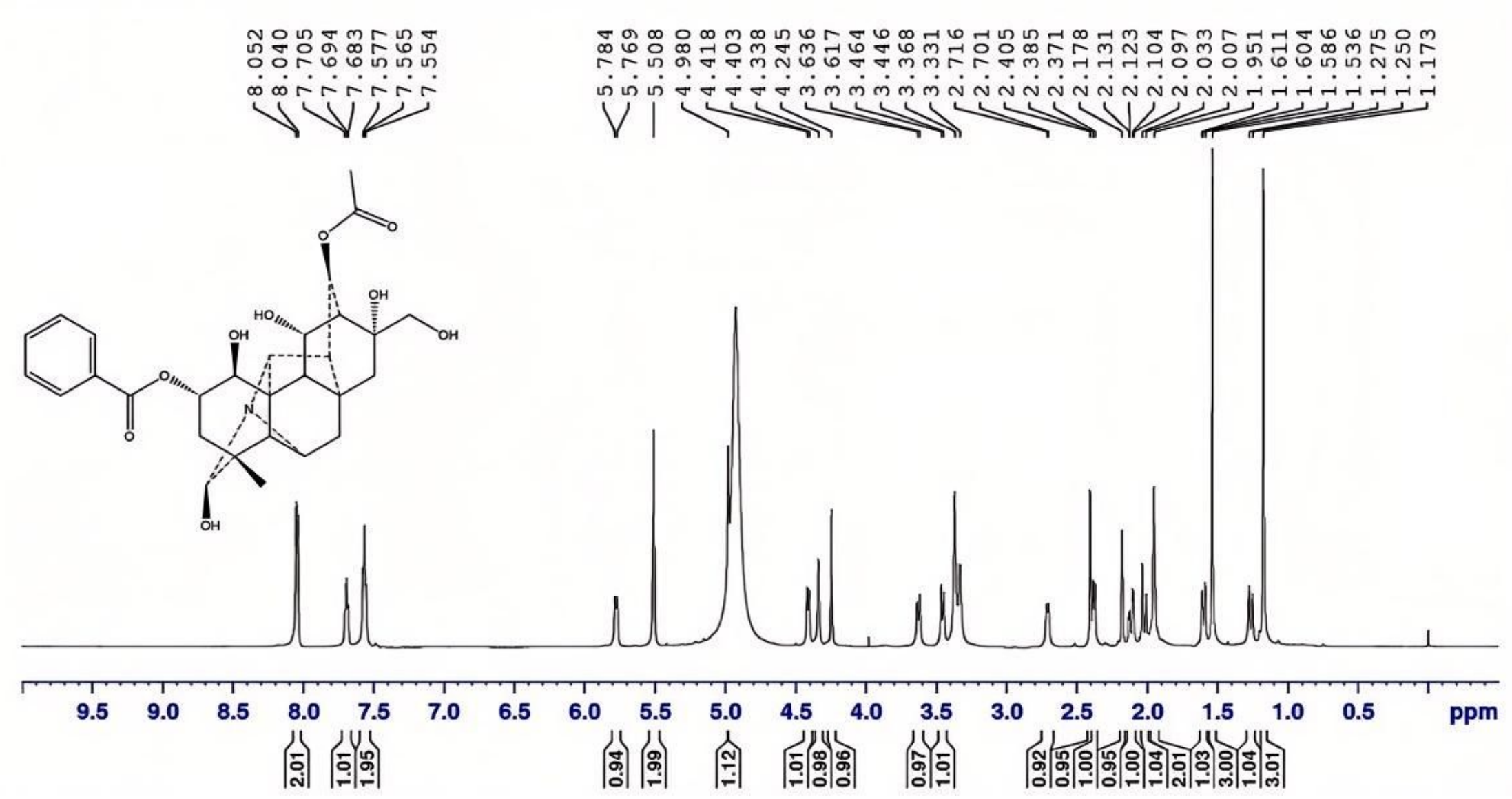

**Figure S131.** $^{1}H$ NMR spectrum of **14** in methanol-$d_4$

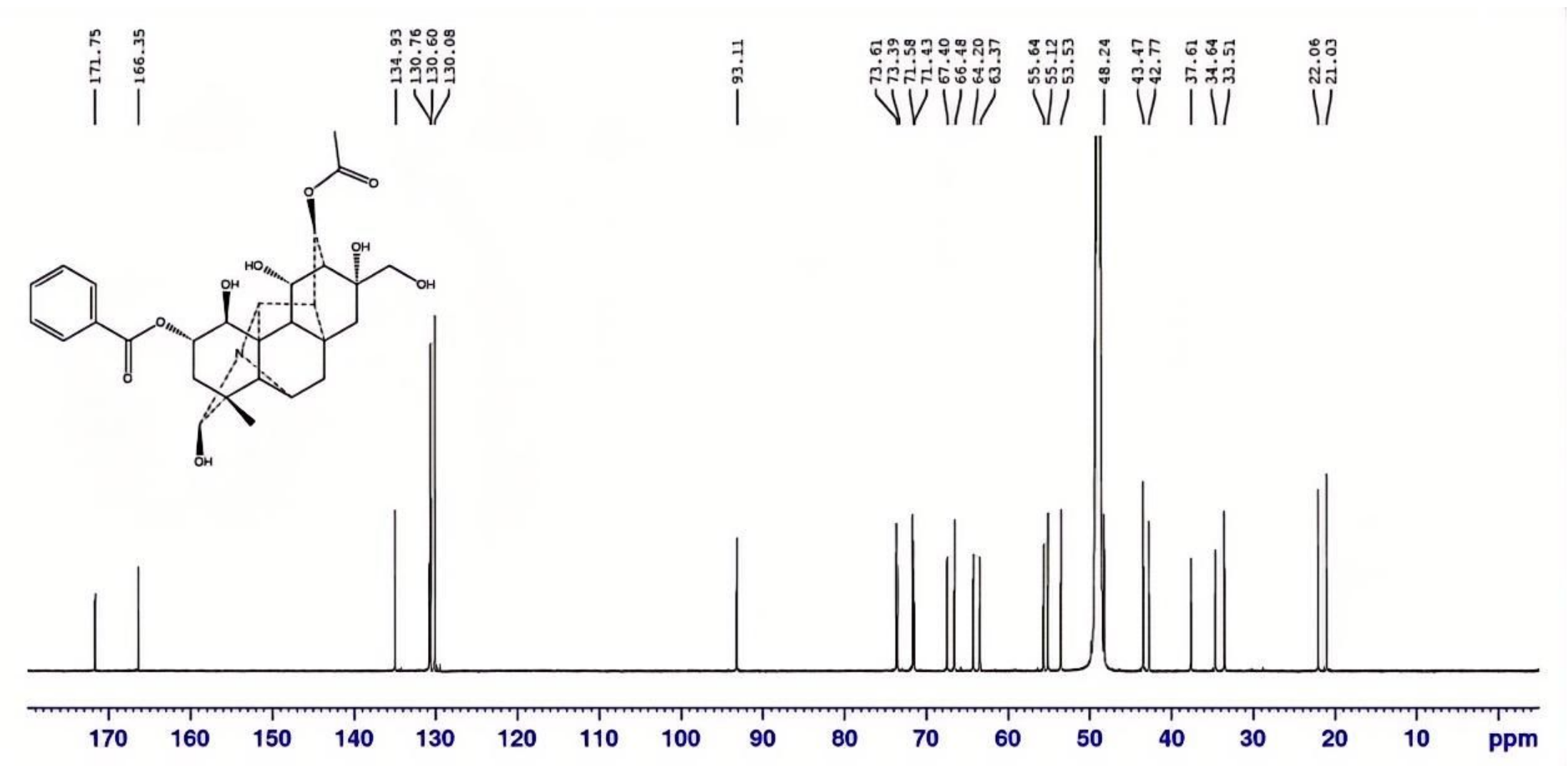

**Figure S132.** $^{13}C$ NMR spectrum of **14** in methanol-$d_4$

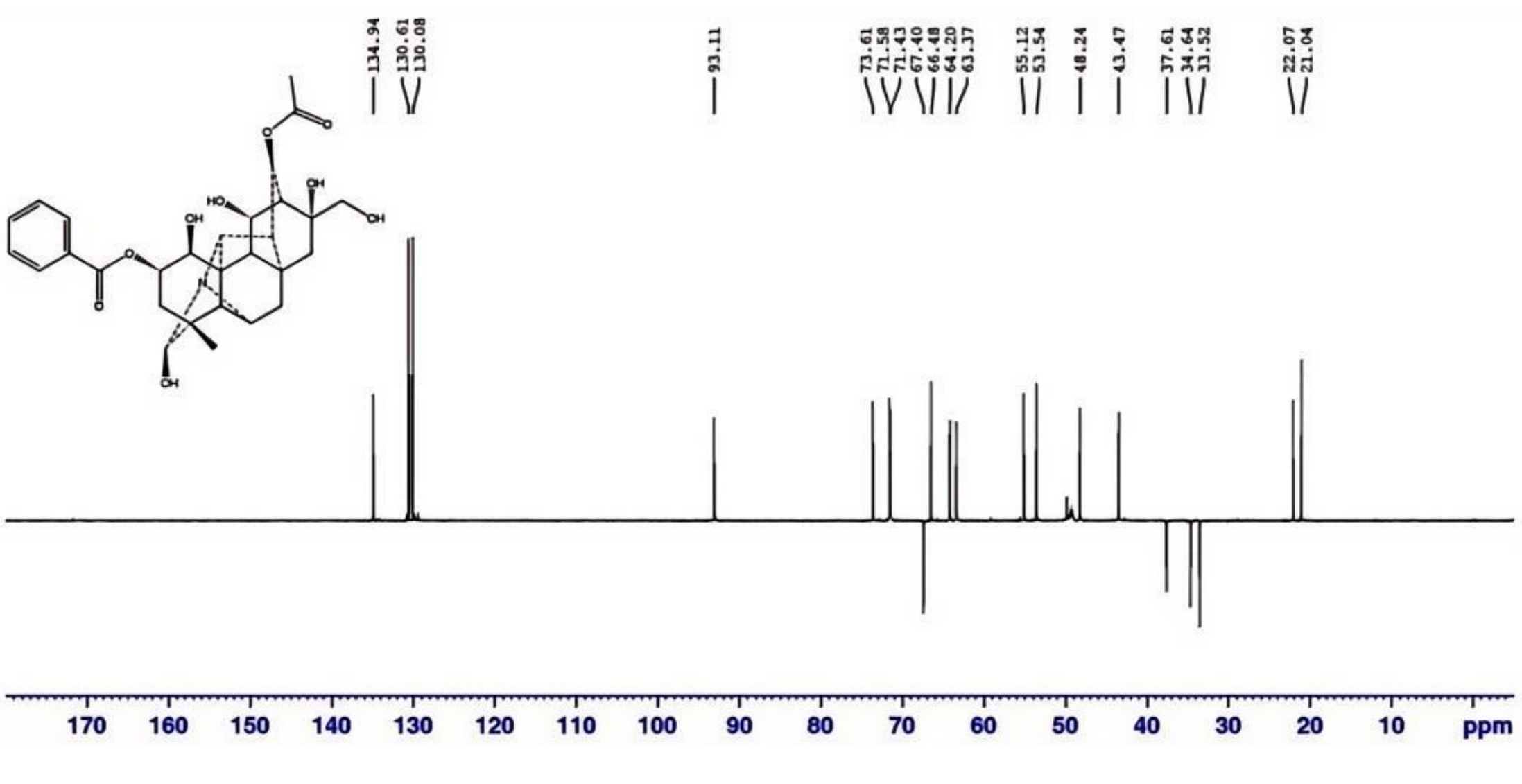

**Figure S133.** DEPT NMR spectrum of **14** in methanol-$d_4$

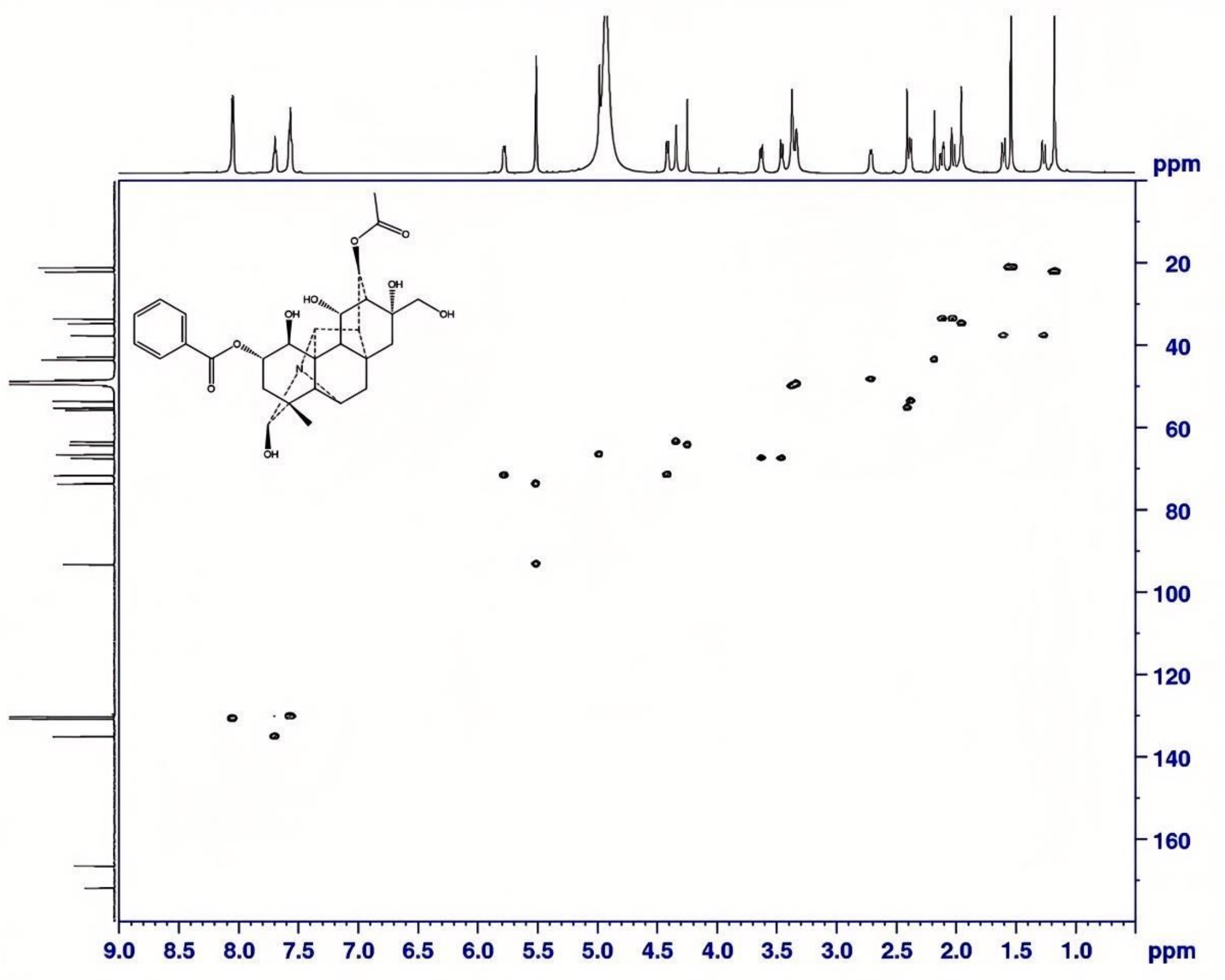

**Figure S134.** HSQC spectrum of **14**

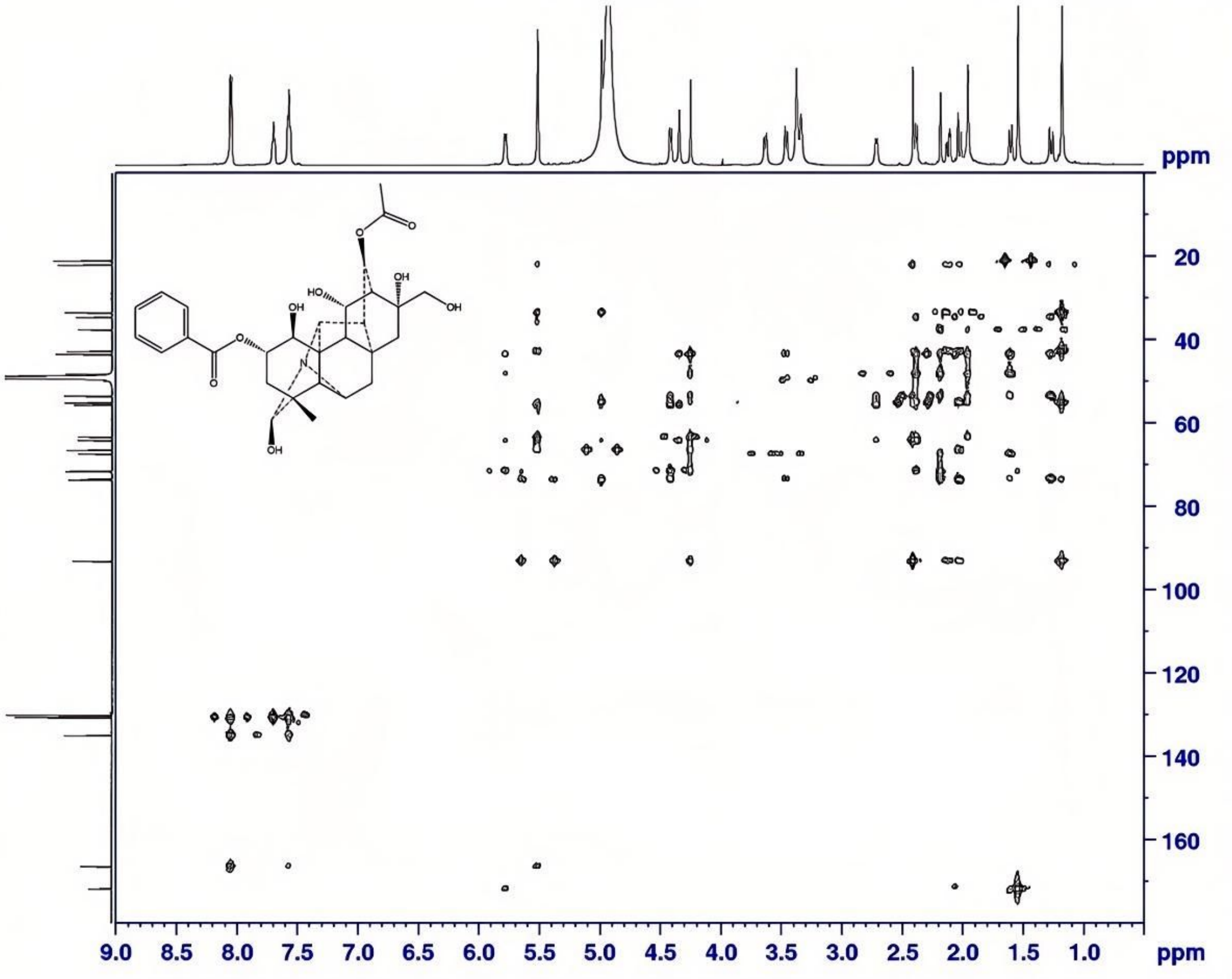


**Figure S135.** HMBC spectrum of **14**

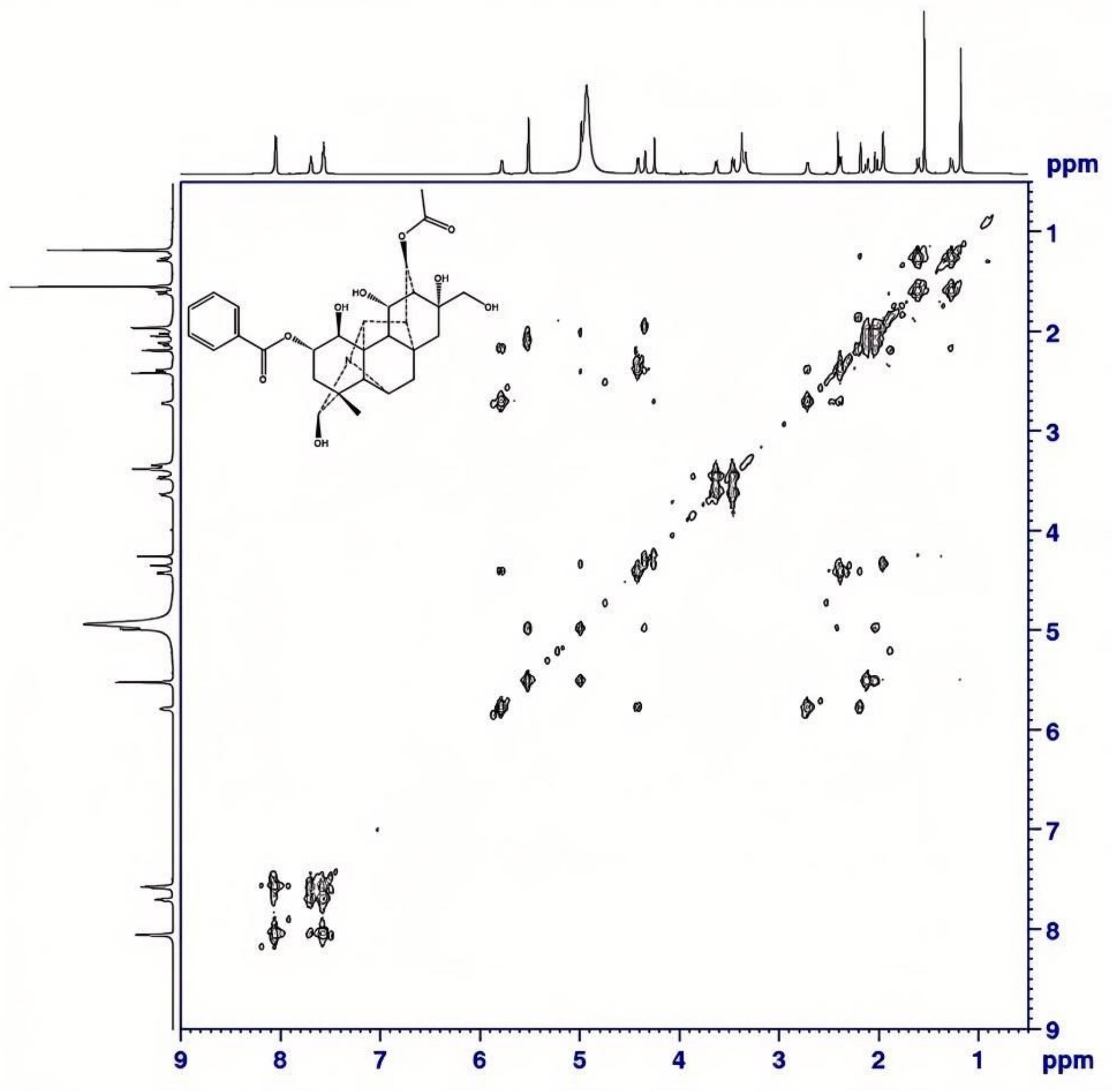

**Figure S136.** $^{1}H$-$^{1}H$ COSY spectrum of **14**

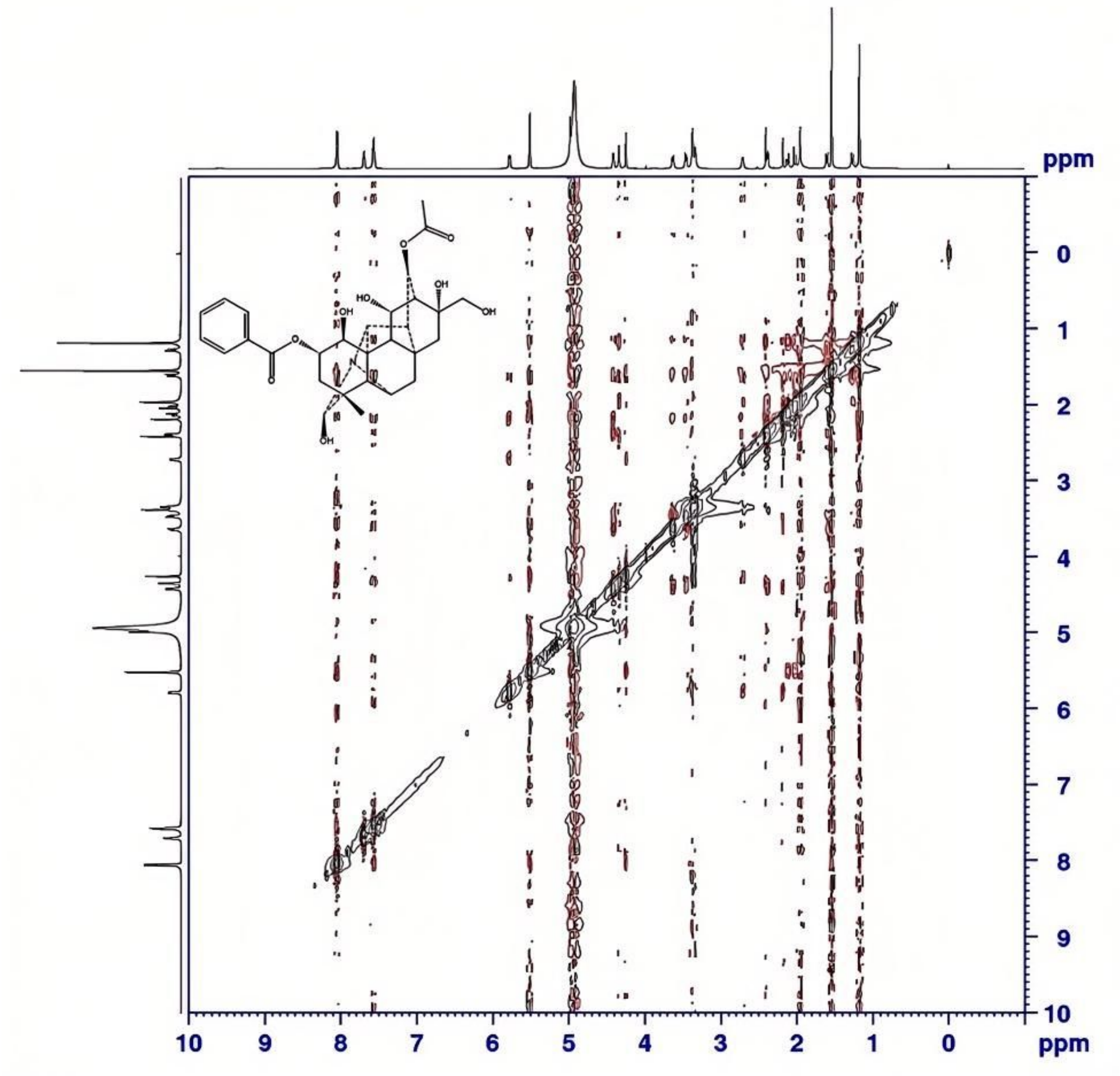

**Figure S137.** NOESY spectrum of **14**

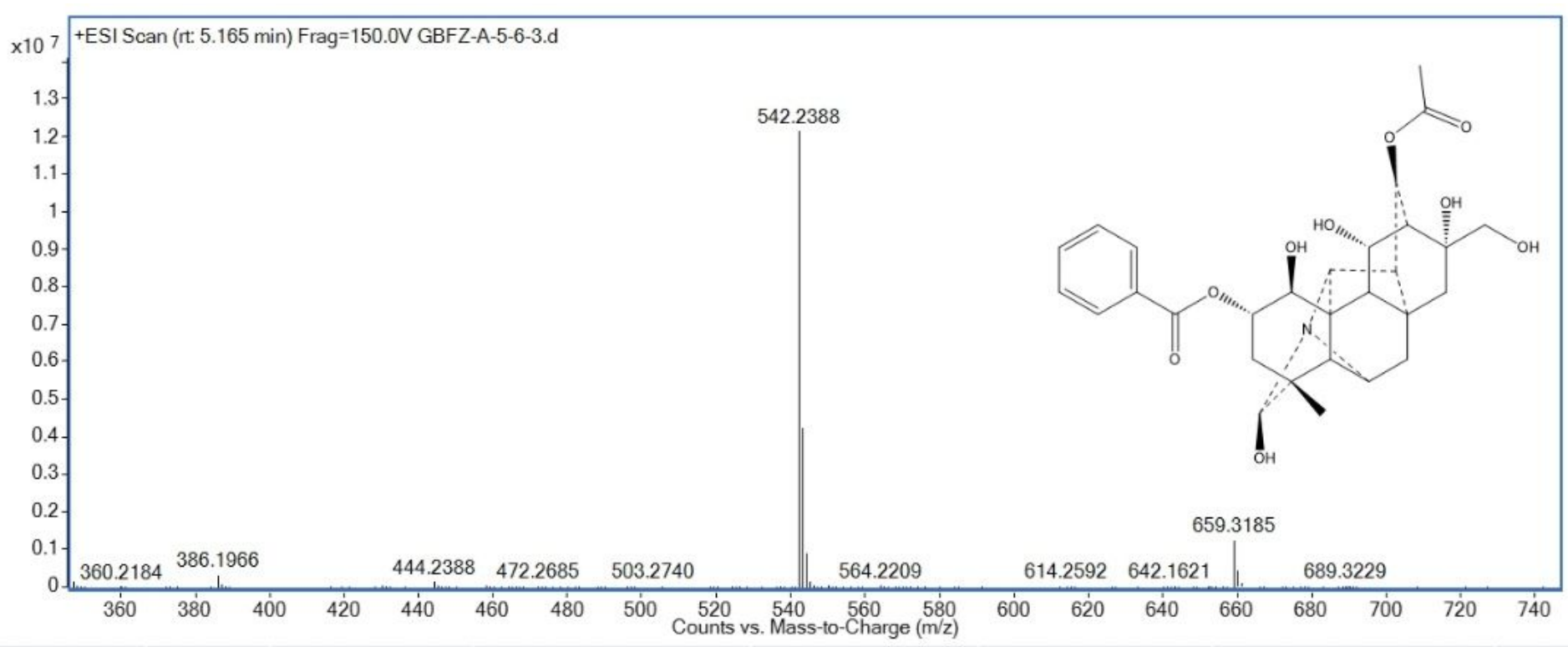


| Formula | Score | Mass | Mass (MFG) | m/z (Calc) | Diff (ppm) | Ion Formula | m/z |
|---|---|---|---|---|---|---|---|
| $C_{29}H_{35}NO_9$ | 99.78 | 541.2315 | 541.2312 | 542.2385 | -0.63 | $C_{29}H_{36}NO_9$ | 542.2388 |
| $C_{29}H_{35}NO_9$ | 99.54 | 541.2317 | 541.2312 | 564.2204 | -0.92 | $C_{29}H_{35}NO_9Na$ | 564.2209 |

**Figure S138.** HRESIMS spectrum of **14**

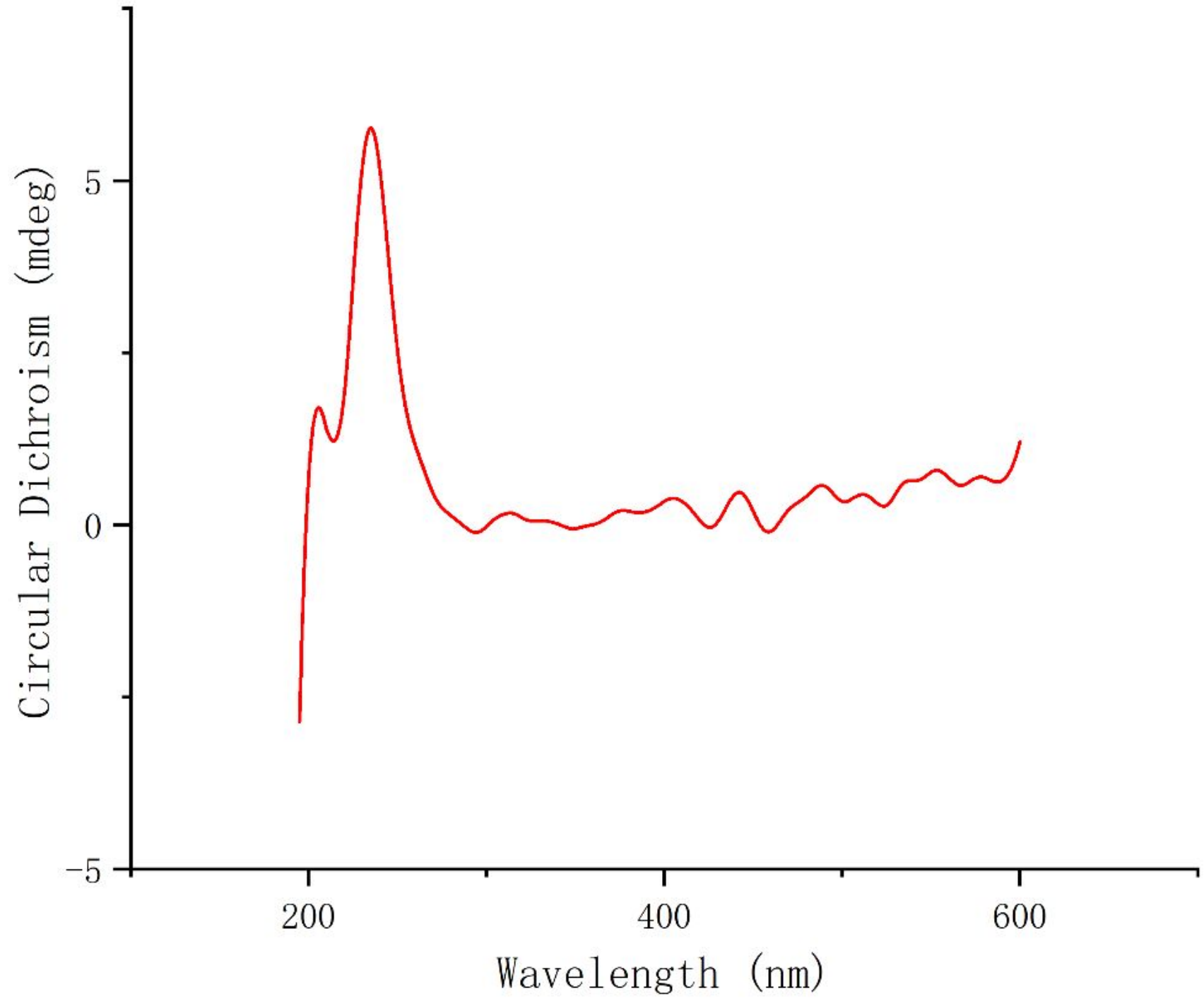


**Figure S139.** ECD spectrum of **14**

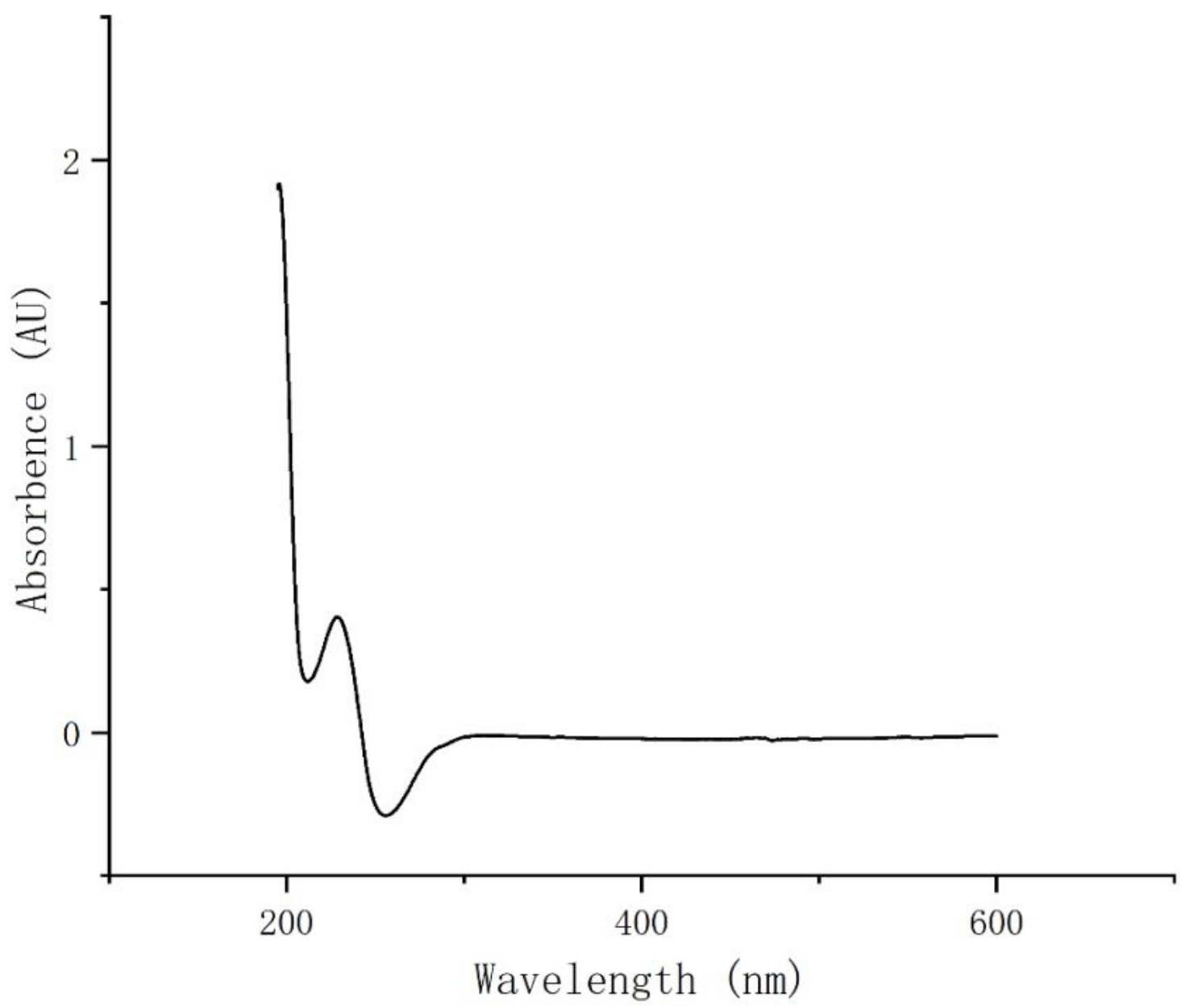


**Figure S140.** UV spectrum of **14**

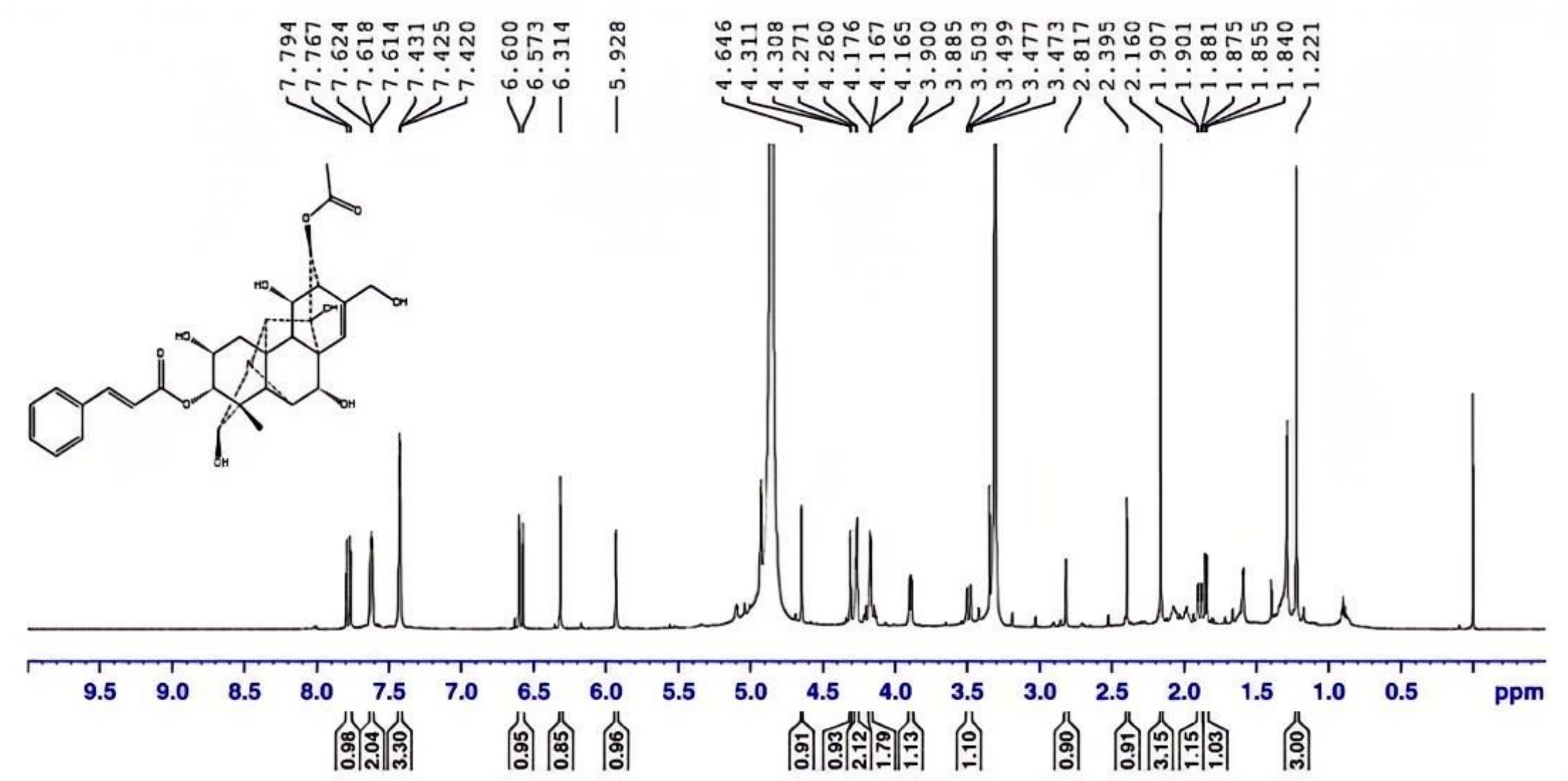

**Figure S141.** $^{1}$H NMR spectrum of **15** in methanol-$d_4$

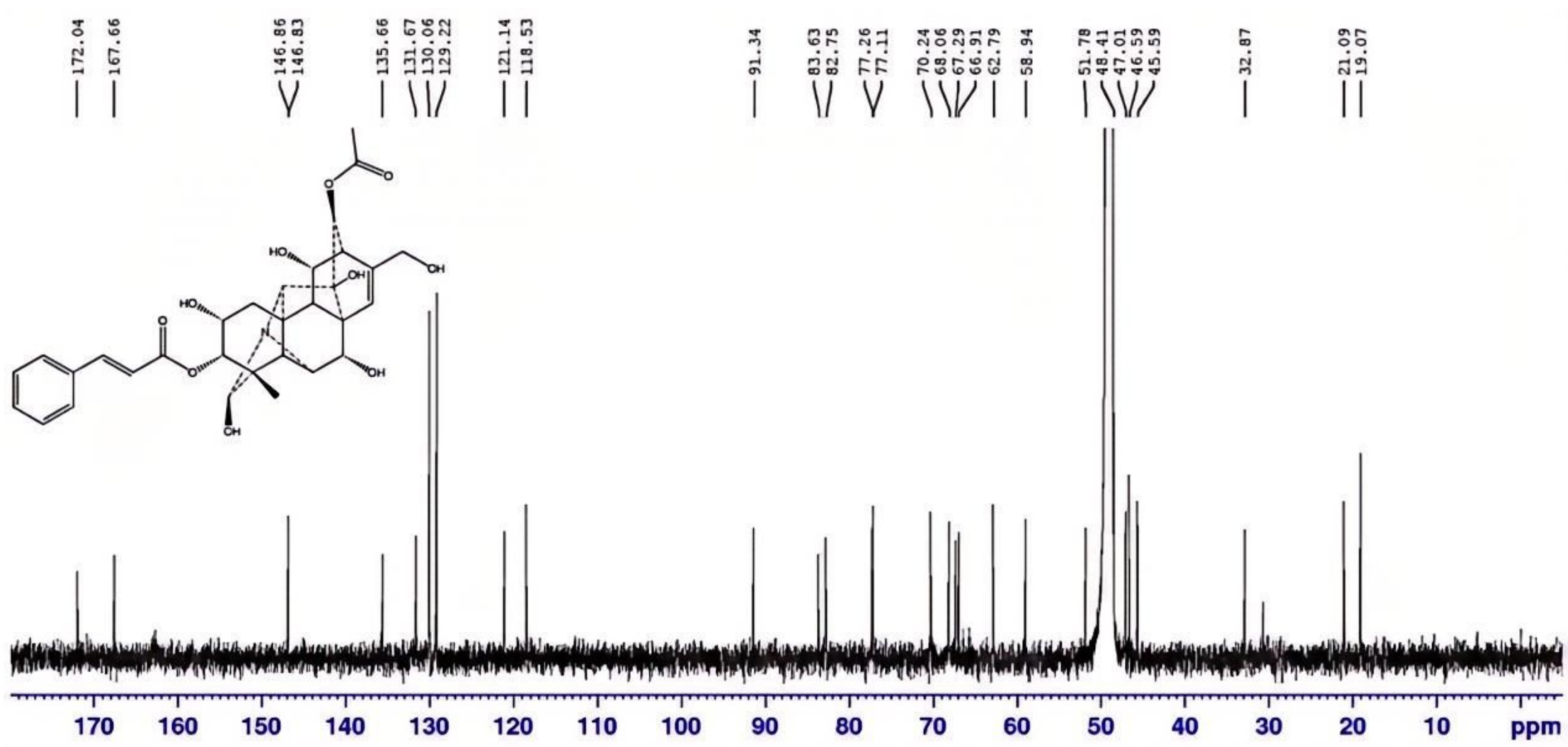

**Figure S142.** $^{13}$C NMR spectrum of **15** in methanol-$d_4$

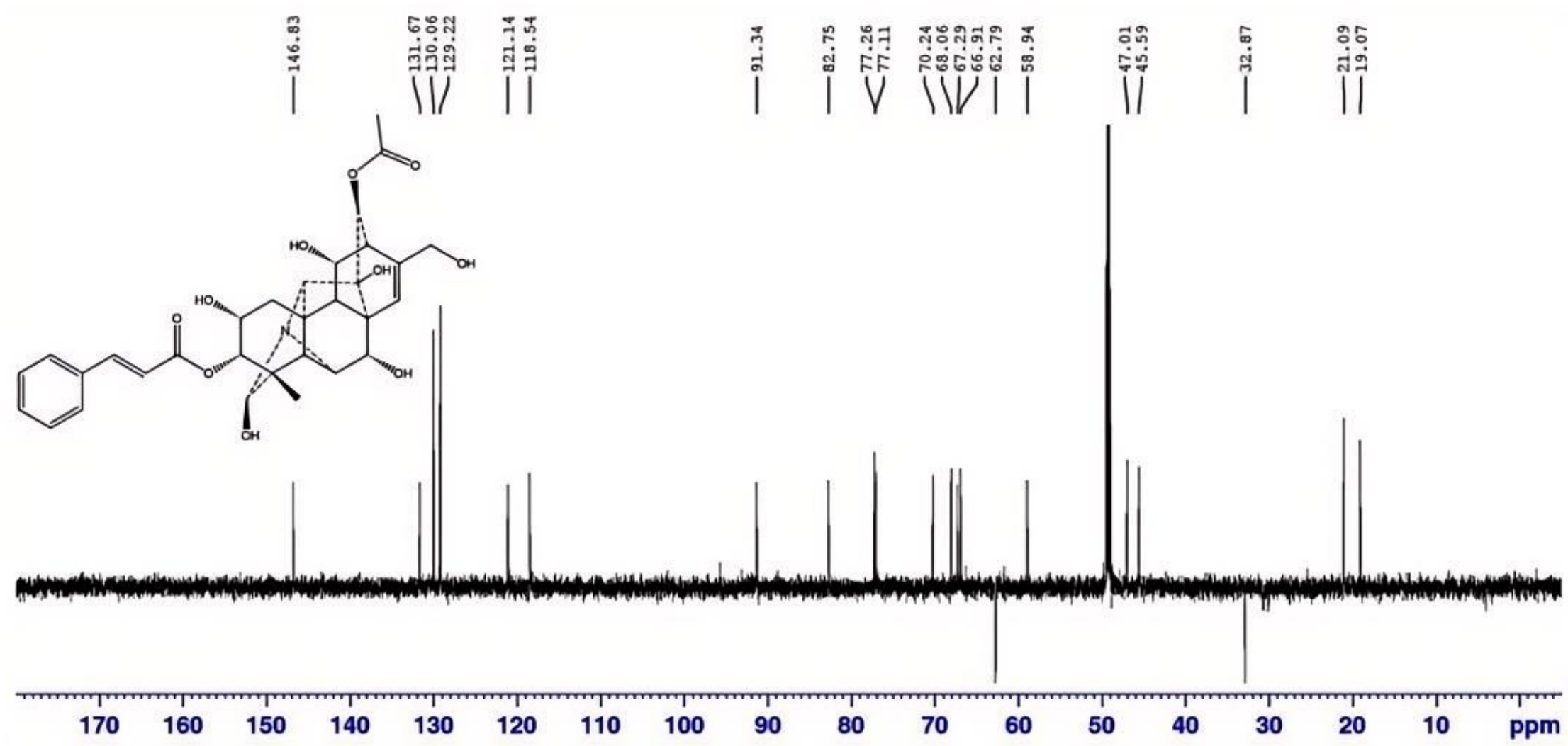

**Figure S143.** DEPT NMR spectrum of **15** in methanol-$d_4$

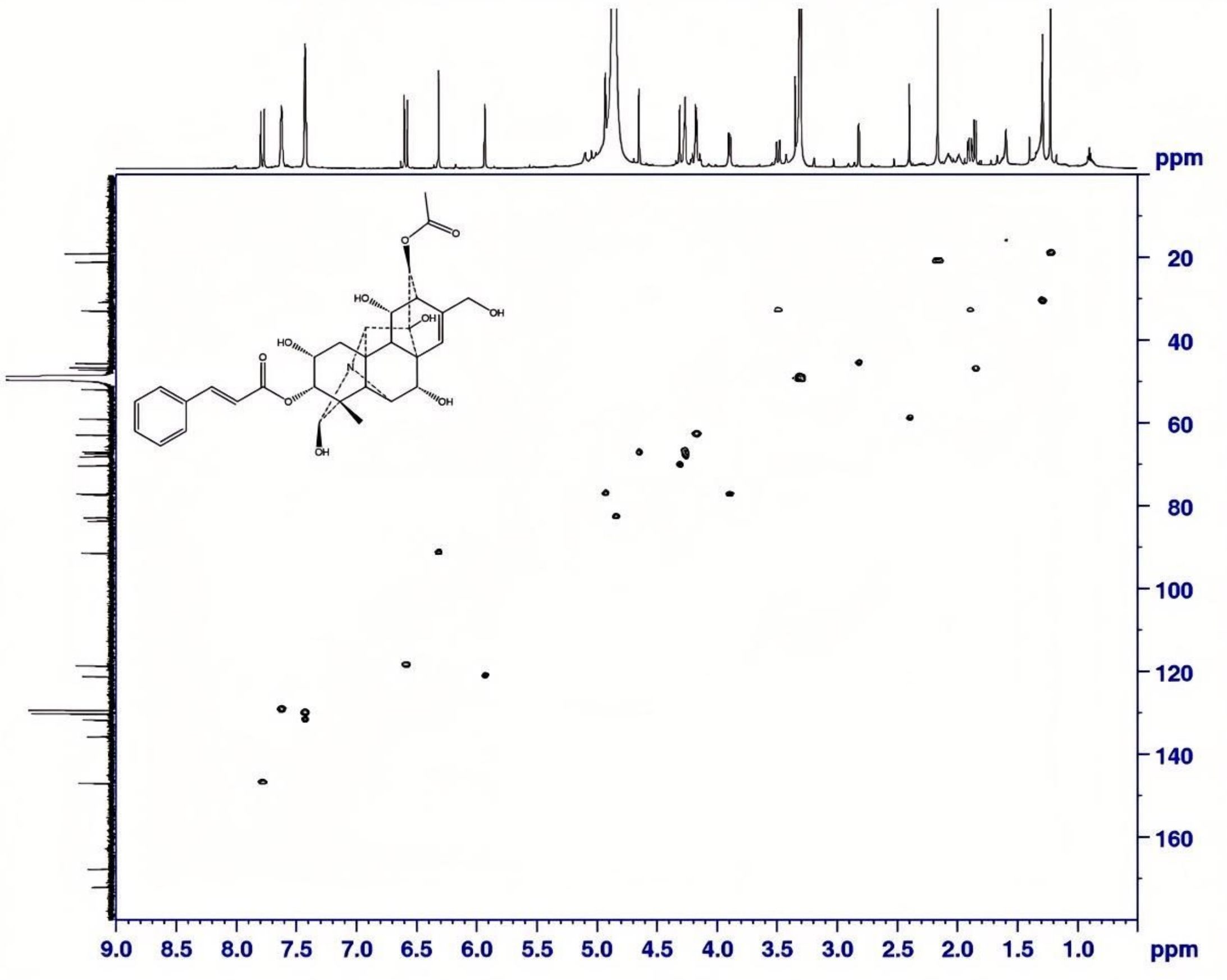


**Figure S144.** HSQC spectrum of **15**

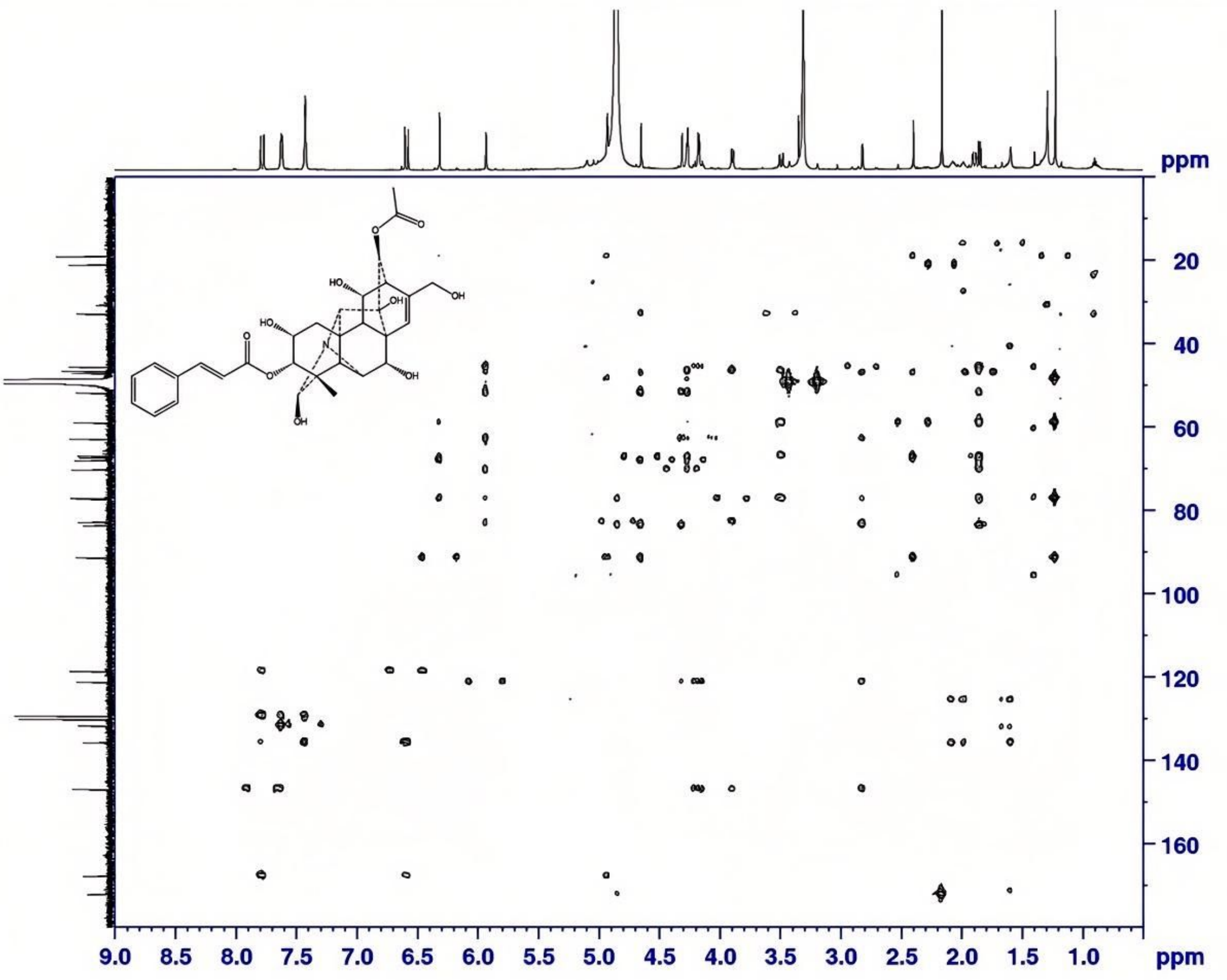


**Figure S145.** HMBC spectrum of **15**

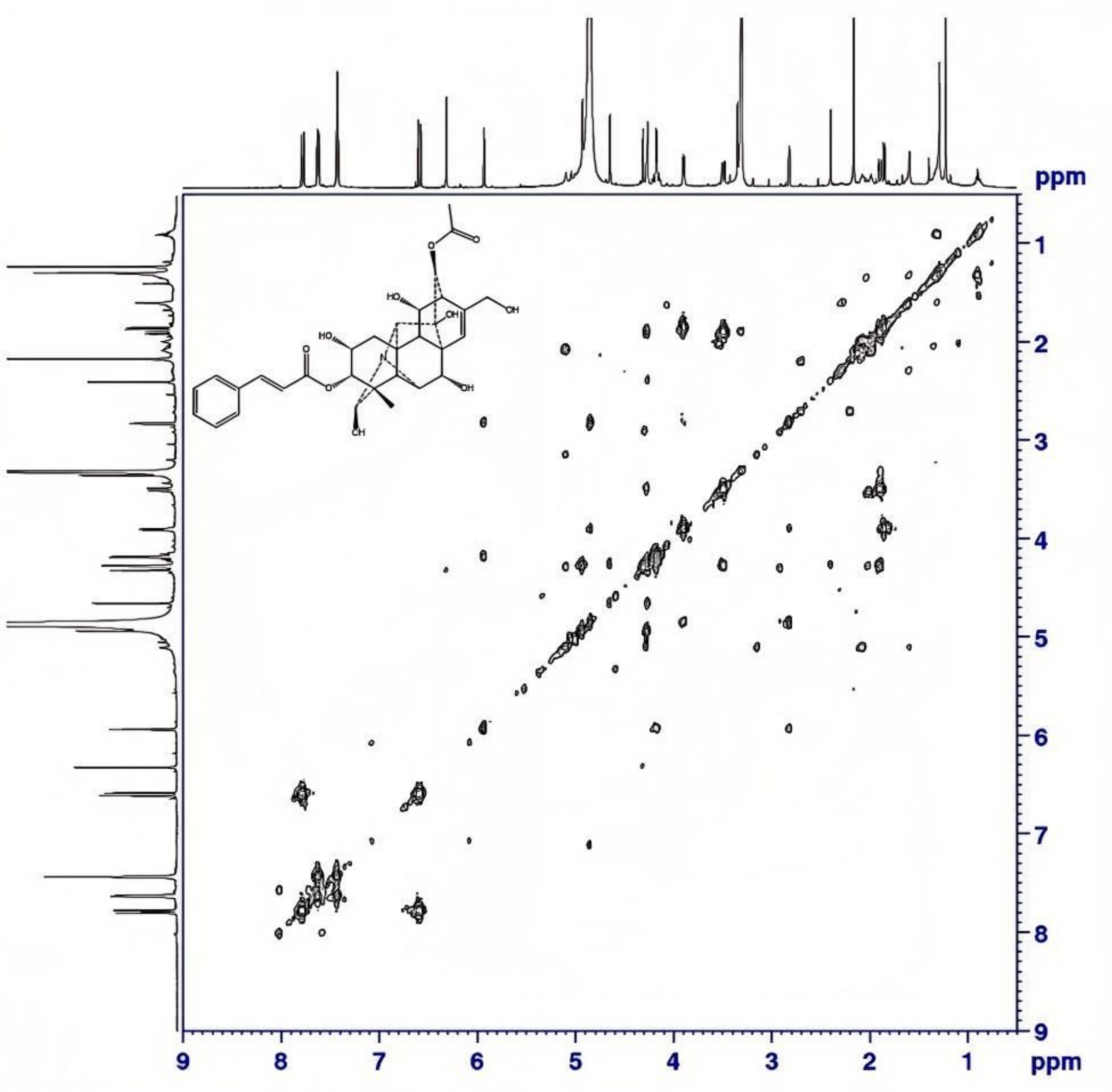


**Figure S146.** $^1H$-$^1H$ COSY spectrum of **15**

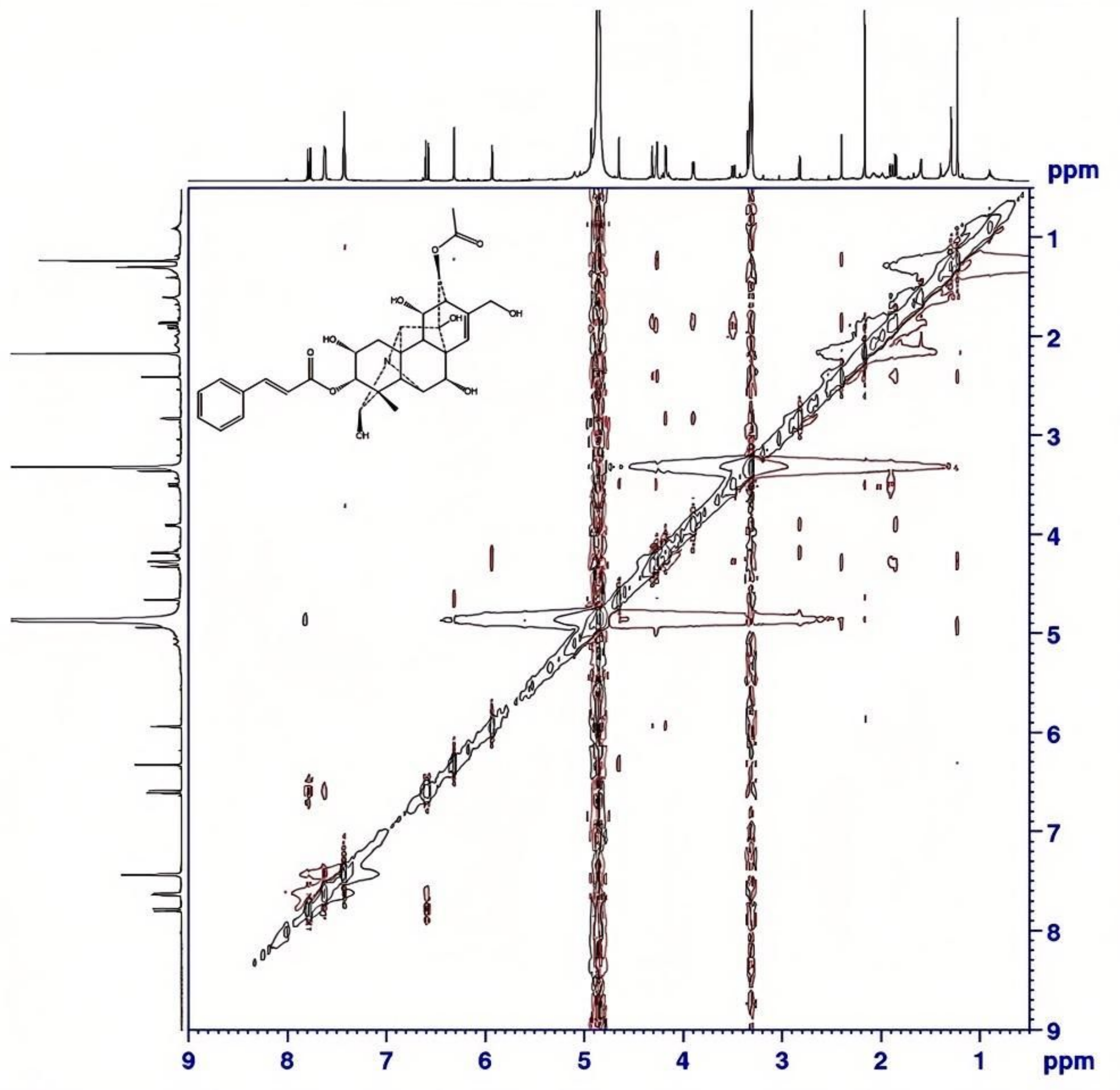

Figure S147. NOESY spectrum of **15**

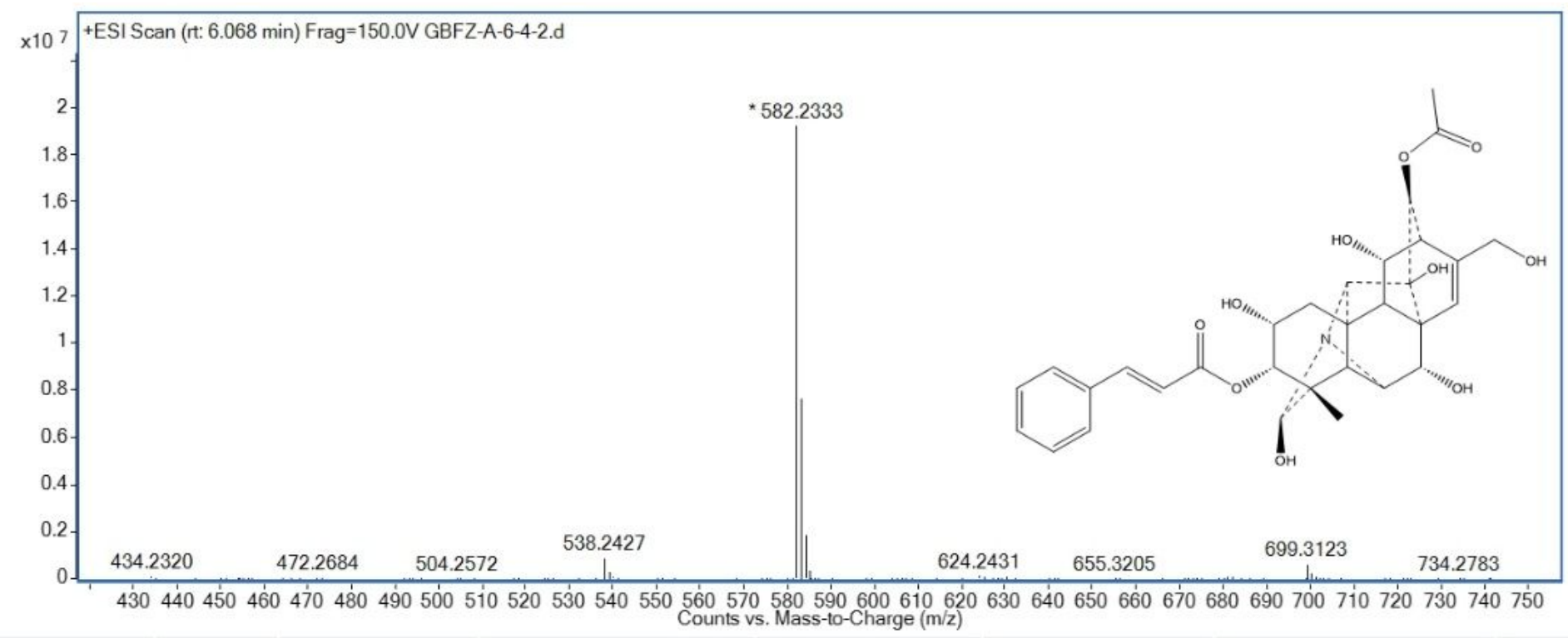

| Formula | Score | Mass | Mass (MFG) | m/z (Calc) | Diff (ppm) | Ion Formula | m/z |
|---|---|---|---|---|---|---|---|
| $C_{31}H_{35}NO_{10}$ | 99.99 | 581.2260 | 581.2261 | 582.2334 | 0.13 | $C_{31}H_{36}NO_{10}$ | 582.2333 |

**Figure S148.** HRESIMS spectrum of **15**

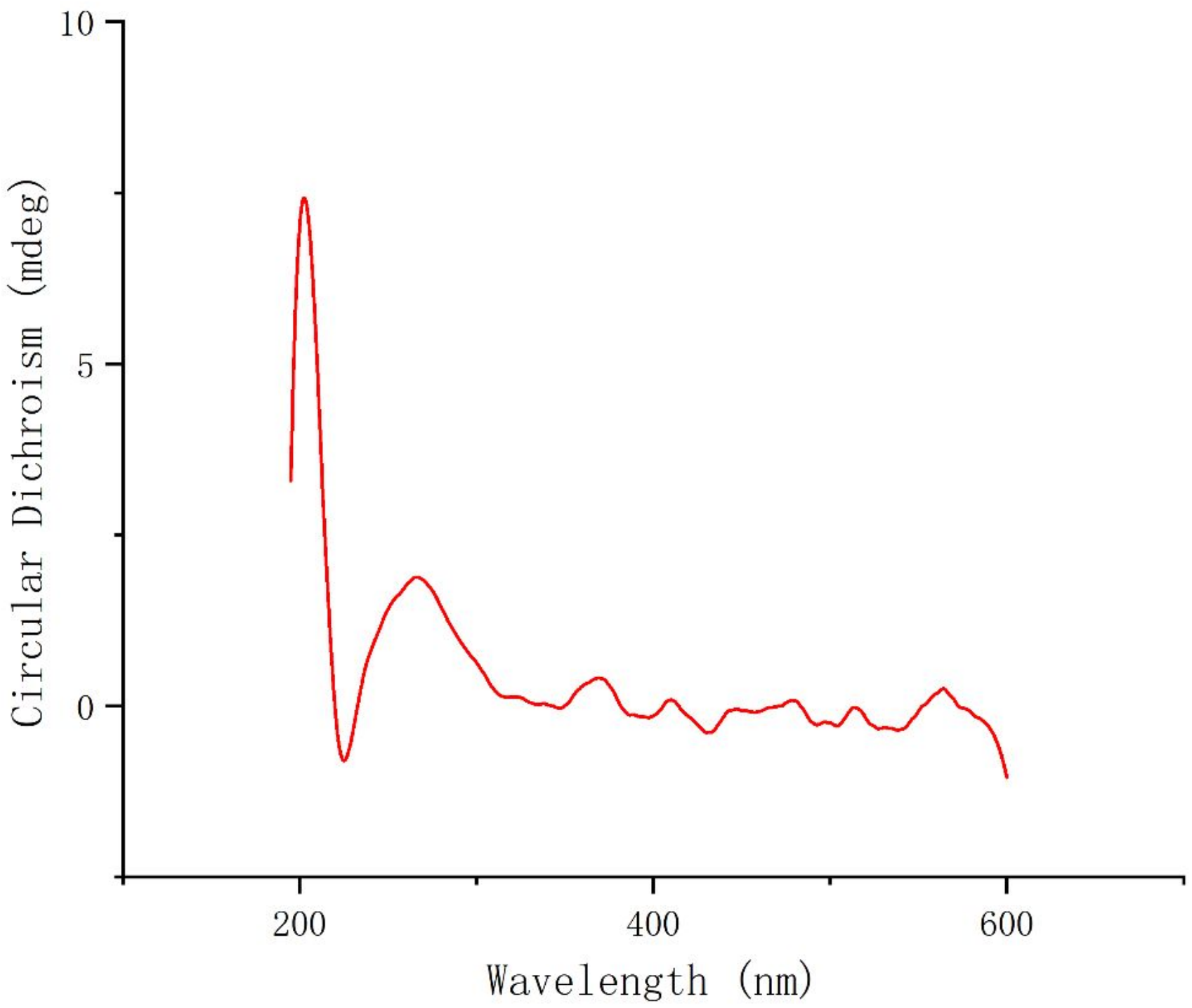


**Figure S149.** ECD spectrum of **15**

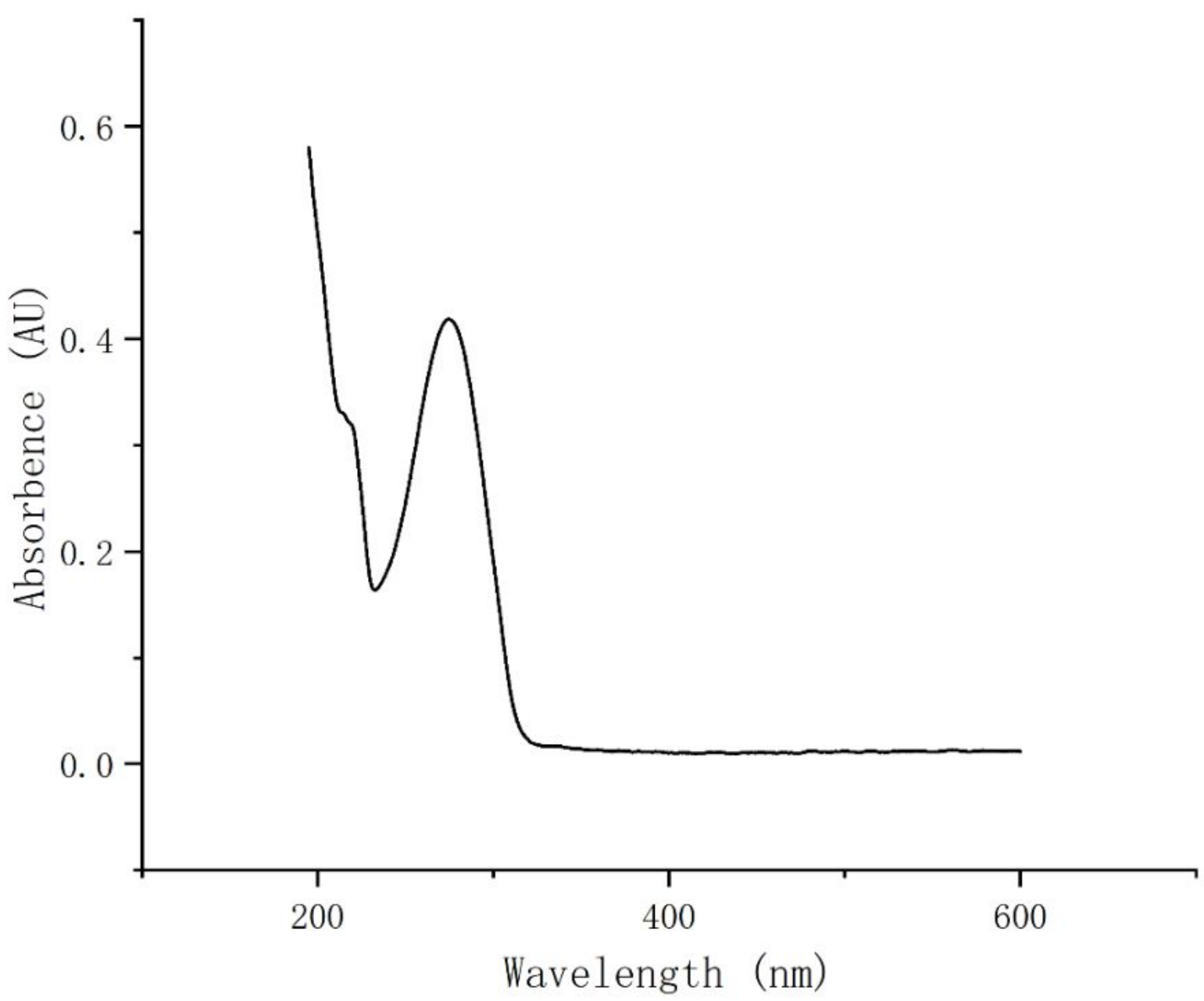


**Figure S150.** UV spectrum of **15**

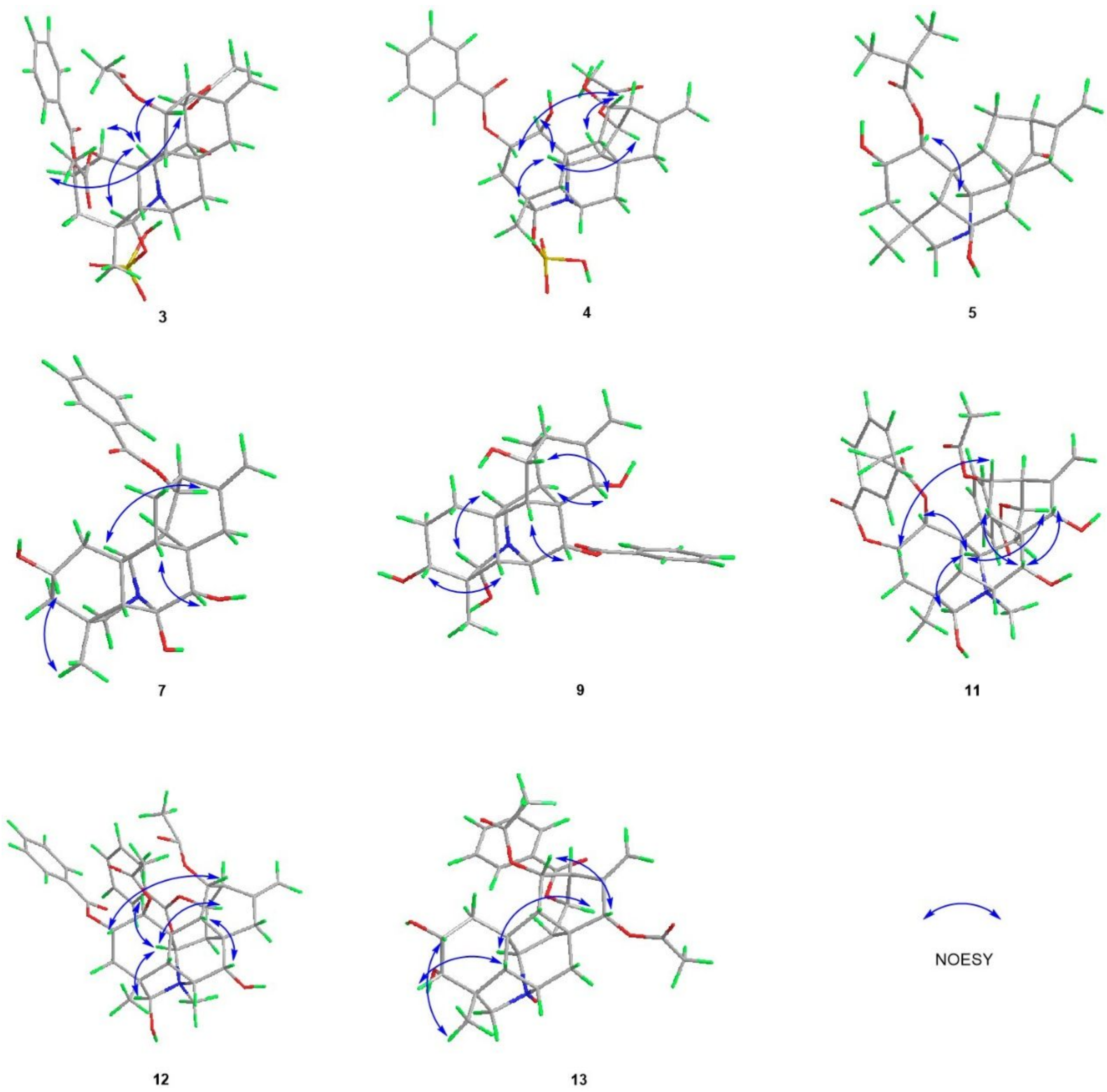


**Figure S151.** Key NOESY correlations of compounds **3**, **4**, **5**, **7**, **9**, **11**, **12**, **13**

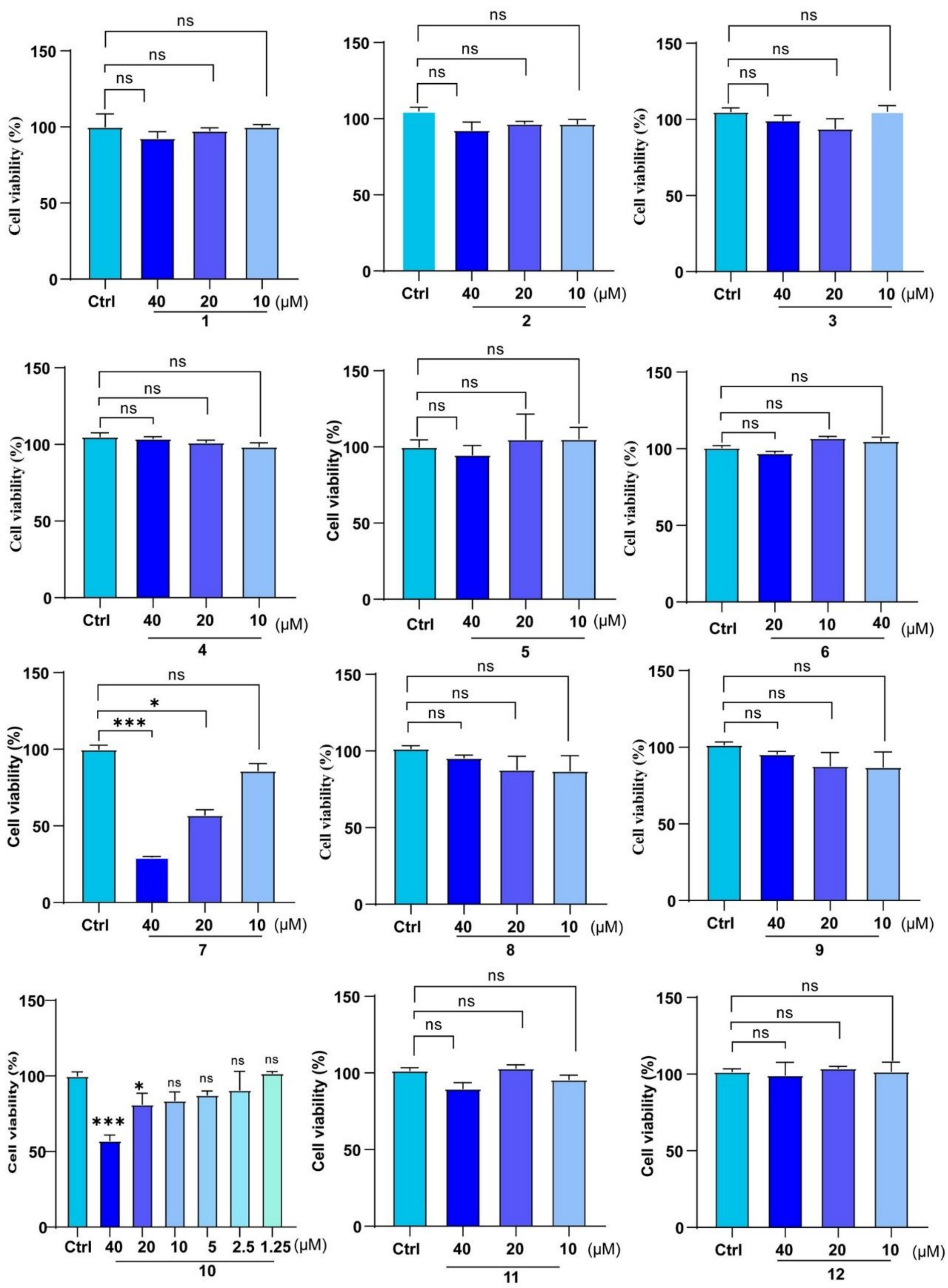


**Figure S152.** Cytotoxicity assay results of compounds **1−12** on MDA-MB-231 cell at a certain concentration

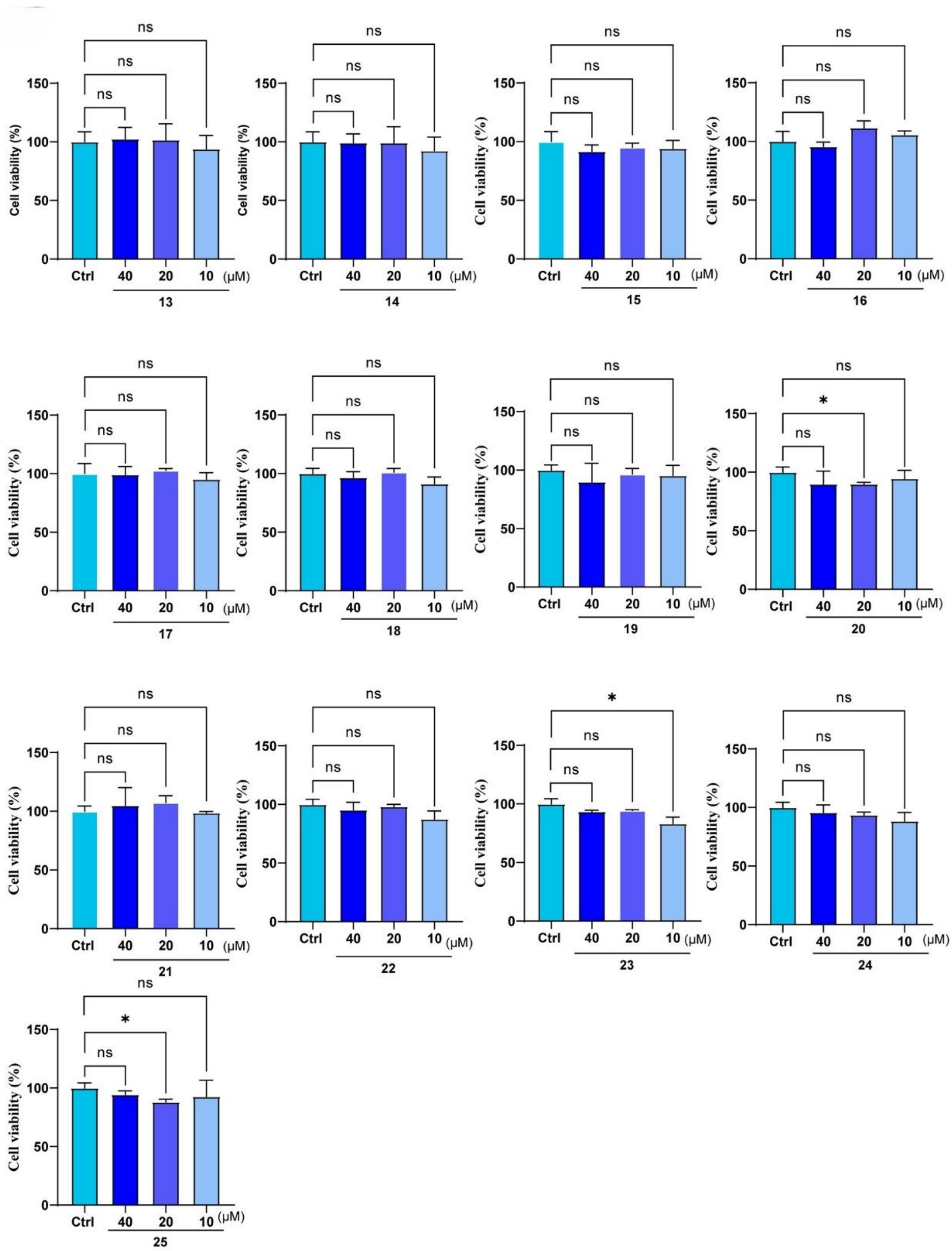


**Figure S153.** Cytotoxicity assay results of compounds **13–25** on MDA-MB-231 cell at a certain concentration
